# Synthesis and Biochemical Evaluation of Ethanoanthracenes and Related Compounds: Antiproliferative and Pro-Apoptotic Effects in Chronic Lymphocytic Leukemia (CLL)

**DOI:** 10.3390/ph17081034

**Published:** 2024-08-05

**Authors:** James P. McKeown, Andrew J. Byrne, Sandra A. Bright, Clara E. Charleton, Shubhangi Kandwal, Ivan Čmelo, Brendan Twamley, Anthony M. McElligott, Darren Fayne, Niamh M. O’Boyle, D. Clive Williams, Mary J. Meegan

**Affiliations:** 1 School of Pharmacy and Pharmaceutical Sciences, Panoz Institute, Trinity College, The University of Dublin, East End 4/5, Dublin 2, D02 PN40 Dublin, Irelandnioboyle@tcd.ie (N.M.O.); 2 School of Pharmacy and Pharmaceutical Sciences, Trinity Biomedical Sciences Institute, Trinity College Dublin, 152-160 Pearse St, Dublin 2, D02 R590 Dublin, Ireland; 3 School of Biochemistry and Immunology, Trinity Biomedical Sciences Institute, Trinity College Dublin, 152-160 Pearse St, Dublin 2, D02 R590 Dublin, Irelandclive.williams@tcd.ie (D.C.W.); 4 Molecular Design Group, School of Biochemistry and Immunology, Trinity Biomedical Sciences Institute, Trinity College Dublin, 152-160 Pearse St, Dublin 2, D02 R590 Dublin, Ireland; 5 Molecular Design Group, School of Chemical Sciences, Dublin City University, Glasnevin, D09 V209 Dublin, Ireland; 6 DCU Life Sciences Institute, Dublin City University, Glasnevin, D09 V209 Dublin, Ireland; 7 School of Chemistry, Trinity College Dublin, Dublin 2, D02 P3X2 Dublin, Ireland; 8 Discipline of Haematology, School of Medicine, Trinity Translational Medicine Institute, St. James’s Hospital and Trinity College, Dublin 8, D08 W9RT Dublin, Ireland; tony.mcelligott@tcd.ie

**Keywords:** ethanoanthracene, chalcone, B cell, chronic lymphocytic leukemia (CLL), antiproliferative, pro-apoptotic

## Abstract

Chronic lymphocytic leukemia (CLL) is a malignancy of mature B cells, and it is the most frequent form of leukemia diagnosed in Western countries. It is characterized by the proliferation and accumulation of neoplastic B lymphocytes in the blood, lymph nodes, bone marrow and spleen. We report the synthesis and antiproliferative effects of a series of novel ethanoanthracene compounds in CLL cell lines. Structural modifications were achieved via the Diels–Alder reaction of 9-(2-nitrovinyl)anthracene and 3-(anthracen-9-yl)-1-arylprop-2-en-1-ones (anthracene chalcones) with dienophiles, including maleic anhydride and *N*-substituted maleimides, to afford a series of 9-(*E*)-(2-nitrovinyl)-9,10-dihydro-9,10-[3,4]epipyrroloanthracene-12,14-diones, 9-(*E*)-3-oxo-3-phenylprop-1-en-1-yl)-9,10-dihydro-9,10-[3,4]epipyrroloanthracene-12,14-diones and related compounds. Single-crystal X-ray analysis confirmed the structures of the novel ethanoanthracenes **23f**, **23h**, **24a**, **24g**, **25f** and **27**. The products were evaluated in HG-3 and PGA-1 CLL cell lines (representative of poor and good patient prognosis, respectively). The most potent compounds were identified as **20a**, **20f, 23a** and **25n** with IC_50_ values in the ranges of 0.17–2.69 µM (HG-3) and 0.35–1.97 µM (PGA-1). The pro-apoptotic effects of the potent compounds **20a**, **20f, 23a** and **25n** were demonstrated in CLL cell lines HG-3 (82–95%) and PGA-1 (87–97%) at 10 µM, with low toxicity (12–16%) observed in healthy-donor peripheral blood mononuclear cells (PBMCs) at concentrations representative of the compounds IC_50_ values for both the HG-3 and PGA-1 CLL cell lines. The antiproliferative effect of the selected compounds, **20a**, **20f, 23a** and **25n,** was mediated through ROS flux with a marked increase in cell viability upon pretreatment with the antioxidant NAC. **25n** also demonstrated sub-micromolar activity in the NCI 60 cancer cell line panel, with a mean GI_50_ value of 0.245 µM. This ethanoanthracene series of compounds offers potential for the further development of lead structures as novel chemotherapeutics to target CLL.

## 1. Introduction

Chronic lymphocytic leukemia (CLL) is a malignancy of mature B cells, and it is the most frequent form of leukemia diagnosed in Western countries. The incidence of CLL varies by race and geographical location [1,2], with Ireland having one of the highest incidence rates [3]. It is typically a slow-growing cancer, and it is characterized by the proliferation and accumulation of neoplastic B lymphocytes in the blood, lymph nodes, bone marrow and spleen. Traditional chemoimmunotherapy approaches have been the mainstay of CLL treatment. However, with an evolving understanding of the biology of CLL and the development of targeted therapies, the treatment of patients diagnosed with CLL has changed dramatically over the past 5–10 years [4,5,6,7].

**Figure 1:**
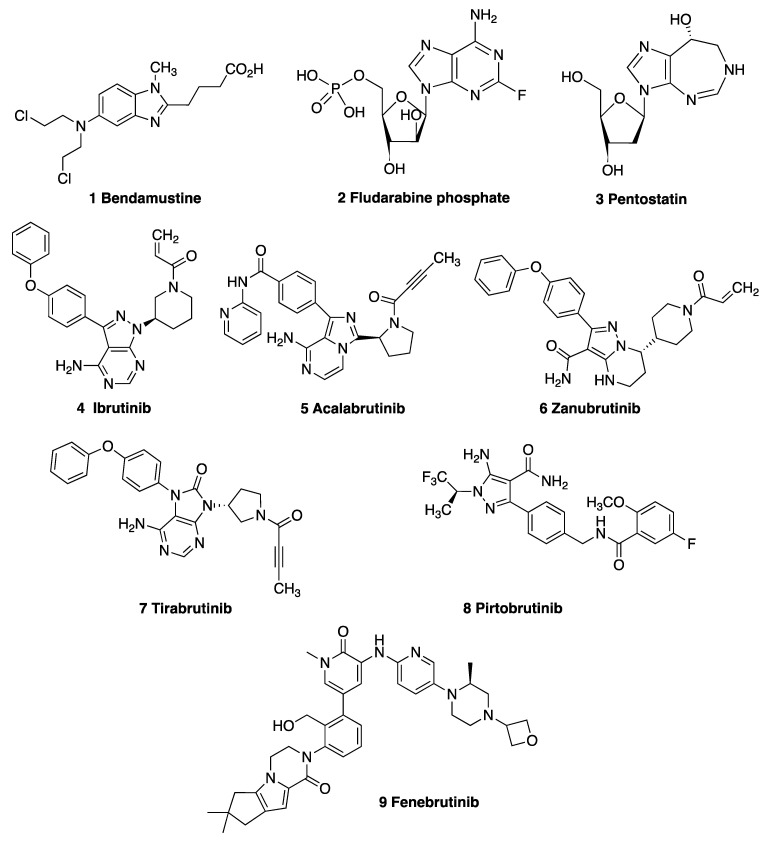
Drugs used in the treatment of CLL: alkylating agents **1** bendamustine, **2** fludarabine phosphate and **3** pentostatin; covalent BTK inhibitors **4** ibrutinib, **5** acalabrutinib, **6** zanubrutinib and **7** tirabrutinib; and non-covalent BTK inhibitors **8** pirtobrutinib and **9** fenebrutinib.

The most commonly used chemotherapy drugs approved by the Food and Drug Administration (FDA) for the treatment of CLL include the alkylating agents bendamustine **1** [8] and cyclophosphamide, together with the nucleoside fludarabine phosphate **2,** while the adenosine deaminase (ADA) inhibitor pentostatin **3** is also used in CLL patients who have relapsed (Figure 1). The development of small molecules targeting the B-cell receptor (BCR) signaling pathway has revolutionized the treatment of CLL [9]. Bruton’s tyrosine kinase (BTK) [10] and the phosphoinositide 3-kinase δ (PI3Kδ) isoform [11] are essential for BCR signaling [12]. The targeted orally active inhibitor ibrutinib **4** was the first kinase inhibitor to be approved for CLL treatment, and it is now used to treat patients with CLL regardless of their treatment history [13]. Ibrutinib **4** [14], acalabrutinib **5** [15], zanubrutinib **6** [16] and tirabrutinib **7** [17,18] interact with BTK to form a covalent bond with the cysteine residue Cys481, resulting in the inhibition of BTK activity. While these inhibitors have had a significant impact on the treatment of B-cell malignancies, acquired resistance frequently emerges in patients, leading to clinical relapse and disease progression [19]. Non-covalent inhibitors of BTK, such as pirtobrutinib **8** [20] and fenebrutinib **9** [21], are also effective in the treatment of CLL [22], and they have been evaluated in relapsed or refractory B-cell non-Hodgkin lymphoma (NHL) and CLL [23]. Many promising small-molecule, irreversible BTK inhibitors are under clinical evaluation in targeted cancer therapy development [24].

**Figure 2:**
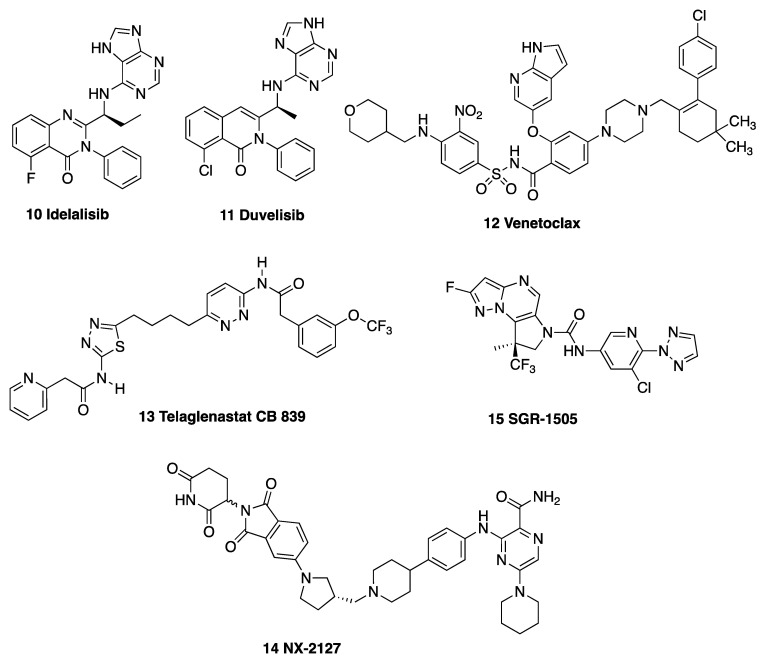
Drugs targeting CLL: PI3Kδ inhibitor idelalasilib **10**; PI3Kδ and PI3Kγ inhibitor duvelisib **11**; Bcl-2 inhibitor venetoclax **12**; glutaminase inhibitor telaglenastat CB 839 **13**; and dual BTK degrader NX-2127 **14** and MALT-1 inhibitor SGR-1505 **15**.

The PI3Kδ inhibitor idelalisib **10** is an inhibitor of B-cell receptor signaling, and it is useful in patients with 17p deletion or TP53 mutation, who usually have a poor outcome, and in relapsed CLL [25,26]. Duvelisib **11** is an inhibitor of PI3Kδ and PI3Kγ, and it has been used in the treatment of high-risk CLL patients [27]. Venetoclax **12** is an orally bioavailable selective inhibitor of the anti-apoptotic B-cell lymphoma 2 (Bcl-2) protein, and it provides targeted therapy for the treatment of CLL patients, with a high overall response (80%) [28,29] (Figure 2); however, the development of clinical resistance is associated with its use [30]. Additionally, immunotherapies are now successfully used in the clinical treatment of CLL, e.g., the CD20 targeting monoclonal antibodies including rituximab, obinutuzumab and ofatumumab [27,31]. The programmed cell death protein 1 (PD-1) blocking antibodies pembrolizumab and nivolumab [27] can provide selective efficacy for CLL patients with Richter’s transformation (RT), an aggressive complication of CLL [28]. Many of the oral agents are more tolerable than the traditional combination of chemotherapy and immunotherapy programs, and they are suitable for the treatment of older and more frail patients [32].

Small molecules discovered in preclinical development studies for CLL include the glutaminase inhibitor CB-839 (telaglenastat **13**) alone and in combinations with the widely used CLL drugs venetoclax, ibrutinib or MCL-1 inhibitor AZD-5991 [33]. The novel compound NX-2127 **14,** currently in Phase 1b clinical trials, combines the activity of a targeted BTK degrader with a second compound that degrades the Ikaros zinc finger transcription factors [34] (Figure 2). SGR-1505 **15** is an investigational mucosa-associated lymphoid tissue–lymphoma translocation protein 1 (MALT1) inhibitor, and it is currently in Phase 1 clinical development for refractory B-cell neoplasms [35]. The pre-clinical modeling of novel therapeutics in CLL has resulted in the discovery of new agents with potential for targeted therapies [36]. Recently reported molecules that target the BCR-associated kinases BTK, PI3K and spleen tyrosine kinase (Syk), together with inhibitors of Bcl-2, are currently either approved or in additional clinical trials [22,37].

**Figure 3:**
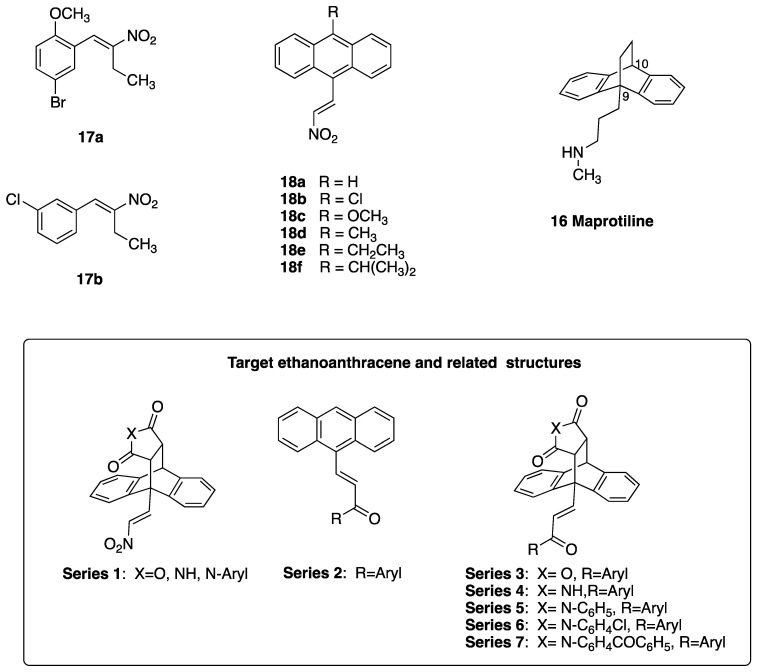
Nitrostyrenes **17a**, **17b**, nitrovinylanthracenes **18a–e** and maprotiline **16**; target ethanoanthracene structures, Series 1–7.

Previously, we identified a series of (*E*)-nitrostyrenes, e.g., **17a**, **17b** and (*E*)-9-(2-nitrovinyl)anthracenes **18a–e** (Figure 3), which potently reduced cell viability in the Burkitt’s lymphoma (BL) cell lines MUTU-I and DG-75 [38]. In CLL cells associated with a poor patient prognosis, they demonstrated IC_50_ values of <10 µM [38,39,40] and were significantly more potent than fludarabine phosphate [39]. Anti-cancer and apoptotic effects were reported for nitrostyrenes and nitrovinyl compounds in oral and colon cancers, together with the modulation of tumorigenesis in colon and breast cancers via reactive oxygen species (ROS) effects [41,42]. We also demonstrated the potent antiproliferative and pro-apoptotic effects of (*E*)-9-(2-nitrovinyl)-9,10-dihydro-9,10-ethanoanthracenes in BL cells [43], indicating that the nitrovinylanthracene pharmacophore may have potential for design of CLL targeting compounds. The tetracyclic ethanoanthracene scaffold is similar to the antidepressant maprotiline **16** (Figure 3), which demonstrated antiproliferative effects in B-cell malignancies [44]. Maleimide-based ethanoanthracenes have demonstrated selective cannabinoid receptor CB2 [45], antimicrobial [46] and anti-inflammatory activities [47], as well as the inhibition of S100P/RAGE interaction in pancreatic cancer cells [48]. Glucocorticoid receptor modulation [49] and neuronal calcium channel and glycogen synthase kinase-3 beta (GSK-3 beta) modulation targeting Alzheimer’s disease [50] have also been reported for ethanoanthracene derivatives.

In the present work, we report the design and synthesis of 110 novel compounds that are based on the 9,10-dihydro-9,10-ethanoanthracene and related scaffolds and that are grouped into seven series. Our objective was the identification of potent and selective compounds to target CLL with pro-apoptotic effects. The cycloaddition reaction of anthracene dienes with diverse dienophiles affords structurally varied ethanoanthracene products [43,45,46,47,48,49,50,51,52], while stereoselective Diels–Alder reactions of chiral C-9-substituted anthracenes with various maleimides have been reported [53]. Anthracenes suitably substituted with a nitrovinyl or α,β-unsaturated ketone group at C-9 were reacted with the dienophiles maleic anhydride, maleimide, *N*-phenylmaleimides, acrylonitrile and acetylenedicarboxylate to afford products containing a modified ethanoanthracene-bridgehead structure (Figure 3, target structures, Series 1–7). The ethanoanthracene compounds were biologically evaluated in the CLL cell lines HG-3 (containing unmutated immunoglobulin heavy-chain variable-region gene (IGHV), representing poor-prognosis CLL) and PGA-1 (containing mutated IGVH, representing good-prognosis disease).

## 2. Results and Discussion

### 2.1. Chemistry

#### 2.1.1. Series 1: (E)-9-(2-Nitrovinyl)-9,10-dihydro-9,10-[3,4]epipyrroloanthracene-12,14-diones (**20a–20h**)

**Scheme 1:**
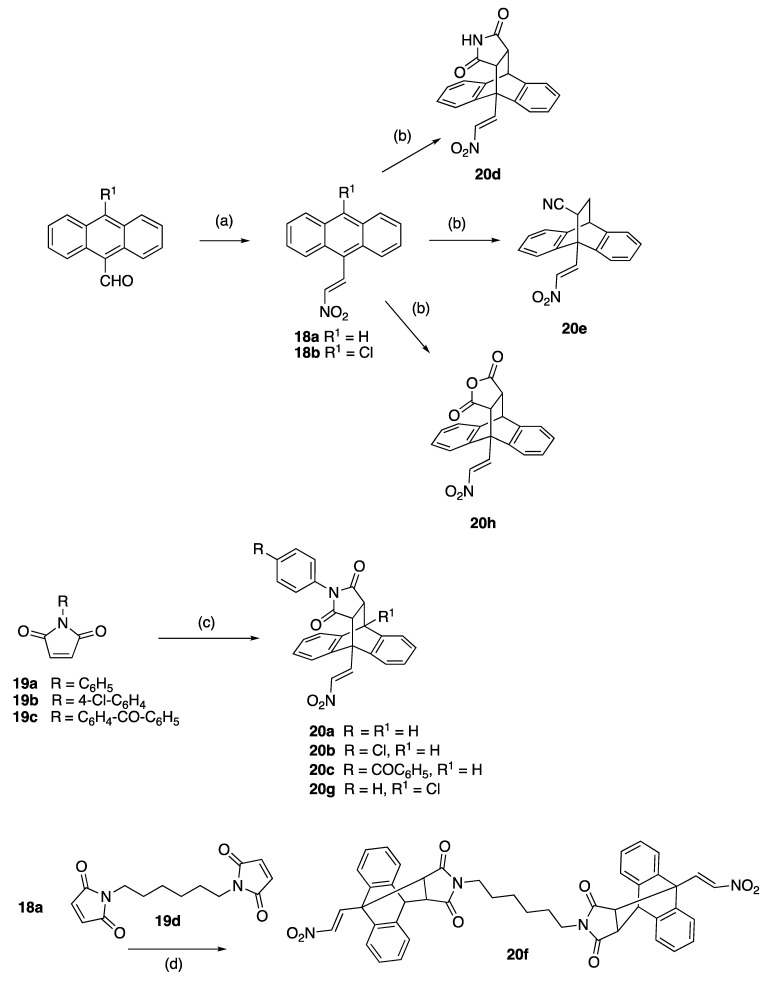
Synthesis of Series 1 ethanoanthracenes **20a–g** reagents and conditions: (**a**) piperidine acetate, excess nitromethane (CH_3_NO_2_), 90 °C, N_2_, 1.5 h (71–99%); (**b**) dienophile (maleic anhydride) for **20h**, maleimide for **20d**, NCHC=CH_2_ for **20e**, toluene, 90 °C, 48 h (30–80%); (**c**) dienophile **19a** for *N*-arylmaleimides **20a** and **20g**, **19b** for **20g**, **19c** for **20c**, toluene, 90 °C, 48 h, (15–51%); and (**d**) toluene, 90 °C, 48 h (10%).

Based on our previous research with (*E*)-9-(2-nitrovinyl)-9,10-dihydro-9,10-[3,4]epipyrroloanthracene-12,14-diones in BL [43], a focused series of the most potent compounds was identified for further evaluation and testing in related B-cell malignancy CLL, (Scheme 1). (*E*)-9-(2-Nitrovinyl)anthracenes **18a** and **18b** were prepared via a piperidine-catalyzed Henry–Knoevenagel condensation reaction of 9-anthraldehyde with nitromethane [43]. The dienophiles acrylonitrile, maleimide, maleic anhydride, *N*-phenylmaleimides **19a–c**, dimaleimide **19d** and dimethyl acetylenedicarboxylate were chosen for Diels–Alder cycloaddition. The maleimide dienophiles **19b, 19c** and **19d** were prepared from 2,5-furandione via amic acid intermediates [43]. The cycloaddition of dienophiles **19a–c** with (*E*)-9-(2-nitrovinyl)anthracene **18a** afforded the adducts **20a–20c**, whereas that with **18b** provided compound **20g** (30–51%), Scheme 1. Adducts **20d**, **20e** and **20h** were obtained upon the reaction of maleimide, acrylonitrile and maleic anhydride, respectively, with diene **18a**, while the dimer **20f** was obtained upon the cycloaddition of anthracene **18a** with the dimaleimide **19d,** as previously reported [43] (Scheme 1). These lead compounds **20a–20h** from BL studies bear a structural similarity to the tetracyclic antidepressant maprotiline **16**, containing the three main characteristics of an electrophilic functional group chain at position 9 of the anthracene-based core, the polyaromatic core itself and the two carbon-aliphatic bridge structure-linking positions 9 and 10 of the center ring.

#### 2.1.2. Series 2: (E)-3-(Anthracen-9-yl)-1-phenylprop-2-en-1-ones (**21a–q**)

The α,β-unsaturated carbonyl bioisosteric alternatives to the nitrovinyl group (located at C9 of the anthracene core) of our initial lead compounds (Series 1) were next investigated, principally due to potential nitrovinyl group genotoxicity and mutagenicity arising from biological metabolites of the nitro group [54]. Carcinogenicity, hepatotoxicity, mutagenicity and bone marrow suppression have been the major adverse toxicity issues associated with nitro-containing drugs [55]. The α,β-unsaturated ketone (chalcone) offers an alternative to the nitrovinyl group as an electrophilic system if required as a potential target for the enzymatic action of the compounds [56]. Covalent interactions of chalcones with biological targets can occur via the Michael-acceptor activity of the α,β-unsaturated carbonyl system, or they can also be mediated through radical-scavenging or reduction. [57] It is of interest that electrophilic compounds such as α,β-unsaturated ketones with CLL-selective cytotoxicity can interact with redox-sensitive proteins in primary CLL cells, resulting in the induction of nuclear factor erythroid 2–related factor 2 (Nrf2) signaling [58].

**Scheme 2:**
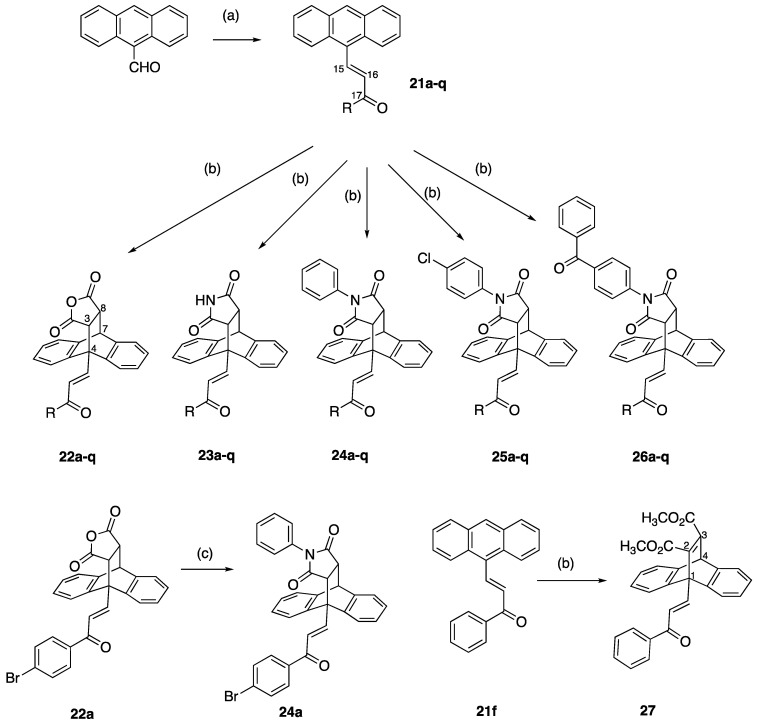
Synthesis of Series 2 anthracene chalcones **21a–q** and Series 3–7 ethanoanthracenes **22a–q**, **23a–q**, **24a–q**, **25a–q**, **26a–q** and **27** (see Table 1 for substituents and yields). Reagents and conditions: (**a**) Appropriate aryl methyl ketone, EtOH, NaOH, 20 °C, 24 h. (**b**) Appropriate anthracene chalcone **21a–q**, dieneophile (maleic anhydride for **22a–q**, maleimide for **23a–q**, *N*-phenylmaleimide for **24a–q**, *N*-(4-chlorophenyl)maleimide for **25a–q**, *N*-(4-benzoylphenyl)maleimide for **26a–q**, dimethyl acetylenedicarboxylate for **27**), toluene, 90 °C, 48 h. (**c**) Aniline, acetic acid 120 °C, 2–3 h (72%).

In the present work, a series of (*E*)-3-(anthracen-9-yl)-1-phenylprop-2-en-1-ones (Series 2) **21a–21q** was prepared using a Claisen–Schmidt condensation reaction between 9-anthraldehyde and a panel of substituted aromatic and heterocyclic acetophenone derivatives in the presence of sodium hydroxide (Scheme 2, Table 1, Step a, yields 38–88%). The chalcones contained the diverse substitution of the aryl ring in both positions and atom types, including halogens, alkyl, methoxy groups and an unsubstituted benzoyl ring. The 2-naphthyl, 2 and 4-pyridyl rings, together with 2-furyl and 2-thienyl heterocycles, were also investigated (Scheme 2, Table 1). In the ^1^H NMR spectrum of **21a**, H15 is identified as a doublet at δ 8.80 (*J* = 15.8 Hz), and it confirms the *E* alkene configuration [59]. The ^13^C NMR spectrum of compound **21a** identified the alkene C-15 and C-16 signals at 142.5 and 130.4 ppm, respectively (see Supplementary Information). The *E* configuration of alkene **21k** was confirmed via X-ray crystallography (Table 2) [60,61].

#### 2.1.3. Series 3: Maleic Anhydride-Substituted Ethanoanthracenes **22a–22q**

The preparation of compounds **22a–22q** was achieved using a Diels–Alder reaction, with the panel of substituted anthracene-chalcones **21a–21q** acting as a diene system and maleic anhydride acting as the dienophile [51] in yields of 30–99% (Scheme 2, step (b), Table 1). The products contained three distinct structural features: a dihydrofuran-2,5-dione bridgehead, an anthracene-derived central core and an α,β-unsaturated ketone with a substituted aryl ring. The IR spectrum of **22e** confirmed absorbances at 1669 cm^−1^ (α,β-unsaturated ketone) and 1774 cm^−1^ (maleic anhydride carbonyl). The ^1^H NMR spectrum of **22e** confirmed the anthracene core protons at δ 3.63 (dd, *J* = 9.5, 3.1 Hz), δ 3.81 (d, *J* = 9.5 Hz) and δ 4.85 (d, *J* = 3.3 Hz) assigned to H8, H3 and H7, respectively. The alkene H22 is identified at δ 7.87 (d, *J* = 16.2 Hz). In the ^13^C NMR spectrum of **22e**, C8, C3, C7 and quaternary C4 were identified at 54.1, 54.3, 49.4 and 56.33 ppm, respectively. The maleic anhydride C2, C9 and ketone C23 carbonyls were assigned at 175.4, 176.2 and 192.8 ppm. The alkenes C21 (136.8 ppm) and C22 (144.5 ppm) and ethanoanthracene bridges C3 (54.3, ppm), C7 (49.4 ppm) and C8 (54.1 ppm) were identified from the DEPT 90, HSQC and HMBC spectra (see Supplementary Information).

#### 2.1.4. Series 4: Maleimide-Substituted Ethanoanthracenes (**23a–23q**)

The ethanoanthracene compound panel **23a–23q** was prepared using the Diels–Alder reaction of the substituted chalcones **21a–21q** with maleimide (Scheme 2, Table 1). In the ^1^H NMR spectrum of **23a**, the signals at δ 3.32 (m), 3.82 (d, *J* = 8.7 Hz) and 4.81 (*J* = 3.3 Hz) were assigned to the aliphatic H8, H3 and H7, respectively. The maleimide NH was identified at δ 10.87. The ^13^C NMR spectrum of **23a** shows C8 of the ethanoanthracene bridge at 49.3 ppm, while the C3 was observed at 49.1 ppm. The signals at 45.0 and 51.7 ppm represent C7 and C4 of the anthracene core. The DEPT 90 spectrum was used to confirm the assignment of the alkene carbons C21 (143.1 ppm), C22 (131.5 ppm) and ethanoanthracene aliphatic bridge carbons C3 (49.1 ppm), C8 (49.3 ppm) and C7 (45 ppm) (Supplementary Information). The *E* configuration of the ethanoanthracene chalcone alkene of products **23f** and **23h** was confirmed via X-ray crystallography (Table 2) [61,62,63].

#### 2.1.5. Series 5: *N*-Phenylmaleimide-Substituted Ethanoanthracenes (**24a–24q**)

Compounds **24a–24q** were obtained using the Diels–Alder reaction of the anthracene chalcones **21a–21q** with *N*-phenylmaleimide (Table 1, Scheme 2). The *N*-phenylmaleimide adduct **24a** was alternatively prepared by reacting the corresponding maleic anhydride chalcone adduct **22f** with aniline in acid-catalyzed dehydration and the subsequent cyclization of the amic acid intermediate (Scheme 2, step c). This method resulted in a lower yield (72%) compared to the direct Diels–Alder cycloaddition of the chalcone **21f** and *N*-phenylmaleimide (84%). In the ^1^H NMR spectrum of **24i,** the characteristic aliphatic protons H8, H3 and H7 were identified at δ 3.55 (dd, *J* = 8.3, 3.3 Hz), 3.99 (d, *J* = 8.3 Hz) and 4.94 (d, *J* = 3.7 Hz), respectively, while the alkene protons H28 and H27 were observed at δ 7.81 and 7.89 (*J* = 16.3 Hz), confirming the *E* configuration. In the ^13^C NMR spectrum of **24i,** the signals at 45.4, 48.2 and 48.3 ppm were assigned to C7, C3 and C8, respectively, of the aliphatic ethanoanthracene bridge using the HMBC and HSQC spectra, while C4 was identified at 52.04 ppm.

The stereo- and regioselectivity of Diels–Alder reaction and retention of the alkene *E* configuration was obtained from the X-ray crystallography of **24a** and **24g** (Table 2) [61,62,63]. Interestingly, the reaction of *N*-cyclohexylmaleimide and 9-hydroxymethylanthracene in the confined space inside carbon nanotubes resulted in the 1,4-exo Diels–Alder cycloaddition product instead of the 9,10-endo adduct obtained in conventional conditions [64].

#### 2.1.6. Series 6: *N*-(4-Chlorophenyl)maleimide-Substituted Ethanoanthracenes (**25a–25q**)

The ethanoanthracenes **25a–25q** were prepared using the Diels–Alder reaction as above, with the substituted chalcones **21a–21q** acting as the diene and *N*-(4-chlorophenyl)maleimide **19b** as the dienophile (yields 3–95%) (Scheme 2, Table 1). The ^1^H NMR spectrum of **25k** shows H-8, H-3 and H-7 at δ 3.53 (dd, *J* = 8.3, 3.3 Hz), δ 4.00 (d, *J* = 8.3 Hz) and δ 4.94 (*J* = 3.3 Hz), while the alkene H-26 and H-27 were identified as doublets at δ 7.80 and δ 7.88 (*J* = 16.2 Hz), respectively. In the ^13^C NMR spectrum of **25k**, C-8, C-3, C-7 and C-4 of the ethanoanthracene bridge were identified at 45.4, 48.3, 48.6 and 52.11 ppm, respectively. (See Supplementary Information for HSQC, DEPT 90 and HMBC spectra.) The X-ray structure of **25f** is displayed in Table 2.

#### 2.1.7. Series 7: *N*-(4-Benzoylphenyl)maleimide-Substituted Ethanoanthracenes (**26a–26q**) and Adduct 27

The *N*-(4-benzoylphenyl)maleimide adducts **26a–26q** were obtained using the Diels–Alder cycloaddition reaction of the anthracene chalcones **21a–21q** with the *N*-(4-benzoylphenyl)maleimide **19c** (Scheme 2, Step b, Table 1). In the ^1^H NMR spectrum of **26l**, the ethanoanthracene carbon bridge protons H8, H3 and H7 were identified at δ 3.59 (*J* = 3.3, 8.3 Hz), 4.04 (d, *J* = 8.3 Hz) and δ 4.97 (d, *J* = 2.9 Hz). In the ^13^C NMR spectrum of **26l,** the ethanoanthracene bridge carbons C3, C8 and C7 and alkenes C27 and C28 were identified at 48.5, 45.4, 52.2, 142.8 and 131.8 ppm, respectively (see Supplementary Information).

The Diels–Alder reaction of the chalcone **21f** with dimethyl acetylenedicarboxylate afforded the adduct **27**, containing an alkene on the central carbon bridge (Scheme 2, Step b) [65]. H-4 (center ring of the polycyclic core) was identified at δ 5.80 ppm, while H-10 and H-13 of the polycyclic core, together with alkene H-18, were identified at δ 7.55 as a multiplet; the doublet δ 7.73, *J* = 16.1 Hz, corresponded to alkene H-17. In the ^13^C NMR spectrum, the signals at 49.9, 52.9, 53.2 and 57.8 ppm were assigned to C1, C4, C2 and C3 (bridgehead alkene). The structure of the adduct **27** was confirmed via X-ray crystallography (Table 2) [61,62].

### 2.2. X-ray Crystallography Study of Ethanoanthracenes

The X-ray-crystallographic analysis of a series of the novel ethanoanthracene adducts was carried out, and it confirmed the regioisomer obtained from the Diels–Alder reactions (Table 2). Initially, the *E* configuration of the alkene **21k** was observed via X-ray crystallography (Table 2). The C15–C16 bond length was consistent with an alkene (1.336 Å), and the C17–O18 bond length was consistent with a carbonyl group (1.228 Å) [60,61]. The trigonal–planar nature of the alkene bond was also evident in bond angles of 120.1° and 124.6° for C15-C16-C17 and C1-C15-C16, respectively (Supplementary Information, Table S1 and Figure S3 for crystal data and structure refinement for **21k**).

The retention of the *E* configuration for the alkene double bond of the maleimide-substituted ethanoanthracenes products **23f** and **23h** was confirmed via X-ray crystallography with a C6–C7 alkene bond length 1.321 Å and a O3–C8 carbonyl bond length of 1.219 Å [61,62] (Table 2). Bond angles of 128.06° and 120.82° for C5–C6–C7 and C6–C7–C8, respectively, indicated the trigonal–planar configuration of the alkene. The core dihydroethanoanthracene moiety was rigid, with the flat succinimide ring fused to the ethano-bridge; the presence of the imide was supported by the carbonyl bond lengths of 1.205 Å for O1–C1 and O2–C2. In addition, the N1–C1 and N1–C2 bond lengths of 1.375 Å and 1.380 Å, respectively, confirm the C-N imide bond [63]. The X-ray crystal showed the presence of a racemic mixture, with the two stereocenters of the ethanoanthracene carbon bridge in the (*R*,*R*) and (*S*,*S*) stereochemical configuration (Supplementary Information, Table S1, and Figures S4, S7 and S8).

Additional evidence to confirm the stereo- and regioselectivity of the Diels–Alder reaction and retention of the alkene *E* configuration was obtained from the X-ray crystallography of the *N*-phenylmaleimide-substituted ethanoanthracenes **24a** and **24g** (Table 2). For compound **24a**, the C10–C11 (1.335 Å) and O9–C8 (1.227 Å) bond lengths were consistent with alkene and carbonyl functional groups. Bond angles of 123.9° and 125.6° for C10–C11–C12 and C8–C10–C11, respectively supported the trigonal–planar configuration [61,62]. Bond lengths of 1.18 Å and 1.19 Å for O32–C31 and O29–C28 are typical of the maleimide carbonyl groups. Furthermore, the N30–C33 bond (1.36 Å) was within the expected range for conjugated C–N bonds [63]. (See Figure S5 and Table S1, Supplementary Information, for crystal data and structure refinement for **24a** and **24g.**)

The X-ray structure of the *N*-(4-chlorophenyl)maleimide-substituted ethanoanthracene **25f** is displayed in Table 2, which confirms the structural assignment, showing the core dihydroethanoanthracene moiety to be rigid, with similar conformations observed in the various X-ray structures discussed above. (See Table 2 and Table S1, Supplementary Information, for crystal data and structure refinement for compound **25f**.)

The structure of the adduct **27** obtained from the chalcone diene **21f** with dienophile dimethyl acetylenedicarboxylate was also confirmed via X-ray crystallography (Table 2). The bond length of C15–C16 was consistent with an alkene (1.3269 Å), and the bond length of 1.2263 Å was consistent with a carbonyl (O28–C27) [61,62]. The bond angles for C14–C25–C26 and C27–C26–C25 were observed as 126.47° and 120.01°, respectively, indicating the trigonal–planar structure. The ester carbonyl bond lengths of 1.2073 Å and 1.2021 Å were observed for O18–C17 and O22–C21, while the O23–C24 and O19–C20 bond lengths of 1.45 Å and 1.4464 Å are typical of the ester C–O bond. The distinctive ethenoanthracene alkene bridgehead was observed with a bond length of 1.34 Å (see Figure S7 and Table S1, Supplementary Information, for crystal data and structure refinement for compound **27**).

### 2.3. Stability Study for Chalcones ***21a***, ***21i***, Anhydride ***22h***, and Maleimides ***23a***, ***23g***, ***23n***, ***24a***, ***24h***, ***26a*** and ***26n***

**Figure 4:**
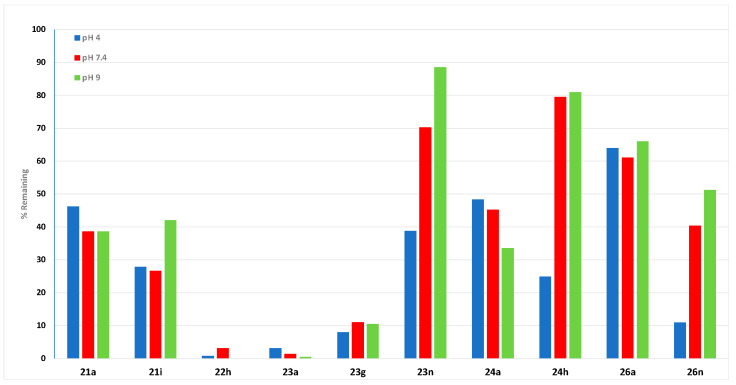
Stability study for compounds **21a**, **21i**, **22h**, **23a**, **23g**, **23n**, **24a**, **24h**, **26a** and **26n** at pH 4.0, pH 7.5 and pH 9.0 over 24 h.

A preliminary stability study was carried out on selected chalcones, maleic anhydride and maleimide adducts at acidic, neutral and basic conditions (pH 4, 7.4 and 9) using HPLC to determine suitability for further preclinical investigations. Chalcones **21a** and **21i**, anhydride **22h**, and maleimides **23a**, **23g** and **24a** demonstrated poor stability over the pH range studied, with <50% recovery at 24 h (Figure 4 and Supplementary Information Table S2, Figure S1). At pH 4.0, the rate of degradation of 2-pyridyl-substituted ethanoanthracene **23n** was minimal up to 8 h, with a half-life (t_1/2_) of 19 h, while at pH 7.4, degradation was slower; t_1/2_ >24 h. The compound remained stable at pH 9.0, with an 11% decrease in compound concentration over 24 h and t_1/2_> 24 h. Compound **23n** was most stable at pH 9.0 (89% drug remaining at 24 h) in comparison with 70% and 39% remaining at pH 7.4 and 4.0 after 24 h. A possible degradation route for the maleimide **23n** could be through the hydrolysis of the cyclic imide of the maleimide ring, leading to the resultant amic acid product [66,67]. Compound **23n** was the most stable of the compounds evaluated at pH 9, and it would be suitable for further preclinical investigation. The *N*-phenylmaleimide adduct **24h** demonstrated notable stability at pH 7.4 and pH 9, with 80% and 81%, respectively, remaining at 24 h, and t_1/2_ >24 h. At pH 4, it was less stable with 25% remaining (t_1/2_ = 14.8 h). The *N*-benzoylmaleimide **26a** demonstrated good stability with 64%, 61% and 66% remaining after 24 h in pH conditions 4, 7.4 and 9, respectively, and t_1/2_> 24 h, while **26n** was less stable with 12% remaining at pH 4 (24 h).

All samples were analyzed using acetonitrile–water (80:20%, 70:30%, 60:40% isocratic) as the mobile phase over 10 min and a flow rate of 1 mL/min. Stock solutions were prepared by dissolving 5 mg of compounds in a mobile phase ([(acetonitrile (80%): water (20%)], 10 mL). Anhydride **22h** was not detected at its retention time of 5.2 min at pH 9.0. The data presented represent the results of single experiments.

### 2.4. Biological Results and Discussion

#### 2.4.1. Evaluation of In Vitro Antiproliferative Activity of Chalcone and Nitrovinyl-Substituted Ethanoanthracenes in CLL Cell Lines HG-3 and PGA-1

The phenotypic cellular responses of the synthesized ethanoanthracenes were then investigated in the CLL cell lines HG-3 and PGA-1. The HG-3 cell line is representative of a poor patient prognosis; the cell line was established via EBV (Epstein–Barr virus) infection from an IGVH1–2 unmutated B1 lymphocyte-origin CLL patient clone [68]. The PGA-1 cell line, which is representative of a good patient prognosis, was established from leukemic B cells of a CLL patient with mutated IGVH1-2 [69]. The compounds were evaluated at 10-µM and 1-µM concentrations using an alamarBlue viability assay, and fludarabine was used as a clinically relevant comparative positive control [39]. Vehicle-treated cells [(DMSO (1% *v*/*v*)] were considered 100% viable, and the viabilities of each compound were calculated accordingly. The structure–activity relationships for the series were determined, and the most potent ethanoanthracene compounds were identified for further investigation. The present study was arranged according to structural type (Series 1–7).

#### 2.4.2. Series 1: Nitrovinyl-Substituted Ethanoanthracenes (**20a–20h**)

**Figure 5:**
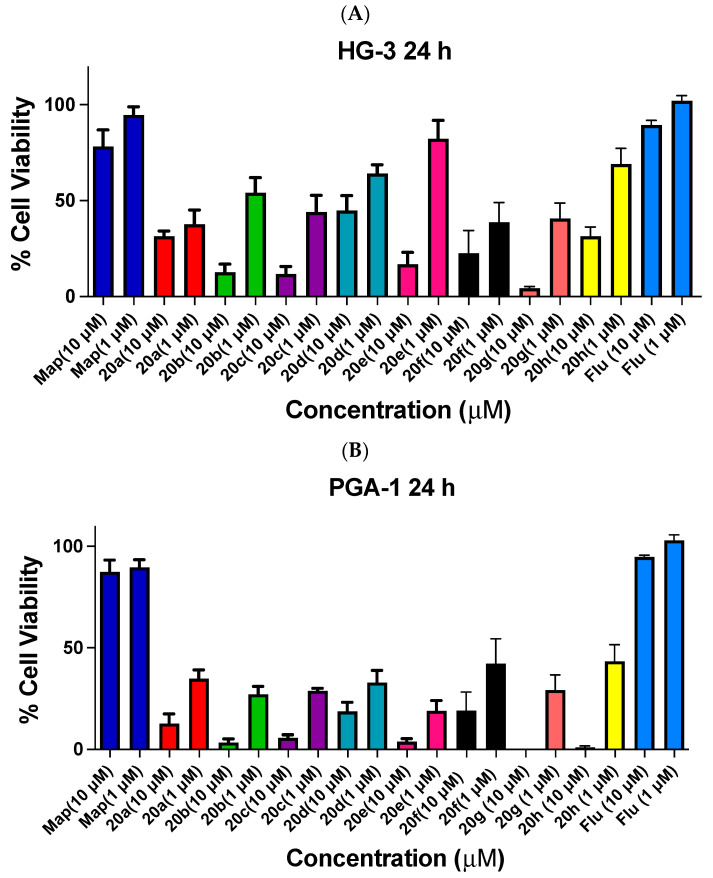
Cell viability data for ethanoanthracenes **20a–e**, **20g**, **20h** and **20f**. Cell viability data for (*E*)-9-(2-Nitrovinyl)-9,10,11,15-tetrahydro-9,10-[3,4]epipyrroloanthracene-12,14-diones **20a–e, 20g** and **20h** and the related dimer **20f** in CLL: (**A**) HG-3 cells (1 and 10 µM) and (**B**) PGA-1 cells (1 and 10 µM). The cell proliferation of HG-3 and PGA-1 cells was determined with an alamarBlue assay. Compound concentrations of either 1 µM or 10 µM for 24 h were used to treat the cells (in triplicate) with control wells containing vehicle DMSO (1% *v*/*v*). Map = maprotiline; Flu = fludarabine. The mean value for three experiments is shown.

The antiproliferative activities of the nitrovinyl-substituted ethanoanthracenes **20a–20h** in HG-3 and PGA-1 cell lines are shown in Figure 5, with reference compounds maprotiline and fludarabine. These were among the most potent compounds initially identified in our previous work in the related B-cell malignancy BL cell lines (e.g., compound **20a** IC_50_ = 0.21 µM in a chemosensitive MUTU-1 cell line and IC_50_ = 0.71 µM in a chemoresistant DG-75 cell line [43]). In HG-3 cells, at 10 µM treatment concentration, the most potent compounds were identified as the *N*-phenylmaleimide adducts **20g** (2% cell viability), **20b** (8%), and **20c** (11%); the dimer **20f**, *N*-phenylmaleimide **20a**, maleimide **20d** and acrylonitrile adduct **20e** show reduced cell viability (14–43%). In PGA-1 cells, the most effective compounds were identified as **20g** (1% viable cells), **20h** (2%), **20b** (3%), **20c** (4%) and **20e** (3%) at 10 µM concentration, while compounds **20a**, **20d** and **20f** were less potent (11%, 17% and 17%, respectively). The compounds were more effective in PGA-1 compared to HG-3 cells with the *p*-chloro- and benzophenone-based *N*-phenylmaleimides **20b** and **20c,** as well as the acrylonitrile adduct **20e**, initially identified as promising antiproliferative agents.

#### 2.4.3. Series 2: (E)-3-(Anthracen-9-yl)-1-phenylprop-2-en-1-ones (21a-21q)

**Figure 6:**
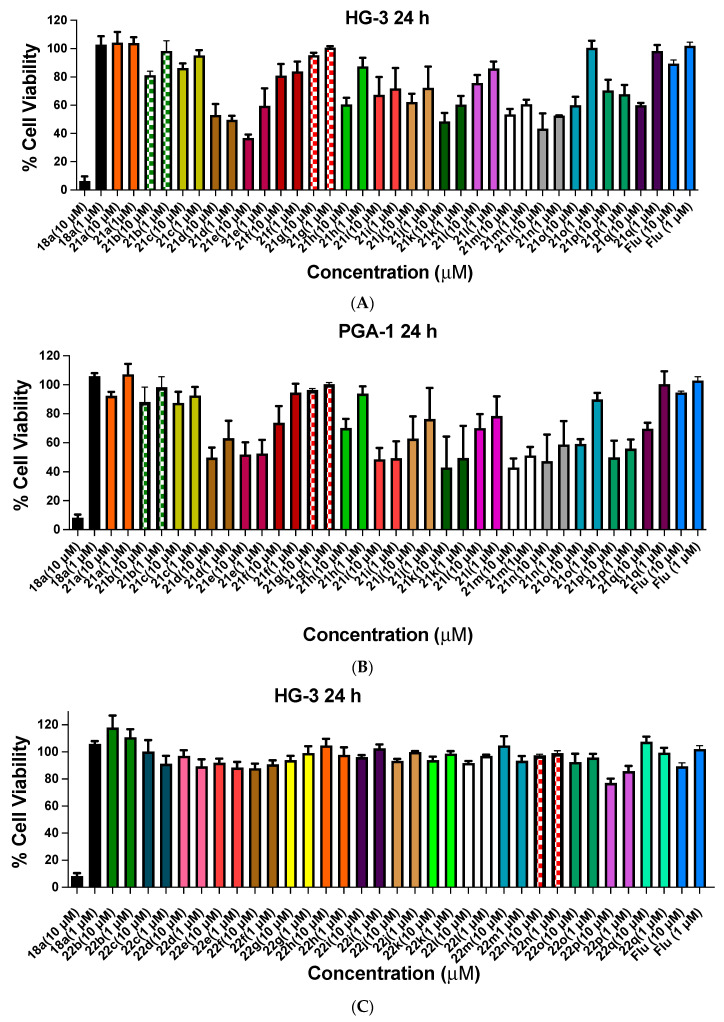

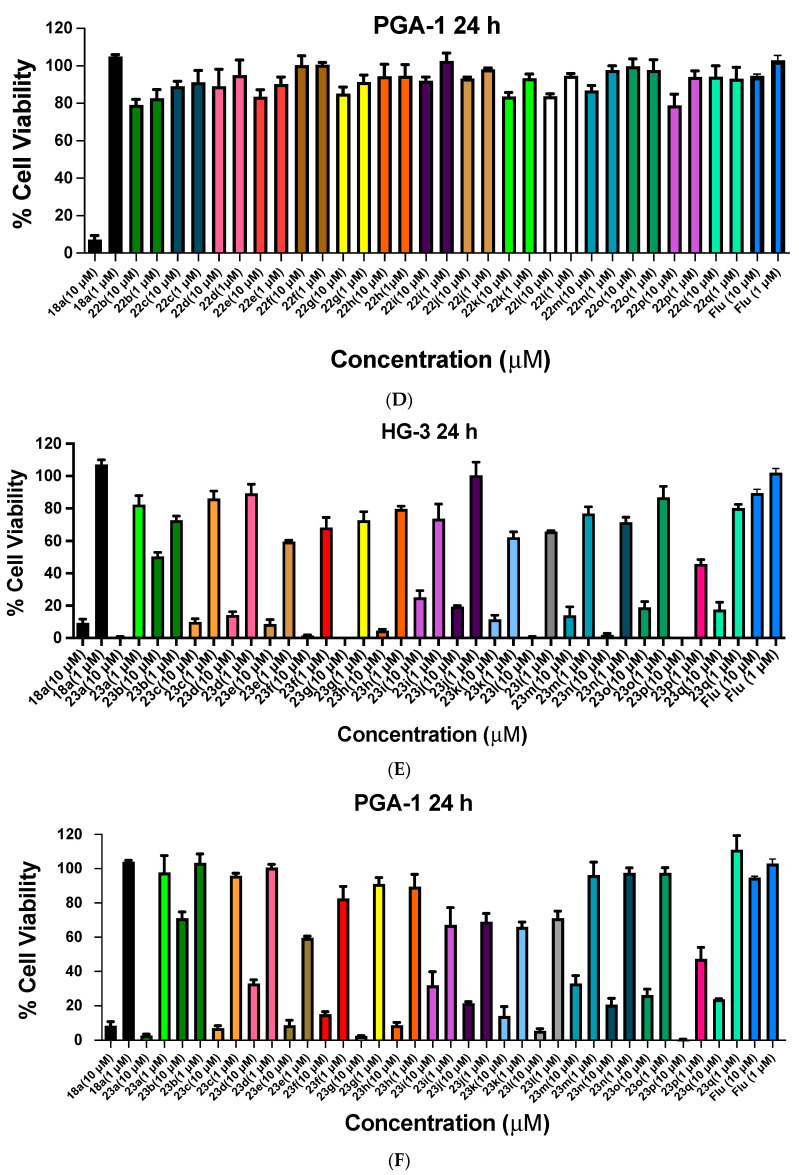
Cell viability data for chalcones **21a–q** and ethanoanthracenes **22a–22q** and **23a–23q** in CLL cell lines HG-3 and PGA-1. Cell viability data for chalcones **21a–q** (**A**,**B**), maleic anhydride ethanoanthracene adducts **22a–22q** (**C**,**D**) and maleimide ethanoanthracene adducts **23a–23q** (**E**,**F**) in CLL cell lines: the cell proliferation of HG-3 and PGA-1 cells was determined with an alamarBlue assay. Compound concentrations of either 1 µM or 10 µM for 24 h were used to treat the cells (in triplicate) with control wells containing vehicle DMSO (1% *v*/*v*). Flu = fludarabine. The mean value for three independent experiments is shown.

The biochemical activity of the anthracene-based chalcones **21a–21q** in the HG-3 and PGA-1 cell lines are shown in Figure 6A,B, with fludarabine and nitrovinylanthracene **18a** acting as internal standards. These structures contain diverse functional group substitutions on the α,β-unsaturated ketone, ranging from ring-activating groups (4-nitro aryl, **21b**) to ring-deactivating groups (3,4-dimethoxy aryl, **21e**) and polyaromatic systems (2-naphthyl, **21p**). From the initial HG-3 cell line screen, the most potent compounds observed were **21e** (3,4-dimethoxy), **21n** (2-pyridyl), **21k** (3,4,5-trimethoxy), **21d** (2,4-dichloro) and **21m** (4-pyridyl) with % viability in the range of 37–54% at a 10 µM concentration and a 50–61% viability range at 1 µM (Figure 6A). In the PGA-1 cell line, the most potent compounds at a 10-µM treatment concentration were **21m** (4-pyridyl, 43% cell viability), **21k** (3,4,5-trimethoxy, 43%), **21n** (2-pyridyl, 47%), **21i** (4-methoxy, 49%), **21d** (2,4-dichloro, 50%) and **21p** (2-naphthyl, 50%). The halogenated compounds were weakly active at 10 µM with **21h** (4-fluoro) and **21l** (4-chloro) (70% viability) (Figure 6B). The anthracene chalcones from Series 2 were not evaluated further due to their poor activity, although the antiproliferative activity of chalcones was previously reported in various leukemia cell lines [70].

#### 2.4.4. Series 3: Maleic Anhydride-Substituted Ethanoanthracenes (**22a–22q**)

The cell viability of compounds **22a–22q** in the HG-3 and PGA-1 cell lines is shown in Figure 6C,D. The majority of this compound series was weakly active or inactive across both treatment concentrations with the exception of **22p** (2-naphthyl) with cell viability of 77% in HG-3 and 79% in PGA-1 cells. The poor activity displayed for these compounds in series 3 did not merit further investigation.

#### 2.4.5. Series 4: Maleimide-Substituted Ethanoanthracenes (**23a–23q**)

The antiproliferative activities of the maleimide-substituted ethanoanthracenes compounds **23a–23q** in the HG-3 cell line are shown in Figure 6E. In contrast to the maleic anhydride Series 3 compounds, the maleimide adducts demonstrated promising activity with the most potent of the panel identified as **23p** (2-naphthyl, 0% viable cells), **23g** (4-iodo 0%), **23a** (4-bromo, 0.5%), **23l** (4-chloro, 0.7%), **23f** (phenyl, 2%) and **23n** (2-pyridyl, 2%) when screened at a 10-µM concentration. Similar trends were observed at the 1-µM concentration, with the most potent compound identified as **23p** (2-naphthyl derivative, 46%). The most potent of the panel screened in the PGA-1 cell line at 10 µM were identified as **23p** (2-naphthyl, 0% viable cells), **23g** (4-iodo, 2%), **23a** (4-bromo, 3%), **23l** (4-chloro, 5%), **23c** (4-ethyl, 7%) and **23h** (4-fluoro, 9%); see Figure 6F. With an increasing size of the halogen substituent and increasing electronegativity on the *para* position of the acetophenone-derived aryl ring, an increase in compound potency was observed (I > Br > Cl > F), as also noted for the HG-3 cells. Compared to maleic anhydride (Series 3), the maleimide-derived adducts elicited greater biological activity at both concentrations and in both cell lines tested. These diverse sets of aryl substituents (alkyl, halogens and nitro) for heterocycles eliciting a similar degree of antiproliferative activity in the CLL cells suggests a moderate tolerance for substitution on the acetophenone-derived portion of the molecules in relation to their anticancer effect. The Series 4 compounds are generally more effective in the aggressive HG-3 cell type than in PGA-1 cells; e.g., compounds **23n** (2-pyridyl) and **23f** (phenyl) were notably less effective at 10 µM in PGA-1 cells (19% higher viability for **23n** and 13% higher viability for **23f** compared to HG-3 cells).

#### 2.4.6. Series 5: *N*-Phenylmaleimide Substituted Ethanoanthracenes (**24a–24q**)

**Figure 7:**
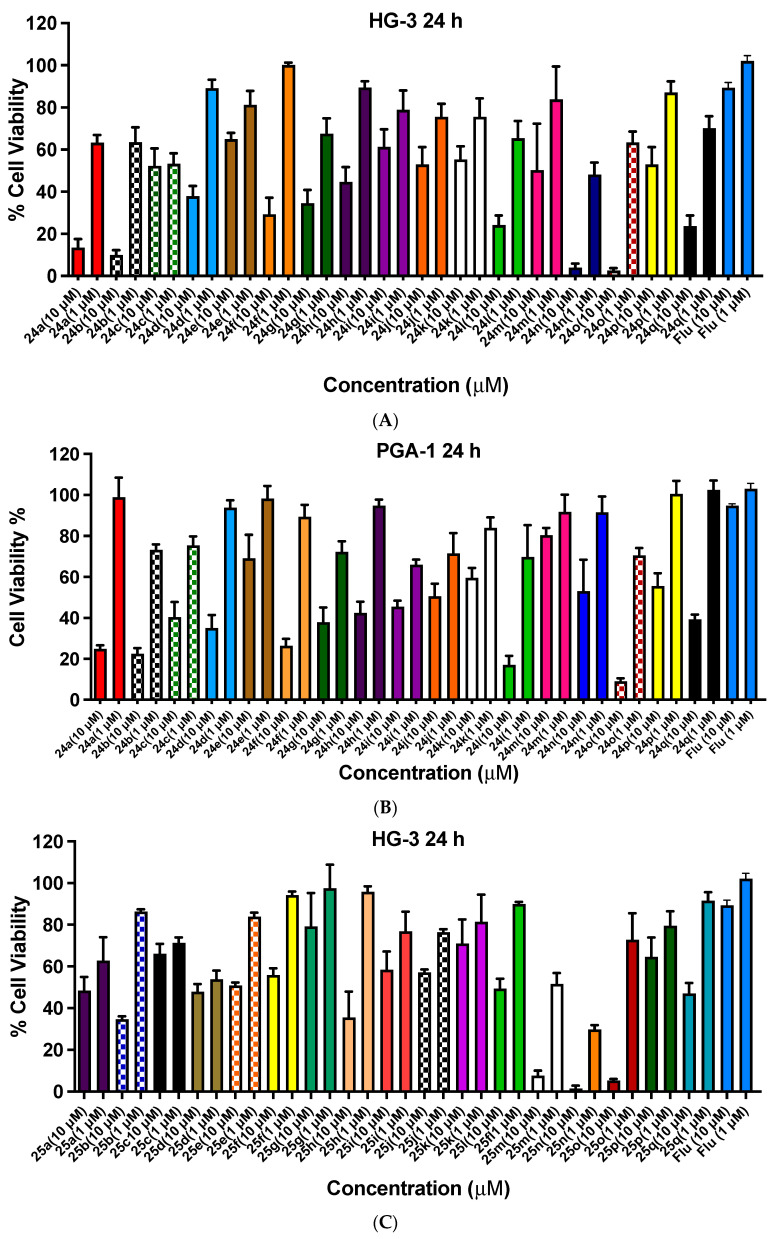

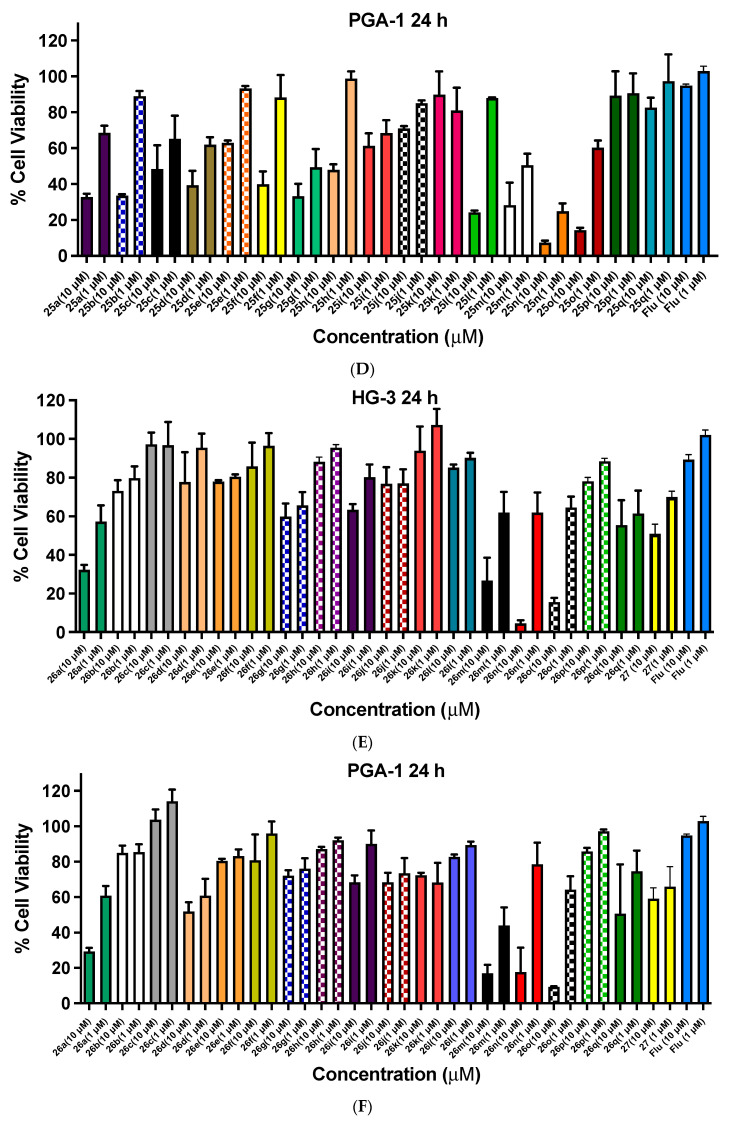
Cell viability data for ethanoanthracenes **24a–q**, **25a–q**, **26a–q** and **27** in CLL cell lines HG-3 and PGA-1. Cell viability data for *N*-phenylmaleimide-substituted ethanoanthracenes (**24a–q**, Panels **A**,**B**), *N*-(4-chlorophenyl)maleimide-substituted ethanoanthracenes (**25a–q,** Panels **C** and **D**) and *N-*(4-benzoylphenyl)maleimide-substituted ethanoanthracenes (**26a–q**, Panels **E**,**F**) and **27** (**E**,**F**) were determined in CLL cells HG-3 cells (1 and 10 µM) and PGA-1 cells (1 and 10 µM). The cell proliferation of HG-3 and PGA-1 cells was determined with an alamarBlue assay. Compound concentrations of either 1 µM or 10 µM for 24 h were used to treat the cells (in triplicate) with control wells containing vehicle DMSO (1% *v*/*v*). Flu = fludarabine. The mean value for three independent experiments is shown.

The cell viability results for the *N*-phenylmaleimide compounds **24a–24q** in the HG-3 cell line are shown in Figure 7A. All compounds were effective at a 10-µM concentration, with the most potent compounds identified in the screen as follows: **24o** (2-furyl, 3% cell viability), **24n** (2-pyridyl, 4%), **24b** (4-nitro, 10%), **24a** (4-bromo, 14%,), **24l** (4-chloro, 24%,) and **24q** (2-thienyl, 24%). The remaining compounds were also moderately effective, with cell viability in the range of 29–65%. At the lower treatment concentration (1 µM), **24n** (2-pyridyl) showed the greatest activity (48% viable cells remaining). In the PGA-1 cell line (Figure 7B), a similar trend was observed with the most potent compounds identified as **24o** (2-furyl, 9% viable cells), **24l** (4-chloro, 17%), **24b** (4-nitro, 22%), **24a** (4-bromo, 25%) and **24f** (phenyl, 26%).

#### 2.4.7. Series 6: *N*-(4-Chlorophenyl)maleimide-Substituted Ethanoanthracenes (**25a–25q**)

The antiproliferative activity of the *N*-(4-chlorophenyl)maleimide series **25a–25q** in the HG-3 cell line is shown in Figure 7C. The most potent compounds of the series screened were identified as **25n** (2-pyridyl, 2% viable cells), **25o** (2-furyl, 5%,), **25m** (4-pyridyl, 8%), **25h** (4-fluoro, 35%,), **25b** (4-nitro, 35%) and **25q** (2-thienyl, 47%). **25n** (2-pyridyl) was also identified as the most potent at 1 µM with 30% cell viability. The *N*-(4-chlorophenyl)maleimides identified with promising activity from the panel screened in the PGA-1 cell at a 10-µM concentration were **25n** (2-pyridyl, 7% viable cells), **25o** (2-furyl, 14%), **25l** (24%, 4-chloro), **25m** (4-pyridyl, 28%), **25a** (4-bromo, 33%), **25g** (4-iodo, 33%) and **25b** (4-nitro, 34%). At the lower treatment concentration (1 µM), **25n** was also observed to have the greatest activity (25% cell viability), (Figure 7D).

#### 2.4.8. Series 7: *N*-(4-Benzoylphenyl)maleimide-Substituted Ethanoanthracenes (**26a–26q**)

The preliminary biochemical evaluation of the *N*-(4-benzoylphenyl)maleimide compound series **26a–26q** in the HG-3 cell line is shown in Figure 7E, with the most potent compounds of the panel screened at 10 µM identified as **26n** (2-pyridyl, 4% viable cells), **26o** (2-furyl, 15%), **26m** (4-pyridyl, 27%), **26q** (2-thienyl, 56%) and **26a** (4-bromo, 31%). At a 1-µM treatment concentration, **26a** (4-bromo) was the most effective (55% cell viability). In the PGA-1 cell line (Figure 7F), **26o** (2-furyl, 9% viable cells remaining), **26m** (4-pyridyl, 17%), **26n** (2-pyridyl, 18%) and **26a** (4-bromo, 29%) were also identified as potent lead compounds. At a lower treatment concentration of 1 µM, **26m** (4-pyridyl) demonstrated the greatest activity with 43% cell viability.

Following this preliminary evaluation of the panel of ethanoanthracene compounds described above, a number of key structural features were identified as critical for antiproliferative activity in CLL cells. The nitrovinyl and 1-phenyl-2-propen-1-one pharmacophores were demonstrated to be critical for cytotoxic effect. The maleimide-derived adducts (Series 4–7) were also found to produce a more potent effect than the corresponding furan-based products (Series 3). However, the nitrovinyl-based maleic anhydride adduct **20h** (Series 1) demonstrated significantly greater efficacy in both cell lines (31% viability in HG-3; 2% in PGA-1 at 10 µM) when compared with the most potent maleic anhydride chalcone adduct **22p** (>77% viability in both cell lines at 10 µM), thus confirming the requirement for the nitrovinyl pharmacophore for potency in the maleic anhydride-based series. For the maleimide-based compounds (Series 4–7), a comparison of the potent 2-pyridylchalcones compounds **23n** (Series 4), **24n** (Series 5), **25n** (Series 6) and **26n** (Series 7) confirmed the *N*-(4-benzoylphenyl)maleimide substitution as the most effective of the structural series evaluated.

In a more detailed analysis of the structure–activity relationships of the *N*-(4-benzoylphenyl)maleimide compounds (Series 7), it was observed that the replacement of the unsubstituted benzoyl aryl ring of **26f** with the heterocycles in **26m** (4-pyridyl)**, 26n** (2-pyridyl) and **26q** (2-thienyl) on the molecular scaffold resulted in an increase in potency for the compounds across both cell lines. The 4-pyridyl derivative **26m** showed a notable decrease in cell viability of 64% (to 17% viable cells) at a 1-µM treatment concentration in comparison to the unsubstituted **26f** in the PGA-1 cell line. In HG-3 cells, a 59% reduction in cell viability was observed at 10 µM for **26m** and a 64% decrease at the 1-µM concentration compared to **26f**. With **26n** (2-pyridyl derivative), a significant reduction in cell viability was noted in PGA-1 cells, showing decreases of 63% and 20% at 10 µM and 1 µM, respectively. Similarly, in HG-3 cells, **26n** showed substantial decreases in cell viability compared to **26f** (82% greater decrease in cell viability at 10 µM and 34% greater decrease at 1 µM). In addition, the presence of bromine at C-4 of the benzoyl aryl ring of **26a** resulted in a significant increase in biological activity when compared to **26f,** leading to 20% and 70% reduction in cell viability at 10 µM and 1 µM in PGA-1 cells. In HG-3 cells, a similar decrease in cell viability of 29% and 64% at 10- and 1-µM treatment concentrations, respectively, was observed. The acetylene adduct **27** showed moderate activity (50% viability in HG-3 and 58% in PGA-1 cells) at 10 µM. This result indicated that the maleimide structure present in the maleimide series 4 adducts resulted in superior efficacy for corresponding compound **23f** (0.5%, viability in HG-3, 13.9% viability in PGA-1 cells) when compared with the alkene-type bridge structure in compound **27**.

The more potent compounds identified from Series 1 and Series 4–7 were then progressed to further biological investigations.

#### 2.4.9. In vitro IC_50_ Determination of Selected Nitrovinyl-Based Ethanoanthracene and Chalcone-Based Ethanoanthracene Derivatives in CLL Cells

The IC_50_ values of the most promising anti-proliferative nitrovinyl-based ethanoanthracene and chalcone-based ethanoanthracene compounds identified from preliminary biological screening at 10-µM and 1-µM concentrations were obtained for both HG-3 and PGA-1 cell lines. Fludarabine was used as a clinically relevant positive control; IC_50_ = 28.1 µM (HG-3) and 32.0 µM (PGA-1).

#### 2.4.10. (E)-9-(2-Nitrovinyl)-Substituted Ethanoanthracenes (Series 1)

Compounds **20a–20f** were selected and tested across a concentration range of 5–0.01 µM and assessed for viability at 24 h using the alamarBlue assay. The compounds exerted a more potent effect than the fludarabine control (18–59-fold greater in HG-3 cells, 29–53-fold greater in PGA-1 cells) across both HG-3 and PGA-1 cell lines with IC_50_ ranges of 0.48–1.6 µM and 0.61–1.1 µM, respectively. In HG-3 cells, the most potent compounds identified were **20a** (IC_50_ 0.48 µM, *N*-phenylmaleimide derivative) and **20c** (IC_50_ 0.71 µM, *N*-(4-benzoylphenyl)maleimide; while in PGA-1 cells, the most potent compounds were **20a** (IC_50_ 0.61 µM) and **20b** (IC_50_ 0.66 µM, *N*-(4-chlorophenylmaleimide). Overall, the two most potent compounds across both cell lines were **20a** (mean IC_50_: 0.55 µM) and **20b** (mean IC_50_: 0.76 µM). In both cell lines, **20e** (cyano-derivative) had the highest IC_50_ values (Table 3) with the remaining compounds having sub-micromolar activity. This suggests that the general maleimide structure plays an important role in the biological activity observed, as we previously reported in BL [71]. In addition, the presence of an unsubstituted aromatic ring on the maleimide-derived functional group **20a** leads to better activity over the unsubstituted maleimide **20d**, *N*-(4-chloromaleimide **20b** and the *N*-(4-benzoylphenyl)maleimide **20c** adducts. The novel dimeric compound **20f,** which we identified with potent anti-proliferative activity in BL [43] [71], was also demonstrated to exert a more potent effect than the fludarabine control (165-fold greater in HG-3 cells, 91-fold greater in PGA-1 cells) across both HG-3 and PGA-1 cell lines with IC_50_ values of 0.17 µM and 0.35 µM, respectively, and a mean IC_50_ value of 0.53 µM.

#### 2.4.11. In Vitro IC_50_ Determination of the Most Potent Chalcone-Based Ethanoanthracene Derivatives in HG-3 and PGA-1 Cells (Series 4–7)

The most potent compounds from the preliminary screening of Series **2–7** (compounds **23a**, **23c**, **23f–23i**, **23k, 23l, 23n, 23p**, **24f**, **24l**, **25n** and **26n**) were chosen for IC_50_ determination in the PGA-1 and HG-3 cell lines. The compounds were tested over a concentration range of 50–0.01 µM and assessed at 24 h via an alamarBlue cell viability assay. The results are presented in Table 4. All compounds exerted a more potent effect than the fludarabine control (3–22-fold greater in HG-3 cells, 3–37-fold greater in PGA-1 cells) across both HG-3 and PGA-1 cell lines with IC_50_ ranges of 1.31–10.28 µM and 0.31–12.6 µM, respectively. In HG-3 cells, the most potent compounds identified were **25n** (IC_50_ 1.31 µM, 2-pyridyl, *N-*(4-chlorophenyl)maleimide derivative) and **23p** (IC_50_ 1.41 µM, 2-naphthyl, maleimide derivative), while in PGA-1 cells, the most potent compounds were **24l** (IC_50_, 0.31 µM, 4-chloro, maleimide derivative) and **26n** (IC_50_ 0.39 µM, 2-pyridyl, N-(4-benzoylphenyl)maleimide derivative). Overall, the two most potent compounds across both cell lines were **25n** (mean IC_50_ 1.09 µM) and **26n** (mean IC_50_ 1.12 µM).

#### 2.4.12. National Cancer Institute (NCI) 60-Cell-Line Panel Screening

A number of our most promising compounds identified through preliminary cell viability testing (**23c**, **23d**, **23f**, **23h**, **23l**, **23m**, **23n**, **23o**, **24f**, **24l**, **25n** and **26n**) were selected for NCI drug screening in the 60-cell-line panel. The panel includes nine subcategories based on cancer tissue types, which include leukemia, ovarian, central nervous system, breast, lung, prostate, skin, renal and colon cancer. The compounds were initially assessed in a one-dose assay at a fixed treatment concentration of 10 µM. The compounds selected for further investigation were progressed to a five-dose assay across the concentration ranges of 0.01, 0.1, 1, 10 and 100 µM [72]. The endpoints assessed in the screening protocol were GI_50_, TGI and LC_50_ [72].

As the main focus of this work is the antiproliferative and anti-cancer effects of ethanoanthracenes in CLL, attention was focused primarily on leukemia cell line results within the wider 60-cell-line panel. The leukemia cell lines used for the assessment were as follows: CCRF-CEM (acute lymphoblastic leukemia), HL-60 (TB) (acute promyelocytic leukemia), K-562 (chronic myeloid leukemia), MOLT-4 (acute lymphoblastic leukemia), RPMI-8226 (plasmacytoma/myeloma) and SR (large cell immune blastic lymphoma). The resultant GI_50_ values obtained are presented in Table 5. All compounds demonstrated GI_50_ values in the low and sub-micromolar range across all leukemia cell lines, with compound **25n** identified as the most potent with a mean GI_50_ of 0.29 µM, indicating the potential for the compound for further investigation. The mean GI_50_ values across the leukemia cell panel were determined in the range of 0.29–2.08 µM. Compounds **25n** (0.29 µM), **24l** (0.98 µM), **23h** (1.00 µM) and **23n** (1.32 µM) were the most promising overall anti-leukemic agents screened. These results are in good alignment with those determined in the HG-3 and PGA-1 CLL cell lines (Table 4), with compound **25n** identified as the most potent compound in both CLL cell lines (HG-3, IC_50_ = 1.31 µM; PGA-1, IC_50_ = 0.87 µM and mean IC_50_ = 1.09 µM). They warrant further study as lead compounds for the development of more selective and potent anti-cancer agents.

In addition to the leukemia results above, promising anticancer activity was observed across the NCI 60-cell-line panel with particularly interesting activity in melanoma and breast cancer cell lines and notable GI_50_ value ranges of **25n** (melanoma: 0.194–1.08 µM; breast: 0.295–0.627 µM), **23h** (melanoma: 1.60–2.48 µM; breast: 0.885–1.88 µM), **23n** (melanoma: 1.12–2.14 µM; breast: 0.295–0.627 µM) and **23l** (melanoma: 1.40–1.81 µM; breast: 1.34–1.94 µM) (Table 6 and Table 7). These values suggest the potential exploration of these compounds in breast and skin cancers in the future.

**Figure 8:**
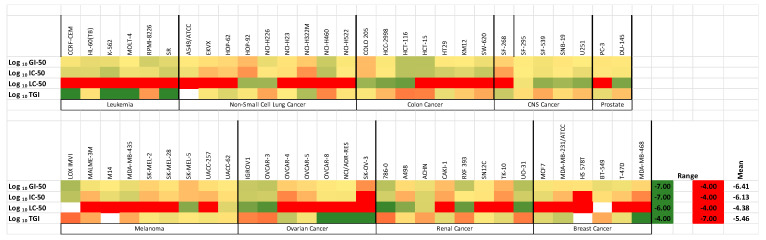
Heatmap for compound **25n** across cell lines in the NCI-60 screen.

The *N*-(4-chlorophenyl)maleimide ethanoanthracene adduct **25n** was identified as the most potent compound evaluated in the NCI panel, with a mean GI_50_ value of 0.245 nM determined across the NCI-60 panel of cell lines screened. (See Table 6: NCI-60 cell line screen, Figure 8: Heatmap for the activity of compound **25n** in the cell lines of the NCI-60 screen). Significantly, the GI_50_ values for **25n** were in the sub-micromolar range for all but six of the cell lines investigated. **25n** displayed significant potency in all breast cancer cell lines evaluated in the panel, with GI_50_ values in the range of 312–0.627 µM. GI_50_ values below 300 nM were obtained for compound **25n** in 17 of the panel cell lines tested with activity against non-small-cell lung, colon, CNS, ovarian and prostate cell lines tested. Figure 8 displays a heatmap of the activity of compound **25n** across the cell lines in the NCI-60 screen. In addition, compound **25n** was particularly effective in the chemoresistant HT-29 human colorectal adenocarcinoma cell line with an IC_50_ value of 0.335 µM. The mean GI_50_ values for the panel of 60 cell lines for the most potent compounds evaluated were determined to be in the range of 0.245–2.511 µM (apart from compound **26n** GI_50_ = 7.585 µM), and they are shown in Table 7.

Heatmap for the antiproliferative activity of compound **25n** across cell lines in the NCI-60 screen using three different values (growth-inhibitory effect, GI_50_; drug concentration at which the response is reduced by half, IC_50_; cytostatic effect, TGI; cytotoxic effect, LC_50_; concentration in molar).

#### 2.4.13. COMPARE Analysis of Compounds **23h**, **23n**, **24l** and **25n**

The COMPARE algorithm [74], allows for the comparison of compound activity profiles across the NCI 60-cell-line panel. Compounds that display similar activity profiles often result in cell growth inhibition through related mechanisms of action and display robust correlations in mean profiles across the screening assays. Representative potent compounds **23h, 23n**, **24l and 25n** were used as COMPARE seeds, with the NCI Standard Agents database selected for comparison. The COMPARE program was then used to probe the mechanism of action of the series of ethanoanthracene maleimide adducts [75]. The antiproliferative profiles (GI_50_, TGI and LC_50_ values) of potent compounds **23h, 23n**, **24l** and **25n** were compared with compounds with a known mechanism of antiproliferative action in the NCI Standard Agents Database. The top five ranked compounds for each seed based on Pearson’s correlation coefficient were identified. Based on GI_50_ values, the chemotherapeutic agents that had fair to strong positive Pearson’s correlation coefficients (0.34–0.92) were identified (Supplementary Information, Tables S3–S6). Compound **23h** demonstrated a good correlation with the antitumor purine analog pentostatin, effective in lymphoid neoplasms and a potent inhibitor of ADA (r = 0.918). The correlation of **23h** with the anticancer ribonucleotide reductase inhibitor caracemide, (r = 0.663) was also observed. **24l** correlates with the DNA-alkylating drugs fluorodopan (r = 0.631) and melphalan (r = 0.538), while **23n** correlated with dichloroallyl lawsone (r = 0.505), an antineoplastic dihydroorotate dehydrogenase (DHOD) inhibitor (see Supplementary Information, Tables S3–S6). Many of the positively correlated chemotherapeutic agents identified in the COMPARE correlation analysis mediate cytotoxicity through DNA synthetic pathways and resultant DNA functionality, which may be relevant in determining the mechanism of action of the ethanoanthracene compounds screened.

#### 2.4.14. Cheminformatics Analysis of Lead Ethanoanthracene Compounds

Estimation of ADME (absorption, distribution, metabolism and excretion) parameters early in the preclinical discovery phase can potentially reduce the number of pharmacokinetics-related drug failures in the clinical phases of drug development [76]. Based on the preliminary antiproliferative studies, a panel of novel compounds (**20a–20e**, **23a**, **23c**, **23d**, **23f–23i**, **23k–23p**, **24f**, **24l**, **25n** and **26n**) was selected for further investigation of their ADME properties using the Swiss ADME cheminformatics webtool [77] (Supplementary Information Figure S2 and Tables S7–S9). The majority of the compounds satisfy the criteria for effective oral drug development, demonstrating logP values in the range of 1.86–4.98, HBD of 0–1, HBA of 3–5, rotatable bonds of 2–6 and TPSA of 54–91 Å^2^. The maleimide and *N*-phenylmaleimide series of compounds are not ionized at a physiological pH, (e.g., **20d**, theoretical pKa value of 10.4, calculated from Chemicalise). High blood–brain barrier (BBB) absorption levels and high GI absorption were predicted for the compounds, indicating the potential of these compounds as lead compounds for further development. **20a** was identified as the most potent analog evaluated in the nitrovinylanthracene series with an IC_50_ value of 0.48 µM (HG-3) and 0.61 µM (PGA-1) cell lines (MW = 422, HBD = 0, HBA = 4, Rot bonds = 3, cLogP 3.29, TPSA = 83.20 Å^2^). The lead compounds tested were not predicted to inhibit the metabolic activity of CYP2D6 or CYP3A4.

The aqueous solubility predicted for the compounds was in the range of 0.011–61.4 µg/mL, with the compounds **20d** and **23m** predicted to have the highest solubility in the series (61.4 µg/mL and 29.8 µg/mL, respectively). Potential correlations were assessed between the estimated physicochemical properties and biological activity. A negative correlation was determined for molar refractivity, log P and the skin permeation coefficient, while a positive correlation was observed for water solubility and may contribute to the identification of suitable lead compounds.

Pan Assay Interference Compounds (PAINS) contain functional groups or fragments that contribute to high reactivity. Lead compounds (**20a–20e**, **23a**, **23c**, **23d**, **23f–23i**, **23k–23p**, **24f**, **24l**, **25n** and **26n**) were screened according to PAINS filters to reduce the possibility of such compounds being selected for progression and optimization. Despite the presence of the α,β-unsaturated ketone and nitrovinyl group (Michael acceptors) in the lead molecules considered, no PAINS alerts were flagged (Supplementary Information Table S9) [77]. The Brenk filter (used to identify fragments known to induce toxicity directly or post-metabolic activation for highly reactive and metabolically labile functional groups) identified two structural alerts in the series: the maleimide bridgehead structure and the α,β-unsaturated ketone (Michael acceptor) [77].

#### 2.4.15. In vitro LDH Cytotoxicity Investigation of Selected Lead Compounds **20a**, **20f**, **23a** and **25n**

**Figure 9:**
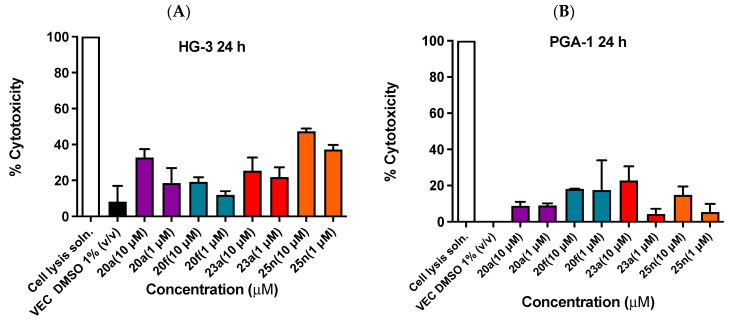
LDH assay for cytotoxicity of compounds **20a**, **20f**, **23a** and **25n** in the HG-3 (Panel **A**) and PGA-1 (Panel **B**) cell lines.

Further biochemical studies were then investigated for selected lead compounds **20a**, **20f**, **23a** and **25n** based on their potency in the antiproliferative assays in CLL cells. The lactate dehydrogenase (LDH) assay is used to assess the membrane integrity of cells as a function of the cytoplasmic LDH released. The loss of cell membrane integrity (due to cytotoxic insult) is detectable through LDH release into the cell growth medium. The assay is initiated via the reduction in cellular co-factor NAD^+^ to NADH catalyzed via the LDH enzyme. Newly formed NADH catalyzes the acidic reduction in iodonitrotetrazolium chloride (INT) to the highly colored formazan (red) (mediated via the diaphorase enzyme) [78,79]. The results for the LDH cytotoxicity assay of the HG-3 cell line for the selected compounds **20a**, **20f**, **23a** and **25n** (which were representative of the most potent ethanoanthracene compounds identified in preliminary biochemical screening) are shown in Figure 9A. At the 10-µM treatment concentration, mild to moderate cytotoxicity (19–47%) was observed. The nitrovinyl dimer **20f** displayed the lowest LDH release at 19%, with **23a** (chalcone maleimide, 25%), **20a** (nitrovinyl *N*-phenylmaleimide 33%) and **25n** (chalcone, *N*-(4-chlorophenyl)maleimide) having the highest cytotoxicity (47%). At the 1-µM treatment concentration, cytotoxicity remained mild to moderate with an observed range of 12–37% with **20f** displaying the lowest % LDH release (12%). In the PGA-1 cell line (Figure 9B), **20a** displayed the lowest LDH release (9%) at 10 µM, followed by **25n** (15%), **20f** (19%) and **23a** (23%). Cytotoxicity remained low to mild at 1 µM (4–18%) with **23a** displaying the lowest LDH release (4%). Interestingly, the assessed compounds were observed to cause a greater degree of LDH release from HG-3 cells compared to PGA-1, suggesting greater cytotoxicity against more aggressive variants of CLL. In addition, the mean TGI (total growth inhibition) value for the potent compound **25n** (mean GI_50_: 0.245 µM) over the NCI 60-cancer-cell-line panel was 18.62 µM, indicating a wide therapeutic window for the compound (Table 7).

Following the treatment of the HG-3 and PGA-1 cells at 10 µM for 24 hr, the amount of LDH released was determined using an LDH assay kit from Promega (G1780). Cell lysis solution = 100% cell lysis and LDH release). Data are presented as % cell death at a concentration of 10 µM. Cells were treated with ethanoanthracene compounds **20a**, **20f**, **23a** and **25n** (1 µM and 10 µM) for 24 h. Values are shown as the mean of three independent experiments.

#### 2.4.16. Pro-Apoptotic Effects of Selected Compounds **20a**, **20f**, **23a** and **25n** in HG-3 Cells and PGA-1 Cells

The pro-apoptotic effects of selected nitrovinylethanoanthracene compounds **20a** and **20f** and anthracene chalcones **23a** and **25n** in HG-3 cells and PGA-1 cells was then investigated using FITC (fluorescein isothiocyanate), Annexin V/PI (propidium iodide) and FACS (fluorescence-activated cell sorting) analysis to characterize the mode of cellular death induced. Apoptosis was assessed as % total apoptosis (total of early (Q3) and late (Q2) apoptosis).

**Figure 10:**
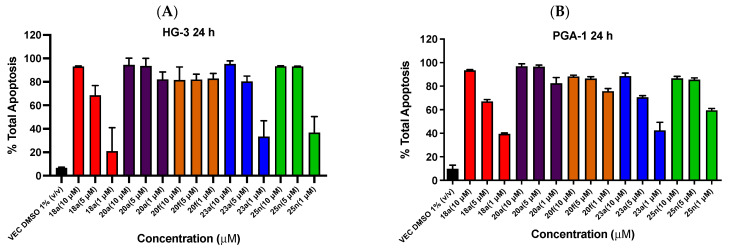

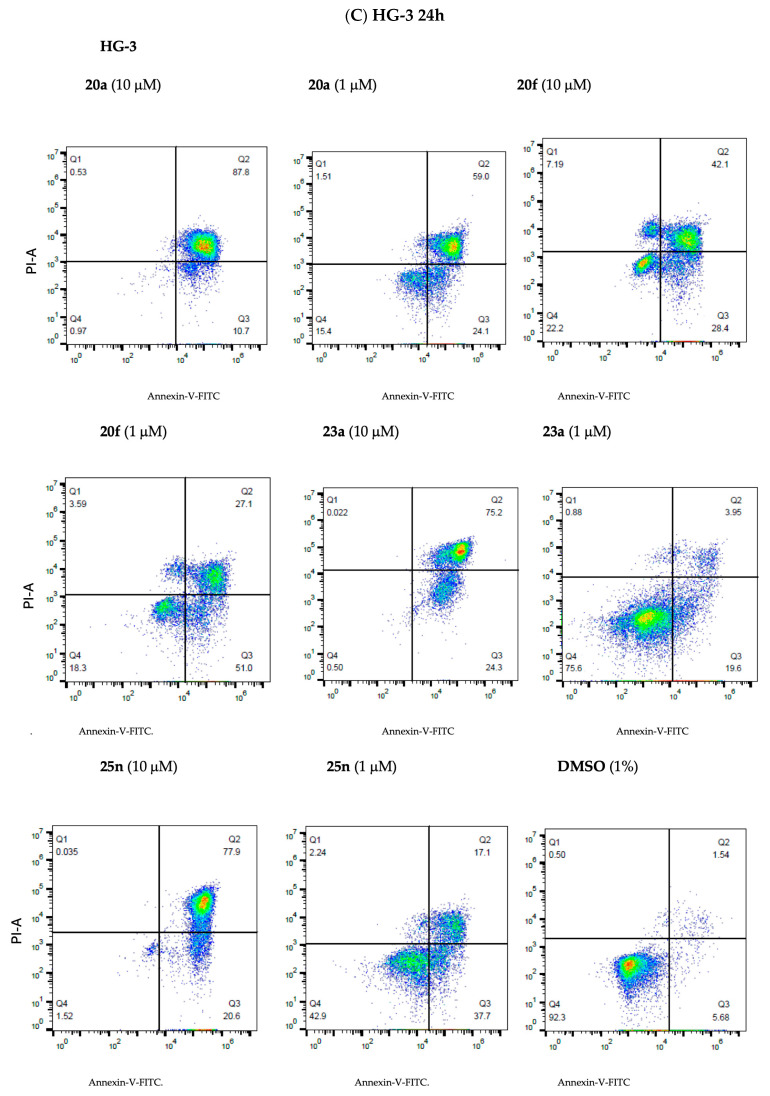

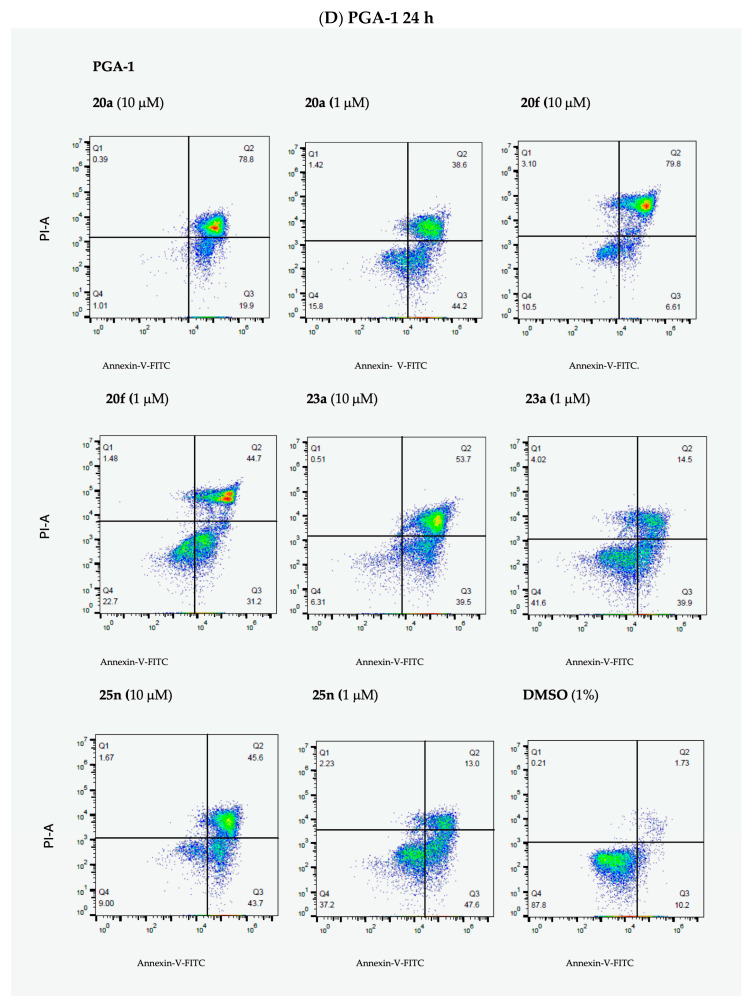
Ethanoanthracene nitrostyrene compounds **18a**, **20a**, **20f**, **23a** and **25n** induce apoptosis in HG-3 and PGA-1 CLL cells. Compounds **18a**, **20a**, **20f**, **23a** and **25n** potently induce apoptosis in HG-3 and PGA-1 cell lines (Annexin V/PI FACS). HG-3 and PGA-1 leukemia cells were treated with **18a**, **20a**, **20f**, **23a** and **25n** (10 µM, 5 µM and 1 µM) and a control vehicle [(1% DMSO (*v*/*v*))] at 48 h for panels **A** and **B,** respectively. The % of apoptotic cells was determined by staining with Annexin V-FITC and PI (Panels **C** and **D** show compounds **20a**, **20f**, **23a** and **25n** at 1- and 10-µM concentrations). The lower left quadrant cells are negative for both Annexin V-FITC and PI, and the upper left shows PI cells that are necrotic. The lower right quadrant shows Annexin-positive cells in the early apoptotic stage, and the upper right shows both Annexin- and PI-positive cells in the late apoptosis stage. The experiment was replicated on three independent days.

The results from the Annexin V/PI studies of representative potent lead ethanoanthracene compounds **20a**, **20f**, **23a** and **25n** are shown in Figure 10, together with our previously reported lead nitrovinylanthracene compound **18a** [40]. In HG-3 cells, all compounds produced significant apoptosis that was largely concentration-dependent (Figure 10A,C). At the 10-µM concentration, the most active compounds with 95% total apoptosis were **23a** (4-bromophenylchalcone, maleimide) and **20a** (nitrostyrene, *N*-phenylmaleimide), followed by **25n** (2-pyridylchalcone, *N*-(4-chlorophenyl)maleimide) and **20f** (nitrostyrene dimer) with 93% and 82% apoptosis, respectively. At a 1-µM treatment concentration, the most potent pro-apoptotic effects were identified for the nitrostyrene derivatives **20a** and **20f** (both inducing 82% total apoptosis), while the chalcone ethanoanthracenes **25n** (37%) and **23a** (33% apoptosis) were less effective. The pro-apoptotic effect of nitrostyrene dimer **20f** remained constant across all three concentrations, possibly associated with the potent antiproliferative effect in HG-3 cells (IC_50_ value of 0.17 µM). In PGA-1 cells, all compounds produced significant concentration-dependent apoptosis (Figure 10B,D). At the 10-µM treatment concentration, the nitrostyrene **20a** induced the greatest apoptosis (97% total apoptosis), followed by **23a** (89%), **20f** (88%) and **25n** (87%). At the 1-µM treatment concentration, the nitrovinyl compounds **20a** and **20f** produced the most potent effect with 83% and 76% apoptosis, respectively. These results from compounds **20a**, **23a**, **25n** and **20f** compare favorably with the effect of the clinically used drug fludarabine phosphate, which was found to induce a poorer increase in apoptosis (24.6%) for cancer cells isolated from CLL patients [39]. In summary, the results across both cell lines indicate that compounds **20a**, **20f**, **23a** and **25n** induce significant apoptosis in a largely concentration-dependent manner and support a pro-apoptotic mechanism of action for this class of ethanoanthracene compounds.

#### 2.4.17. Ex Vivo Assessment of Selected Compounds **20a**, **23a** and **25n** in Donors’ Peripheral Blood Mononuclear Cells (PBMCs)

**Figure 11:**
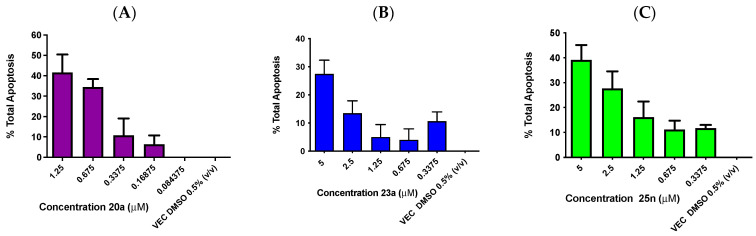
Percentage of total apoptosis observed upon treatment of isolated donor PBMCs with compounds **20a**, **23a** and **25n**. Ethanoanthracene compounds **20a**, **23a** and **25n** induced apoptosis upon the treatment of isolated human-donor peripheral blood mononuclear cells (PBMCs). Isolated donor PBMCs were treated with compound **20a** (Panel **A**) at 1.25 µM, 0.68 µM, 0.34 µM, 0.17 µM and 0.08 µM concentrations, compounds **23a** (Panel **B**) and **25n** (Panel **C**) at 5 µM, 2.5 µM, 1.25 µM, 0.68 µM and 0.34 µM concentrations, and all were normalized against a control vehicle (0.5% DMSO (*v*/*v*)) at 48 h. The % of apoptotic cells was determined via staining with Annexin V-FITC and PI. The experiment was performed individually and replicated on three independent days.

Representative examples of the most potent ethanoanthracene compounds **20a**, **23a** and **25n** were assessed in donor peripheral blood mononuclear cells (PBMCs). Whole blood samples from healthy donors (×5) were isolated, prepared and treated following the protocol previously described [40]. The percentage of apoptosis observed in the treatment of isolated donor PBMCs with nitrovinylethanoanthracene **20a** is concentration-dependent (Figure 11A). **20a** exhibited moderate toxicity (40%) towards the healthy-donor lymphocytes at the highest concentration tested of 1.25 µM. When compared to the IC_50_ values in both HG-3 and PGA-1 CLL cell lines of 0.48 µM and 0.61 µM, respectively, relatively low to moderate amounts of apoptotic death in healthy cells was noted (10–35% at concentrations of 0.34 and 0.68 µM). For ethanoanthracene **23a**, the percentage of total apoptosis observed is illustrated in Figure 11B. **23a** exhibited the lowest toxicity of the three compounds evaluated in healthy-donor PBMCs at its highest treatment concentration of 5 µM with observed total apoptosis of 28%. When these results were compared to the IC_50_ values of **23a** in both HG-3 and PGA-1 CLL cell lines of 2.69 µM and 1.97 µM, respectively, very low amounts of lymphocyte cell death were detected (5–14% at 1.25–2.5 µM). The percentage of total apoptosis observed in the treatment of isolated donor PBMCs with the anthracene–chalcone **25n** (normalized against vehicle 0.5% *v*/*v* DMSO) is illustrated in Figure 11C, and the dose-response behavior was also concentration-dependent, with compound **25n** moderately toxic to healthy-donor PBMCs when treated at a 5.00 µM concentration (39%). Relatively low toxicity (12–16%) was observed at concentrations representative of the IC_50_ values in both HG-3 and PGA-1 CLL cell lines, which were 1.31 µM and 0.87 µM, respectively.

#### 2.4.18. Effect of Pre-Treatment with Antioxidants on Cellular Viability of HG-3 and PGA-1 CLL Cells

Due to the pivotal role that reactive oxygen species (ROS) play in malignant cell transformation and progression, a wide variety of chemotherapeutic agents that rely on ROS modulation (either directly or indirectly) have been identified e.g., the quinone-based anticancer agents doxorubicin, mitomycin C and bleomycin used clinically for breast, small-cell lung and hematologic cancers [80,81,82].

**Figure 12:**
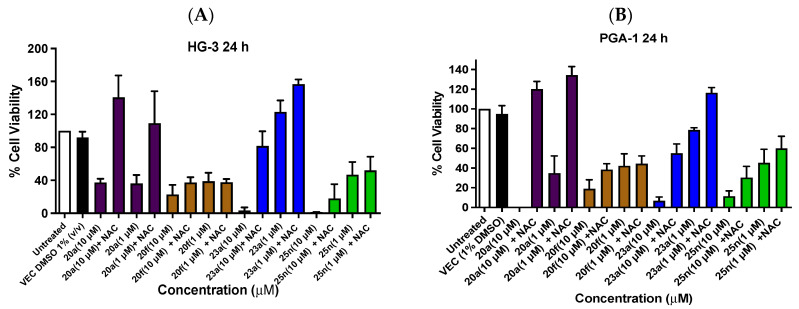
Effect of antioxidant pre-treatment (N-acetylcysteine, NAC) on the viability of HG-3 and PGA-1 CLL cells treated with compounds **20a**, **20f**, **23a** and **25n. The** cell viability of HG-3 and PGA-1 cells was determined with an alamarBlue assay (seeding density: 2 × 10^5^ cells/mL per well for 96-well plates). Compound concentrations of either 1 µM or 10 µM for 24 h were used to treat the HG-3 and PGA-1 CLL cells (in triplicate) with control wells containing vehicle DMSO (1% *v*/*v*). The cells were pre-treated with NAC (2 µL, 5 mM) for 1 h, (Panel **A**,**B**) and protected from light before then being treated with the compound. The mean value for three independent experiments is shown.

Compounds **20a** and **20f** (potent nitrovinyl-based ethanoanthracenes), and **23a** and **25n** (potent chalcone-based ethanoanthracenes), were assessed at treatment concentrations of 10 µM and 1 µM in the absence and presence of the ROS inhibitor N-acetylcysteine (NAC) to assess the potential for the synthesized compounds to mediate their antiproliferative effect through ROS flux. In HG-3 cells, NAC addition impacts the ability of all compounds to reduce cell viability to varying degrees (Figure 12A). With the nitrovinyl **20a** and chalcone compound **23a,** a dramatic increase of 103% and 78%, respectively, in cell viability was noted at a 10-µM treatment concentration, following pre-treatment with NAC. Smaller relative increases in cell viability were observed for chalcone **25n** and nitrovinyl dimer **20f** (17% and 14%, respectively). A similar trend in viability was observed at the 1-µM treatment concentration. In PGA-1 cells, NAC addition also impacts on the ability of all compounds to reduce cell viability (Figure 12B). For **20a,** viability was increased to 120% upon pre-treatment with NAC, while a moderate increase of 48% in cell viability was observed with chalcone **23a**; **25n** and **20f** showed little change in cell viability. At the lower 1-µM treatment concentration, **20a** was the most effective with a 92% increase in cell viability. These results strongly suggest a potential ROS-dependent mechanism for chalcone-based compound **23a** and nitrovinyl-based compound **20a**, and they support our previous reports of the ROS-dependent action of nitrovinyl-based ethanoantracenes in BL cell lines [43]; meanwhile, the antiproliferative action of chalcone **25n** and nitrovinyl dimer **20f** showed less dependence on ROS.

#### 2.4.19. Caspase Inhibition Assay

**Figure 13:**
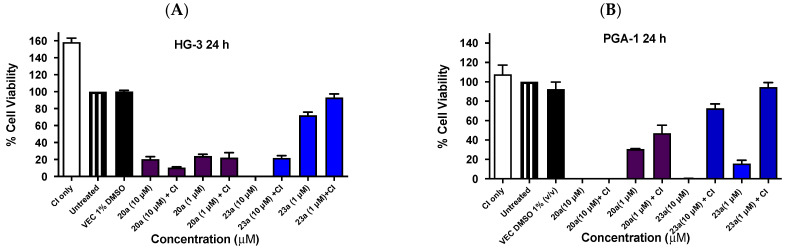
Effect of pre-treatment with caspase inhibitor Z-VAD-FMK on HG-3 and PGA-1 cell viability for compounds **20a** and **23a**. Cell viability analysis (24 h) for inhibitor studies of compounds **20a** and **23a** in HG-3 (Panel **A**) and PGA-1 (Panel **B**) CLL cell lines: the HG-3 and PGA-1 CLL cells (2 × 10^5^ cells/mL) were pre-treated at 37 °C with 40 µM of caspase inhibitor (CI) (Z-VAD-FMK) for 4 h prior to compound treatment at 1 µM and 10 µM for 24 h. The cell proliferation of HG-3 and PGA-1 cells was determined with an alamarBlue assay (CI = caspase inhibitor, n = 2).

Caspases (cysteine-aspartic proteases) are a family of aspartic acid residue-specific regulatory cysteine proteases that play an essential role in key cellular homeostatic processes such as inflammation and programmed cell death through the induction of apoptosis [83,84]. To assess whether the biological activity of both major ethanoanthracene classes (nitrostyrene and chalcone-based) is caspase-dependent, compounds **20a** and **23a** (representing Series 1 and Series 4, respectively) (Figure 13) were selected for evaluation at treatment concentrations of 10 µM and 1 µM across both CLL cell lines using a caspase assay protocol. In HG-3 cells, pre-treatment with pan-caspase inhibitor (CI) Z-VAD-FMK [85] (20 µM) resulted in an increase in the viable cells remaining of 24% for compound **23a** (10 µM) and 21% at 1 µM (Figure 13A). In contrast to this, with compound **20a**, a decrease of 10% in cell viability was noted at 10 µM and 2% at 1 µM. Little difference was found between cell viability responses (with or without the caspase inhibitor) at both 10-µM and 1-µM concentrations for compound **20a**. In the PGA-1 cell lines, pre-treatment with Z-VAD-FMK (20 µM) resulted in a 68% increase in viable cells for compound **23a** at 10 µM and an increase of 79% at 1 µM (Figure 13B). For compound **20a**, an increase of 17% at 1 µM was observed, suggesting a correlation between antiproliferative activity and the induction of apoptotic cell death for nitrostyrene compound **20a**. The involvement of caspases in the ability of compound **23a** to reduces cell viability in HG-3 and PGA-1 cells was demonstrated, together with the pro-apoptotic effects of **20a** in PGA-1 cells, suggesting the selective roles of the nitrovinyl and α,β-unsaturated ketone pharmacophores in the induction of apoptosis via caspases.

### 2.5. Computational Study

#### 2.5.1. Molecular Modeling Study

The synthesized ethanoanthracene compounds (series 1–7) are structurally related to the antidepressant maprotiline **16,** which displays antiproliferative effects in B-cell malignancies and may have similar cellular antiproliferative effects. To examine the structural similarities, selected compounds (**20a–20f**, **23a**, **23c**, **23f–23i**, **23k**, **23l**, **23n**, **23p**, **24f**, **24l**, **25n** and **26n**) were overlaid on maprotiline using MOE flexible alignment, as described in our previous work; this approach is based on similarity terms such as hydrogen bond donor/acceptor, aromaticity and partial charge [40]. Similar functional groups in each molecule are flexibly aligned and superimposed using a stochastic search procedure while the full conformational flexibility for each structure is samples. All databases and reference structures are provided in the Supplementary Information as .sdf or .mdb files.

An overlay of the most potent ethanoanthracenes **20a**, **20b**, **20d**, **20f**, **23a** and **25n** (displayed as green in their respective overlays with maprotiline pink) and reference nitrovinylanthracene compound **18b**, together with the IC_50_ values in the HG-3 and PGA-1 CLL cell lines, is shown in Table 8. The overlay scores of the enthanoanthracene compounds were lower (better) than the lead nitrostyrene compound **18b**. Common molecular features were clearly identified, e.g., the ethanoanthracene structure that overlays with the 9,10-dihydroanthracene core structure of maprotiline, whereas the nitrostyrene and α,β-unsaturated ketone mapped closely to the cyclic core of maprotiline. The MOE flexible alignment demonstrated a lack of correlation between the lowest-(best-) scored compounds and those with the most promising cellular data for the CLL cell lines (Table 8). The overlay results obtained for all the ethanoanthracene compounds in this study are inconclusive (Table S10, Supplementary Information), and they suggest that these compounds may have a different mechanism of action when compared with maprotiline.

#### 2.5.2. In Silico Target Prediction

Computational modeling was investigated to gain insight into the possible mechanisms of action of the ethanoanthracenes. Selected novel compounds (**20a**, **20b**, **20d–20f**, **23a**, **23c**, **23f–23i**, **23k**, **23l**, **23n**, **23p**, **24f**, **24l**, **25n** and **26n**), together with lead anthracene **18b** and maprotiline, were submitted to the SwissTargetPrediction service (STP) [86] for evaluation. The STP methodology is conceptually rooted in the SAR principle, which states that similar compounds tend to have similar biological activity. Thus, for a given query structure, STP finds structurally similar compounds with known activities and uses them to compile a list of potential targets for the given query structure.

**Figure 14:**
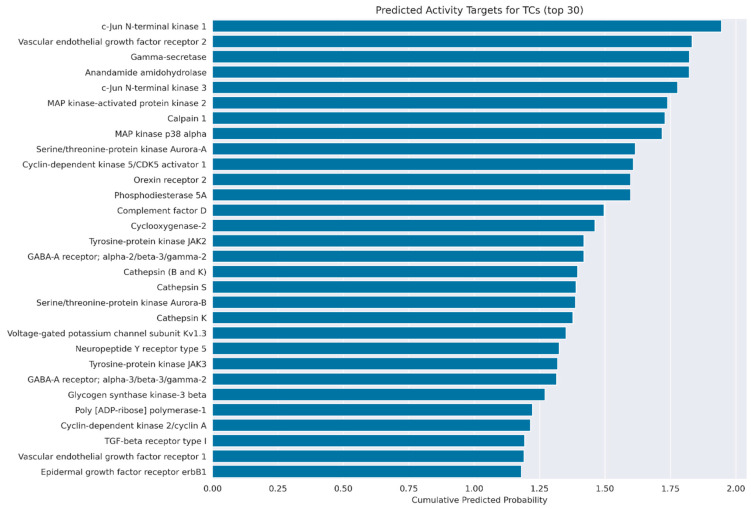
Cumulative probability scores for compounds **20a**, **20b**, **20d–20f**, **23a**, **23c**, **23f–23i**, **23k**, **23l**, **23n**, **23p**, **24f**, **24l**, **25n** and **26n** for the 30 targets most strongly indicated via STP.

STP generated a list of potential targets for each of the submitted compounds. Overall, all predictions were assigned relatively low internal confidence scores via STP, mostly at around 0.05–0.15. However, this was consistent with most predictions for maprotiline itself, except for a full 1.00 prediction confidence score for its several already experimentally confirmed targets. For dimeric compound **20f**, STP was not able to provide predictions with any degree of confidence. The tested compounds share similar in vitro cytotoxic effects, and it could be assumed that these observed effects are caused by the compounds hitting the same unknown targets(s). Thus, targets predicted for the majority of the tested compounds became the focus of the STP-based analysis. To identify the most prevalent predicted targets among the 21 tested compounds, the STP target lists were aggregated by summing the individual prediction confidence scores for each compound. The resulting target list thus contains the cumulative prediction confidences for each given target, within the 21 tested compounds (Figure 14).

**Figure 15:**
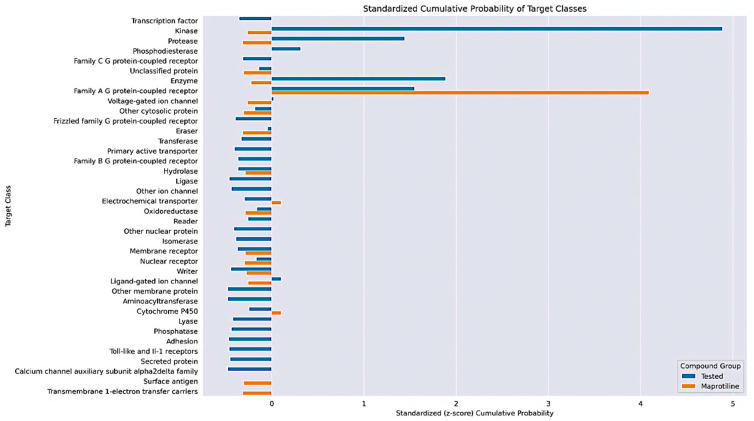
Standardized cumulative probability scores of target groups indicated via STP for the tested compounds **20a**, **20b**, **20d–20f**, **23a**, **23c**, **23f–23i**, **23k**, **23l**, **23n**, **23p**, **24f**, **24l**, **25n** and **26n** compared to Maprotiline. Scores were standardized using z-scores (i.e., differences in standard deviations from their mean).

Since the score contributions of individual compounds are in the 0.1-score range, these targets were found to be a hit by STP for most of the 21 tested compounds. For a more general view of the target types predicted for the tested compounds, the cumulative probability scores for individual targets were further aggregated by summing based on their general protein family groups. To obtain a direct comparison between target groups predicted for the tested compounds and target groups predicted for maprotiline, both results were individually converted to standard z-scores (Figure 15).

The aggregated STP outputs indicate that the tested compounds might, indeed, share some family A GPCR activity with maprotiline, which is a known antagonist of muscarinic acetylcholine receptors, having antiproliferative activity [44]. However, in contrast with maprotiline, there are stronger ligand-similarity hits against a variety of kinases, especially the c-Jun N-terminal (JNK), MAP and VEGFR kinases. These predicted kinase targets are reasonable, as each has a role in promoting cellular proliferation, so blocking them would promote the observed functional phenotype. It is interesting that the COMPARE analysis indicated that the compounds mediate cytotoxicity through DNA synthetic pathways. The in silico target evaluation remains inconclusive, but it offers insight into future investigations for these compounds, e.g., molecular docking with select family-A GPCRs, as well as JNK, MAP and VEGFR kinases.

## 3. Materials and Methods

### 3.1. Chemistry

All reagents were commercially available and were used without any further purification unless otherwise indicated. Melting points were measured on a Stuart Melting Point Apparatus, SMP20, and they are uncorrected. Infra-red (IR) spectra were recorded on a Perkin Elmer FT-IR Paragon 1000 spectrometer. ^1^H and ^13^C nuclear magnetic resonance spectra (NMR) were recorded at 25 °C on a Bruker DPX 400 spectrometer (400.13 MHz, ^1^H; 100.61 MHz, ^13^C), Bruker Avance III 400 or Avance II 600 (400.13 MHz/600.13 MHz, ^1^H; 100.61 MHz/150.61 MHz, ^13^C) in either CDCl_3_ (internal standard tetramethylsilane (TMS)) or DMSO-*d_6_*. For CDCl_3_, ^1^H-NMR spectra were assigned relative to the TMS peak at 0.00 ppm, and ^13^C-NMR spectra were assigned relative to the middle CDCl_3_ peak at 77.0 ppm. For CD_3_OD, ^1^H and ^13^C-NMR spectra were assigned relative to the center peaks of the CD_3_OD multiplets at 3.30 ppm and 49.00 ppm, respectively. Coupling constants are reported in Hertz. High-resolution mass spectrometry (HRMS) was carried out by Dr. Gary Hessman of the School of Chemistry, Trinity College Dublin, using atmospheric pressure chemical ionization (APCI) with the occasional use of electrospray ionization (ESI-MS) in both positive and negative modes. ESI mass spectra were acquired using a Bruker micrOTOF-Q III spectrometer interfaced to a Dionex UltiMate 3000 LC in positive and negative modes, as required. Masses were recorded over the range of 100–1400 *m*/*z*. APCI experiments were carried out on a Bruker micrOTOF-Q III spectrometer interfaced with a Dionex UltiMate 3000 LC or direct insertion probe. The instrument was operated in positive or negative mode, as required. Masses were recorded over a range of 100–1600 *m*/*z*. Mass measurement accuracies of <±5 ppm were obtained. TLC was carried out on silica gel on aluminum foils with fluorescent indicator F-254 nm. Flash-column chromatography was carried out on Merck Kieselgel 60 (particle size: 0.040–0.063 mm). Analytical high-performance liquid chromatography (HPLC) was performed using a Waters 2487 Dual Wavelength Absorbance detector, a Waters 1525 binary HPLC pump and a Waters 717plus Autosampler with a Thermo Scientific Hypersil GOLD C18 reverse-phase 5 µm 150 × 4.6 mm chromatography column. Samples were detected using wavelengths of 232 nm compounds. All samples were analyzed using acetonitrile (75%)–TFA (0.1%) in water (25%) over 15 min and a flow rate of 1 mL/min. Microwave experiments were carried out using the Discover CEM microwave synthesizer on standard power setting (300 watts) unless otherwise stated. Maleimides **19a–c** and (*E*)-9-(2-nitrovinyl)-9,10,11,15- tetrahydro-9,10-[3,4]epipyrroloanthracene-12,14-diones **20a–h** were prepared as we previously reported [43] (see Supplementary Information). (*E*)-3-(Anthracen-9-yl)-1- phenylprop-2-en-1-ones **21a–d**, **21f–j** and **21l–q** were prepared as previously reported [87,88,89,90,91,92,93,94] (see Supplementary Information).

### 3.2. General Procedure for the Preparation of (E)-3-(anthracen-9-yl)-1-phenylprop-2-en-1-ones ***21a–q***

To a solution of NaOH (6 mmol, 0.24 g) in 50% aqueous EtOH (20 mL) was added the appropriate acetophenone (5.02 mmol). After the dissolution of the acetophenone, 9-anthracenecarboxaldehyde (5.02 mmol, 1.035 g) was added, resulting in a colored solution. This solution was stirred at room temperature for 24 h to achieve a colored suspension that was filtered, washed with minimal cold EtOH and dried. The crude product was then recrystallized from the minimal amount of EtOH or MeOH to afford a pure product.

(*E*)-3-(Anthracen-9-yl)-1-(3,4-dimethoxyphenyl)prop-2-en-1-one (**21e**) was synthesized from 3′,4′-dimethoxyacetophenone (5.02 mmol, 0.904 g) and 9-anthracenecarboxaldehyde (5.02 mmol, 1.035 g), according to the general procedure above; yellow powder (85%) Mp. 138–139 °C. ^1^H NMR (400 MHz, CDCl_3_) δ 3.97 (d, *J* = 11.61 Hz, 6 H, 2xCH_3_), 6.91 (d, *J* = 8.71 Hz, 1 H), 7.40–7.53 (m, 4 H, ArH), 7.54–7.58 (d, *J* = 15.76 Hz, 1 H,CH=C), 7.65–7.72 (m, 2 H, ArH), 8.01–8.05 (m, 2H, ArH), 8.29–8.32 (m, 2H, ArH), 8.47 (br.s, 1H, ArH), 8.77 (d, *J* = 15.76 Hz, 1H, ArH). ^13^C NMR (101 MHz, CDCl_3_) ppm 53.07, 53.11, 110.07, 110.85, 123.34, 125.39, 126.31, 128.20, 128.87, 129.61, 130.80, 131.03, 131.31, 141.00, 187.81 (C=O). IR *ν* max (KBr): 3081.44, 3049.16, 2968.80 (Ar C-H), 1656.98 (C=O), 1622.51 (*trans* C=C), 1591.24, 1580.66, 1515.04, 1442.23, 1416.45 (Ar C=C), 1266.73, 1014.80 (C-O) cm^−1^. HRMS (ESI) calculated for C_25_H_20_NaO_3_ [M^+^+Na] 391.1305: found 391.1307.

(*E*)-3-(Anthracen-9-yl)-1-(3,4,5-trimethoxyphenyl)prop-2-en-1-one (**21k**) was synthesized from 1-(3,4,5-trimethoxyphenyl)ethan-1-one (5.02 mmol, 0.65 mL) and 9-anthracenecarboxaldehyde (5.02 mmol, 1.035 g) according to the general procedure above; yellow crystals (86%), Mp. 160–164 °C. ^1^H NMR (400 MHz, CDCl_3_) δ 3.90–3.99 (m, 9H), 7.35 (s, 2 H), 7.49–7.56 (m, 5H), 8.03–8.08 (m, 2H), 8.28–8.35 (m, H), 8.50 (s, 1 H), 8.79 (d, *J* = 15.76 Hz, 1H). ^13^C NMR (101 MHz, CDCl_3_) ppm. 56.35, 61.00, 61.45, 62.52, 106.22, 125.38, 126.40, 128.37, 128.92, 129.62, 130.27, 130.70–131.49, 133.10, 141.70, 153.24, 188.33 (C=O). IR *ν* max (KBr): 3045.78 (Ar C-H), 1657.91 (C=O), 1592.99 (Ar C=C), 1268.62 (C-O) cm^−1^. HRMS (APCI) calculated for C_26_H_22_NaO_4_ [M^+^+Na]: 421.1421; found: 421.1410.

### 3.3. General Preparation for (E)-9-(3-oxo-3-phenylprop-1-en-1-yl)-9,10-dihydro-9,10-[3,4]furanoanthracene-12,14-diones ***22a-q***

To a solution of the appropriate chalcone anthracene analog **21a-q** (1.0 mmol) in toluene (2 mL) was added dienophile maleic anhydride (1.3 mmol). The mixture was stirred and heated at 90 °C for 48 h. The reaction mixture was then cooled to room temperature, and the resulting solid was isolated via filtration. The solid product was washed with toluene (2 mL) and diethyl ether (2 mL) and then recrystallized from toluene.

9-(*E*)-3-(4-Bromophenyl)-3-oxoprop-1-en-1-yl)-9,10-dihydro-9,10-[3,4]furanoanthracene-12,14-dione (**22a**) was prepared from (*E*)-3-(anthracen-9-yl)-1-(4-bromophenyl)prop-2-en-1-one (1.0 mmol, 0.3873 g) and maleic anhydride (1.3 mmol, 0.13 g) according to the general procedure; light yellow powder (86%), Mp. 254–256 °C [51]. ^1^H NMR (400 MHz, CDCl_3_) δ 3.65 (dd, *J* = 9.12, 3.32 Hz, 1 H, CH), 3.81 (d, *J* = 9.12 Hz, 1 H, CH), 4.87 (d, *J* = 3.32 Hz, 1 H, CH), 7.16–7.36 (m, 6 H,5 x ArH, 1xC=CH), 7.39–7.44 (m, 2 H), 7.66–7.76 (m, 3 H, ArH), 7.95 (d, *J* = 16.17 Hz, 1 H, C=CH), 8.03 (d, *J* = 8.71 Hz, 2 H, ArH)^13^C NMR (101 MHz, CDCl_3_) ppm 45.46, 48.96, 49.28, 51.93, 123.52, 123.87, 124.18, 127.12, 127.63, 130.39, 130.92, 132.16, 136.24, 137.36, 138.85, 140.22, 141.51, 168.82, 169.74, 188.80 (C=O). IR *ν* max (KBr): 3328.89, 3074.11 (Ar C-H), 1773.04, 1665.51 (C=O), 1617.10, *trans* (C=C), 1581.33, 1458.65, 1397.18, 1076.35 (C-O) cm^−1^. LRMS (APCI) 485.04 [M^+^+H].

9-(*E*)-3-(4-Nitrophenyl)-3-oxoprop-1-en-1-yl)-9,10-dihydro-9,10-[3,4]furanoanthracene-12,14-dione (**22b**) was prepared from (*E*)-3-(anthracen-9-yl)-1-(4-nitrophenyl)prop-2-en-1-one (1.0 mmol, 0.3534 g) and maleic anhydride (1.3 mmol, 0.13 g) according to the general procedure above; cream powder (77%), Mp. >200 °C. ^1^H NMR (400 MHz, DMSO-*d_6_*) δ 3.80 (dd, *J* = 9.12, 3.32 Hz, 1 H), 4.25 (d, *J* = 9.12 Hz, 1 H), 4.95 (d, *J* = 3.32 Hz, 1 H), 7.16–7.28 (m, 3 H), 7.31–7.36 (m, 1 H), 7.41 (dd, *J* = 5.60, 3.11 Hz, 1 H), 7.53–7.57 (m, 1 H), 7.55 (d, *J* = 7.05 Hz, 1 H), 7.86 (s, 1 H), 8.34–8.44 (m, 4 H). ^13^C NMR (101 MHz, DMSO-*d_6_*) 49.73, 49.78, 51.71, 56.89, 109.99, 123.47, 124.01, 124.46, 124.51, 125.00, 125.74, 125.77, 127.03, 127.53, 127.94, 130.55, 132.01, 138.92, 139.80, 141.19, 142.06, 142.92, 150.52, 170.66, 171.37, 189.02 (chalcone C=O) ppm. IR *ν* max (KBr): 2972.12, (C-H), 1770.72 (C=O), 1667.96, (Ar C=C), 1596.43, (NO_2_), 1516.78, 1346.74 (C-O) cm^−1^. HRMS (APCI) calculated for C_27_H_16_NO_6_ [M^+^−H]: 450.0983; found: 450.0982.

(*E*)-3-(4-Ethylphenyl)-3-oxoprop-1-en-1-yl)-9,10-dihydro-9,10-[3,4]furanoanthracene-12,14-dione (**22c**) was prepared from (*E*)-3-(anthracen-9-yl)-1-(4-ethylphenyl)prop-2-en-1-one (1.0 mmol, 0.336 g) and maleic anhydride (1.3 mmol, 0.13 g) according to the general procedure above; cream powder (70%), Mp. 195–200 °C. ^1^H NMR (400 MHz, DMSO-*d_6_*) δ 1.16–1.23 (m, 3H), 2.70 (q, *J* = 7.46 Hz, 7H), 3.79 (dd, *J* = 9.12, 3.32 Hz, 4H), 4.25 (d, *J* = 9.12 Hz, 4H), 4.94 (d, *J* = 3.32 Hz, 4H), 7.16–7.20 (m, 8H), 7.20–7.23 (m, H), 7.23–7.27 (m, 9H), 7.28–7.33 (m, 1H), 7.37–7.42 (m, 4H), 7.43 (d, *J* = 8.29 Hz, 2H), 7.54 (d, *J* = 6.63 Hz, 4H), 7.74–7.80 (m, 4H), 7.82–7.88 (m, 4H), 8.09 (d, *J* = 8.29 Hz, 7H). ^13^C NMR (101 MHz, DMSO-*d_6_*) ppm: 15.65, 28.70, 44.80, 49.37, 49.60, 51.58, 123.37, 123.91, 125.77, 127.03, 127.47, 127.79, 129.40, 132.06, 135.37, 138.95, 140.03, 140.65, 141.25, 142.31, 150.55, 170.57, 171.42, 189.00 (chalcone C=O) IR *ν* max (ATR): 3071.36, 2882.33 (Ar C-H), 1778.67, 1670.45 (C=O), 1619.17 (C=C), 1604.57, 1580.44 (Ar C=C), 1465.80 (CH), 1374.93 (CH), 1224.51 (C-O) cm^−1^. HRMS (APCI): calculated for C_29_H_23_O_4_: 435.1591 [M^+^+H]; found: 435.1595.

9-(*E*)-3-(2,4-Dichlorophenyl)-3-oxoprop-1-en-1-yl)-9,10-dihydro-9,10-[3,4]furanoanthracene-12,14-dione (**22d**) was prepared from (*E*)-3-(anthracen-9-yl)-1-(2,4-dichlorophenyl)prop-2-en-1-one (1.0 mmol, 0.377 g) and maleic anhydride (1.3 mmol, 0.13 g) according to the general procedure above; beige powder (76%), Mp. 232–236 °C. ^1^H NMR (400 MHz, CDCl_3_) δ 3.60 (dd, *J* = 9.12, 3.32 Hz, 1H), 3.72 (d, *J* = 9.54 Hz, 1H), 4.84 (d, *J* = 2.90 Hz, H), 7.20–7.28 (m, 5H, ArH), 7.28–7.34 (m, 2 H, ArH, 1, CH=C), 7.35–7.39 (m, 1H, ArH), 7.39–7.44 (m, 2H, ArH), 7.52 (d, *J* = 1.66 Hz, 1H, ArH), 7.68 (d, *J* = 8.29 Hz, 1H, ArH), 7.75 (d, *J* = 16.59 Hz, 1H, CH=C). ^13^C NMR (101 MHz, CDCl_3_) ppm 45.49, 49.12, 51.74, 123.44, 123.73, 124.30, 125.64, 127.11, 127.63, 127.92, 128.16, 130.46, 130.95, 134.60, 140.21, 141.14, 143.00, 168.53, 169.72, 191.11 (chalcone C=O) ppm. IR *ν* max (KBr): 3071.19 (Ar C-H), 1776.62, 1662.20 (C=O), 1619.95 (C=C), 1580.28, 1456.96 (Ar C=C), 1212.92 (C-O) cm^−1^. HRMS (APCI) calculated for C_27_H_17_Cl_2_O_4_: 475.0498 [M^+^+H]; found 475.0491.

9-(*E*)-3-(3,4-Dimethoxyphenyl)-3-oxoprop-1-en-1-yl)-9,10-dihydro-9,10-[3,4]furanoanthracene-12,14-dione (**22e**) was prepared from (*E*)-3-(anthracen-9-yl)-1-(3,4-dimethoxyphenyl)prop-2-en-1-one (1.0 mmol, 0.3684 g) and maleic anhydride (1.3 mmol, 0.13 g) according to the general procedure above; cream/yellow powder (83%), Mp. 243–246 °C. ^1^H NMR (400 MHz, CDCl_3_) δ 3.63 (dd, *J* = 9.33, 3.11 Hz, 1H, CH), 3.81 (d, *J* = 9.54 Hz, 1H, CH), 3.87 (s, 3H, OCH_3_), 3.89 (s, 3H, OCH_3_), 4.85 (d, *J* = 3.32 Hz, 1H, CH), 6.96 (d, *J* = 8.29 Hz, 1H, ArH), 7.19–7.27 (m, 4H, ArH), 7.31–7.37 (m, 2H, ArH), 7.37–7.43 (m, 2H, ArH), 7.73–7.76 (m, 2H, ArH, CH=C), 7.78 (s, 1 H), 7.82 (dd, *J* = 8.29, 2.07 Hz, 1 H, ArH), 7.87 (d, *J* = 16.2 Hz, 1H, CH=C) ppm. ^13^C NMR (101 MHz, CDCl_3_) ppm 49.30, 54.1, 54.38, 56.31, 60.74, 61.03, 110.27, 111.02, 123.59, 123.70, 124.07, 125.53, 127.08, 127.54, 127.89, 128.04, 130.68, 131.47, 137.43, 139.09, 139.75, 140.26, 141.75, 149.30, 153.67, 175.36, 176.17, 192.77 (chalcone C=O). IR *ν* max (ATR): 3071.19 (Ar C-H), 1777.39, 1668.50 (C=O), 1619.46 (C=C), 1580.35, 1456.83 (Ar C=C), 1213.57, 1018.24 (C-O) cm^−1^. HRMS (ESI): calculated for C_29_H_22_NaO_6_; 489.1309 [M^+^−H] found: 489.1305.

(*E*)-9-(3-Oxo-3-phenylprop-1-en-1-yl)-9,10-dihydro-9,10-[3,4]furanoanthracene-12,14-dione (**22f**) was synthesized from (*E*)-3-(anthracene-9-yl)-1-phenylprop-2-en-1-one (1.0 mmol, 0.308 g) and maleic anhydride, (1.3 mmol, 0.13 g) according to the general procedure above; white powder (91%), Mp 214–218 °C. ^1^H NMR (400 MHz, DMSO-*d_6_*) δ 3.81 (dd, *J* = 9.12, 3.32 Hz, 1H), 4.27 (d, *J* = 9.12 Hz, 1H), 4.96 (d, *J* = 3.32 Hz, 1H), 7.19–7.29 (m, 5H), 7.30–7.35 (m, 1H), 7.39–7.45 (m, 1H), 7.56 (d, *J* = 6.63 Hz, 1H), 7.58–7.65 (m, 2H), 7.68–7.74 (m, 1H), 7.77–7.92 (m, 2H), 8.14–8.21 (m, 2H). ^13^C NMR (101 MHz, DMSO-*d_6_*) 38.57–41.35, 44.84, 48.69–50.08, 51.64, 122.68–124.27,124.99, 125.48–125.98, 127.06, 127.40–128.13, 128.64, 128.91–129.60, 132.07, 134.01, 138.96,141.21, 142.29, 170.61, 171.43, 189.75 (chalcone C=O) ppm. IR *ν* max (ATR): 1774.31 (C=O), 1634.26 (C=C), 1221.80 (C-O) cm^−1^. HRMS (APCI): calculated for C_27_H_18_O_4_ [M^+^+Na]:429.1097; found 429.1101.

9-(*E*)-3-(4-Iodophenyl)-3-oxoprop-1-en-1-yl)-9,10-dihydro-9,10-[3,4]furanoanthracene-12,14-dione (**22g**) was prepared from (*E*)-3-(anthracen-9-yl)-1-(4-iodophenyl)prop-2-en-1-one (1.0 mmol, 0.434g) and maleic anhydride (1.3 mmol, 0.13 g) according to the general procedure above, light yellow powder (81%), Mp. 228–231 °C. ^1^H NMR (400 MHz, CDCl_3_) δ 3.63 (dd, *J* = 9.12, 2.90 Hz, 1H, CH), 3.79 (d, *J* = 9.12 Hz, 1H, CH), 4.86 (d, *J* = 3.32 Hz, 1H, CH), 7.15–7.34 (m, 6H, ArH), 7.36–7.44 (m, 2 H, ArH), 7.70 (d, *J* = 16.17 Hz, 1H, CH=C), 7.83–7.97 (m, 5 H, ArH, CH=C). ^13^C NMR (101 MHz, CDCl_3_) ppm 45.46, 48.97, 49.27, 51.93, 123.53, 123.88, 124.20, 125.59, 127.12, 127.63, 127.95, 128.13, 130.24, 130.88, 136.77, 137.38, 138.17, 138.86, 140.22, 141.53, 168.83, 189.12 (chalcone C=O). IR *ν* max (ATR): (C-H) 3016.49, 2876.80, (*trans* C=C) 1861.84, (C=O) 1771.38, (Ar C=C) 1619.73, 1579.29, 1479.43, 1456.94, (C-O) 1292.32, (C-I) 619.66 cm^−1^. LRMS (APCI): 531.20 [M^+^−H]. HRMS (APCI): calculated for C_27_H_16_IO_4_ [M^+^−H]: 531.0099; found 531.0102.

9-(*E*)-3-(4-Fluorophenyl)-3-oxoprop-1-en-1-yl)-9,10-dihydro-9,10-[3,4]furanoanthracene-12,14-dione (**22h**) was prepared from (*E*)-3-(anthracen-9-yl)-1-(4-fluorophenyl)prop-2-en-1-one (1.0 mmol, 0.3264 g) and maleic anhydride (1.3 mmol, 0.13 g) according to the general procedure above; cream/yellow powder (79%), Mp. 242–247 °C. (HPLC: 97.8%, RT 6.17 min). ^1^H NMR (400 MHz, CDCl_3_) δ 3.64 (dd, *J* = 9.54, 3.32 Hz, 1H, CH), 3.81 (d, *J* = 9.12 Hz, 1H, CH), 4.87 (d, *J* = 3.32 Hz, 1H, CH), 7.18–7.28 (m, 6H, ArH), 7.29–7.36 (m, 2 H, ArH), 7.36–7.45 (m, 2H, ArH), 7.75 (d, *J* = 16.59 Hz, 1H, CH=C), 7.93 (d, *J* = 16.59 Hz, CH=C), 8.16–8.24 (m, 2H, ArH). ^13^C NMR (101 MHz, CDCl_3_) ppm 45.47, 48.96, 49.30, 51.91, 115.73–116.31, 123.54, 123.91, 124.17, 125.58, 127.12, 127.61, 128.03, 131.06, 131.59, 137.38, 140.23, 141.09, 141.58,168.86, 169.78, 188.26 (chalcone C=O). IR *ν* max (ATR): 3064.79, 2968.92 (Ar C-H), 1773.01 (C=O),1667.75 (C=C), 1625.39, 1585.13, 1481.57, 1458.78 (Ar C=C), 1292.88 (C-F), 1069.82 (C-O) cm^−1^. LRMS (APCI): 423.23 [M^+^−H]. HRMS (APCI): calculated for C_27_H_16_FO_4_ [M^+^−H]: 423.103811; found 423.103417.

(*E*)-9-(3-(4-Methoxyphenyl)-3-oxoprop-1-en-1-yl)-9,10-dihydro-9,10-[3,4]furanoanthracene-12,14-dione (**22i**) was synthesized from (*E*)-3-(anthracen-9-yl)-1-(4-methoxyphenyl)prop-2-en-1-one (1.0 mmol, 0.338 g) and maleic anhydride (1.3 mmol, 0.128 g) according to the general procedure above; (30%), Mp. 200–205 °C. ^1^H NMR (400 MHz, CDCl_3_) δ 3.81 (dd, *J* = 8.92, 3.11 Hz, 1H) 3.87 (s, 3H) 4.27 (d, *J* = 9.12 Hz, 1H) 4.96 (d, *J* = 3.32 Hz, 1H) 7.10–7.16 (m, 2H) 7.20 (d, *J* = 3.73 Hz, 2H) 7.21 -7.29 (m, 4H) 7.29–7.33 (m, 1H) 7.42 (dd, *J* = 4.98, 3.73 Hz, 1H) 7.56 (d, *J* = 6.63 Hz, 1H) 7.73–7.91 (m, 1H) 8.15–8.20 (m, 2H)^13^C NMR (101 MHz, CDCl_3_) ppm 40.15–40.69, 44.50, 49.19–50.26, 51.26, 51.57, 55.08, 57.04–58.17, 114.72, 122.69, 127.03, 130.49, 131.59, 138.97, 140.11, 141.27, 142.39, 170.58, 171.44, 175.90, 185.10, 187.87 (chalcone C=O). IR *ν* max (ATR): 1670.35 (C=O), 1600.48 (Ar C=C), 1229.19 (C-O) cm^−1^. HRMS (APCI): calculated for C_28_H_19_O_5_ [M^+^−H]: 435.1238; found: 435.1241.

(*E*)-9-(3-Oxo-3-(*p*-tolyl)prop-1-en-1-yl)-9,10-dihydro-9,10-[3,4] furanoanthracene-12,14-dione (**22j**) was synthesized from (*E*)-3-(anthracen-9-yl)-1-(*p*-tolyl)prop-2-en-1-one (1.0 mmol, 0.3384 g) and maleic anhydride (1.3 mmol, 0.13 g) according to the general procedure above; white powder (73%), Mp. 223–227 °C [51]. ^1^H NMR (400 MHz, CDCl_3_) δ 2.41 (s, 3H) 3.81 (dd, *J* = 8.92, 3.11 Hz, 1H) 4.27 (d, *J* = 9.12 Hz, 1H) 4.96 (d, *J* = 2.90 Hz, 1H) 7.20 (d, *J* = 3.73 Hz, 2H) 7.21–7.29 (m, 3H) 7.29–7.34 (m, H) 7.42 (d, *J* = 7.88 Hz, 3H) 7.56 (d, *J* = 6.63 Hz, 1H) 7.82 (q, *J* = 16.17 Hz, 2H) 8.08 (d, *J* = 7.88 Hz, 2H). ^13^C NMR (101 MHz, CDCl_3_) ppm 21.70, 44.81, 49.00–50.00, 123.38, 124.98, 127.04, 127.49, 127.85, 129.30, 129.99, 132.05, 135.15, 138.96, 140.06, 140.69, 142.33, 144.52, 170.59, 171.43, 189.16 (chalcone C=O). IR *ν* max (ATR): 1769.23 (C=O), 1603.35 (Ar C=C), 1458.64 (C-CH_3_), 1228.91 (C-O) cm^−1^. LRMS (APCI): 419.30 [M^+^+H]. HRMS (APCI): calculated for C_29_H_24_NaO_5_ [M^+^+Na]: 475.1516; found 475.1512.

(*E*)-9-(3-Oxo-3-(3,4,5-trimethoxyphenyl)-prop-1-en-1-yl)-9,10-dihydro-9,10-[3,4]furanoanthracene-12,14-dione (**22k**) was synthesized from (*E*)-3-(anthracene-9-yl)-1-(4-chorophenyl)prop-2-en-1-one (1.0 mmol, 0.398 g) and maleic anhydride (1.3 mmol, 0.13 g) according to the general procedure above; white powder (78%), Mp. 262–266 °C. ^1^H NMR (400 MHz, CDCl_3_) δ 3.77 (s, 3H), 3.80 (dd, *J* = 9.12, 3.32 Hz, 1H), 3.87 (s, 6H) 4.27 (d, *J* = 9.12 Hz, 1H), 4.96 (d, *J* = 3.32 Hz, 1H) 7.20–7.23 (m, 2H) 7.23–7.28 (m, 3H) 7.32–7.35 (m, 1H) 7.40–7.43 (m, 1H) 7.48 (s, 2H) 7.56 (d, *J* = 6.22 Hz, 1H) 7.72–7.91 (m, 2 H). ^13^C NMR (101 MHz, CDCl_3_) ppm 43.55, 44.81, 49.23–49.97, 56.55, 60.70, 106.84, 123.39, 123.96, 125.00, 125.78, 127.50, 132.58, 132.95, 138.99, 139.83–140.53, 153.45, 171.40, 188.92 (chalcone C=O). IR *ν* max (ATR): 1662.19 (C=O), 1571.62 (Ar C=C), 1231.78 (C-O) cm^−1^. HRMS (APCI): calculated C_30_H_25_O_7_ [M^+^+H]: 495.1449; found: 495.1449.

(*E*)-9-(3-(4-Chlorophenyl)-3-oxoprop-1-en-1-yl)-9,10-dihydro-9,10[3,4]furanoanthracene-12,14-dione (**22l**) was synthesized from (*E*)-3-(anthracen-9-yl)-1-(4-chlorophenyl)prop-2-en-1-one (1.0 mmol, 0.3428 g) and maleic anhydride (1.3 mmol, 0.13 g) according to the general procedure above; white powder (89%), Mp. 248–252 °C. ^1^H NMR (400 MHz, CDCl_3_) δ 3.81 (dd, *J* = 8.71, 3.32 Hz, 1H), 4.27 (d, *J* = 9.12 Hz, 1H), 4.96 (d, *J* = 3.32 Hz, H), 7.18–7.29 (m, 5H), 7.30–7.35 (m, 1H), 7.42 (dd, *J* = 5.60, 3.11 Hz, 1H), 7.56 (d, *J* = 7.05 Hz, 1H), 7.67–7.72 (m, 2H), 7.79–7.90 (m, 2H), 8.16–8.21 (m, 2H). ^13^C NMR (101 MHz, CDCl_3_) ppm 39.13–40.72, 44.90, 125.51, 128.81, 129.41–129.54, 130.18, 131.84, 134.30, 135.66, 142.34, 143.69, 148.97, 149.27, 150.80, 171.10, 191.34 (chalcone C=O). IR *ν* max (ATR): 1773.83 (C=O), 1666.32 (C=C), 1618.07 (Ar C=C), 1221.94 (C-O), 712.12 (C-Cl) cm^−1^ HRMS (APCI): calculated for C_27_H_17_ClO_4_ [M^+^+H]: 439.0743; found: 439.0739.

9-(*E*)-3-Oxo-3-(pyridin-4-yl)prop-1-en-1-yl)-9,10-dihydro-9,10-[3,4]furanoanthracene-12,14-dione (**22m**) was prepared from (*E*)-3-(anthracen-9-yl)-1-(pyridin-4-yl)prop-2-en-1-one (1.0 mmol, 0.309 g) and maleic anhydride (1.3 mmol, 0.13 g) according to the general procedure above; light brown solid (99%), Mp. >200 °C. ^1^H NMR (400 MHz, CDCl_3_) δ 3.65 (dd, *J* = 9.12, 2.90 Hz, 1H, CH), 3.80 (d, *J* = 9.12 Hz, 1H, CH), 4.87 (d, *J* = 2.49 Hz, 1H, CH), 7.10–7.23 (m, 3H, ArH), 7.25–7.34 (m, 3H, ArH), 7.36–7.46 (m, 2H, ArH), 7.70 (d, *J* = 16.17 Hz, 1H, CH=C), 7.94 (d, *J* = 4.98 Hz, 2 H, ArH), 8.01 (d, *J* = 16.17 Hz, CH=C). ^13^C NMR (101 MHz, CDCl_3_) ppm 45.44, 48.99, 49.24, 51.99, 123.42, 123.71, 124.33, 125.70, 127.18, 127.77, 128.02, 128.28, 130.41, 140.20, 143.49, 150.40, 168.91, 169.60, 188.91 (chalcone C=O). IR *ν* max (KBr): 3064.92, 2969.14 (Ar C-H), 1773.15 (C=O), 1638.35, 1677.99 (C=C), 1625.35, 1585.28, 1458.89 (Ar C=C), 1069.92 (C-N) cm^−1^. HRMS (APCI) calculated for C_26_H_18_NO_4_ [M^+^+H]: 408.1230 found: 408.1231.

9-(*E*)-3-Oxo-3-(pyridin-2-yl)prop-1-en-1-yl)-9,10-dihydro-9,10-[3,4]furanoanthracene-12,14-dione (**22n**) prepared from (*E*)-3-(anthracen-9-yl)-1-(pyridin-2-yl)prop-2-en-1-one (1.0 mmol, 0.3094 g) and maleic anhydride (1.3 mmol, 0.13 g) according to the general procedure above; white powder (100%), Mp. 225–227 °C. ^1^H NMR (400 MHz, CDCl_3_) δ 3.62 (dd, *J* = 9.12, 3.32 Hz, 1H, CH), 3.93 (d, *J* = 9.54 Hz, 1H, CH), 4.85 (d, *J* = 3.32 Hz, 1H, CH), 7.13–7.24 (m, 3H, ArH), 7.25–7.31 (m, 3 H,ArH), 7.36–7.45 (m, 3 H ArH), 7.51 (ddd, *J* = 7.57, 4.67, 1.04 Hz, 1 ArH), 7.93 (td, *J* = 7.67, 1.66 Hz, 1H, ArH), 8.13 (d, *J* = 16.59 Hz, 1 H, CH=C), 8.29 (dt, *J* = 7.88, 1.04 Hz, 1H, ArH), 8.42 (d, *J* = 16.59 Hz, 1H, CH=C), 8.70–8.73 (m, 1H, ArH). IR *ν* max (ATR): 3087.05, 3064.46, 2968.93 (Ar C-H), 1770.84 (C=O), 1676.99 (C=C), 1625.37, 1584.71, 1479.76, 1456.99 (Ar C=C), 1325.60 (C-N), 1084.30 (C-O) cm^−1^. HRMS (APCI): calculated for C_26_H_18_NO_4_: 408.1230 [M^+^+H]; found: 408.1237.

9-(*E*)-3-(Furan-2-yl)-3-oxoprop-1-en-1-yl)-9,10-dihydro-9,10-[3,4]furanoanthracene-12,14-dione (**22o**) was prepared from (*E*)-3-(anthracen-9-yl)-1-(furan-2-yl)prop-2-en-1-one (1.0 mmol, 0.298 g) and maleic anhydride (1.3 mmol, 0.13 g) according to the general procedure above; cream powder (81%), Mp. 222–225 °C. ^1^H NMR (400 MHz, CDCl_3_) δ 3.63 (dd, *J* = 9.54, 3.32 Hz, 1H, CH), 3.82 (d, *J* = 9.12 Hz, 1H, CH), 4.85 (d, *J* = 3.32 Hz, 1H, CH), 6.63 (dd, *J* = 3.73, 1.66 Hz, 1H, CH=C), 7.15–7.29, 5H, ArH, CH=C), 7.33–7.40 (m, 2H, ArH), 7.40–7.46 (m, 2H, ArH), 7.63 (d, *J* = 16.59 Hz, 1H, CH=C), 7.69 (d, *J* = 1.24 Hz, 1H, CH=C), 8.02 (d, *J* = 16.17 Hz, 1H, CH=C). ^13^C NMR (101 MHz, CDCl_3_) ppm 45.50, 49.08, 49.26, 51.82, 112.78, 118.91, 123.63, 123.95, 124.15, 125.53, 127.05, 127.56, 127.91, 128.04, 130.36, 137.38, 138.96, 140.29, 140.61, 141.55, 147.26, 153.19, 168.65, 169.93, 177.01 (chalcone C=O). IR *ν* max (ATR): 3071.48, 2882.79 (Ar C-H), 1776.90, 1668.01 (C=O), 1580.26, 1405.03 (Ar C=C), 1226.47, 1071.29 (C-O) cm^−1^. HRMS (APCI): calculated for C_25_H_17_O_5_: 397.1071 [M^+^+H]; found: 397.1074.

9-(*E*)-3-(Naphthalen-2-yl)-3-oxoprop-1-en-1-yl)-9,10-dihydro-9,10-[3,4]furanoanthracene-12,14-dione (**22p**) was prepared from (*E*)-3-(anthracen-9-yl)-1-(naphthalen-2-yl)prop-2-en-1-one (1.0 mmol, 0.3584 g) and maleic anhydride (1.3 mmol, 0.13 g) according to the general procedure above; green crystals (86%), Mp. 226–231 °C. ^1^H NMR (400 MHz, CDCl_3_) δ 3.66 (dd, *J* = 9.12, 3.32 Hz, 1H), 3.86 (d, *J* = 9.54 Hz, 1 H), 4.88 (d, *J* = 3.32 Hz, 1H), 7.17–7.31 (m, 4H), 7.34–7.46 (m, 4H), 7.54–7.65 (m, 2H), 7.88–7.94 (m, 2H), 7.96–8.03 (m, 3H), 8.22 (dd, *J* = 8.71, 1.66 Hz, 1H), 8.71 (s, 1 H). ^13^C NMR (101 MHz, CDCl_3_) ppm 55.53–56.38, 60.88, 69.77–71.12, 106.07, 106.66, 107.75, 110.78–111.13, 114.44, 114.76, 123.43, 126.97, 127.76, 128.45. IR *ν* max (ATR): 3064.36, 2968.90 (Ar C-H), 1771.08 (C=O), 1676.62, 1625.39 (C=C), 1584.75, 1458.10 (Ar C=C),1 325.74 (C-O) cm^−1^. HRMS (APCI): calculated for C_31_H_21_O_4_ [M^+^+H]: 457.1434; found: 457.1438.

9-(*E*)-3-Oxo-3-(thiophen-2-yl)prop-1-en-1-yl)-9,10-dihydro-9,10-[3,4]furanoanthracene-12,14-dione (**22q**) was prepared from (*E*)-3-(anthracen-9-yl)-1-(thiophen-2-yl)prop-2-en-1-one (1.0 mmol, 0.3144 g) and maleic anhydride (1.3 mmol, 0.13 g) according to the general procedure above; cream powder (76%), Mp. 214–218 °C. ^1^H NMR (400 MHz, CDCl_3_) δ 3.64 (dd, *J* = 9.12, 2.90 Hz, 1H, CH), 3.81 (d, *J* = 9.12 Hz, 1H, CH), 4.86 (d, *J* = 2.90 Hz, 1H, CH), 7.11–7.28 (m, 5 H, ArH, CH=C), 7.28–7.46 (m, 4H, ArH), 7.66 (d, *J* = 16.17 Hz, 1H, CH=C), 7.75 (d, *J* = 4.98 Hz, 1H, CH=C), 7.91–8.01 (m, 2H, CH=C). ^13^C NMR (101 MHz, CDCl_3_) ppm 45.40, 48.99, 49.63, 52.01, 56.07, 110.20, 111.07, 123.48, 123.70, 123.93, 125.41, 126.70, 127.24, 127.41, 130.88, 131.48, 139.42, 140.66–140.97, 142.44, 149.24, 153.52, 174.93, 175.64, 188.72 (chalcone C=O). IR *ν* max (ATR): 3089.66, 2882.60 (Ar C-H), 1777.72, 1664.07 (C=O) 1616.88 (C=C), 1579.42, 1512.85, 1465.10, 1456.62, 1418.52 (Ar C=C), 1213.16 (C-O) cm-^1^. LRMS (APCI): 411.22 [M^+^−H]. HRMS (APCI): calculated for C_25_H_16_NaO_4_S [M^+^+Na]: 435.0662; found: 435.0658.

### 3.4. General Preparation for (E)-9-(3-oxo-3-phenylprop-1-en-yl)-9,10-dihydro-9,10-[3,4]epipyrroloanthracene-12,14-diones ***23a-q***

To a solution of the appropriate chalcone anthracene analog **21a–21q** (1.0 mmol) in toluene (2 mL) was added dienophile maleimide (1.3 mmol). The mixture was heated and stirred at 90 °C for 48 h. The reaction mixture was then cooled to room temperature, and the resulting product was isolated via filtration and washed with toluene (2 mL) and diethyl ether (2 mL). The product was then recrystallized from toluene.

9-(*E*)-3-(4-Bromophenyl)-3-oxoprop-1-en-1-yl)-9,10-dihydro-9,10-[3,4]epipyrroloanthracene-12,14-dione (**23a**) was prepared from (*E*)-3-(anthracen-9-yl)-1-(4-bromophenyl)prop-2-en-1-one (1.0 mmol, 0.387 g) and maleimide (1.3 mmol, 0.13g) according to the general procedure above; light yellow powder (91%), Mp. 295–297 °C. (HPLC: 87.8%, RT 5.02 min). ^1^H NMR (400 MHz, DMSO-*d_6_*) δ 3.32 (m, 1H, CH), 3.82 (d, *J* = 8.82 Hz, 1H, CH), 4.81 (d, *J* = 3.32 Hz, 1H, CH), 7.11–7.22 (m, 5 H, ArH, CH=C), 7.23–7.29 (m, 1H, ArH), 7.30–7.35 (m, 1H, ArH), 7.51 (d, *J* = 7.05 Hz, 1 H, ArH), 7.78–7.85 (m, 4H), 8.06–8.12 (m, 2H), 10.87 (s, 1H). ^13^C NMR (101 MHz, CDCl_3_) ppm 44.96, 49.09, 49.30, 51.70, 123.24, 123.66, 124.61, 126.65, 127.16, 127.28, 131.14, 131.47, 132.47, 136.76, 139.29, 140.24, 142.15, 143.10, 143.11, 177.48, 177.74, 189.08 (chalcone C=O). IR *ν* max (ATR): 3341 (N-H), 3096.65 (C-H), 1720 (C=O), 1629.57 (Ar C=C), 1603.21, 1522.86, 1458.09, (C-N) 1311, 1165, (C-Br) 1003.06 cm^−1^. HRMS (EI) calculated for C_27_H_17_BrNO_3_ [M^+^−H]: 482.0397; found 482.0391.

9-*(E*)-3-(4-Nitrophenyl)-3-oxoprop-1-en-1-yl)-9,10-dihydro-9,10-[3,4]epipyrroloanthracene-12,14-dione (**23b**) was prepared from (*E*)-3-(anthracen-9-yl)-1-(4-nitrophenyl)prop-2-en-1-one (1.0 mmol, 0.353 g) and maleimide (1.3 mmol, 0.13 g) according to the general procedure above; beige powder (87%), Mp. >300 °C (dec). ^1^H NMR (400 MHz, DMSO-*d*6) δ 3.35 (d, *J* = 3.32 Hz, 1H, CH), 3.82 (d, *J* = 8.71 Hz, 1H, CH), 4.82 (d, *J* = 2.90 Hz, 1H, CH), 7.15–7.23 (m, 4H, ArH), 7.28–7.33 (m, 1 H, ArH), 7.34–7.39 (m, 1H, ArH), 7.52–7.57 (m, 1H, ArH), 7.82–7.89 (d, *J* = 15.8 Hz, 1 H, CH=C), 7.89–8.01 (d, *J* = 16.2 Hz, 1H, CH=C,), 8.37–8.47 (m, 4H, ArH), 10.88 (s, 1H). ^13^C NMR (101 MHz, DMSO-*d_6_*) 20.05, 24.86, 44.97, 49.24, 51.78, 63.44, 73.92, 78.91, 88.39, 109.61, 111.59, 118.64, 123.30, 123.71, 124.64, 125.65, 126.66, 127.25, 130.51, 131.69, 139.28, 140.12, 142.13, 142.52, 142.96, 144.40, 150.46,177.52, 177.70, 189.16 (chalcone C=O) ppm. IR *ν* max (ATR): 3343 (N-H), 3058.45 (C-H), 1719 (C=O), 1677.02, 1638.82 (C=C), 1599.84, 1458.22 (Ar C=C), 1517, 1340 (NO_2_), 1317, 1164 (C-N) cm-^1^. HRMS (EI): calculated for C_27_H_17_N_2_O_3_ [M^+^−H]: 449.1143; found: 449.1146.

9-((*E*)-3-(4-Ethylphenyl)-3-oxoprop-1-en-1-yl)-9,10-dihydro-9,10-[3,4]epipyrroloanthracene-12,14-dione (**23c**) was prepared from (*E*)-3-(anthracen-9-yl)-1-(4-ethylphenyl)prop-2-en-1-one (1.0 mmol, 0.336 g) and maleimide (1.3 mmol, 0.13 g) according to the general procedure above; white powder (82%), Mp. 265–269 °C. ^1^H NMR (400 MHz, DMSO-*d_6_*) δ 1.24 (t, *J* = 7.58 Hz, 3H), 2.73 (q, *J* = 7.83 Hz, 2H), 3.32 (d, *J* = 3.42 Hz, 1H), 3.82 (d, *J* = 8.31 Hz, 1H), 4.82 (d, *J* = 3.42 Hz, 1H), 7.11–7.26 (m, 5H), 7.27–7.32 (m, 6H), 7.34 (d, *J* = 6.85 Hz, 1H), 7.80–7.84 (m, 1H), 7.80–7.84 (m, 1H), 7.87 (d, *J* = 16.14 Hz, 1H), 8.13 (d, *J* = 8.31 Hz, 2H), 10.87 (br. s., 1H). IR *ν* max (ATR): (N-H) 3343 (C-H) 3088.25, (*trans* C=C), 1776.11, 1720 (C=O), 1626.04, 1603.36 (Ar C=C), 1522.96, 1457.88, (C-N) 1313, 1165 cm^−1^. HRMS (EI) calculated for C_29_H_23_NNaO_3_ [M^+^+Na]: 456.1570; found: 456.1562.

9-(*E*)-3-(2,4-Dichlorophenyl)-3-oxoprop-1-en-1-yl)-9,10-dihydro-9,10-[3,4]epipyrroloanthracene-12,14-dione (**23d**) was prepared from (*E*)-3-(anthracen-9-yl)-1-(2,4-dichlorophenyl)prop-2-en-1-one (1.0 mmol, 0.377 g) and maleimide (1.3 mmol, 0.13 g) according to the general procedure above; beige powder (82%), Mp. 265–268 °C. ^1^H NMR (400 MHz, DMSO-*d_6_*) δ 3.24 (dd, *J* = 8.6, 3.2 Hz, 1H), 3.61 (d, *J* = 8.6 Hz, 1H), 4.76 (d, *J* = 3.2 Hz, 1H), 7.23–7.10 (m, 7H), 7.31 (dq, *J* = 4.7, 2.6 Hz, 1H), 7.49 (dd, *J* = 6.6, 1.8 Hz, 1H), 7.67–7.53 (m, 2H), 7.86–7.79 (m, 2H), 10.81 (s, 1H) ^13^C NMR (101 MHz, CDCl_3_) ppm 44.92, 49.24, 51.71, 123.17, 123.47, 124.73, 125.68, 126.63, 127.12, 127.26, 127.38, 128.23, 130.31, 131.41, 131.87, 135.33, 136.45, 137.33, 139.23, 139.82, 142.09, 142.59, 146.63, 177.28, 177.68, 192.42 (chalcone C=O) IR *ν* max (ATR): 3336 (N-H), 3062.21 (C-H), 1778.31, 1720 (C=O), 1601.84 (Ar C=C), 1578.57, 1521.96, 1457.78, (C-N) 1315, 1171 cm^−1^. HRMS (ESI): calculated for C_27_H_16_Cl_2_NO_3_ [M^+^−H]: 472.0513; found: 472.0519.

9-(E)-3-(3,4-Dimethoxyphenyl)-3-oxoprop-1-en-1-yl)-9,10-dihydro-9,10-[3,4]epipyrroloanthracene-12,14-dione (**23e**) was prepared from (*E*)-3-(anthracen-9-yl)-1-(3,4-dimethoxyphenyl)prop-2-en-1-one (1.0 mmol, 0.368 g) and maleimide (1.3 mmol, 0.13 g) according to the general procedure above, cream/yellow powder (83%), Mp. 269–271 °C. ^1^H NMR (400 MHz, CDCl_3_) δ 3.37 (dd, *J* = 8.5, 3.2 Hz, 1H), 3.56 (d, *J* = 8.6 Hz, 1H), 3.98 (d, *J* = 9.3 Hz, 6H), 4.81 (d, *J* = 3.2 Hz, 1H), 6.97 (d, *J* = 8.4 Hz, 1H), 7.14–7.24 (m, 4H), 7.35 (ddd, *J* = 10.9, 6.8, 2.5 Hz, 3H), 7.38–7.45 (m, 1H), 7.47 (s, 1H), 7.75–7.84 (m, 2H), 7.85 (dd, *J* = 8.4, 2.0 Hz, 1H), 7.91 (d, *J* = 16.2 Hz, 1H). ^13^C NMR (101 MHz, CDCl_3_) ppm 45.41, 48.99, 49.63, 52.00, 56.05, 56.09, 110.20, 111.07, 123.48, 123.70, 123.90, 123.95, 125.41, 126.70, 127.20, 127.27, 127.41, 130.88, 131.48, 137.96, 139.42, 140.87, 142.44, 149.24, 153.52, 174.95, 175.67, 188.72 (chalcone C=O). IR *ν* max (ATR): 3342 (N-H), 3061.45 (C-H), 1774.72, 1719 (C=O), 1626.19 (Ar C=C), 1598.13, 1517.42, 1458.15, (C-N) 1317, 1169, (C-O) 1268.92, 1017.26 cm^−1^. HRMS (ESI): calculated for C_29_H_22_NO_5_ [M^+^−H] 464.1504; found: 464.1504.

(*E*)-9-(3-oxo-3-phenylprop-1-en-yl)-9,10-dihydro-9,10-[3,4]epipyrroloanthracene-12,14-dione (**23f**) was synthesized from (*E*)-3-(anthracen-9-yl)-1-phenylprop-2-en-1-one (1.0 mmol, 0.308 g) and maleimide (1.3 mmol, 0.13 g) according to the general procedure above; (61%), Mp. 218–222 °C. ^1^H NMR (400 MHz, DMSO-*d_6_*) δ 3.80 (d, *J* = 8.71 Hz, 1H) 4.79 (d, *J* = 3.32 Hz, 1H) 7.10–7.24 (m, 6H) 7.24–7.29 (m, 1H) 7.32–7.37 (m, 1H) 7.52 (d, *J* = 6.63 Hz, 1H) 7.57–7.65 (m, 2H) 7.67 -7.74 (m, 1H) 7.84 (dd, *J* = 16.60 Hz, 2H) 8.12–8.21 (m, 2H) 10.84 (s, 1H). ^13^C NMR (101 MHz, DMSO-*d_6_*) 123.23, 123.64, 124.62, 125.66, 125.76, 126.67 127.24, 128.64, 129.15, 129.30–129.46 131.65–131.81, 133.89, 137.78, 139.32, 140.34, 142.55, 143.20, 177.31–177.97, 189.92 (chalcone C=O) ppm. IR *ν* max (ATR): 3347.06 (N-H), 1720.18 (C=O), 1674.84, 1628.01 (C=C), 1000.09 (CN) cm^−1^. HRMS (APCI): calculated for C_27_H_20_NO_3_ [M^+^+H]: 406.1438; found: 406.1450.

9-(*E*)-3-(4-Iodophenyl)-3-oxoprop-1-en-1-yl)-9,10-dihydro-9,10-[3,4]epipyrroloanthracene-12,14-dione (**23g**) was prepared from (*E*)-3-(anthracene-9-yl)-1-(4-iodophenyl)prop-2-en-1-one (1.0 mmol, 0.449 g) and maleimide (1.3 mmol, 0.13 g) according to the general procedure above; yellow powder (93%), Mp. 288–293 °C. (HPLC: 98.22, RT 6.72 min). ^1^H NMR (400 MHz, DMSO-*d_6_*) δ 3.79 (d, J = 8.6 Hz, 1H), 3.31–3.29 (m,1H). 4.80 (d, J = 3.2 Hz, 1H), 7.29–7.24 (m,1H), 7.23–7.13 (m, 5H), 7.37–7.31 (m, 1H), 7.52 (dt, J = 7.0, 1.1 Hz, 1H), 7.82 (d, J = 6.7 Hz, 2H), 7.95–7.91 (m, 2H), 8.03–7.98 (m, 2H), 10.85 (s, 1H). ^13^C NMR (101 MHz, DMSO-*d_6_*) 44.98, 49.13, 49.32, 51.72, 102.63, 123.26, 123.67, 124.62, 125.65, 126.66, 127.17, 127.29, 130.84, 131.45, 137.06, 138.35, 139.30, 140.27, 142.17, 143.05, 143.12, 177.48, 177.74, 189.40. ppm IR *ν* max (ATR): 3339 (N-H), 2987.83 (C-H),1688.48 (C=O) 1633, 1621.45 (Ar C=C), 1557.57, 1485.56, 687.18 cm^−1^. HRMS (APCI): calculated C_27_H_19_INO_3_ [M^+^+H]: 532.0404, found 532.0402.

9-(*E*)-3-(4-Fluorophenyl)-3-oxoprop-1-en-1-yl)-9,10-dihydro-9,10-[3,4]epipyrroloanthracene-12,14-dione (**23h**) was prepared from (*E*)-3-(anthracen-9-yl)-1-(4-fluorophenyl)prop-2-en-1-one (1.0 mmol, 0.326 g) and maleimide (1.3 mmol, 0.13 g) according to the general procedure above; cream powder (100%), Mp. 260–268 °C. ^1^H NMR (400 MHz, CDCl_3_) δ 3.36 (dd, *J* = 8.5, 3.2 Hz, 1H), 3.54 (d, *J* = 8.5 Hz, 1H), 4.80 (d, *J* = 3.2 Hz, 1H), 7.12–7.26 (m, 6H), 7.32 (m, 3H), 7.37–7.44 (m, 1H), 7.49 (s, 1H), 7.76 (d, *J* = 16.2 Hz, 1H), 7.94 (d, *J* = 16.2 Hz, 1H), 8.16–8.26 (m, 2H). IR *ν* max (ATR): (N-H) 3340 (C-H) 2984.37, 1776.16, (C=O) 1718, 1630.99 (Ar C=C), 1595.20, 1457.80, (C-N) 1311, 1157, (C-F) 1224.84 cm^−1^. HRMS (APCI): calculated for C_27_H_19_FNO_3_ [M^+^+H]: 412.1002; found: 412.1004.

(*E*)-9-(3-(4-Methoxyphenyl)-3-oxoprop-1-en-1-yl)-9,10-dihydro-9,10-[3,4]epipyrroloanthracene-12,14-dione (**23i**) was synthesized from (*E*)-3-(anthracen-9-yl)-1-(4-methoxyphenyl)prop-2-en-1-one (1.0 mmol, 0.338g) and maleimide (1.3 mmol, 0.13 g) according to the general procedure above; white solid (70%), Mp. 281–284 °C. ^1^H NMR (400 MHz, CDCl_3_) δ 3.80 (d, *J* = 8.29 Hz, 1H), 3.87 (s, 3H), 4.79 (d, *J* = 2.90 Hz, 1H), 7.10–7.19 (m, 5H), 7.19–7.23 (m, 3H), 7.25 (app t, *J* = 5.39 Hz, 2H), 7.32–7.36 (m, 1H), 7.52 (d, *J* = 6.63 Hz, 1H), 7.74–7.88 (m, 2H), 8.17 (d, *J* = 9.12 Hz, 2H). ^13^C NMR (101 MHz, CDCl_3_) ppm 39.15–40.72, 44.97, 49.37, 56.07, 123.21, 124.60, 125.65, 127.01–127.38, 130.62, 131.37–132.01, 140.43, 141.47, 143.30, 177.79, 188.12 (C=O chalcone). IR *ν* max (ATR): 3343.73 (N-H), 1670.36 (C=O), 1601.24 (Ar C=C), 1230.33 (C-O), 1019.60 (C-N) cm-^1^. LRMS (APCI): 434.30 [M^+^−H]. HRMS (APCI): calculated C_28_H_20_NO_4_ [M^+^−H]: 434.1398; found: 434.1392.

(*E*)-9-(3-Oxo-3-(*p*-tolyl)prop-1-en-1-yl)-9,10-dihydro-9,10-[3,4]epipyrroloanthracene-12,14-dione (**23j**) was synthesized from (*E*)-3-(anthracen-9-yl)-1-(*p*-tolyl)prop-2-en-1-one (1.0 mmol, 0.338 g) and maleimide (1.3 mmol, 0.13 g) according to the general procedure above; white powder (46%), Mp. 129–134 °C. ^1^H NMR (400 MHz, CDCl_3_) δ 2.41 (s, 3H) 3.80 (d, *J* = 8.71 Hz, 1H) 4.79 (br. s., 1H) 7.10–7.30 (m, 7H) 7.30–7.45 (m, 3H) 7.52 (d, *J* = 6.63 Hz, 1H) 7.75–7.89 (m, 2H) 8.03–8.04 (m, 1H) 8.04–8.13 (m, 2H)^13^C NMR (101 MHz, CDCl_3_) ppm 21.69, 39.07–40.73, 44.99, 48.86–49.53, 55.35, 123.23, 123.64, 126.93–127.51, 129.04–129.58, 129.96, 135.30, 140.39, 141.79–142.33, 144.37, 177.47, 189.36 (chalcone C=O)IR *ν* max (ATR): 1710.75 (C=O), 1625.28 (Ar C=C), 1458.68 (C-CH_3_), 1231.30 (C-O), 1072.28 (C-N) cm^−1^. HRMS (APCI): calculated C_28_H_22_NO_3_ [M^+^−H]: 420.1594; found: 420.1606.

(*E*)-9-(3-Oxo-3-(3,4,5-trimethoxyphenyl)prop-1-en-1-yl)-9,10-dihydro-9,10-[3,4] epipyrroloanthracene-12,14-dione (**23k**) was synthesized from (*E*)-3-(anthracen-9-yl)-1-(3,4,5-trimethoxyphenyl)prop-2-en-1-one (1.0 mmol, 0.398 g) and maleimide (1.3 mmol, 0.13 g) according to the general procedure above; white powder (53%), Mp. 257–260 °C. ^1^H NMR (400 MHz, CDCl_3_) δ 3.30 (d, *J* = 2.90 Hz, 1H), 3.76–3.79 (m, 3H), 3.81 (s, 1H), 3.87 (s, 6H), 4.79 (d, *J* = 3.32 Hz, 1H), 7.16–7.25 (m, 5H), 7.28 (d, *J* = 4.98 Hz, 1H), 7.32 -7.36 (m, 1H), 7.47–7.54 (m, 3H), 7.72–7.87 (m, 2H), 10.85 (s, 1H). ^13^C NMR (101 MHz, CDCl_3_) ppm 41.66, 43.00, 46.27, 49.08–49.42, 51.67, 56.51, 65.87, 106.82, 123.23, 123.67, 124.66, 125.66, 126.63,127.39, 132.39, 133.09, 139.33, 141.76, 142.56, 143.27,177.96, 189.32 (C=O chalcone). IR *ν* max (ATR): 1670.40 (C=O), 1600.25 (Ar C=C), 1229.98 (C-O), 1017.32 (C-N) cm^−1^. HRMS (APCI): calculated for C_30_H_24_NO_6_ [M^+^−H]: 494.1609; found: 494.1605.

(*E*)-9-(3-(4-Chlorophenyl)-3-oxoprop-1-en-1-yl)-9,10-dihydro-9,10-[3,4]epipyrroloanthracene-12,14-dione (**23l**) was synthesized from (*E*)-3-(anthracen-9-yl)-1-(4-chlorophenyl)prop-2-en-1-one (1.0 mmol, 0.343 g) and maleimide (1.3 mmol, 0.13 g) according to the general procedure above; white solid (65%), Mp. 280–285 °C. (HPLC: 97.72%, RT 7.23 min). ^1^H NMR (400 MHz, CDCl_3_) δ 3.80 (d, *J* = 8.71 Hz, 1H) 4.80 (d, *J* = 2.90 Hz, 1H) 7.09–7.39 (m, 8H) 7.52 (d, *J* = 7.05 Hz, 1H) 7.68 (d, *J* = 8.29 Hz, H) 7.85 (s, 2 H) 8.19 (d, *J* = 8.29Hz, 2H) 10.85 (s, 1H). ^13^C NMR (101 MHz, CDCl_3_) ppm 44.98, 49.11, 49.32, 51.71, 123.26, 123.67,124.13, 124.62, 125.65, 125.75, 126.66, 127.18, 127.29, 128.64, 129.34, 129.54, 136.45, 140.28, 142.17, 143.11, 177.12–178.10, 198.62 (chalcone C=O). IR *ν* max (ATR): 1717.64 (C=O), 754.55 (C-Cl) cm^−1^. LRMS (APCI): 438.2284 [M^+^−H]. HRMS (APCI): calculated for C_27_H_18_ClNNaO_3_ [M^+^+Na]: 462.0867; found 462.0864.

9-(*E*)-3-Oxo-3-(pyridin-4-yl)prop-1-en-1-yl)-9,10-dihydro-9,10-[3,4]epipyrroloanthracene-12,14-dione (**23m**) was prepared from (*E*)-3-(anthracen-9-yl)-1-(pyridin-4-yl)prop-2-en-1-one (1.0 mmol, 0.3094 g) and maleimide (1.3 mmol, 0.13g) according to the general procedure above; powder (100%), Mp. 256–258 °C. ^1^H NMR (400 MHz, CDCl_3_) δ 3.36 (dd, *J* = 8.29, 3.32 Hz, 1H, CH), 3.54 (d, *J* = 8.29 Hz, 1H, CH), 4.81 (d, *J* = 3.32 Hz, 1H, CH), 7.12–7.25 (m, 6H, ArH), 7.26–7.31 (m, 1 H, ArH), 7.33–7.38 (m, 1H, ArH), 7.39–7.43 (m, 1H, ArH), 7.73 (d, *J* = 16.17 Hz, 1 H, CH=C), 7.95 (d, *J* = 5.39 Hz, 2H, ArH), 8.01 (d, *J* = 16.17 Hz, 1H, CH=C), 8.86 (br.s., 1 H, NH). ^13^C NMR (101 MHz, CDCl_3_) ppm 45.37, 48.96, 49.54, 52.07, 121.87, 123.29, 123.57, 124.14, 125.55, 126.76, 127.35, 127.60, 130.63, 1 (N-H), 3010.28 (C-H), 1776.21, 1721 (C=O), 1634.91 (Ar C=C), 1457.77, 1407.25, (C-N) 1317, 1166 cm^−1^. HRMS (ESI): calculated C_26_H_17_N_2_O_3_ [M^+^−H] 405.1245; found: 405.1247.

9-(*E*)-3-Oxo-3-(pyridin-2-yl)prop-1-en-1-yl)-9,10-dihydro-9,10-[3,4]epipyrroloanthracene-12,14-dione (**23n**) was prepared from (*E*)-3-(anthracen-9-yl)-1-(pyridin-2-yl)prop-2-en-1-one (1.0 mmol, 0.309 g) and maleimide (1.3 mmol, 0.13 g) according to the general procedure above; brown powder (72%), Mp. > 200 °C. (HPLC: 92.20%, RT 3.80 min). ^1^H NMR (400 MHz, DMSO-*d_6_*) δ 3.33 (dd, *J* = 8.50, 3.11 Hz, 1H, CH), 3.64 (d, *J* = 8.29 Hz, 1H, CH), 4.78 (d, *J* = 3.32 Hz, 1H, CH), 7.10–7.22 (m, 4H, ArH), 7.29 (d, *J* = 7.46 Hz, 1H, ArH), 7.31–7.35 (m, 1H, ArH), 7.37–7.43 (m, 2H, ArH), 7.49 (ddd, *J* = 7.57, 4.66, 1.04 Hz, 2H, ArH), 7.90 (td, *J* = 7.67, 1.66 Hz, 1H, ArH), 8.11–8.19 (m, 1 H, ArH), 8.15 (d, *J* = 16.59 Hz, 1H, CH=C), 8.28 (d, *J* = 7.88 Hz, 1H, ArH), 8.40 (d, *J* = 16.59 Hz, 1H, CH=C), 8.67–8.72 (m, 1H, ArH). ^13^C NMR (101 MHz, DMSO-*d_6_*) 44.99, 49.37, 49.74–50.02, 51.79, 123.23, 123.29, 123.61, 124.67, 126.62, 127.12, 127.17, 127.30, 130.11, 135.68, 139.39, 140.2 (C-H), 1776.26, 1719 (C=O), 1623.69, 1603.44 (Ar C=C), 1523.46, 1458.07, (C-N) 1323, 1170 cm^−1^. HRMS (ESI): calculated for C_26_H_19_N_2_O_3_ [M^+^−H]: 407.1390; found: 407.1396.

9-(*E*)-3-(Furan-2-yl)-3-oxoprop-1-en-1-yl)-9,10-dihydro-9,10-[3,4]epipyrroloanthracene-12,14-dione (**23o**) was prepared from (*E*)-3-(anthracen-9-yl)-1-(furan-2-yl)prop-2-en-1-one (1.0 mmol, 0.293g) and maleimide (1.3 mmol, 0.13 g) according to the general procedure above; light yellow powder (81%), Mp. 268–270 °C. ^1^H NMR (400 MHz, CDCl_3_) δ 3.34 (dd, *J* = 8.6, 3.2 Hz, 1H), 3.54 (d, *J* = 8.6 Hz, 1H), 4.79 (d, *J* = 3.2 Hz, 1H), 6.61 (dd, *J* = 3.6, 1.7 Hz, 1H), 7.10–7.24 (m, 4H), 7.27 (dd, *J* = 6.9, 1.2 Hz, 1H), 7.34 (dd, *J* = 5.5, 3.2 Hz, 2H), 7.36–7.47 (m, 3H), 7.59–7.71 (m, 2H), 8.04 (d, *J* = 16.3 Hz, 1H)^. 13^C NMR (101 MHz, CDCl_3_) ppm 45.42, 49.06, 49.55, 51.92, 67.18, 112.64, 118.71, 123.52, 123.82, 123.99, 125.41, 126.67, 127.22, 127.29, 127.43, 130.42, 137.89, 139.25, 140.95, 141.73, 142.21, 147.10, 153.32, 174.77, 175.62, 177.38. IR *ν* max (ATR): 3343 (N-H), 3033.42 (C-H), 1776.09, 1719 (C=O), 1626.86, 1602.84 (Ar C=C), 1522.49, 1458.42 (C-N), 1330, 1168, (C-O), 1287.87, 1010.65 cm^−1^ HRMS (ESI): calculated for C_25_H_18_NO_4_ [M^+^+H]: 407.1390; found: 407.1396.

9-(*E*)-3-(Naphthalen-2-yl)-3-oxoprop-1-en-1-yl)-9,10-dihydro-9,10-[3,4]epipyrroloanthracene-12,14-dione (**23p**) was prepared from (*E*)-3-(anthracen-9-yl)-1-(naphthalen-2-yl)prop-2-en-1-one (1.0 mmol, 0.358 g) and maleimide (1.3 mmmol, 0.13 g) according to the general procedure above; cream/yellow powder (71%), Mp. 209–215 °C. ^1^H NMR (400 MHz, CDCl_3_) δ 3.42 (dd, *J* = 8.56, 3.18 Hz, 1H), 3.64 (d, *J* = 8.56 Hz, 1H), 4.86 (d, *J* = 3.18 Hz, 1H), 7.23–7.25 (m, 1H), 7.25–7.28 (m, 2H), 7.37–7.42 (m, 2H), 7.43–7.47 (m, 2H), 7.57–7.62 (m, 1H), 7.63–7.68 (m, 1H), 7.91–7.98 (m, 3H), 8.00–8.08 (m, 3H), 8.26 (dd, *J* = 8.56, 1.71 Hz, 3H), 8.76 (s, 3H). ^13^C NMR (101 MHz, CDCl_3_) ppm 45.5, 49.1, 49.6, 123.6, 123.9, 124.1, 124.6, 125.5, 126.8, 126.9, 127.3, 127.4, 127.5, 127.9, 128.6, 128.8, 129.8, 131.0, 131.6, 135.6, 142.1, 193.0. IR *ν* max (ATR): 3343 (N-H), 3062 (C-H), 1776.30 (C=O), 1719, (Ar C=C),1625.58, 1602.54 (C=C), 1522.97, 1457.95, (C-N) 1312, 1164 cm^−1^. HRMS (APCI): calculated for C_31_H_22_NO_3_ [M^+^+H]: 456.1594; found: 456.1599.

9-(*E*)-3-Oxo-3-(thiophen-2-yl)prop-1-en-1-yl)-9,10-dihydro-9,10-[3,4]epipyrroloanthracene-12,14-dione (**23q**) was prepared from (*E*)-3-(anthracen-9-yl)-1-(thiophen-2-yl)prop-2-en-1-one (1.0 mmol, 0.3144 g) and maleimide (1.3 mmol, 0.13 g) according to the general procedure above; beige/yellow powder (80%), Mp. 263–268 °C. ^1^H NMR (400 MHz, CDCl_3_) δ 3.35 (ddd, *J* = 8.5, 3.2, 0.6 Hz, 1H), 3.53 (d, *J* = 8.5 Hz, 1H), 4.80 (d, *J* = 3.1 Hz, 1H), 7.12–7.23 (m, 5H), 7.27–7.31 (m, 1H), 7.34 (ddd, *J* = 7.9, 4.1, 1.9 Hz, 2H), 7.38–7.44 (m, 1H), 7.63–7.78 (m, 2H), 7.94–8.03 (m, 2H). ^13^C NMR (101 MHz, CDCl_3_) ppm 45.40, 49.05, 49.60, 51.95, 123.51, 123.82, 124.00, 125.42, 126.71, 127.25, 127.31, 127.45, 128.42, 131.06, 132.95, 134.55, 137.91, 139.26, 140.93, 141.51, 142.25, 145.09, 174.85, 175.61, 181.86 (chalcone C=O). IR *ν* max (ATR): 3343 (N-H), 2963.67 (C-H), 1776.30, 1718, (C=O), 1623.56 (Ar C=C), 1582.14, 1524.12, 1457.91, 1324 (C-N), 1169, 720.73 cm^−1^. HRMS (APCI): calculated for C_25_H_18_NO_3_S [M^+^+H]: 412.1002; found: 412.1004.

### 3.5. General Preparation for (E)-9-(3-oxo-3-phenylprop-1-en-yl)-13-phenyl-9,10-dihydro-9,10-[3,4]epipyrroloanthracene-12,14-diones ***24a–q***

To a solution of the appropriate chalcone anthracene **21a–q** (1.0 mmol) in toluene (2 mL) was added dienophile *N*-phenylmaleimide (1.3 mmol). The mixture was heated and stirred at 90 °C for 48 h. The reaction was then cooled to room temperature, and the crude solid product was isolated via filtration, washed with toluene (2 mL) and diethyl ether (2 mL), and then recrystallized from toluene.

(*E*)-3-(4-Bromophenyl)-3-oxoprop-1-en-1-yl)-13-phenyl-9,10-dihydro-9,10-[3,4]epipyrroloanthracene-12,14-dione (**24a**) was obtained from (*E*)-3-(anthracen-9-yl)-1-(4-bromophenyl)prop-2-en-1-one (1.0 mmol, 0.387 g) and *N*-phenylmaleimide (1.3 mmol, 0.23 g) according to the general procedure above; white powder (84%), Mp. 218 °C. (HPLC: 94.98%: RT 1.90 min). ^1^H NMR (400 MHz, CDCl_3_) δ 3.47 (dd, *J* = 8.29, 3.32 Hz, 2H), 3.64 (d, *J* = 8.29 Hz, 2H), 4.92 (d, *J* = 2.90 Hz, 2H), 6.46–6.53 (m, 3H), 7.19–7.23 (m, 2H), 7.25–7.30 (m, 8H), 7.30–7.35 (m, 3H), 7.35–7.43 (m, 4H), 7.45 (dd, *J* = 7.05, 1.24 Hz, 2H), 7.61–7.65 (m, 3H), 7.80 (d, *J* = 16.17 Hz, 1H), 7.97–8.05 (m, 3H). ^13^C NMR (101 MHz, CDCl_3_) ppm 45.94, 47.95, 48.48, 52.49, 123.51, 123.92, 124.17, 125.55, 126.05, 126.44, 126.82, 127.38, 127.55, 127.94, 128.36, 128.91, 129.11, 129.13, 130.49, 131.14, 131.25, 132.06, 134.17, 136.45, 138.17, 139.39, 140.80, 142.17, 142.8 (C=C), 1490, 1455 (Ar C=C), 1380.41 (C-N), 1180 (C-O), 691.05 cm^−1^. LRMS (APCI) 560.08 [M^+^+H]. HRMS (ESI) calculated for C_33_H_22_BrNNaO_3_: 582.0675 [M^+^+Na]; found: 582.0668. *Alternative preparation of* **24a**: 9-(*E*)-3-(4-Bromophenyl)-3-oxoprop-1-en-1-yl)-9,10-dihydro-9,10-[3,4]furanoanthracene-12,14-dione (**22a**) (0.575 mmol) was treated with aniline (0.07 g, 0.748 mmol) in acetic acid (5 mL) at 120 °C for 2–3 h. The reaction mixture was cooled in an ice bath, and then deionized water was added. The colorless, solid product was filtered and dried (72%). The product was identical when compared with the sample obtained above.

9-(*E*)-3-(4-Nitrophenyl)-3-oxoprop-1-en-1-yl)-13-phenyl-9,10-dihydro-9,10-[3,4]epipyrroloanthracene-12,14-dione (**24b**) was prepared from (*E*)-3-(anthracen-9-yl)-1-(4-nitrophenyl)prop-2-en-1-one (1.0 mmol, 0.353 g) and *N*-phenylmaleimide (1.3 mmol, 0.23 g) according to the general procedure above; cream powder (86%), Mp. 242–246 °C. ^1^H NMR (400 MHz, CDCl_3_) δ 3.50 (dd, *J* = 8.4, 3.2 Hz, 1H), 3.67 (d, *J* = 8.5 Hz, 1H), 4.94 (d, *J* = 3.2 Hz, 1H), 6.54–6.45 (m, 2H), 7.40–7.17 (m, 9H), 7.46–7.37 (m, 1H), 7.51–7.43 (m, 1H), 7.85 (d, *J* = 16.2 Hz, 1H), 8.06 (d, *J* = 16.2 Hz, 1H), 8.38–8.26 (m, 4H). ^13^C NMR (101 MHz, CDCl_3_) ppm 45.92, 47.95, 48.43, 52.55, 123.39, 123.77, 123.95, 124.28, 125.65, 126.42, 126.87, 127.42, 127.51, 127.67, 128.99, 129.16, 129.96, 131.04, 131.17, 138.16, 139.18, 140.77, 141.95, 144.32, 174.72, 175.20 (chalcone C=O). IR *ν* max (ATR): 3335.77 (Ar C-H), 1771.68, 1700.63, 1671.17 (C=O), 1519, 1383 (NO_2_), 1179 (C-N) cm^−1^. LRMS (APCI): [M^+^+H] 527.14. HRMS (ESI) calculated for C_33_H_22_N_2_NaO_5_, 549.1421 [M^+^+Na]; found: 549.1424.

(*E*)-9-(3-(4-Ethylphenyl)-3-oxoprop-1-en-1-yl)-13-phenyl-9,10-dihydro-9,10-[3,4]epipyrroloanthracene-12,14-dione (**24c**) was synthesized from (*E*)-3-(anthracen-9-yl)-1-(4-ethylphenyl)prop-2-en-1-one (1.0 mmol, 0.336 g) and *N*-phenylmaleimide (1.3 mmol, 0.23 g) according to the general procedure above; light green powder (90%), Mp. 207 °C. IR *ν* max (ATR): 2969.28, 2922.38, 1708.43, 1380.94, 1178.76, 715.69, 753.23, 689.68 cm^−1^. ^1^H NMR (400 MHz, DMSO-*d_6_*) δ 3.57 (dd, *J* = 8.31, 3.42 Hz, 3H), 4.01 (d, *J* = 8.31 Hz, H), 4.97 (d, *J* = 2.93 Hz, 3H), 6.38–6.46 (m, 6H), 7.26–7.36 (m,2H), 7.36–7.39 (m, 3H), 7.40–7.43 (m, 1H), 7.59–7.65 (m, 1 H), 7.83 (d, *J* = 17.12 Hz, 1 H), 7.96 (d, *J* = 16.63 Hz, 1H), 8.01 (d, *J* = 5.87 Hz, 2 H), 8.89 (d, *J* = 5.87 Hz, 2H). ^13^C NMR (101 MHz, DMSO-*d_6_*) 15.6, 25.9, 28.7, 45.4, 48.3, 52.1, 123.4, 123.9, 124.8, 125.7, 126.9, 127.0, 127.2, 127.3, 127.4, 127.5, 128.2, 128.6, 128.8, 129.0, 129.3, 129.4, 132.0, 132.2, 135.1, 135.5, 139.2, 140.2, 141.7, 141.9, 142.9, 150.4, 170.4, 175.3, 175.7, 189.4 (chalcone C=O) ppm. LRMS (APCI) 510.20 [M+H]. HRMS (ESI) calculated for C_35_H_27_KNO_3_, 548.1623 [M^+^+K ]; found: 548.1627.

(*E*)-9-(3-(2,4-Dichlorophenyl)-3-oxoprop-1-en-1-yl)-13-phenyl-9,10-dihydro-9,10-[3,4]epipyrroloanthracene-12,14-dione (**24d**) was synthesized from (*E*)-3-(anthracen-9-yl)-1-(2,4-dichlorophenyl)prop-2-en-1-one (1.0 mmol, 0.377 g) and *N*-phenylmaleimide (1.3 mmol, 0.23 g) according to the general procedure above; white powder (75%), Mp. 208–210 °C. ^1^H NMR (400 MHz, CDCl_3_) δ 3.44 (dd, *J* = 8.29, 3.32 Hz, 1H), 3.55 (d, *J* = 8.29 Hz, 1H), 4.91 (d, *J* = 3.32 Hz, 1H), 6.46 (m, 2H), 7.24 (m, 7H), 7.35 (m, 5H), 7.46 (m, 1H), 7.50 (d, *J* = 1.66 Hz, H), 7.66 (d, *J* = 8.29 Hz, 1 H), 7.83 (d, *J* = 17.00 Hz, 1H), 7.79 (d, *J* = 17.00 Hz, 1H). ^13^C NMR (101 MHz, CDCl_3_) ppm 45.97, 48.23, 48.34, 52.30, 123.43, 123.76, 124.28, 125.57, 126.38, 126.81, 127.33, 127.43, 127.45, 127.57, 128.88, 129.08, 130.87, 131.16, 132.66, 134.88, 136.80, 138.15, 139.07, 140.78, 141.69, 144.77, 174.43, 175.25, 191.55 (chalcone C=O). IR *ν* max (ATR): 3067.32 (C-H), 1776.64, 1710.54, 1630.86 (C=O), 1381.66 (C-N), 1183.14 (C-O) cm^−1^. HRMS (ESI) calculated for C_33_H_20_Cl_2_NO_3_ [M^+^−H]: 548.0826; found: 548.0825.

9-(*E*)-3-(3,4-Dimethoxyphenyl)-3-oxoprop-1-en-1-yl)-13-phenyl-9,10-dihydro-9,10-[3,4]epipyrroloanthracene-12,14-dione (**24e**) was prepared from (*E*)-3-(anthracen-9-yl)-1-(3,4-dimethoxyphenyl)prop-2-en-1-one (1.0 mmol, 0.368 g) and *N*-phenylmaleimide (1.3 mmol, 0.23 g) according to the general procedure above; white powder (93%), Mp. 242–244 °C. ^1^H NMR (400 MHz, CDCl_3_) δ 3.47 (dd, *J* = 8.4, 3.3 Hz, 1H), 3.65 (d, *J* = 8.4 Hz, 1H), 3.94 (d, *J* = 9.4 Hz, 6H), 4.92 (d, *J* = 3.2 Hz, 1H), 6.45–6.54 (m, 2H), 6.91 (d, *J* = 8.4 Hz, 1H), 7.20 (td, *J* = 7.5, 1.6 Hz, 1H), 7.20–7.28 (m, 3H), 7.24–7.32 (m, 3H), 7.34–7.44 (m, 3H), 7.42–7.48 (m, 1H), 7.75 (d, *J* = 2.0 Hz, 1H), 7.80–7.89 (m, 2H), 7.95 (d, *J* = 16.1 Hz, 1H). ^13^C NMR (101 MHz, CDCl_3_) ppm 45.96, 47.99, 48.51, 52.48, 56.02, 56.08, 110.18, 110.99, 123.60, 123.84, 124.08, 125.49, 126.43, 126.79, 127.30, 127.33, 127.47, 128.82, 129.05, 130.89, 131.54, 138.21, 139.62, 140.83, 141.01, 142.42, 153.49, 188.72 (chalcone C=O). IR *ν* max (ATR): 3011.75 (Ar C-H), 1775.53, 1708.96, 1676.49 (C=O), 1379.80 (C-N), 1181.33 (C-O) cm^−1^. LRMS (APCI): 542.28 [M^+^+H]. HRMS (ESI) calculated for C_35_H_27_NNaO_5_ [M^+^+Na]: 564.1781; found 564.1783.

(*E*)-9-(3-Oxo-3-phenylprop-1-en-yl)-13-phenyl-9,10-dihydro-9,10-[3,4]epipyrroloanthracene-12,14-dione (**24f**) was synthesized from (*E*)-3-(anthracen-9-yl)-1-phenylprop-2-en-1-one (1.0 mmol, 0.308 g) and *N*-phenylmaleimide (1.3 mmol, 0.23 g) according to the general procedure above; white powder (42%). Mp. 103–105 °C. ^1^H NMR (400 MHz, DMSO-*d_6_*) δ 3.54 (dd, *J* = 8.29, 3.32 Hz, 1H) 4.00 (d, *J* = 8.29 Hz, 1H) 4.94 (d, *J* = 3.32 Hz, H) 6.39–6.43 (m, 2H) 7.19–7.27 (m, 3H) 7.27–7.34 (m, 6H) 7.36–7.41 (m, 1H) 7.57–7.62 (m, 3H) 7.66–7.71 (m, 1H) 7.88 (d, *J* = 1.66 Hz, 2H) 8.13–8.17 (m, 2H). ^13^C NMR (101 MHz, DMSO-*d_6_*) 45.41, 48.33, 52.15, 122.71, 123.42, 123.90, 124.86, 125.72, 126.88, 127.06, 127.44, 128.95, 129.20, 129.36, 132.01, 132.18, 133.88, 137.74, 139.24, 140.16, 141.70, 142.35, 142.82, 175.35, 175.76, 189.96 (chalcone C=O) ppm. IR *ν* max (ATR): 1707.50 (C=O), 1673.57 (C=C), 1636.62 (Ar C=C),1332.23 (C-N) cm^−1^. LRMS (APCI) 482.20 [M^+^+H].

9-(*E*)-3-(4-Iodophenyl)-3-oxoprop-1-en-1-yl)-13-phenyl-9,10-dihydro-9,10-[3,4]epipyrroloanthracene-12,14-dione (**24g**) was prepared from (*E*)-3-(anthracen-9-yl)-1-(4-iodophenyl)prop-2-en-1-one (1.0 mmol, 0.434 g) and *N*-phenylmaleimide (1.3 mmol, 0.23 g) according to the general procedure above; white crystals (20%), Mp. 214 °C. ^1^H NMR (400 MHz, DMSO-*d_6_*) δ ppm 3.51 (dd, *J* = 8.3, 3.2 Hz, 1H), 3.97 (d, *J* = 8.4 Hz, 1H), 4.92 (d, *J* = 3.2 Hz, 1H), 6.34–6.43 (m, 2H), 7.09–7.16 (m, 1H), 7.18–7.23 (m, 3H), 7.25–7.33 (m, 7H), 7.33–7.39 (m, 1H), 7.41–7.50 (m, 1H), 7.57 (dt, *J* = 7.1, 1.2 Hz, 1H), 7.78–7.85 (m, 2H), 7.86–7.91 (m, 2H), 7.94–8.01 (m, 2H). ^13^C NMR (101 MHz, DMSO-*d_6_*) 44.98, 49.13, 49.32, 51.72, 102.63, 123.26, 123.67, 124.62,125.65, 126.66, 127.17, 127.29, 130.84,131.45, 137.06, 138.35, 139.30, 140.27, 142.17, 143.05, 143.12, 177.48, 177.74, 189.40 (chalcone C=O) ppm. IR *ν* max (ATR): 3088.53 (C-H), 1772.98, 1705 (C=O), 1633.80 (Ar C=C), 1587.05, 1499.05, (C-N) 1467.03, 1381, 1180, 691.17 cm^−1^. LRMS (APCI) [M^+^+H]: found: 608.03. HRMS (ESI) calculated for C_33_H_21_INO_3_, 606.0572 [M^+^−H]; found: 606.0567.

9-(*E*)-3-(4-Fluorophenyl)-3-oxoprop-1-en-1-yl)-13-phenyl-9,10-dihydro-9,10-[3,4]epipyrroloanthracene-12,14-dione (**24h**) was prepared from (*E*)-3-(anthracen-9-yl)-1-(4-fluorophenyl)prop-2-en-1-one (1.0 mmol, 0.434 g) and *N*-phenylmaleimide (1.3 mmol, 0.23 g) according to the general procedure above; green solid (88%), Mp. 79 °C. (HPLC: 96.41%, RT 10.04 min). ^1^H NMR (400 MHz, CDCl_3_) δ 3.48 (dd, *J* = 8.4, 3.2 Hz, 1H), 3.66 (d, *J* = 8.4 Hz, 1H), 4.93 (d, *J* = 3.2 Hz, 1H), 6.44–6.54 (m, 2H), 7.13–7.21 (m, 2H), 7.22 (dd, *J* = 3.5, 1.6 Hz, 1H), 7.25–7.31 (m, 5H), 7.31–7.42 (m, 4H), 7.43–7.49 (m, 1H), 7.83 (d, *J* = 16.1 Hz, 1H), 8.00 (d, *J* = 16.1 Hz, 1H), 8.15–8.24 (m, 2H). ^13^C NMR (101 MHz, CDCl_3_) ppm 45.39, 48.93, 49.61, 52.01, 115.81, 116.03, 123.41, 123.79, 124.03, 125.47, 126.74, 127.28, 127.32, 127.49, 131.19, 131.58, 131.67, 134.09, 134.12, 137.94, 139.26, 140.92, 142.18, 142.28, 175.17, 175.73, 188.70 (chalcone C=O). LRMS (APCI) 500.20 [M^+^+H]. HRMS (ESI) calculated for C_33_H_22_FNNaO_3_, 522.1476 [M^+^+H]; found 522.1473.

(*E*)-9-(3-(4-Methoxyphenyl)-3-oxoprop-1-en-1-yl)-13-phenyl-9,10-dihydro-9,10-[3,4]epipyrroloanthracene-12,14-dione (**24i**) was synthesized from (*E*)-3-(anthracen-9-yl)-1-(4-methoxyphenyl)prop-2-en-1-one (1.0 mmol, 0.338 g) and *N*-phenylmaleimide (1.3 mmol, 0.23 g) according to the general procedure above; white powder (56%), Mp. 207–212 °C. ^1^H NMR (400 MHz, CDCl_3_) δ 3.55 (dd, *J* = 8.29, 3.32 Hz, 1H), 3.85 (s, 3H), 3.99 (d, *J* = 8.29 Hz, 1H), 4.94 (d, 1H, *J* = 3.7 Hz, 1H), 7.09–7.18 (m, 5H), 7.20–7.34 (m, 11H), 7.81 (d, J = 16.3 Hz, 1H), 7.89 (d, *J* = 16.3 Hz, 1H), 8.16 (d, *J* = 9.12Hz, 2H). ^13^C NMR (101 MHz, CDCl_3_) ppm 45.42, 48.27, 48.34, 52.04, 56.03, 114.67, 122.22, 123.41, 123.90, 124.83, 125.71, 126.85, 127.07, 127.26–127.55, 128.64, 129.00, 129.32, 130.59, 131.53, 131.92, 132.19, 139.24, 140.28, 141.20, 141.72, 142.95, 163.88, 175.34, 175.76, 177.93, 188.14 (chalcone C=O). IR *ν* max (ATR): 3343.74 (N-H), 1670.84 (C=O), 1600.33 (Ar C=C), 1380.68, 1230.69 (C-O), 1024.50 (C-N) cm^−1^. LRMS (APCI) found: 512.19, [M^+^+H]. HRMS (ESI) calculated for C_34_H_24_NO_4_ [M^+^−H]: 510.1705; found: 510.1708.

(*E*)-9-(3-Oxo-3-(*p*-tolyl)prop-1-en-1-yl)-13-phenyl-9,10-dihydro-9,10-[3,4]epipyrroloanthracene-12,14-dione (**24j**) was synthesized from (*E*)-3-(anthracen-9-yl)-1-(*p*-tolyl)prop-2-en-1-one (1.0 mmol, 0.338 g) and *N*-phenylmaleimide (1.3 mmol, 0.23 g) according to the general procedure above; white powder (51%), Mp. 185–190 °C. ^1^H NMR (400 MHz, CDCl_3_) δ 2.39 (s, 3H), 3.53 (dd, *J* = 8.50, 3.11 Hz, 1H), 3.99 (d, *J* = 8.71 Hz, 1H), 4.94 (d, *J* = 2.90 Hz, 1H), 6.38–6.44 (m, 2H), 7.19–7.26 (m, 3H), 7.26–7.34 (m, 6H), 7.36–7.42 (m, 3H), 7.59 (d, *J* = 6.63 Hz, 1H), 7.86 (d, *J* = 3.32 Hz, 2H), 8.06 (d, *J* = 8.29 Hz, 2H). ^13^C NMR (101 MHz, CDCl_3_) ppm 21.69, 39.22–40.74, 48.34, 52.13, 123.89, 125.38 -125.98, 126.76–127.19, 127.26–127.61, 128.92–129.49, 129.95, 131.97, 135.25, 140.21, 141.91, 144.37, 175.34, 189.38 (chalcone C=O). IR *ν* max (ATR): 2917.28 (Ar C-H), 1772.13 (C=O), 1624.76 (Ar C=C), 1458.34 (C-CH_3_), 1230.53 (C-O), 1072.21 (C-N) cm^−1^. LRMS (APCI) 496.19 [M^+^+H]. HRMS (ESI) calculated for C_34_H_25_KNO_3_ [M^+^+K] 534.1466; found 534.1460.

(*E*)-9-(3-Oxo-3-(3,4,5-trimethoxyphenyl)prop-1-en-1-yl)-13-phenyl-9,10-dihydro-9,10-[3,4] epipyrroloanthracene-12,14-dione (**24k**) was synthesized from (*E*)-3-(anthracen-9-yl)-1-(3,4,5-trimethoxyphenyl)prop-2-en-1-one (1.0 mmol, 0.398 g) and *N*-phenylmaleimide (1.3 mmol, 0.23 g) according to the general procedure above; white powder (62%), Mp. 242–246 °C. ^1^H NMR (400 MHz, CDCl_3_) δ 3.53 (dd, *J* = 8.50, 3.11 Hz, 1H), 3.76 (s, 3H), 3.68 (s, 6H), 4.00 (d, *J* = 8.29 Hz, 1H), 4.94 (d, *J* = 3.32 Hz, 1H), 6.42 (dd, *J* = 7.46, 2.07 Hz, 2H), 7.23–7.26 (m, 2H), 7.26–7.33 (m, 6H), 7.34 (s, 1H), 7.37–7.40 (m, H), 7.47 (s, 2H), 7.58–7.61 (m, 1H), 7.78–7.91 (m, 2H). ^13^C NMR (101 MHz, CDCl_3_) ppm 45.43, 48.1–48.62, 56.54, 60.67, 106.88, 123.42, 123.95, 124.88, 125.71, 126.75 -126.96, 127.29–127.48, 128.94, 129.26, 132.13, 132.50, 133.05, 140.18, 141.65, 153.41, 175.39, 175.73, 189.11 (chalcone C=O) IR *ν* max (ATR): 1702.71 (C=.O), 1123.37 (C-O) cm^−1^. LRMS (APCI) 572.19 [M^+^+H]. HRMS (ESI) calculated for C_36_H_28_NO_6_ [M^+^−H]: 570.1922; found: 570.1920

(*E*)-9-(3-(4-Chlorophenyl)-3-oxoprop-1-en-1-yl)-13-phenyl-9,10-dihydro-9,10-[3,4] epipyrroloanthracene-12,14-dione (**24l**) was synthesized from (*E*)-3-(anthracen-9-yl)-1-(4-chlorophenyl) prop-2-en-1-one (1.0 mmol, 0.343 g) and *N*-phenylmaleimide (1.3 mmol, 0.23 g) according to the general procedure above; white powder (20%), Mp. 207–211 °C. ^1^H NMR (400 MHz, DMSO-*d_6_*) δ 3.53 (dd, *J* = 8.29, 3.32 Hz, 1H), 3.99 (d, *J* = 8.29Hz, 1H), 4.94 (d, *J* = 3.32 Hz, 1H), 6.37–6.44 (m, 2H), 7.23 (d, *J* = 0.83 Hz, 3H), 7.27–7.35 (m, 6H), 7.35–7.40 (m, 1H), 7.59 (d, *J* = 6.63 Hz, 1H), 7.67 (d, *J* = 8.29 Hz, 2H), 7.87 (s, 2H), 8.16 (d, *J* = 8.29 Hz, 2H). ^13^C NMR (101 MHz, CDCl_3_) ppm: 39.12–40.77, 45.42, 52.17, 123.44, 123.92, 124.86, 125.72, 126.72 -127.66, 129.24–129.66, 131.03, 131.75, 132.16, 135.09, 138.84, 139.21, 141.68, 142.74, 142.92, 175.37, 175.76, 188.95 (chalcone C=O). IR *ν* max (ATR): 3058.71 (Ar C-H), 1708.71(C=O), 1674.04 (C=C), 1591.39 (Ar C=C), 1455.69 (C-C), 1015.46 (C-N), 743.22 (C-Cl) cm^−1^. LRMS (APCI) 516.13 [M^+^+H]. HRMS (ESI) calculated for C_33_H_23_ClNO_3_ [M^+^+H]: 516.1361; found: 516.1361.

9-(*E*)-3-Oxo-3-(pyridin-4-yl)prop-1-en-1-yl)-13-phenyl-9,10-dihydro-9,10-[3,4]epipyrroloanthracene-12,14-dione (**24m**) was prepared from (*E*)-3-(anthracen-9-yl)-1-(pyridin-4-yl)prop-2-en-1-one (1.0 mmol, 0.309 g) and *N*-phenylmaleimide (1.3 mmol, 0.23 g) according to the general procedure above; cream crystals (82%), Mp. 203–206 °C. ^1^H NMR (400 MHz, DMSO-*d_6_*) δ 3.57 (dd, *J* = 8.4, 3.2 Hz, 1H, H-8), 4.01 (d, *J* = 8.4 Hz, 1H, H-3), 4.97 (d, *J* = 3.2 Hz, 1H, H7), 6.42 (d, *J* = 15.1 Hz, 1H), 7.16–7.62 (m, 14H), 8.01 (m, 2H), 8.88 (m, 2H). ^13^C NMR (101 MHz, DMSO-*d_6_*) ppm 44.93 (C-7), 47.78 (C-8), 48.04 (C-3), 51.76 (C-4), 121.73, 123.03, 123.50, 124.43, 126.41, 126.58, 126.79, 126.93, 127.06, 128.55, 128.86, 131.30, 131.67, 134.67, 138.74, 139.46, 141.18, 142.09, 143.29, 143.96, 150.89, 174.93, 175.28, 189.58 (chalcone C=O). ^15^N (400 MHz, DMSO-*d_6_*) 194.8 maleimide), 329.7 (pyridine) ppm. HRMS (ESI) calculated for C_32_H_22_N_2_NaO_3_ [M^+^+Na]: 505.1523; found 505.1519.

9-(*E*)-3-Oxo-3-(pyridin-2-yl)prop-1-en-1-yl)-13-phenyl-9,10-dihydro-9,10-[3,4]epipyrroloanthracene-12,14-dione (**24n**) was prepared from (*E*)-3-(anthracen-9-yl)-1-(pyridin-2-yl)prop-2-en-1-one (1.0 mmol, 0.309 g) and *N*-phenylmaleimide (1.3 mmol, 0.23 g) according to the general procedure above; gray powder (91%), Mp. >256 °C (dec). ^1^H NMR (400 MHz, CDCl_3_) δ 3.47 (dd, *J* = 8.4, 3.2 Hz, 1H), 3.75 (d, *J* = 8.4 Hz, 1H), 4.92 (d, *J* = 3.2 Hz, 1H), 6.44–6.53 (m, 2H), 7.14–7.30 (m, 7H), 7.31–7.42 (m, 2H), 7.46 (ddt, *J* = 7.2, 6.1, 1.1 Hz, 3H), 7.88 (td, *J* = 7.7, 1.8 Hz, 1H), 8.21 (d, *J* = 16.6 Hz, 1H), 8.26 (dt, *J* = 7.9, 1.1 Hz, 1H), 8.49 (d, *J* = 16.5 Hz, 1H), 8.69 (ddd, *J* = 4.8, 1.8, 1.0 Hz, 1H). ^13^C NMR (101 MHz, CDCl_3_) ppm 46.06, 48.18, 48.39, 52.51, 123.15, 123.74, 124.07, 124.15, 125.42, 126.41, 126.70, 127.00, 127.21, 127.31, 127.38, 128.69, 128.94, 130.07, 131.31, 137.00, 138.22, 139.70, 140.93, 142.30, 142.60, 149.08, 174.40, 175.50, 188.99 (chalcone C=O). IR *ν* max (ATR): 3054.46 (Ar C-H), 1779.59, 1713.79, 1674.55 (C=O), 1201.95 (C-N), 1181.33 (C-O) cm^−1^. LRMS (APCI) 483.23 [M^+^+H].

9-(*E*)-3-(Furan-2-yl)-3-oxoprop-1-en-1-yl)-13-phenyl-9,10-dihydro-9,10-[3,4]epipyrroloanthracene-12,14-dione (**24o**) was prepared from (*E*)-3-(anthracen-9-yl)-1-(furan-2-yl)prop-2-en-1-one (1.0 mmol, 0.298 g) and *N*-phenylmaleimide according to the general procedure above; cream powder (87%), Mp. >200 °C. ^1^H NMR (400 MHz, CDCl_3_) δ 3.47 (dd, *J* = 8.4, 3.2 Hz, 1H), 3.65 (d, *J* = 8.4 Hz, 1H), 4.92 (d, *J* = 3.2 Hz, 1H), 6.43–6.53 (m, 2H), 6.58 (dd, *J* = 3.6, 1.7 Hz, 1H), 7.13–7.29 (m, 5H), 7.25–7.31 (m, 2H), 7.33 (dd, *J* = 7.4, 1.5 Hz, 1H), 7.36–7.44 (m, 2H), 7.41–7.49 (m, 2H), 7.66 (dd, *J* = 1.7, 0.8 Hz, 1H), 7.71 (d, *J* = 16.3 Hz, 1H), 8.09 (d, *J* = 16.3 Hz, 1H). ^13^C NMR (101 MHz, CDCl_3_) ppm 45.96, 48.07, 48.46, 52.41, 112.61, 118.83, 123.63, 123.99, 124.13, 125.49, 126.40, 126.78, 127.34, 127.50, 128.81, 129.04, 130.61, 131.25, 138.15, 139.45, 140.83, 141.76, 142.19, 147.13, 175.35. IR *ν* max (ATR): 1738.78, 1695.65, 1665.48, 1646.52, 1616.16, 1562.62 cm^−1^. LRMS (APCI) 472.19 [M^+^+H]. HRMS (ESI) calculated for C_31_H_20_NO_4_ [M^+^−H]: 470.1398; found: 470.1394

9-(*E*)-3-(Naphthalen-2-yl)-3-oxoprop-1-en-1-yl)-13-phenyl-9,10-dihydro-9,10-[3,4]epipyrroloanthracene-12,14-dione (**24p**) was prepared from (*E*)-3-(anthracen-9-yl)-1-(naphthalen-2-yl)prop-2-en-1-one (1.0 mmol, 0.358 g) and *N*-phenylmaleimide (1.3 mmol, 0.23g) according to the general procedure above; light green powder (65%), Mp. 202–206 °C. ^1^H NMR (400 MHz, CDCl_3_) δ 3.52 (dd, *J* = 8.4, 3.2 Hz, 1H), 3.69 (d, *J* = 8.5 Hz, 1H), 4.94 (d, *J* = 3.2 Hz, 1H), 6.64–6.71 (m, 2H), 7.10–7.22 (m, 2H), 7.19–7.25 (m, 1H), 7.22–7.31 (m, 3H), 7.33–7.42 (m, 2H), 7.38–7.48 (m, 3H), 7.44–7.64 (m, 2H), 7.67–7.75 (m, 4H), 7.77 (s, 1H), 7.97 (dd, *J* = 3.8, 1.1 Hz, 1H), 8.05 (d, *J* = 16.1 Hz, 1H). IR *ν* max (ATR): 3068.21 (Ar C-H), 1773.41, 1710.67, 1675.13 (C=O), 1380.71 (C-N), 1195.89 (C-O) cm^−1^. LRMS (APCI) 530.31 [M^+^−H]. HRMS (ESI) calculated for C_37_H_24_NO_3_ [M^+^−H]: 530.1762; found: 530.1751.

9-(*E*)-3-Oxo-3-(thiophen-2-yl)prop-1-en-1-yl)-13-phenyl-9,10-dihydro-9,10-[3,4]epipyrroloanthracene-12,14-dione (**24q**) was prepared from (*E*)-3-(anthracen-9-yl)-1-(thiophen-2-yl)prop-2-en-1-one (1.0 mmol, 0.314 g) and *N*-phenylmaleimide (1.3 mmol, 0.23 g) according to the general procedure above; cream powder (81%), Mp. 233–236 °C. ^1^H NMR (400 MHz, CDCl_3_) δ 3.48 (dd, *J* = 8.4, 3.2 Hz, 1H), 3.65 (d, *J* = 8.4 Hz, 1H), 4.93 (d, *J* = 3.2 Hz, 1H), 6.45–6.54 (m, 2H), 7.14–7.19 (m, 1H), 7.19–7.24 (m, 1H), 7.24–7.31 (m, 6H), 7.38 (ddd, *J* = 13.1, 6.4, 2.5 Hz, 3H), 7.44–7.49 (m, 1H), 7.70 (dd, *J* = 4.9, 1.1 Hz, 1H), 7.77 (d, *J* = 16.1 Hz, 1H), 7.98 (dd, *J* = 3.8, 1.1 Hz, 1H), 8.04 (d, *J* = 16.1 Hz, 1H). ^13^C NMR (101 MHz, CDCl_3_) ppm 45.96, 48.06, 48.51, 52.44, 123.63, 124.01, 124.14, 125.51, 126.43, 126.82, 127.36, 127.52, 128.43, 128.85, 129.08, 131.20, 133.10, 134.54, 138.17, 139.46, 140.82, 141.60, 142.24, 174.57. IR *ν* max (ATR): 1729.12, 1660.73, 1393.28, 1327.06, 1204.75 cm^−1^. LRMS (APCI) [M^+^+Na]: 510.30. HRMS (ESI) calculated for C_31_H_21_NNaO_3_S [M^+^+Na]: 510.1134; found: 510.1132.

### 3.6. General Preparation for (E)-13-(4-Chlorophenyl)-9-(3-oxo-3-phenylprop-1-en-1-yl)-9,10-dihydro-9,10-[3,4]epipyrroloanthracene-12,14-diones ***25a–q***

To a solution of the appropriate chalcone anthracene **21a–q** (1.0 mmol) in toluene (2 mL) was added dienophile 1-(4-chlorophenyl)-1*H*-pyrrole-2,5-dione (1.3 mmol). The mixture was stirred and heated at 90 °C for 48 h. The reaction was then cooled to room temperature, and the resulting solid was isolated via filtration. The crude product was washed with toluene (2 mL) and diethyl ether (2 mL) and then recrystallized from toluene.

(*E*)-9-(3-(4-Bromophenyl)-3-oxoprop-1-en-1-yl)-13-(4-chlorophenyl)-9,10-dihydro-9,10-[3,4]epipyrroloanthracene-12,14-dione (**25a**) was prepared from (*E*)-3-(anthracen-9-yl)-1-(4-bromophenyl)prop-2-en-1-one (1.0 mmol, 0.387 g) and 1-(4-chlorophenyl)-1*H*-pyrrole-2,5-dione (1.3 mmol, 0.27 g) according to the general procedure above; white powder (95%) Mp. 253–254 °C. ^1^H NMR (400 MHz, CDCl_3_) δ 3.52 (dd, J = 8.32, 3.15 Hz, 1H), 4.01 (d, *J* = 8.33 Hz, 1H), 4.94 (d, *J* = 3.22 Hz, 1H), 6.47 (d, *J* = 8.28 Hz, 2H), 7.14–7.32 (m, 9H), 7.45–7.56 (m, 1H), 7.60 (d, *J* = 6.3 Hz, 1H), 7.82 (m, 2H), 7.85 (d, J = 16.12 Hz, 1 H), 7.97 (d, *J* = 8.4 Hz, 2H). ^13^C NMR (101 MHz, CDCl_3_) 45.96, 48.00, 48.47, 52.52, 123.53, 123.98, 124.22, 125.56, 126.92, 127.43, 127.48, 127.62, 127.71, 128.46, 129.38, 129.65, 130.50, 131.21, 132.12, 134.86, 136.44, 138.13,139.37, 140.66, 142.05, 142.68, 174.42, 175.10, 189.26 ppm. IR *ν* max (ATR): 1708.82, 1673.68, 1624.31, 1585.32, 1492.81, 1386.87, 1315.79, 1189.52, 1067.58, 1005.54, 850.69, 830.15 cm^−1^. HRMS (ESI) calculated for C_33_H_21_BrClNNaO_3_ [M^+^+Na]: 616.0286; found: 616.0287.

(*E*)-13-(4-Chlorophenyl)-9-(3-(4-nitrophenyl)-3-oxoprop-1-en-1-yl)-9,10-dihydro-9,10-[3,4]epipyrroloanthracene-12,14-dione (**25b**) was synthesized from (*E*)-3-(anthracen-9-yl)-1-(4-nitrophenyl)prop-2-en-1-one (1.0 mmol, 0.353 g) and 1-(4-chlorophenyl)-1*H*-pyrrole-2,5-dione (1.3 mmol, 0.27 g) according to the general procedure above; white powder (82%), ^1^H NMR (400 MHz, CDCl_3_) δ 3.51 (dd, *J* = 8.50, 3.11 Hz, 4 H), 3.67 (d, *J* = 8.29 Hz, 1 H), 4.94 (d, *J* = 3.32 Hz, 1 H), 6.47 (d, *J* = 9.12 Hz, 2 H), 7.20–7.31 (m, 6 H), 7.31–7.38 (m, 2 H), 7.39–7.45 (m, 1 H), 7.46–7.51 (m, 1 H), 7.84 (d, *J* = 16.17 Hz, 1 H), 8.06 (d, *J* = 16.17 Hz, 1 H), 8.32 (d, *J* = 8.71 Hz, 2 H), 8.37 (d, *J* = 8.71 Hz, 2 H. IR *ν* max (ATR): 1739.76, 1665.28, 1646.57, 1562.09, 1494.06 cm^−1^. ^13^C NMR (101 MHz, CDCl_3_) 121.93, 123.44, 123.80, 125.15, 125.30, 125.58, 125.62, 126.00, 126.39, 126.51, 127.27, 127.35, 127.52, 127.72, 127.77, 128.06, 128.56, 128.73, 128.80, 129.03, 130.79, 131.16, 133.12, 134.17, 136.03, 138.44. HRMS (ESI) calculated for C_33_H_21_Cl_2_N_2_O_5_ [M^+^+Cl]: 595.0833; found: 595.0820.

(*E*)-13-(4-Chlorophenyl)-9-(3-(4-ethylphenyl)-3-oxoprop-1-en-1-yl)-9,10-dihydro-9,10-[3,4]epipyrroloanthracene-12,14-dione (**25c**) was synthesized from (*E*)-3-(anthracen-9-yl)-1-(4-ethylphenyl)prop-2-en-1-one (1 mmol, 0.336 g) and 1-(4-chlorophenyl)-1*H*-pyrrole-2,5-dione (1.3 mmol, 0.27 g) according to the general procedure above; white powder (3%), Mp. >220 (dec). ^1^H NMR (400 MHz, CDCl_3_) δ 1.19–1.25 (m, 3H), 2.37 (m, 2H), 3.54 (dd, *J* = 8.27, 3.39 Hz, 1H), 3.98 (d, *J* = 8.27 Hz, 1 H), 4.97 (d, *J* = 2.91 Hz, 1 H), 6.39–6.47 (m, 2H), 7.23–7.32 (m, 5H), 7.34–7.36 (m, 1 H), 7.37–7.43 (m, 3H), 7.47–7.59 (m, 2 H), 7.78 (d, *J* = 16.2 Hz, 1 H), 7.83 (d, *J* = 5.87 Hz, 2 H), 7.98 (d, *J* = 5.87 Hz, 2 H). IR *ν* max (ATR): 1733.88, 1695.56, 1665.62, 1646.02, 1616.24, 1562.03, 1520.74 cm^−1^. ^13^C NMR (101 MHz, CDCl_3_) 15.22, 29.04, 46.00, 48.05, 48.49, 52.51, 123.66, 124.12, 124.14, 125.49, 126.89, 127.39, 127.53, 127.72, 128.31, 129.22, 129.34, 129.36, 129.71, 131.71, 134.78, 135.42, 138.15, 139.56, 140.70, 141.53, 142.24, 150.28, 174.32, 175.15, 189.78 ppm. HRMS (ESI) calculated for C_35_H_26_ClNNaO_3_ [M^+^+Na]: 566.1493; found: 556.1500.

(*E*)-13-(4-Chlorophenyl)-9-(3-(2,4-dichlorophenyl)-3-oxoprop-1-en-1-yl)-9,10-dihydro-9,10-[3,4]epipyrroloanthracene-12,14-dione (**25d**) was synthesized from (*E*)-3-(anthracen-9-yl)-1-(2,4-dichlorophenyl)prop-2-en-1-one (1.0 mmol, 0.309 g) and 1-(4-chlorophenyl)-1*H*-pyrrole-2,5-dione (1.3 mmol, 0.27 g) according to the general procedure above; white powder (87%), Mp. 251 (dec) °C. ^1^H NMR (400 MHz, CDCl_3_) δ 3.45 (dd, *J* = 8.50, 3.11 Hz, 3H), 3.56 (d, *J* = 8.29 Hz, 2H), 4.91 (d, *J* = 3.32 Hz, 2H), 6.43 (d, *J* = 9.12 Hz, 2H), 7.19–7.23 (m, 1H), 7.25 -7.30 (m, 4H), 7.30–7.37 (m, 3H), 7.41 (d, *J* = 2.07 Hz, 1H), 7.43 -7.48 (m, 1H), 7.52 (d, *J* = 1.66 Hz, 1H), 7.67 (d, *J* = 8.29 Hz, 1H), 7.81 (d, *J* = 16.59 Hz, 1H). IR *ν* max (ATR): 1738.28, 1695.68, 1648.38, 1616.26, 1562.04, 1520.75, 1451.55 cm^−1^. ^13^C NMR (101 MHz, CDCl_3_) 46.00, 48.23, 48.37, 52.33, 123.46, 123.83, 124.34, 125.59, 126.92, 127.40, 127.53, 27.64, 127.67, 129.36, 129.59, 130.45, 130.91, 132.71, 134.84, 134.94, 136.81, 137.53, 138.13, 139.06, 140.66, 141.59, 144.60, 174.21, 175.04, 191.56.ppm. HRMS (ESI) calculated for C_32_H_20_Cl_4_NO_3_ [M^+^+Cl]: 618.0203; found: 618.0193.

(*E*)-13-(4-Chlorophenyl)-9-(3-(3,4-dimethoxyphenyl)-3-oxoprop-1-en-1-yl)-9,10-dihydro-9,10-[3,4]epipyrroloanthracene-12,14-dione (**25e**) was synthesized from (*E*)-3-(anthracen-9-yl)-1-(3,4-dimethoxyphenyl)prop-2-en-1-one (1.0 mmol, 0.368 g) and 1-(4-chlorophenyl)-1*H*-pyrrole-2,5-dione (1.3 mmol, 0.27 g) according to the general procedure above; white powder (4%) Mp. 167–169 °C. ^1^H NMR (400 MHz, CDCl_3_) δ 2.83 (d, *J* = 4.98 Hz, 1H), 2.88 (d, *J* = 4.98 Hz, 1H), 3.49 (dd, *J* = 8.09, 3.11 Hz, 1H), 3.71 (d, *J* = 9.12 Hz, 1H), 4.93 (d, *J* = 3.73 Hz, 1H), 6.48 (d, *J* = 9.12 Hz, 1H), 7.50–7.67 (m, 5H), 7.87–7.91 (m, 1H), 7.93–7.99 (m, 1H), 8.06 (d, *J* = 15.76 Hz, 1H), 8.21 (d, *J* = 9.54 Hz, 1H). IR *ν* max (ATR): 1713.42, 1604.96, 1542.00, 1493.58, 1397.57, 1247.61, 1184.72 1091.31 cm^−1^. ^13^C NMR (101 MHz, CDCl_3_) 35.72, 67.62, 123.46, 123.83, 124.34, 125.59, 126.92, 127.40, 127.53, 127.64, 127.67, 129.36, 129.59, 130.45, 130.91, 132.71, 134.84, 134.94, 136.81, 137.53, 138.13, 139.06, 140.66, 141.60, 144.40, 169.94, 171.92, 172.24 ppm; LRMS (APCI) 514.20. HRMS 621.1041, found 621.1008.

(*E*)-13-(4-Chlorophenyl)-9-(3-oxo-3-phenylprop-1-en-1-yl)-9,10-dihydro-9,10-[3,4]epipyrroloanthracene-12,14-dione (**25f**) was synthesized from (*E*)-3-(anthracen-9-yl)-1-phenylprop-2-en-1-one (1.0 mmol, 0.308g) and 1-(4-chlorophenyl)-1*H*-pyrrole-2,5-dione (1.3 mmol, 0.27 g) according to the general procedure above; white powder (48%), Mp. 205–210 °C. ^1^H NMR (400 MHz, DMSO-*d_6_*) δ 3.53 (dd, *J* = 8.50, 3.11 Hz, 1H) 4.00 (d, *J* = 8.71 Hz, 1H) 4.94 (d, *J* = 3.32 Hz, 1H) 6.43–6.49 (m, 2H) 7.20–7.29 (m, 5H) 7.29–7.35 (m, 1H) 7.36–7.44 (m, 3H) 7.55–7.62 (m, 3H) 7.68 (d, *J* = 7.46 Hz, 1H) 7.86 (d, *J* = 1.24 Hz, 2H) 8.13–8.18 (m, 2H). ^13^C NMR (101 MHz, DMSO-*d_6_*) 39.13–40.81, 48.39, 52.14, 123.39, 123.92, 124.88, 125.69, 127.46, 129.11, 129.44, 130.91, 132.03, 133.89, 137.73, 139.18, 140.10, 141.60, 142.26, 142.73, 175.14, 175.56, 189.95 (chalcone C=O) ppm. IR *ν* max (ATR): 3059.03 (Ar C-H), 1707.71 (C=O), 1673.57 (C=C), 1637.61 (Ar C=C), 1327.46 (C-N), 767.80 (C-Cl) cm^−1^. LRMS (APCI) 514.21 [M^+^−H]. HRMS (ESI) calculated for C_33_H_21_ClNO_3_ [M^+^−H]: 514.1209; found: 514.1191.

(*E*)-13-(4-Chlorophenyl)-9-(3-(4-iodophenyl)-3-oxoprop-1-en-1-yl)-9,10-dihydro-9,10-[3,4]epipyrroloanthracene-12,14-dione (**25g**) was synthesized (*E*)-3-(anthracen-9-yl)-1-(4-iodophenyl)prop-2-en-1-one (1.0 mmol, 0.4493 g) and 1-(4-chlorophenyl)-1*H*-pyrrole-2,5-dione (1.3 mmol, 0.27 g) according to the general procedure above; white powder (92%), Mp. 225–228 °C. ^1^H NMR (400 MHz, DMSO-*d_6_*) δ 3.52 (dd, *J* = 8.29, 3.32 Hz, H), 3.98 (d, *J* = 8.29 Hz, 2H), 4.93 (d, *J* = 3.32 Hz, 2H), 6.45 (d, *J* = 8.29 Hz, 2H), 7.16–7.23 (m, 2H), 7.23–7.28 (m, 2H), 7.29–7.34 (m, 1H), 7.36 (d, *J* = 3.73 Hz, H), 7.37–7.41 (m, H), 7.54–7.59 (m, 1H), 7.81 (d, *J* = 16.59 Hz, 1H), 7.84–7.92 (m, H), 7.97 (d, *J* = 8.29 Hz, 2H). IR *ν* max (ATR): 1716.83, 1661.60, 1493.51, 1392.83, 1325.76 cm^−1^. LRMS (APCI) [M^+^+H]: 642.04. HRMS (ESI) calculated for C_33_H_21_ClINNaO_3_ [M^+^−H]: 664.0147; found: 664.0133.

(*E*)-13-(4-Chlorophenyl)-9-(3-(4-fluorophenyl)-3-oxoprop-1-en-1-yl)-9,10-dihydro-9,10[3,4]epipyrroloanthracene-12,14-dione (**25h**) was synthesized from (*E*)-3-(anthracen-9-yl)-1-(4-fluorophenyl)prop-2-en-1-one (1.0 mmol, 0.326 g) and 1-(4-chlorophenyl)-1*H*-pyrrole-2,5-dione (1.3 mmol, 0.27 g) according to the general procedure above; white powder (88%) Mp. >250 °C. ^1^H NMR (400 MHz, DMSO-*d_6_*) δ 3.49 (dd, *J* = 8.50, 3.11 Hz, 4 H), 3.67 (d, *J* = 8.29 Hz, 5 H), 4.93 (d, *J* = 3.32 Hz, 1 H), 6.47 (d, *J* = 8.71 Hz, 2 H), 7.16–7.23 (m, 3 H), 7.23–7.31 (m, 6 H), 7.34–7.43 (m, 4 H), 7.47 (d, *J* = 6.63 Hz, 1 H), 7.83 (d, *J* = 15.76 Hz, 1 H), 8.00 (d, *J* = 15.76 Hz, 1 H), 8.21 (dd, *J* = 8.71, 5.39 Hz, 5 H). ^13^C NMR (101 MHz, DMSO-*d_6_*) 45.4, 48.4, 48.4, 52.1, 116.4, 116.6, 123.4, 123.9, 124.9, 125.7, 126.9, 127.4, 127.4, 127.5, 128.7, 129.5, 130.9, 131.9, 132.1, 132.2, 133.6, 134.4, 134.5, 139.2, 140.1, 141.6, 142.4, 142.7, 175.2, 175.5, 188.5 ppm. IR *ν* max (ATR): 1709.12, 1681.34, 1638.55, 1596.79, 1494.89, 1480.52, 1390.28, 1324.82 cm^−1^. HRMS (ESI) calculated for C_33_H_21_ClFNNaO_3_ [M^+^+Na]: 556.1086; found: 556.1081.

(*E*)-13-(4-Chlorophenyl)-9-(3-(4-methoxyphenyl)-3-oxoprop-1-en-1-yl)-9,10-dihydro-9,10-[3,4]epipyrroloanthracene-12,14-dione (**25i**) was synthesized from (*E*)-3-(anthracen-9-yl)-1-(4-methoxyphenyl)prop-2-en-1-one (1.0 mmol, 0.338 g) and 1-(4-chlorophenyl)-1*H*-pyrrole-2,5-dione (1.3 mmol, 0.27 g) according to the general procedure above; white powder (63%), Mp. 220–225 °C. ^1^H NMR (400 MHz, CDCl_3_) δ 3.53 (dd, *J* = 8.50, 3.11 Hz, 1H), 3.85 (s, 3H), 3.99 (d, *J* = 8.29 Hz, 1H), 4.94 (d, *J* = 3.32 Hz, 1H), 6.44–6.48 (m, 2H), 7.11 (d, *J* = 8.71 Hz, 2H), 7.19–7.34 (m, 6H), 7.35–7.40 (m, 1H), 7.39–7.43 (m, 2H), 7.59 (d, *J* = 7.05 Hz, 1H), 7.77–7.91 (m, 2H), 8.15 (d, *J* = 9.12 Hz, 2H). ^13^C NMR (101 MHz, CDCl_3_) ppm 39.21–40.74, 45.39, 48.40, 56.07, 123.39, 123.92, 124.86, 125.68, 126.88, 127.23–127.57, 128.72, 129.46, 131.53, 131.94, 140.21, 141.11, 142.86, 175.13, 175.56, 188.11 (chalcone C=O). IR *ν* max (ATR): 3344.59 (N-H), 1669.34 (C=O), 1599.27 (Ar C=C), 1230.74 (C-O), 1021.65 (C-N), 766.69 (C-Cl) cm^−1^. LRMS (APCI) 544.24 [M^+^−H]. HRMS (ESI) calculated for C_34_H_24_ClNNaO_4_ [M^+^+Na]: 568.1286; found 568.1283.

(*E*)-13-(4-Chlorophenyl)-9-(3-oxo-3-(*p*-tolyl)prop-1-en-1-yl)-9,10-dihydro-9,10-[3,4]epipyrroloanthracene-12,14-dione (**25j**) was synthesized from (*E*)-3-(anthracen-9-yl)-1-(*p*-tolyl)prop-2-en-1-one (1.0 mmol, 0.338 g) and 1-(4-chlorophenyl)-1*H*-pyrrole-2,5-dione (1.3 mmol, 0.27 g) according to the general procedure above; white powder (60%), Mp. 215–220 °C. ^1^H NMR (400 MHz, CDCl_3_) δ 2.39 (s, 3H), 3.53 (dd, *J* = 8.29, 3.32 Hz, 1H), 3.99 (d, *J* = 8.29 Hz, 1H), 4.94 (d, *J* = 2.90 Hz, 1H), 6.43–6.49 (m, 2H, 7.20–7.29 (m, 5H), 7.29–7.34 (m, 1H), 7.35–7.43 (m, 5H), 7.55–7.61 (m, 1H), 7.85 (d, *J* = 2.49 Hz, 2H), 8.06 (d, *J* = 7.88 Hz, 2H). ^13^C NMR (101 MHz, CDCl_3_) ppm 21.69, 39.15–40.74, 48.40, 52.13, 123.39, 123.92, 124.87, 125.72, 126.88, 127.18–127.58, 128.44–128.84, 129.05 -129.59, 129.95, 132.00, 135.24, 140.15, 141.53–141.91, 142.78, 175.13, 189.36 (chalcone C=O) IR *ν* max (ATR): 3340.19 (N-H), 2970.43 (C-H), 1709.33 (C=O), 1623.92 (Ar C=C), 1464.76 (C-C), 1224.81 (C-O), 1014.05 (C-N), 728.59 (C-Cl) cm^−1^. LRMS (APCI) 528.33 [M^+^−H]. HRMS (ESI) calculated for C_34_H_24_ClNNaO_3_ [M^+^+Na]: 552.1337; found 552.1337.

(*E*)-13-(4-Chlorophenyl)-9-(3-oxo-3-(3,4,5-trimethoxyphenyl)-prop-1-en-1-yl)-9,10-dihydro-9,10-[3,4]epipyrroloanthracene-12,14-dione (**25k**) was synthesized from (*E*)-3-(anthracen-9-yl)-1-(3,4,5-trimethoxyphenyl)prop-2-en-1-one (1.0 mmol, 0.398 g) and 1-(4-chlorophenyl)-1*H*-pyrrole-2,5-dione (1.3 mmol, 0.27 g) according to the general procedure above; white powder (35%), Mp. 160–163 °C. ^1^H NMR (400 MHz, CDCl_3_) δ 3.53 (dd, *J* = 8.29, 3.32 Hz, 1H), 3.76 (s, 3H), 3.85 (s, 6H), 4.00 (d, *J* = 8.29 Hz, 1H), 4.94 (d, *J* = 3.32 Hz, 1H), 6.45–6.49 (m, 2H), 7.20–7.31 (m, 5H), 7.31–7.43 (m, 4H), 7.47 (s, 2H), 7.57–7.61 (m, 1H), 7.80 (d, *J* = 16.0 Hz, 1H), 7.88 (d, *J* = 16.0 Hz, 1H). ^13^C NMR (101 MHz, CDCl_3_) ppm 45.41, 48.3, 48.6, 52.11, 56.54, 60.67, 106.87, 124.90, 125.68, 126.85, 127.24–127.60, 128.55, 129.30–129.66, 132.54, 141.50, 142.60, 142.78, 153.42, 175.19, 189.10 (chalcone C=O). IR *ν* max (ATR): 2940.25 (C-H), 1707.34 (C=O), 1579.08 (Ar C=C), 1234.05 (C-O), 1090.23 (C-N), 766.71 cm^−1^. LRMS (APCI): 606.21 [M^+^+H].

(*E*)-13-(4-Chlorophenyl)-9-(3-(4-chlorophenyl)-3-oxoprop-1-en-1-yl)-9,10-dihydro-9,10-[3,4]epipyrroloanthracene-12,14-dione (**25l**) was synthesized from (*E*)-3-(anthracen-9-yl)-1-(4-chlorophenyl)prop-2-en-1-one (1.0 mmol, 0.343 g) and 1-(4-chlorophenyl)-1*H*-pyrrole-2,5-dione (1.3 mmol, 0.27 g) according to the general procedure above; white powder (39%), Mp. 268–271 °C. ^1^H NMR (400 MHz, CDCl_3_) δ 3.53 (dd, *J* = 8.29, 3.32 Hz, 1H), 4.00 (d, *J* = 8.29 Hz, 1H), 4.94 (d, *J* = 3.32 Hz, 1H), 6.42–6.48 (m, 2H), 7.20–7.31 (m, 6H), 7.32 (s, H), 7.36–7.43 (m, 3H), 7.59 (d, *J* = 7.05 Hz, 1H), 7.65–7.70 (m, 2H), 7.87 (s, 2H), 8.14–8.19 (m, 2H). ^13^C NMR (101 MHz, CDCl_3_) ppm 39.07–40.76, 45.39, 48.39, 52.16, 123.42, 123.94, 124.89, 125.72, 127.46, 128.68, 129.32–129.73, 130.69–131.19, 131.78, 133.58, 138.84, 139.17, 140.04, 141.59, 142.74, 175.16, 175.55, 188.92 (chalcone C=O). IR *ν* max (ATR): 1707.42 (C=O), 1673.82 (C=C), 1589.01 (Ar C=C), 1015.71 (C-N), 746.57 cm^−1^. LRMS (APCI) 548.09 [M^+^−H]. HRMS Found 645.4544 [M^+^+H_3_Cl_2_Na}; C_33_H_24_Cl_4_NO_3_Na requires 645.0408.

(*E*)-13-(4-Chlorophenyl)-9-(3-oxo-3-(pyridin-4-yl)prop-1-en-1-yl)-9,10-dihydro-9,10-[3,4]epipyrroloanthracene-12,14-dione (**25m**) was synthesized from (*E*)-3-(anthracen-9-yl)-1-(pyridin-2-yl)prop-2-en-1-one (1.0 mmol, 0.309 g) and 1-(4-chlorophenyl)-1*H*-pyrrole-2,5-dione (1.3 mmol, 0.27 g) according to the general procedure (4), (92%), powder, Mp. 197–198 °C. IR *ν* max (ATR): 1703.19, 1493.90, 777.18 cm^−1^. ^1^H NMR (400 MHz, DMSO-*d_6_*) δ 3.52 (dd, *J* = 8.29, 3.32 Hz, 1H), 3.97 (d, *J* = 8.71 Hz, 1H), 4.93 (d, *J* = 2.90 Hz, 1H), 6.44 (d, *J* = 8.71 Hz, 2H), 7.24–7.29 (m, 4H), 7.30–7.35 (m, 1H), 7.35–7.42 (m, 3H), 7.54–7.60 (m, 2H), 7.78 (d, *J* = 16.17 Hz, 1H), 7.91 (d, *J* = 15.76 Hz, 1H), 7.97 (d, *J* = 6.63 Hz, 2H), 8.85 (d, *J* = 6.22 Hz, 2H). ^13^C NMR (101 MHz, CDCl_3_) 44.89. 47.79, 48.23, 51.82, 123.07, 123.53, 123.59, 124.47, 125.23, 126.40, 126.89, 126.97, 127.11, 128.22, 129.00, 130.39, 131.39, 133.12, 138.68, 139.29, 141.07, 141.88, 145.03, 147.47 ppm. HRMS (ESI) calculated for C_32_H_22_Cl_2_NO_3_ [M^+^+H]: 551.0935; found: 551.0924.

(*E*)-13-(4-Chlorophenyl)-9-(3-oxo-3-(pyridin-2-yl)prop-1-en-1-yl)-9,10-dihydro-9,10-[3,4]epipyrroloanthracene-12,14-dione (**25n**) was synthesized from (*E*)-3-(anthracen-9-yl)-1-(pyridin-2-yl)prop-2-en-1-one (1.0 mmol, 0.309 g) and 1-(4-chlorophenyl)-1*H*-pyrrole-2,5-dione (1.3 mmol, 0.27 g), according to the general procedure above; cream powder, 57%. IR *ν* max (ATR): 1710.53, 1677.50, 1624.02, 1490.52, 1388.35, 1324.42, 1457.44, 1190.22, 1085.27, 1015.50, 996.38 cm^−1^. ^1^H NMR (400 MHz, DMSO-*d_6_*) δ 3.50 (dd, *J* = 8.29, 3.32 Hz, 3H), 3.84 (d, *J* = 8.71 Hz, H), 4.94 (d, *J* = 2.90 Hz, 3 H), 6.45 (d, *J* = 8.29 Hz, H), 7.11–7.22 (m, 1 H), 7.22–7.30 (m, 4H), 7.30–7.35 (m, 1H), 7.36–7.42 (m, 3H), 7.59 (d, *J* = 7.05 Hz, 1H), 7.70 (dd, *J* = 7.46, 4.98 Hz, 1H), 8.04 (d, *J* = 17.00 Hz, 1H), 8.07–8.12 (m, 1H), 8.21 (d, *J* = 7.88 Hz, 1H), 8.27 (d, *J* = 16.59 Hz, 1H), 8.76 (d, *J* = 4.98 Hz, 1H). ^13^C NMR (101 MHz, DMSO-*d_6_*) 45.41, 48.40, 48.96, 52.25, 127.37, 127.41, 127.54, 128.31, 128.64, 129.4, 130.28, 130.89, 133.51, 138.34, 139.29, 140.07, 141.66, 142.60, 143.09, 149.75, 153.68, 174.99, 175.58, 188.85 ppm. HRMS (ESI) calculated for C_23_H_22_ClN_2_O_3_ [M^+^+H]: 517.1313; found: 517.1032.

(*E*)-13-(4-Chlorophenyl)-9-(3-(furan-2-yl)-3-oxoprop-1-en-1-yl)-9,10-dihydro-9,10-[3,4]epipyrroloanthracene-12,14-dione (**25o**) was prepared from (*E*)-3-(anthracen-9-yl)-1-(furan-2-yl)prop-2-en-1-one (1.0 mmol, 0.298 g) and 1-(4-chlorophenyl)-1*H*-pyrrole-2,5-dione (1.3 mmol, 0.27 g) according to the general procedure above; white powder (46%) Mp. 256 °C (dec). ^1^H NMR (400 MHz, CDCl_3_) δ 3.54 (dd, *J* = 8.33, 3.18 Hz, 1H), 4.03 (d, *J* = 8.21 Hz, 1H), 4.94 (d, *J* = 3.3 Hz, 1H), 6.61 (m, 2H), 7.12–7.32 (m, 6H), 7.35–7.43 (m, 2H), 7.45–7.49 (m, 2H), 7.55–7.63 (m, 2H), 7.66 (m, 1H), 7.75 (d, *J* = 16.18 Hz, 1H), 8.13 (d, *J* = 16.18 Hz, 1H). ^13^C NMR (101 MHz, CDCl_3_) ppm 45.4, 48.3, 48.8, 52.0, 113.4, 120.6, 123.4, 123.9, 124.9, 125.7, 125.7, 126.9, 127.4, 127.5, 128.6, 129.3, 129.4, 130.9, 131.4, 133.5, 139.2, 140.0, 141.5, 141.6, 142.6, 149.2, 153.1, 167.3, 175.1, 175.5, 176.8. IR *ν* max (ATR): 1709.72, 1675.02, 1626.02, 1586.77, 1494.51, 1480.32, 1388.67 cm^−1^. LRMS (APCI): 506.14 [M^+^+H].

(*E*)-13-(4-Chlorophenyl)-9-(3-(naphthalen-2-yl)-3-oxoprop-1-en-1-yl)-9,10-dihydro-9,10-[3,4]epipyrroloanthracene-12,14-dione (**25p**) was prepared from (*E*)-3-(anthracen-9-yl)-1-(naphthalen-2-yl)prop-2-en-1-one (1.0 mmol, 0.358 g) and 1-(4-chlorophenyl)-1*H*-pyrrole-2,5-dione (1.3 mmol, 0.27 g) according to the general procedure above; gray powder (88%), Mp. 243–244 °C. ^1^H NMR (400 MHz, CDCl_3_) δ 3.51 (dd, *J* = 8.50, 3.11 Hz, 3H), 3.72 (d, *J* = 8.29 Hz, 3H), 4.95 (d, *J* = 3.32 Hz, 1H), 6.51 (d, *J* = 8.71 Hz, 2H), 7.24–7.32 (m, 6H), 7.38–7.43 (m, 2H), 7.43–7.50 (m, 2H), 7.52–7.58 (m, 1H), 7.58–7.64 (m, 1H), 7.91 (d, *J* = 7.88 Hz, 1H), 7.94–8.02 (m, H), 8.05–8.11 (m, 1H), 8.23 (dd, *J* = 8.50, 1.45 Hz, 1H), 8.71 (s, 1H). ^13^C NMR (101 MHz, CDCl_3_) ppm 46.00, 48.1, 48.47, 52.5, 123.63, 124.08, 124.16, 124.57, 125.50, 126.78, 126.89, 127.40, 127.54, 127.70, 127.82, 128.57, 128.71, 129.31, 129.48, 129.71, 129.74, 130.81, 131.62, 132.59, 134.74, 135.05, 135.68, 138.16, 140.69, 142.05, 142.19, 174.34, 175.15, 189.99. IR *ν* max (ATR): 1709.81, 1668.28, 1645.28, 1584.47, 1459.45, 1420.04, 1389.07, 1493.53, 1442.07 cm^−1^. LRMS (APCI): [M^+^+Cl] 602.31. HRMS calculated for C_37_H_24_Cl_2_NO_3_ [M+Cl-2H] 598.0977; found 598.1371.

(*E*)-13-(4-Chlorophenyl)-9-(3-oxo-3-(thiophen-2-yl)prop-1-en-1-yl)-9,10-dihydro-9,10-[3,4]epipyrroloanthracene-12,14-dione (**25q**) was prepared from (*E*)-3-(anthracen-9-yl)-1-(thiophen-2-yl)prop-2-en-1-one (1.0 mmol, 0.314 g) and 1-(4-chlorophenyl)-1*H*-pyrrole-2,5-dione (1.3 mmol, 0.27 g) according to the general procedure above; white powder (70%), Mp. 180–181 °C. ^1^H NMR (400 MHz, DMSO-*d_6_*) δ 3.56 (dd, *J* = 8.29, 3.32 Hz, 1H), 3.99 (d, *J* = 8.29 Hz, 1H), 4.95 (d, *J* = 2.90 Hz, 1H), 6.48 (d, *J* = 8.71 Hz, 2H), 7.09–7.17 (m, 2H), 7.18–7.32 (m, H), 7.32–7.36 (m, 1 H), 7.36–7.43 (m, 3H), 7.59 (d, *J* = 6.63 Hz, 1H), 7.84 (d, *J* = 15.76 Hz, H), 7.93 (d, *J* = 16.59 Hz, 1H), 8.10 (d, *J* = 4.56 Hz, 1H), 8.21 (d, *J* = 3.32 Hz, 1H). ^13^C NMR (101 MHz, DMSO-*d_6_*) 45.45, 48.35, 48.68, 52.08, 123.42, 123.9, 124.88, 125.7, 125.74, 126.88, 127.3, 127.5, 129.33, 129.44, 129.4, 130.90, 131.70, 133.58, 134.6, 136.50, 137.79, 139.18, 140.05, 141.60, 142.67, 145.27 ppm. IR *ν* max (ATR): 1733.84, 1708.84, 1699.66, 1662.25, 1616.18, 1582.06, 1546.74, 1562.06 cm^−1^. LRMS (APCI): 520.30 [M^+^−H]. HRMS (APCI) calculated for C_31_H_20_ClNNaO_3_S [M^+^+Na]: 544.0745; found: 544.0735.

### 3.7. General Preparation for (E)-13-(4-Benzoylphenyl)-9-(3-oxo-3-phenylprop-1-en-1-yl)-9,10-dihydro-9,10-[3,4] Epipyrroloanthracene-12,14-diones ***26a–q***

To a solution of the appropriate chalcone anthracene **21a–q** (1.0 mmol) in toluene (2 mL) was added dienophile 1-(4-benzoylphenyl)-1*H*-pyrrole-2,5-dione (1.3 mmol). The mixture was heated with stirring at 90 °C for 48 h. The reaction mixture was then cooled to room temperature, and the resulting crude solid product was isolated via filtration. The product was washed with toluene (2 mL) and diethyl ether (2 mL) and then recrystallized from toluene.

(*E*)-13-(4-Benzoylphenyl)-9-(3-(4-bromophenyl)-3-oxoprop-1-en-1-yl)-9,10-dihydro-9,10-[3,4]epipyrroloanthracene-12,14-dione (**26a**) was synthesized from (*E*)-3-(anthracen-9-yl)-1-(4-bromophenyl)prop-2-en-1-one (1.0 mmol, 0.387 g) and 1-(4-benzoylphenyl)-1*H*-pyrrole-2,5-dione (1.3 mmol, 0.361 g) according to the general procedure above; white powder (71%), Mp. 209–210 °C. (HPLC: 93.25%, RT 2.57 min). ^1^H NMR (400 MHz, DMSO-*d_6_*) δ 3.57 (dd, *J* = 8.29, 2.90 Hz, 3H), 4.03 (d, *J* = 8.71 Hz, 3H), 4.96 (d, *J* = 2.90 Hz, 3H), 6.65 (d, *J* = 8.29 Hz, 6H), 7.20–7.30 (m, 15H), 7.32–7.36 (m, 3H), 7.36–7.41 (m, 3H), 7.48–7.54 (m, 6H), 7.56–7.61 (m, 3H), 7.61–7.69 (m, 15H), 7.80 (d, *J* = 8.71 Hz, 6H), 7.83–7.87 (m, 2H), 7.88–7.93 (m, 1H), 8.08 (d, *J* = 8.71 Hz, 2H). ^13^C NMR (101 MHz, DMSO-*d_6_*) 45.4, 48.4, 48.5, 52.2, 123.4, 124.0, 124.9, 125.7, 126.9, 127.0, 127.4, 127.5, 127.6, 128.0, 129.1, 130.1, 130.6, 131.1, 131.8, 132.5, 133.4, 135.5, 136.7, 137.0, 137.4, 139.2, 140.0, 141.6, 142.7, 142.8, 175.1, 175.5, 189.1, 195.3 ppm. LRMS (APCI) [M^+^+H]: 664.16.

(*E*)-13-(4-Benzoylphenyl)-9-(3-(4-nitrophenyl)-3-oxoprop-1-en-1-yl)-9,10-dihydro-9,10-[3,4]epipyrroloanthracene-12,14-dione (**26b**) was prepared from (*E*)-3-(anthracen-9-yl)-1-(4-nitrophenyl)prop-2-en-1-one (1.0 mmol, 0.353 g) and 1-(4-benzoylphenyl)-1*H*-pyrrole-2,5-dione (1.3 mmol, 0.361 g) according to the general procedure above; white powder (75%); Mp. 175–177 °C. ^1^H NMR (400 MHz, DMSO-*d_6_*) δ 3.59 (dd, *J* = 8.50, 3.11 Hz, 2H), 4.04 (d, *J* = 8.71 Hz, 2H), 4.98 (d, *J* = 3.32 Hz, 2H), 6.66 (d, *J* = 8.29 Hz, 3H), 7.09–7.17 (m, 4H), 7.19–7.26 (m, 5H), 7.26–7.32 (m, 6H), 7.34–7.42 (m, 4H), 7.50–7.55 (m, 4H), 7.58–7.64 (m, 2H), 7.65–7.70 (m, 8H), 7.92 (dd, *J* = 16.60 Hz, 2H), 8.34–8.43 (m, 4H). ^13^C NMR (101 MHz, DMSO-*d_6_*) 45.45, 48.43, 48.66, 52.30, 123.51, 124.02, 124.49, 124.94, 125.75, 126.91, 127.01, 127.48, 127.63, 128.6, 129.07, 129.34, 130.08, 130.50, 130.61, 132.03, 133.36, 135.54, 136.98, 137.40, 137.79, 139.17, 139.91, 141.60, 142.52, 142.54, 144.14, 150.47, 175.15, 175.51, 189.23 (chalcone C=O), 195.28 (benzophenone C=O) ppm. IR *ν* max (ATR): 1714.57, 1687.42, 1639.11, 1653.38, 1604.55, 1557.12, 1506.50, 1449.13, 1409.43, 1377.97, 1275.37, 1173.59 cm^−1^. LRMS (APCI): 631.19 [M^+^+H]. HRMS (APCI) calculated for C_40_H_26_KN_2_O_6_ [M^+^+K]: 669.1423; found: 669.1418.

(*E*)-13-(4-Benzoylphenyl)-9-(3-(4-ethylphenyl)-3-oxoprop-1-en-1-yl)-9,10-dihydro-9,10-[3,4]epipyrroloanthracene-12,14-dione (**26c**) was prepared from (*E*)-3-(anthracen-9-yl)-1-(4-ethylphenyl)prop-2-en-1-one (1.0 mmol, 0.336 g) and 1-(4-benzoylphenyl)-1*H*-pyrrole-2,5-dione (1.3 mmol, 0.361 g) according to the general procedure above; white powder (76%), Mp. 246–247 °C. ^1^H NMR (400 MHz, DMSO-*d_6_*) δ 3.58 (dd, *J* = 8.50, 3.11 Hz, 1H), 3.84 (s, 2H), 3.85 (s, 3H), 4.04 (d, *J* = 8.71 Hz, 1H), 4.97 (d, *J* = 3.32 Hz, 1H), 6.67 (d, *J* = 8.29 Hz, 2H), 7.13 (d, *J* = 8.29 Hz, H), 7.19–7.32 (m, 5), 7.32–7.36 (m, 1H), 7.37–7.42 (m, 1H), 7.49–7.56 (m, 2H), 7.60 (d, *J* = 6.63 Hz, 1H), 7.63–7.72 (m, 5H), 7.81 (d, *J* = 16.17 Hz, 1H), 7.78–7.84 (m, 1H), 7.87 (dd, *J* = 8.71, 2.07 Hz, 1H), 7.91 (d, *J* = 16.17 Hz, 1H). ^13^C NMR (101 MHz, DMSO-*d_6_*) 45.43,48.47, 48.55, 52.14, 56.01, 56.29, 111.31, 111.51,123.39, 123.93, 124.08, 124.89, 125.71, 126.88, 126.97, 127.37, 127.45, 127.55, 129.08, 130.08, 130.60, 132.11, 133.36, 135.56, 136.97, 137.35, 139.20, 140.21, 141.65, 149.34, 175.10, 175.52, 188.25 (chalcone C=O), 195.30 (benzophenone C=O) ppm. IR *ν* max (ATR): 1716.45, 1660.71, 1391.17, 1325.22 cm^−1^. LRMS (APCI) 614.14 [M^+^+H].

(*E*)-13-(4-Benzoylphenyl)-9-(3-(2,4-dichlorophenyl)-3-oxoprop-1-en-1-yl)-9,10-dihydro-9,10-[3,4]epipyrroloanthracene-12,14-dione (**26d**) was prepared from (*E*)-3-(anthracen-9-yl)-1-(2,4-dichlorophenyl)prop-2-en-1-one (1.0 mmol, 0.377 g) and 1-(4-benzoylphenyl)-1*H*-pyrrole-2,5-dione (1.3 mmol, 0.361 g) according to the general procedure above; white powder (63%). ^1^H NMR (400 MHz, DMSO-*d_6_*) δ 3.58 (dd, *J* = 8.29, 3.32 Hz, 1H), 4.04 (d, *J* = 8.29 Hz, 1H), 4.97 (d, *J* = 2.90 Hz, 1H), 6.67 (d, *J* = 8.29 Hz, 2H), 7.09–7.16 (m, 1H), 7.19–7.32 (m, 5H), 7.32–7.36 (m, 1H), 7.37–7.43 (m, 1H), 7.50–7.56 (m, 2H), 7.60 (d, *J* = 6.22 Hz, 1H), 7.63–7.71 (m, 6H), 7.81 (d, *J* = 16.17 Hz, 1H), 7.86 (d, *J* = 1.66 Hz, 1H), 7.91 (d, *J* = 16.17 Hz, 1H). ^13^C NMR (101 MHz, DMSO-*d_6_*) 48.5, 52.1, 56.0, 56.3, 111.3, 111.5, 123.9, 124.1, 124.9, 126.9, 127.0, 127.5, 129.1, 130.1, 130.6, 132.1, 133.4, 135.6, 137.0, 137.4, 139.2, 140.2, 140.9, 141.7, 142.9, 144.5, 145.7, 149.3, 153.9, 175.1, 175.5, 188.2 (chalcone C=O), 195.3 (benzophenone C=O) ppm. LRMS (APCI): 614.15 [M^+^-C_3_H_3_].

(*E*)-13-(4-Benzoylphenyl)-9-(3-(3,4-dimethoxyphenyl)-3-oxoprop-1-en-1-yl)-9,10-dihydro-9,10-[3,4]epipyrroloanthracene-12,14-dione (**26e**) was prepared from (*E*)-3-(anthracen-9-yl)-1-(3,4-dimethoxyphenyl)prop-2-en-1-one (1.0 mmol, 0.368 g) and 1-(4-benzoylphenyl)-1*H*-pyrrole-2,5-dione (1.3 mmol, 0.361 g) according to the general procedure above; white powder (72%), Mp. 161–164 °C. IR *ν* max (ATR): 2969, 1714.39, 1651.24, 1603.13, 1507.90, 1458.19, 1378.67, 1275.61, 1177.36, 929.79, 925.10 cm^−1^. ^1^H NMR (400 MHz, DMSO-*d_6_*) δ 1.20 (t, *J* = 7.46 Hz, 2H), 2.28 (s, 2H), 3.58 (dd, *J* = 8.50, 3.11 Hz, 1H), 4.03 (d, *J* = 8.29 Hz, 1H), 4.97 (d, *J* = 3.32 Hz, 1H), 6.67 (d, *J* = 8.29 Hz, 1H), 7.09–7.18 (m, 2H), 7.19–7.25 (m, 3H), 7.26–7.32 (m, 2H), 7.32–7.36 (m, H), 7.38–7.45 (m, 2H), 7.50–7.55 (m, 2H), 7.58–7.63 (m, 1H), 7.63–7.71 (m, 4H), 7.82–7.93 (m, 2H), 7.87 (dd, *J* = 16.20 Hz, 2H), 8.10 (d, *J* = 7.88 Hz, 1H). ^13^C NMR (101 MHz, DMSO-*d_6_*) 21.49, 28.70, 45.4, 48.47, 48.52, 52.18, 123.41, 123.93, 124.89, 126.90, 127.02, 127.38, 127.48, 127.57, 128.64, 128.80, 129.07, 129.34, 129.39, 130.08, 130.60, 132.09, 133.36, 135.50, 135.56, 136.97, 137.38, 137.78, 139.18, 140.14, 141.63, 141.76, 142.80, 150.41, 175.09, 175.51, 189.38 (chalcone C=O), 195.29 (benzophenone C=O) ppm. LRMS (APCI) 614.19 [M^+^-OCH_3_].

(*E*)-13-(4-Benzoylphenyl)-9-(3-oxo-3-phenylprop-1-en-1-yl)-9,10-dihydro-9,10-[3,4]epipyrroloanthracene-12,14-dione (**26f**) was synthesized from (*E*)-3-(anthracen-9-yl)-1-phenylprop-2-en-1-one (1.0 mmol, 0.308 g) and 1-(4-benzoylphenyl)-1*H*-pyrrole-2,5-dione (1.3 mmol, 0.361 g) according to the general procedure above; white powder (49%), Mp. 240–241 °C. ^1^H NMR (400 MHz, DMSO-*d_6_*) δ 3.58 (dd, *J* = 8.50, 3.11 Hz, 1H), 4.04 (d, *J* = 8.29 Hz, 1H), 4.97 (d, *J* = 2.90 Hz, 1H), 6.67 (d, *J* = 8.29 Hz, 2H), 7.19–7.32 (m, 6H), 7.34 (s, 1H), 7.37–7.42 (m, 1H), 7.49–7.56 (m, 2H), 7.57–7.64 (m, 4H), 7.64–7.72 (m, 6H), 7.89 (s, 2H), 8.16 (d, *J* = 7.05 Hz, 2H). ^13^C NMR (101 MHz, DMSO-*d_6_*) 39.21–40.63, 48.48, 52.20, 55.34, 125.75, 127.03, 127.48, 128.94–129.60, 130.08, 130.61, 131.99, 136.97, 137.39, 139.17, 142.75, 174.93–175.59, 176.01, 189.96 (chalcone C=O), 195.31 (benzophenone C=O) ppm. IR *ν* max (ATR): 1708.57 (C=O), 1670.41 (C=C), 1603.92 (Ar C=C), 1308.22 (C-N) cm^−1^. HRMS (APCI) calculated for C_40_H_27_NNaO_4_ [M^+^+Na]: 608.1832; found: 608.1830.

(*E*)-13-(4-Benzoylphenyl)-9-(3-(4-iodophenyl)-3-oxoprop-1-en-1-yl)-9,10-dihydro-9,10-[3,4]epipyrroloanthracene-12,14-dione (**26g**) was prepared from (*E*)-3-(anthracen-9-yl)-1-(4-iodophenyl)prop-2-en-1-one (1.0 mmol, 0.338 g) and 1-(4-benzoylphenyl)-1*H*-pyrrole-2,5-dione (1.3 mmol, 0.361 g) according to the general procedure above; white powder (85%), Mp. 186–188 °C. ^1^H NMR (400 MHz, DMSO-*d_6_*) δ 3.59 (dd, *J* = 8.29, 2.90 Hz, 1H), 4.04 (d, *J* = 8.71 Hz, 1H), 4.98 (d, *J* = 3.32 Hz, 1H), 6.67 (d, *J* = 8.29 Hz, 2H), 7.10–7.17 (m, 2H), 7.19–7.32 (m, 6H), 7.33–7.37 (m, 1H), 7.38–7.43 (m, 1H), 7.50–7.56 (m, 2H), 7.61 (d, *J* = 7.05 Hz, 1H), 7.63–7.71 (m, 5H), 7.86 (d, *J* = 16.59 Hz, 1H), 7.89–7.95 (m, 3H), 7.96–8.03 (m, 2H). ^13^C NMR (101 MHz, DMSO-*d_6_*) 45.45, 48.4, 48.55, 52.23, 102.63, 123.45, 123.97, 124.90, 125.72, 125.7, 126.91, 127.03, 127.40, 127.48, 127.59, 128.64, 129.07, 129.34, 130.09, 130.61, 130.82, 131.76, 133.35, 135.56, 136.98, 137.04, 137.39, 137.79, 138.36, 139.17, 140.04, 141.61, 142.69, 142.77, 175.11, 175.50, 189.44, 195.28 ppm. IR *ν* max (ATR): 1715.20, 1687.34, 1638.37, 1653.19, 1604.04, 1448.80, 1379.55, 1275.10, 1173.70, 1190.33 cm^−1^. LRMS (APCI) 710.13 [M^+^−H]. HRMS (ESI) calculated for C_40_H_25_INO_4_ [M^+^−H]: 710.0834; found: 710.0823.

(*E*)-13-(4-Benzoylphenyl)-9-(3-(4-fluorophenyl)-3-oxoprop-1-en-1-yl)-9,10-dihydro-9,10-[3,4]epipyrroloanthracene-12,14-dione (**26h**) was synthesized from (*E*)-3-(anthracen-9-yl)-1-(4-fluorophenyl)prop-2-en-1-one (1.0 mmol, 0.387 g) and 1-(4-benzoylphenyl)-1*H*-pyrrole-2,5-dione (1.3 mmol, 0.361 g) according to the general procedure above, white powder (77%). ^1^H NMR (400 MHz, DMSO-*d_6_*) δ 3.51 (dd, *J* = 8.22, 3.17 Hz, 1H), 4.02 (d, *J* = 8.4 Hz, 1H), 4.98 (d, *J* = 3.28 Hz, 1H), 6.49–6.54 (m, 2H), 7.15–7.32 (m, 5H), 7.35 (m, 1H), 7.36–7.40 (m, 4H), 7.41–7.46 (m, 4H), 7.47–7.49 (m, 1H), 7.83 (m, 3H), 8.02 (d, *J* = 16.18 Hz, 1H), 8.13–8.23 (m, 2H).

(*E*)-13-(4-Benzoylphenyl)-9-(3-(4-methoxyphenyl)-3-oxoprop-1-en-1-yl)-9,10-dihydro-9,10-[3,4]epipyrroloanthracene-12,14-dione (**26i**) was prepared from (*E*)-3-(anthracen-9-yl)-1-(4-methoxyphenyl)prop-2-en-1-one (1.0 mmol, 0.338 g) and 1-(4-benzoylphenyl)-1*H*-pyrrole-2,5-dione (1.3 mmol, 0.361 g) according to the general procedure above; white powder (50%), Mp. 224–228 °C. ^1^H NMR (400 MHz, CDCl_3_) δ 3.58 (dd, *J* = 8.29, 3.32 Hz, 1H), 3.86 (s, 3H), 4.04 (d, *J* = 8.29 Hz, 1H), 4.97 (d, *J* = 3.32 Hz, H), 6.66 (d, *J* = 8.29 Hz, 2H), 7.12 (d, *J* = 8.71 Hz, 2H), 7.20–7.31 (m, 5H), 7.31–7.35 (m, 1H), 7.38–7.41 (m, 1H), 7.51–7.56 (m, 2H), 7.60 (d, *J* = 6.63 Hz, 1H), 7.64–7.71 (m, 5H), 7.79–7.93 (m, 2H), 8.16 (d, *J* = 8.71 Hz, 2H). ^13^C NMR (101 MHz, DMSO-*d_6_*) 39.26, 40.72, 48.48, 52.15, 56.07, 114.67, 123.42, 123.93, 124.87, 125.71, 126.76, 127.69, 130.08, 131.53, 133.36, 136.97, 139.18, 141.10, 141.64, 142.88, 163.89, 175.09, 175.52, 188.12 (chalcone C=O), 195.29 (benzophenone C=O) ppm. IR *ν* max (ATR): 3344.36 (N-H), 1659.32 (C=O), 1599.79 (Ar C=C), 1231.15 (C-O), 1020.42 (C-N) cm^−1^. HRMS (ESI) calculated for C_41_H_29_NNaO_5_ [M^+^+Na]: 638.1938; found: 638.1943.

(*E*)-13-(4-Benzoylphenyl)-9-(3-oxo-3-(*p*-tolyl)prop-1-en-1-yl)-9,10-dihydro-9,10-[3,4]epipyrroloanthracene-12,14-dione (**26j**) was prepared from (*E*)-3-(anthracen-9-yl)-1-(*p*-tolyl)prop-2-en-1-one (1.0 mmol, 0.3384 g) and 1-(4-benzoylphenyl)-1*H*-pyrrole-2,5-dione (1.3 mmol, 0.361g) according to the general procedure above; white powder (59%), Mp. 256–260 °C. ^1^H NMR (400 MHz, CDCl_3_) δ 2.40 (s, 3H), 3.57 (dd, *J* = 8.29, 2.90 Hz, 1H), 4.03 (d, *J* = 8.29 Hz, 1H), 4.96 (d, *J* = 2.90 Hz, 1H), 6.66 (d, *J* = 8.29 Hz, 2H), 7.20–7.31 (m, 5H), 7.31–7.36 (m, 1H), 7.40 (d, *J* = 7.88 Hz, 3H), 7.50–7.56 (m, 2H), 7.56–7.71 (m, 6H), 7.86 (d, *J* = 3.32 Hz, 2H), 8.06 (d, *J* = 7.88 Hz, 2H). ^13^C NMR (101 MHz, CDCl_3_) 21.69, 39.21–40.78, 48.49, 55.35, 123.41, 127.03, 128.83–129.51, 130.02, 136.97, 137.38, 139.18, 142.80, 144.38, 175.51 (chalcone C=O), 189.37 (benzophenone C=O) ppm. IR *ν* max (ATR): 2965 (O-H), 1712.89 (C=O), 1626.70 (Ar C=C), 1458.41 (C-C), 1231.54 (C-O), 1128.77 (C-N) cm^−1^. HRMS (ESI) calculated for C_41_H_29_NNaO_4_ [M^+^+Na]: 622.1989; found: 622.1982.

(*E*)-13-(4-Benzoylphenyl)-9-(3-oxo-3-(3,4,5-trimethoxyphenyl)-prop-1-en-1-yl)-9,10-dihydro-9,10-[3,4]epipyrroloanthracene-12,14-dione (**26k**) was prepared from (*E*)-3-(anthracen-9-yl)-1-(3,4,5-trimethoxyphenyl)prop-2-en-1-one (1 mmol, 0.398 g) and 1-(4-benzoylphenyl)-1*H*-pyrrole-2,5-dione (1.3 mmol, 0.361 g) according to the general procedure above; white powder (44%), Mp. 210–216 °C. ^1^H NMR (400 MHz, CDCl_3_) δ 3.58 (dd, *J* = 8.50, 3.11 Hz, 1H), 3.76 (s, 3H), 3.85 (s, 6 H), 4.04 (d, *J* = 8.71 Hz, 1H), 4.97 (d, *J* = 3.32 Hz, 1H), 6.65–6.70 (m, 2H), 7.21–7.31 (m, 5H), 7.35–7.38 (m, 1H), 7.38–7.42 (m, 1H), 7.48 (s, 2H), 7.51–7.56 (m, 2H), 7.59–7.62 (m, 1H), 7.63–7.70 (m, 5H), 7.80–7.93 (m, 2H). ^13^C NMR (101 MHz, CDCl_3_) 39.24, 40.78, 48.40, 52.16, 56.56, 60.68, 106.89, 125.53, 125.91, 126.85, 127.40, 128.64, 129.07, 129.33, 130.07, 130.56, 132.54, 133.05, 133.34, 137.00, 140.12, 141.56, 153.43, 175.03, 175.61, 189.07 (chalcone C=O), 195.29 (benzophenone C=O) ppm. IR *ν* max (ATR): 2941.10 (O-H), 1708.03 (C=O), 1579.54 (Ar C=C), 1234.15 (C-O), 1091.54 (C-N) cm^−1^. HRMS (ESI) calculated for C_43_H_33_NNaO_7_ [M^+^+Na]: 698.2149; found: 698.2141.

(*E*)-13-(4-Benzoylphenyl)-9-(3-(4-chlorophenyl)-3-oxoprop-1-en-1-yl)-9,10-dihydro-9,10-[3,4]epipyrroloanthracene-12,14-dione (**26l**) was synthesized from (*E*)-3-(anthracen-9-yl)-1-(4-chorophenyl)prop-2-en-1-one (1.0 mmol, 0.343 g) and 1-(4-benzoylphenyl)-1*H*-pyrrole-2,5-dione (1.3 mmol, 0.361 g) according to the general procedure above; white powder (30%), Mp. 217–220 °C. ^1^H NMR (400 MHz, DMSO-*d_6_*) δ 3.59 (dd, *J* = 3.32, 8.29 Hz, 1H), 4.04 (d, *J* = 8.29 Hz, 1H), 4.97 (d, *J* = 2.90 Hz, 1H), 6.66 (d, *J* = 8.71 Hz, 2H), 7.20–7.31 (m, 6H), 7.34 (m, 1H), 7.39 (m, 1H), 7.50–7.56 (m, 2H), 7.60 (d, *J* = 6.63 Hz, 1H), 7.66 (m, 6H), 7.89 (m, 2H), 8.18 (d, *J* = 8.0 Hz, 2H) ^13^C NMR (101 MHz, DMSO-*d_6_*) 42.31, 45.42, 48.48, 52.20, 123.44, 123.95, 124.90, 125.64, 125.78, 126.85, 127.14, 127.35, 127.66, 128.64, 129.08, 129.55, 130.08, 130.61, 131.03, 131.81, 136.41, 138.85, 139.16, 142.68, 142.81, 175.12, 175.51, 195.31 (chalcone C=O), 197.42 (benzophenone C=O) ppm. IR *ν* max (ATR): 3024.65 (Ar C-H), 1712.90 (C=O), 1672.35 (C=C), 1603.46 (Ar C=C), 1013.52 (C-N), 769.64 cm^−1^. HRMS (ESI) calculated for C_40_H_25_ClNO_4_ [M^+^−H]: 618.1478; found: 618.1487.

(*E*)-13-(4-Benzoylphenyl)-9-(3-oxo-3-(pyridin-4-yl)prop-1-en-1-yl)-9,10-dihydro-9,10-[3,4]epipyrroloanthracene-12,14-dione (**26m**) was synthesized from (*E*)-3-(anthracen-9-yl)-1-(pyridin-4-yl)prop-2-en-1-one (1.0 mmol, 0.309 g) and 1-(4-benzoylphenyl)-1*H*-pyrrole-2,5-dione (1.3 mmol, 0.361 g) according to the general procedure above; white powder (62%), Mp. 230–231 °C. ^1^H NMR (400 MHz, DMSO-*d_6_*) δ 3.58 (dd, *J* = 8.29, 3.32 Hz, 2H), 4.02 (d, *J* = 8.29 Hz, 2H), 4.97 (d, *J* = 3.32 Hz, 2H), 6.66 (d, *J* = 8.71 Hz, 4H), 7.19–7.32 (m, 10H), 7.33–7.38 (m, 2 H), 7.38–7.42 (m, 2 H), 7.49–7.56 (m, 4H), 7.59–7.64 (m, 3H), 7.64–7.69 (m, 9H), 7.78–7.85 (m, 2H), 7.81 (d, *J* = 16.17 Hz, 1H), 7.91–7.97 (m, 2H), 7.94 (d, *J* = 16.17 Hz, 3H), 7.99 (d, *J* = 5.81 Hz, 4H). ^13^C NMR (101 MHz, DMSO-*d_6_*) 45.41, 48.40, 48.67, 52.27, 122.20, 123.48, 123.99, 124.94, 125.73, 126.93, 127.02, 127.48, 127.63, 129.08, 130.08, 130.61, 131.80, 133.38, 135.51, 136.96, 137.40, 139.14, 139.85, 141.57, 142.48, 143.77, 144.35, 151.35, 175.14, 175.51, 190.04 (chalcone C=O), 195.32 (benzophenone C=O) ppm. IR *ν* max (ATR): 1714.95, 1687.33, 1654.19, 1604.43, 1449.30, 1377.44, 1275.15, 1173.54, 1190.08 cm^−1^. LRMS (APCI) 587.37 [M^+^+H]. HRMS (ESI) calculated for C_39_H_25_N_2_O_4_ [M^+^−H]: 585.1820; found: 585.1818.

(*E*)-13-(4-Benzoylphenyl)-9-(3-oxo-3-(pyridin-2-yl)prop-1-en-1-yl)-9,10-dihydro-9,10-[3,4]epipyrroloanthracene-12,14-dione (**26n**) was prepared from (*E*)-3-(anthracen-9-yl)-1-(pyridin-2-yl)prop-2-en-1-one (1.0 mmol, 0.309 g) and 1-(4-benzoylphenyl)-1*H*-pyrrole-2,5-dione (1.3 mmol, 0.361 g) according to the general procedure above; gray powder (75%), Mp. > 240 °C (dec). HPLC: 96.29%, RT 8.88 min). ^1^H NMR (400 MHz, DMSO-*d_6_*) δ 3.54 (dd, *J* = 4.98, 3.32 Hz, 1H), 3.86–3.91 (m, 1H), 4.96 (d, *J* = 3.32 Hz, 1H), 6.66 (d, *J* = 8.71 Hz, 2H), 7.19–7.31 (m, 6H), 7.32–7.38 (m, 1H), 7.38–7.42 (m, 1H), 7.52 (s, 2H), 7.58–7.64 (m, 2H), 7.64–7.73 (m, H), 8.08 (d, *J* = 16.59 Hz, 1H), 8.09–8.12 (m, H), 8.20–8.23 (m, 1H), 8.27 (d, *J* = 16.59 Hz, 1H). ^13^C NMR (101 MHz, DMSO-*d_6_*) 45.44, 48.50, 49.04, 52.29, 123.30, 123.42, 123.89, 124.96, 125.75, 126.89, 126.95, 127.44, 128.31, 128.64, 129.07, 129.33, 130.06, 130.32, 130.56, 133.36, 135.53, 136.97, 137.33, 137.79, 138.34, 139.23, 140.06, 141.67, 142.62, 143.08, 149.7, 153.68, 174.9, 175.54, 188.8 (chalcone C=O), 195.32 (benzophenone C=O) ppm. LRMS (APCI) 587.49 [M^+^+H]. IR *ν* max (ATR): 1710.44, 1678.62, 1651.53, 1622.75, 1372.38, 1278.33, 1323.21, 1022.35, 999.00, 926.58 cm^−1^. HRMS (ESI) calculated for C_39_H_26_N_2_NaO_4_ [M^+^+Na]: 609.1785; found: 609.1786.

(*E*)-13-(4-Benzoylphenyl)-9-(3-(furan-2-yl)-3-oxoprop-1-en-1-yl)-9,10-dihydro-9,10-[3,4]epipyrroloanthracene-12,14-dione (**26o**) was prepared from (*E*)-3-(anthracen-9-yl)-1-(furan-2-yl)prop-2-en-1-one (1.0 mmol, 0.298 g) and 1-(4-benzoylphenyl)-1*H*-pyrrole-2,5-dione (1.3 mmol, 0.361 g) according to the general procedure above; white powder (60%), Mp. >230 °C (dec). IR *ν* max (ATR) 2961.18, 1709.80, 1667.15, 1661.00, 1652.47, 1620.31, 1604.15, 1577.76, 1461.92, 1373.31, 1276.89, 1156.72, 940.89, 724.18 cm^−1^. ^1^H NMR (400 MHz, DMSO-*d_6_*) δ 3.60 (dd, *J* = 8.56, 3.18 Hz, 1H), 3.98 (d, *J* = 8.31 Hz, 1H), 4.99 (d, *J* = 3.42 Hz, 3H), 6.68 (d, *J* = 8.31 Hz, 2H), 7.21–7.28 (m, 2H), 7.28–7.33 (m, 2 H), 7.33–7.38 (m, 1H), 7.39–7.44 (m, 1H), 7.52–7.60 (m, 2H), 7.60–7.64 (m, 1H), 7.65–7.74 (m, 6H), 7.75–7.80 (m, 1H), 7.95 (d, *J* = 16.63 Hz, 1H), 8.14 (d, *J* = 1.47 Hz, 1H). LRMS (APCI) 576.11 [M^+^+H].

(*E*)-13-(4-Benzoylphenyl)-9-(3-(naphthalen-2-yl)-3-oxoprop-1-en-1-yl)-9,10-dihydro-9,10-[3,4]epipyrroloanthracene-12,14-dione (**26p**) was synthesized from (*E*)-3-(anthracen-9-yl)-1-(naphthalen-2-yl)prop-2-en-1-one (1.0 mmol, 0.358 g) and 1-(4-benzoylphenyl)-1*H*-pyrrole-2,5-dione (1.3 mmol, 0.361 g) according to the general procedure above; gray powder (88%). ^1^H NMR (400 MHz, DMSO-*d_6_*) δ 3.58 (dd, *J* = 8.31, 3.42 Hz, 1H), 3.91 (d, *J* = 8.80 Hz, 1H), 5.00 (d, *J* = 3.42 Hz, 1H), 6.69 (d, *J* = 8.31 Hz, 2H), 7.19–7.34 (m, 5H), 7.38 (d, *J* = 4.89 Hz, 1H), 7.41–7.45 (m, H), 7.51–7.58 (m, 2 H), 7.60–7.67 (m, 1H), 7.67–7.77 (m, 5H), 8.11 (d, *J* = 16.14 Hz, H), 8.24 (d, *J* = 7.83 Hz, 1H), 8.32 (d, *J* = 16.63 Hz, 1H), 8.79 (d, *J* = 4.40 Hz, 1H). LRMS (APCI): 636.24 [M^+^+H].

(*E*)-13-(4-Benzoylphenyl)-9-(3-oxo-3-(thiophen-2-yl)prop-1-en-1-yl)-9,10-dihydro-9,10-[3,4]epipyrroloanthracene-12,14-dione (**26q**) was synthesized from (*E*)-3-(anthracen-9-yl)-1-(thiophen-2-yl)prop-2-en-1-one (1.0 mmol, 0.3144 g) and 1-(4-benzoylphenyl)-1*H*-pyrrole-2,5-dione (1.3 mmol, 0.361 g) according to the general procedure above; white solid (51%). Mp. 255–257 °C. ^1^H NMR (400 MHz, DMSO-*d_6_*) δ 3.59 (dd, *J* = 5.39, 3.73 Hz, 1H), 4.02 (d, *J* = 8.71 Hz, 1H), 4.97 (d, *J* = 3.32 Hz, 1H), 6.66 (d, *J* = 8.29 Hz, 2H), 7.19–7.32 (m, 6H), 7.34 (s, 1H), 7.38–7.42 (m, 1H), 7.54 (d, *J* = 7.46 Hz, 2H), 7.60 (s, 1H), 7.65–7.71 (m, 5H), 7.85 (d, *J* = 15.76 Hz, 1H), 7.89 (d, *J* = 16.59 Hz, 1H), 8.12 (dd, *J* = 4.98, 0.83 Hz, 1H), 8.21 (dd, *J* = 3.73, 0.83 Hz, 1H). ^13^C NMR (101 MHz, DMSO-*d_6_*) 45.4, 48.4, 48.8, 52.1, 123.4, 124.0, 124.9, 125.7, 127.0, 127.4, 127.5, 127.6, 128.6, 129.1, 129.3, 129.5, 130.1, 130.6, 131.7, 133.3, 134.6, 135.5, 136.5, 137.0, 137.4, 139.2, 140.0, 141.5, 141.6, 142.7, 145.2, 175.1, 175.5, 182.0, 195.3 ppm. IR *ν* max (ATR): 1734.00, 1699.51, 1665.19, 1646.71, 1618.07, 1520.87, 1493.38, 1460.27 cm^−1^. LRMS (APCI) [M^+^−H]: 590.54. HRMS (ESI) calculated for C_38_H_24_NO_4_S [M^+^−H] 590.1431; Found 590.1430.

Dimethyl (*E*)-9-(3-oxo-3-phenylprop-1-en-1-yl)-9,10-dihydro-9,10-ethenoanthracene-11,12-dicarboxylate (**27**) was synthesized from (*E*)-3-(anthracen-9-yl)-1-phenylprop-2-en-1-one (1 mmol, 0.3084 g) and dimethyl acetylenedicarboxylate (1.3 mmol, 0.16 mL) according to the general procedure above; orange/yellow crystalline solid (62%), [65], Mp. >250 °C. IR *ν* max (ATR): 1726.80, 1714.47, 1679.84, 1644.84, 1601.46, 1448.65, 1325.56, 1294.56, 1276.25, 947.76 cm^−1^. ^1^H NMR (400 MHz, DMSO-*d_6_*) δ 3.64 (s, 3H), 3.72 (s, 3H), 5.80 (s, 1H), 7.07–7.15 (m, 4H), 7.45–7.50 (m, 2H), 7.52–7.58 (m, 3H), 7.61 (m, 2H), 7.73 (d, *J* = 16.12 Hz, 2H), 8.08 (d, *J* = 7.77 Hz, 2H). ^13^C NMR (101 MHz, DMSO-*d_6_*) 49.98, 52.95, 53.23, 57.83, 122.94, 124.84, 125.77, 126.31, 129.13, 129.56, 133.42, 134.17, 137.31, 137.80, 142.62, 143.97, 145.09, 154.91, 163.57, 167.01, 189.21 ppm. HRMS (ESI) calculated for C_29_H_22_NaO_5_ [M^+^+Na]: 473.1360; found: 473.1351.

### 3.8. Stability Study of Compounds ***21a***, ***21i***, ***22h***, ***23a***, ***23g***, ***23n***, ***24a***, **24h**, **26a** and **26n**

The HPLC stability studies for compounds **21a**, **21i**, **22h**, **23a**, **23g**, **23n**, **24a**, **24h**, **26a** and **26n** were performed using a Symmetry^®^ column (C18, 5 µm, 4.6 × 150 mm), a Waters 2487 Dual Wavelength Absorbance detector, and an HPLC pump: Waters 1525 binary and Autosampler; Waters 717 plus (Waters Corporation, Milford, MA, USA), mobile phase [(acetonitrile (80%) and water (20%)] with a typical run time of 10 min and a flow rate of 1 mL/min with detection at 254 nm. The selected compounds **21a**, **21i**, **22h**, **23a**, **23g**, **23n**, **24a**, **24h**, **26a** and **26n** (5 mg) in the mobile phase (10 mL) provided the stock solution for the analysis. The phosphate buffers were prepared at pH values of 4, 7.4 and 9, as described in the *British Pharmacopoeia* monograph 2019. The stock solution (30 µL) was added to the appropriate buffer (1 mL) and retained at 37 °C. Samples were then analyzed over the following 24-h period at selected time intervals (*t* = 0 min, 5 min, 30 min, 60 min, 90 min, 120 min and 24 h).

### 3.9. X-ray Crystallography Analysis

Samples were mounted on a MiTeGen micromount with NVH immersion oil. Data for **21k**, **23f**, **23h**, **24a** and **24g** were collected from a shock-cooled single crystal at 100(2) K on a Bruker D8 Quest ECO three-circle diffractometer with a standard sealed X-ray tube using a graphite monochromator and a CMOS area detector. The diffractometer was equipped with an Oxford Cryostream 800 low-temperature device, and it used Mo*K_α_* radiation (λ = 0.71073 Å). Data for **25f** and **27** were collected from a shock-cooled single crystal at 100(2) K on a Bruker APEX2 Kappa Duo Kappa diffractometer with a microfocus sealed X-ray tube using mirror optics as a monochromator and an APEX2 detector. The diffractometer was equipped with an Oxford Cobra low-temperature device, and it used Cu *K_α_* radiation (λ = 1.54178 Å). All data were integrated with SAINT, and a multi-scan absorption correction using SADABS was applied [95,96]. Structures were solved with dual methods using XT and refined with full-matrix least-squares methods against *F*^2^ by XL using Olex2 [97,98,99]. All non-hydrogen atoms were refined with anisotropic displacement parameters. All hydrogen atoms were refined in an isotropic manner on calculated positions using a riding model with their *U*_iso_ values constrained to 1.5 times the *U*_eq_ of their pivot atoms for terminal sp^3^ carbon atoms and 1.2 times for all other carbon atoms. Disordered moieties were refined using bond lengths’ restraints and displacement parameter restraints.

In **23f**, the N-H hydrogen was located on the difference map and refined with restraints (DFIX). The molecule has a chiral center at C3 and C4; however, it is a racemate, and the R configuration is shown (centrosymmetric space group). In **23h**, the pendant fluorophenyl group was modeled as disordered over two positions (C24–F1) with 50% occupancy. Restraints (SADI, DFIX and RIGU) and constraints (EADP) were used to model the disorder.

In **24a**, the majority of the molecule was disordered over two positions with approx. 61:39% occupancy. Restraints (SIMU and ISOR) and some constraints (EADP) were used in the model for L.S. convergence. **25f** was a poorly diffracting small crystal with weak high-angle data and incomplete Friedel pair collection. The model has chirality at C25 and C31, S (Polar Space Group).

The crystallographic data for the structures reported here were deposited with the Cambridge Crystallographic Data Centre [100] (CCDC) for **21k** (Deposition Number 2341799), **23f** (Deposition Number 2341800), **23h** (Deposition Number 2341801), **24a** (Deposition Number 2341802), **24g** (Deposition Number 2341803), **25f** (Deposition Number 2341804) and **27** (Deposition Number 2341805), and they contain the supplementary crystallographic data for this paper. These data can be obtained free of charge from the Cambridge Crystallographic Data Centre via www.ccdc.cam.ac.uk/structures.

### 3.10. In Silico Computational Study

Two-dimensional structures of the 21 novel anthracene compounds and maprotiline were exported from MOE post-standardization in the SMILES format [101]. The exported structures were then individually submitted as queries to the SwissTargetPrediction (STP) web service [86]. For each of the 22 submitted structures, STP automatically generated its structural fingerprints and estimated its 3D shape. Based on these characteristics, STP then searched its internal database of known active compounds (based mainly on ChEMBL data) [102] for ones that are similar in structure and/or shape [103]. All compounds discovered through the similarity search were then tracked via STP to their specific biological targets. All targets hit by these similar compounds were then compiled into a prioritized list. This potential target list was generated and downloaded in a tabular format for each of the 21 + 1 query structures. The resulting target lists were aggregated and further processed in a standard Python/Pandas data analysis environment [104].

### 3.11. Molecular Modeling: Computational Overlay Study

All the compounds were opened in a database viewer. The compounds were washed with default values, and explicit hydrogens were added. For each compound, MMFF94x partial charges were calculated, and each was minimized to a gradient of 0.001 kcal/mol/Å. The compounds were then overlaid individually on a 3D structure of maprotiline using flexible alignment on MOE with default values [105].

### 3.12. Biochemistry

#### 3.12.1. Materials

The EBV-transformed CLL PGA-1 (M-IGVH, good prognosis) and HG-3 (UM-IGVH, poor prognosis) cell lines were provided by Professor Anders Rosén (Linköping University, Linköping, Sweden) [106]. alamarBlue was obtained from BioSource, Nivelles, Belgium, and fetal bovine serum (FBS) was sourced from Invitrogen, U.K. RPMI 1640 medium, HEPES, gentamycin (G418) and glutamine were sourced from Thermo Fisher Scientific, Dun Laoghaire, Ireland. Cell culture consumables were obtained from Greiner Bio-One Ltd., Stonehouse, U.K., while all other reagents used were obtained from Sigma-Aldrich (now Merck), Arklow, Ireland.

#### 3.12.2. Cell Culture

The CLL PGA-1 and HG-3 leukemia cell lines were grown in RPMI-1640 (Glutamax; Thermo Fisher Scientific, Inc., Dun Laoghaire, Dublin, Ireland) medium that was supplemented with 10% (*v*/*v*) FBS, 50 µg/mL of streptomycin and 50 U/mL of penicillin. The cells were seeded at a density of 2 × 10^5^ cells/mL. The cell cultures were maintained at 37 ◦C in a humidified atmosphere of 5% CO_2_/95% O_2_, and the cells were passaged at least twice weekly, according to their confluency.

#### 3.12.3. AlamarBlue Cell Viability Assay

CLL cells (HG-3 and PGA-1) were seeded at a density of 2 × 10^5^ cells/well (200 µL per well) in 96-well plates. The cells were treated with the appropriate concentrations of test compounds or control drugs (maprotiline and fludarabine), and the samples were incubated for 24 h. alamarBlue (20 µL) was then added to each well, and the samples were further incubated at 37 ◦C in the dark for 4 h. Wells containing only the reagent and cell culture medium (without cells) were used as blank controls, and the vehicle used was DMSO (1% *v/v*) in all experiments. The 96-well plates were analyzed on a fluorescence plate reader (SpectraMax Gemini, Molecular Devices) with emission and excitement wavelengths of 590 nm and 544 nm, respectively, and fluorescence was measured. The decrease in cell viability was obtained with reference to the vehicle (100% viability). An RPMI medium containing alamarBlue was used as a blank. The viabilities of the compounds were calculated with reference to the vehicle-treated cells (100% viable). A non-linear, sigmoidal dose–response curve was obtained from transformed data (Final Concentration = Log (Final Concentration); IC_50_ values (concentration of drug required for 50% reduction in cell survival) were obtained using Prism (Prism 10 for mac OS 10.0.2 GraphPad Software, Inc., La Jolla, CA, USA). The biochemical assays were performed in triplicate on at least three independent occasions, and the mean values were determined.

#### 3.12.4. Annexin V/PI Apoptosis Assay

CLL cells PGA-1 and HG-3 (1 × 10^6^ cells/mL) were treated at 37 °C with either the vehicle (1% (*v*/*v*) DMSO) or the ethanoanthracene compounds **20a**, **20f**, **23a** and **25n** (10 µM, 5 µM and 1 µM) for 48 h, following the protocol previously described [40]. The cells were then harvested via centrifugation at 400× *g* in a temperature-controlled Sorvall centrifuge. The cells were rinsed with Ca^2+^ Annexin-V-binding buffer (0.1 M HEPES, pH 7.4; 0.14 M NaCl; 25 mM CaCl_2_, 0.5 mL). The samples were resuspended in FITC Annexin V (diluted 1:33 in Ca^2+^ Annexin V-binding buffer, 50 µL) and incubated for 10 min (on ice and protected from light). The samples were washed with Annexin-V-binding buffer and resuspended in a PI solution (0.5 µg/mL, 500 µL) and analyzed within 1 h using a BD Accuri C6 flow cytometer counting 10,000 cells using the FlowJo software Version 10 (FlowJo LLC, Ashland, OR, USA).

#### 3.12.5. Inhibitor Studies: Reactive Oxygen Species (ROS)

The ROS cell viability assay included the pre-treatment of CLL cells PGA-1 and HG-3 with *N*-acetyl cysteine. *N*-acetyl cysteine (NAC) (Sigma, Berrinba, Queensland, Australia) was dissolved in sterile water and subsequently diluted to obtain a 5-mM stock solution concentration. Fresh solutions were prepared for each experiment. CLL cells PGA-1 and HG-3 were seeded at a density of 2 × 10^5^ cells/mL in a 96-well plate. The cells were protected from light and pre-treated with NAC (2 µL) for 1 h before then being treated with compounds **20a, 20f, 23a** and **25n** at concentrations 1 µM or 10 µM for 24 h. The remainder of the assay was carried out as described previously for the alamarBlue cell viability assay.

#### 3.12.6. Caspase Inhibitor Study

Compounds **20a** and **23a** (representing Series 1 and Series 4, respectively) were selected for evaluation at two treatment concentrations, 10 µM and 1 µM, across both HG-3 and PGA-1 CLL cells. The HG-3 and PGA-1 CLL cells were seeded at a density of 2 × 10^5^ cells/mL and were subsequently pre-treated at 37 ◦C with 40 µM of pan-caspase inhibitor Z-VAD-FMK (G-Biosciences, Geno Technology Inc., St. Louis, MO, USA) per well for 4 h prior to compound treatment for 24 h. The caspase assay protocol was adapted from Bright et al. [85], and the remainder of the assay was carried out as described above for the alamarBlue cell viability assay and completed in triplicate on two independent days. The output was statistically analyzed using a one-way ANOVA with a Bonferroni multiple-comparison test. Statistical significance was defined as any comparison with a generated *p*-value of <0.05.

#### 3.12.7. Cytotoxicity Assay

The cytotoxicity of the selected compounds **20a, 20f, 23a** and **25n** was determined using the CytoTox 96 non-radioactive cytotoxicity assay (Promega Corporation; 2800 Woods Hollow Road, Madison, WI, USA). CLL cells HG-3 and PGA-1 were incubated for 24 h and then treated with the selected compounds **20a, 20f, 23a** and **25n** at 10-µM and 1-µM concentrations, following the protocol outlined above in the cell viability assay. After 24 h, the lysis solution (10×) (20 µL) was added, and the cells were incubated for a further 1 h to ensure complete cell death. Supernatant (50 µL) was removed from each well and transferred to a clean 96-well plate. “Substrate mix” (50 µL) was added, and the plate was stored in the dark at 20 °C for 30 min. “Stop solution” (50 µL) was added to each well, and the absorbance at 490 nm was recorded using a Dynatech MR5000 plate reader; the % cell death was determined relative to the control cell lysis solution.

#### 3.12.8. Evaluation of Selected Compounds **20a**, **23a** and **25n** in Donor Peripheral Blood Mononuclear Cells (PBMCs)

The human peripheral blood mononuclear cells (PBMCs) were generated as follows: peripheral blood was obtained from healthy donors (n = 2) after informed consent was received. The blood was then placed into a 50-mL falcon tube and diluted with an equal volume of phosphate-buffered saline (PBS). PBMCs were isolated using density-gradient centrifugation with LymphoPrep, as described previously [107]. The cells were resuspended in RPMI media containing 10% FBS and 1% penicillin/streptomycin (P/S). The cells were counted using a haemocytometer and seeded into 96 well plates at a density of 1 × 10^5^ cells/well (200 µL), and they were incubated at 37 °C for 1 h prior to compound treatment. The Annexin V/PI assay was used as a preliminary method of assessing the selective toxicity of the above compounds when compared with a previous alamarBlue cell viability assay in CLL cell lines. The assay was carried out as described above for the Annexin V/PI assay previously described above, and it was completed in triplicate on two independent days. Approval for this study was obtained from the School of Pharmacy and Pharmaceutical Sciences Trinity College Dublin Research Ethics Committee (2020-06-01-MS).

## 4. Conclusions

Three new classes of targeted agents have been approved for the treatment of CLL in the last decade: BTK inhibitors (ibrutinib, acalabrutinib and zanubrutinib), the BCL2 inhibitor (venetoclax) and PI3K inhibitors (idelalisib and duvelisib). However, the development of resistance or intolerance to these multiple classes of drugs is now observed, and new therapeutic options for this growing population of patients is identified as an unmet medical need [7,30,34,36,108,109,110,111].

In our work, we report the synthesis of compounds that demonstrate a significant antiproliferative action in CLL cell lines and also promote apoptosis. However, we have not yet identified the molecular target(s) for these compounds. The further investigation of the mechanism of action of the compounds reported in our research (particularly against the targets identified in our in silico study) may offer an alternative approach to the development of effective drugs for the treatment of CLL patients who have development resistance to the targeted therapy drugs.

In the present work, we have identified a series of potent lead compounds that have nitrostyrene-ethanoanthracene and chalcone-ethanoanthracene scaffold structures. The structures of the novel ethanoanthracene compounds were confirmed via X-ray crystallography. According to preliminary biochemical screening in CLL cell lines HG-3 and PGA-1, the compounds displayed highly effective antiproliferative activity in the CLL cell lines. The lead compounds were identified as **20a**, **20f**, **23a** and **25n**, with IC_50_ values in the ranges of 0.17–2.69 µM (HG-3) and 0.35–1.97 µM (PGA-1, with a more potent effect than the fludarabine control drug). **25n** was the most potent compound in both CLL cell lines (HG-3, IC_50_ = 1.31 µM; PGA-1, IC_50_ = 0.87 µM). The lead compounds were subsequently found to induce cell death via a pro-apoptotic mechanism. The compounds also demonstrated potent antiproliferative activity in a panel of leukemia cell lines, with the mean GI_50_ values for compound **25n** across the leukemia cell panel determined as 0.29 µM. Evidence for ROS involvement in their antiproliferative activity suggested a structure-dependent factor linked to the potential degree of ROS involvement. Caspase dependence was indicated for the representative anthracene–chalcone lead compound **23a**. The compounds were demonstrated to be relatively non-toxic in LDH assays and also in PBMCs. While the biological macromolecular target(s) for the antiproliferative action of these compounds has yet to be identified, they may be effective through the disruption of signaling pathways or tumor-microenvironmental signaling. The antiproliferative nitroalkenes and chalcones identified in this study may act as electrophilic substrates for Michael-addition reactions of nucleophiles occurring in CLL cells and biological systems. The in silico target prediction remains inconclusive, as no singular target responsible for the activity has been identified. However, the target prediction indicated some potential targets for future investigations of molecular docking with selected family-A GPCRs, together with JNK, MAP and VEGFR kinases. This docking campaign, followed by molecular dynamics confirmation of the simulated protein–ligand interactions, could indicate which of these targets, if any, should be selected for in vitro experiments.

Overall, the identified lead compounds **20a**, **23a**, **25n** and **20f** represent promising potential scaffolds for CLL drug discovery, and they warrant further preclinical study as lead compounds for the development of more selective and potent antileukemic agents.

## Figures and Tables

**Table 1:** Anthracene chalcones **21a–q** and ethanoanthracenes **22a–q**, **23a–q**, **24a–q**, **25a–q** and **26a–q**.

	Series 2		Series 3		Series 4		Series 5		Series 6		Series 7	
R	Cpd No.	Yield %	Cpd No.	Yield %	Cpd No.	Yield %	Cpd No.	Yield %	Cpd No.	Yield %	Cpd No.	Yield %
4-Br-C_6_H_4_	**21a**	65	**22a**	86	**23a**	91	**24a**	84	**25a**	95	**26a**	71
4-NO_2_-C_6_H_4_	**21b**	78	**22b**	77	**23b**	87	**24b**	86	**25b**	82	**26b**	75
4-C_2_H_5_-C_6_H_4_	**21c**	61	**22c**	30	**23c**	82	**24c**	90	**25c**	3	**26c**	76
2,4-(Cl)_2_-C_6_H_3_	**21d**	75	**22d**	76	**23d**	82	**24d**	75	**25d**	87	**26d**	63
3,4-(OCH_3_)_2_-C_6_H_3_	**21e**	85	**22e**	83	**23e**	83	**24e**	93	**25e**	4	**26e**	72
C_6_H_5_	**21f**	44	**22f**	91	**23f**	61	**24f**	42	**25f**	48	**26f**	49
4-I- C_6_H_4_	**21g**	70	**22g**	81	**23g**	93	**24g**	20	**25g**	92	**26g**	85
4-F-C_6_H_4_	**21h**	88	**22h**	79	**23h**	100	**24h**	88	**25h**	88	**26h**	77
4-OCH_3_-C_6_H_4_	**21i**	58	**22i**	30	**23i**	70	**24i**	56	**25i**	63	**26i**	50
4-CH_3_-C_6_H_4_	**21j**	61	**22j**	73	**23j**	45	**24j**	51	**25j**	60	**26j**	59
3,4,5-(OCH_3_)_3_-C_6_H_2_	**21k**	86	**22k**	78	**23k**	53	**24k**	62	**25j**	35	**26k**	44
4-Cl-C_6_H_4_	**21l**	65	**22l**	89	**23l**	65	**24l**	20	**25l**	39	**26l**	30
4-(4-Pyridyl)	**21m**	48	**22m**	99	**23m**	100	**24m**	82	**25m**	92	**26m**	62
4-(2-Pyridyl)	**21n**	58	**22n**	81	**23n**	72	**24n**	91	**25n**	57	**26n**	75
2-Furyl	**21o**	67	**22o**	81	**23o**	81	**24o**	87	**25o**	46	**26o**	60
2-Naphthyl	**21p**	38	**22p**	86	**23p**	86	**24p**	65	**25p**	88	**26p**	55
2-Thienyl	**21q**	86	**22q**	31	**23q**	80	**24q**	81	**25q**	70	**26q**	51

**Table 2:** X-ray crystal structure of compounds **21k**, **23f**, **23h**, **24a**, **24g**, **25f** and **27** ^a,b^.

Compound	X-Ray Structure	Compound	X-Ray Structure
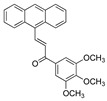 **21k**	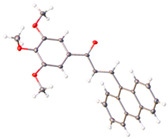	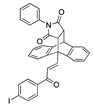 **24g**	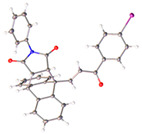
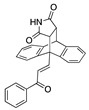 **23f**	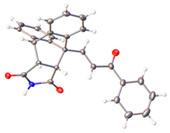	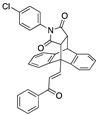 **25f**	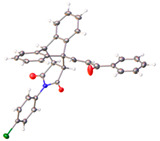
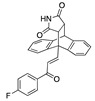 **23h**	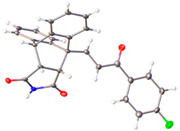	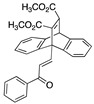 **27**	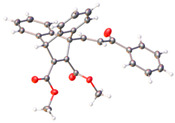
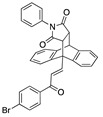 **24a**	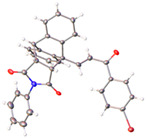		

^a^ X-ray crystal structure of compounds **21k**, **23f**, **23h**, **24a**, **24g**, **25f** and **27** with the thermal ellipsoids set to 50% probability. ^b^ Crystallographic data deposited with the Cambridge Crystallographic Data Centre (CCDC) **21k** (Deposition Number 2341799)**, 23f** (Deposition Number 2341800), **23h** (Deposition Number 2341801), **24a** (Deposition Number 2341802), **24g** (Deposition Number 2341803), **25f** (Deposition Number 2341804) and **27** (Deposition Number 2341805).

**Table 3:** IC_50_ values of selected (*E*)-9-(2-nitrovinyl)ethanoanthracenes **20a–20f** in HG-3 and PGA-1 cell lines.

Compound Number	HG-3 IC_50_ (µM) ^a^	PGA-1 IC_50_ (µM) ^a^	Mean IC_50_ (µM) ^a^	logP ^b^
**20a**	0.48	0.61	0.55	3.29
**20b**	0.85	0.66	0.76	3.81
**20c**	0.71	0.88	0.8	3.54
**20d**	0.78	0.76	0.77	1.86
**20e**	1.6	1.1	1.4	2.98
**20f**	0.17	0.35	0.26	4.30
**Fludarabine**	28.1	32	30.1	−2.5 ^c^

^a^ The cell proliferation of HG-3 and PGA-1 cells was determined with an alamarBlue assay (seeding density: 1–5 × 10^4^ cells/mL per well for 96-well plates). IC_50_ values are half-maximal inhibitory concentrations required to block the growth stimulation of HG3 and PGA-1 cells. The values represent the mean ± SEM (error values × 10^−6^) for at least three experiments performed in triplicate at 24 h. Treatment at seven different concentrations (0.01–50 µM) was used for the determination of the IC_50_ values for each compound. ^b^ Predicted logP (consensus) property using Swiss ADME; ^c^ predicted property using Chemaxon.

**Table 4:** IC_50_ values of selected ethanoanthracene derivatives in HG-3 and PGA-1 CLL cell lines.

Compound Number	HG-3 IC_50_ (µM) ^a^	PGA-1 IC_50_ (µM) ^a^	Mean IC_50_ (µM) ^a^	logP ^b^
**23a**	2.69	1.97	2.33	4.35
**23c**	4.61	2.72	3.67	4.36
**23f**	3.55	11.3	7.43	3.73
**23g**	4.47	2.77	3.62	4.38
**23h**	4.65	2.88	3.77	4.05
**23i**	10.28	12.6	11.4	3.72
**23k**	4.9	5.38	5.19	3.66
**23l**	2.23	3.21	2.72	4.27
**23n**	2.39	1.48	1.94	3.03
**23p**	1.41	1.30	1.36	4.62
**24f**	7.35	7.13	7.24	3.69
**24l**	3.45	0.31	1.88	5.66
**25n**	1.31	0.87	1.09	4.98
**26n**	1.85	0.39	1.12	5.51
**Fludarabine**	28.1	32.0	30.1	−2.5 ^c^

^a^ The cell proliferation of HG-3 and PGA-1 cells was determined with an alamarBlue assay (seeding density: 1–5 × 10^4^ cells/mL per well for 96-well plates). IC_50_ values are half-maximal inhibitory concentrations required to block the growth stimulation of HG-3 and PGA-1 cells. Values represent the mean ± SEM (error values × 10−6) for at least three experiments performed in triplicate at 24 h. Treatment at seven different concentrations (0.01–50 µM) was used for the determination of the IC_50_ values for each compound. ^b^ Predicted logP (consensus) property using Swiss ADME; ^c^ predicted logP property using Chemaxon.

**Table 5:** Summary of NCI 60-cell-line screening results for selected ethanoanthracene compounds in the leukemia cell panel **^a^**.

Cell Line	GI_50_ µM ^b^											
Compound Number	23c ^c^	23d ^d^	23f ^e^	23h ^f^	23l ^g^	23m ^h^	23n ^i^	23o ^j^	24f ^k^	24l ^l^	25n ^m^	26n ^n^
**CCRF-CEM**	2.66	1.24	2.16	2.45	1.66	2.39	1.44	1.89	1.45	0.57	0.32	2.59
**HL-60 (TB)**	0.07	1.58	1.5	0.02	1.47	1.76	1.02	0.32	1.02	0.8	0.29	1.9
**K-562**	2.42	2.19	1.94	1.42	1.99	1.88	1.37	2.57	1.39	1.55	0.29	1.77
**MOLT-4**	2.23	2.16	2.17	0.46	1.7	2.57	2.11	1.77	1.23	0.76	0.32	2.29
**RPMI-8226**	1.87	1.1	1.52	1.62	1.66	2.11	1.22	1.68	1.55	1.16	0.25	2.49
**SR**	1.37	1.25	1.57	0.29	2.15	1.76	0.78	1.59	2.03	1.07	0.28	1.37
**Mean**	1.77	1.59	1.81	1	1.77	2.08	1.32	1.64	1.45	0.98	0.29	2.07

^a^ Data obtained from NCI in vitro, human-tumor-cell-screen, five-dose assay [72]. ^b^ GI50 is the molar concentration of the compound causing a 50% inhibition to the growth of the tumor cells; ^c^ NSC 803357; ^d^ NSC 803361; ^e^ NSC 815555; ^f^ NSC 803358; ^g^ 815556; ^h^ 803363; ^i^ 803355; ^j^ 803360; ^k^ 815557; ^l^ 815558; ^m^ 815561; and ^n^ 815562.

**Table 6:** Antitumor evaluation of compounds **23h, 23l** and **25n** in NCI 60-cell-line screen ^a^.

Cell Line		Compound 23h	Compound 23l	Compound 25n
		GI_50_ (µM) ^b,c^	GI_50_ (µM) ^b,d^	GI_50_ (µM) ^b,e^
***NSC Lung Cancer***	A549/ATCC	2.37	2.40	0.390
	EKVX	1.85	1.43	0.491
	HOP-62	2.45	1.69	0.587
	HOP-92	1.33	1.32	1.19
	NCI-H226	2.04	1.92	0.293
	NCI-H23	2.61	1.78	0.446
	NCI-H332M	2.64	1.42	0.821
	NCI-H460	1.64	1.80	0.241
	NCI-H552	1.74	1.46	0.331
***Colon Cancer***	COLO205	1.79	1.91	1.16
	HCT-2998	1.70	1.69	0.335
	HCT-116	0.537	1.23	0.212
	HCT-15	1.19	1.47	0.213
	HT29	1.86	2.27	0.335
	KM12	2.18	2.14	0.344
	SW-620	2.27	1.89	0.299
***CNS Cancer***	SF-268	4.56	1.99	0.699
	SF295	nd ^f^	nd^f^	0.379
	SF539	1.69	1.51	0.316
	SNB-19	2.68	1.58	0.425
	SNB-75	1.37	1.10	nd^f^
	U251	1.84	1.52	0.358
***Prostate Cancer***	PC-3	1.42	1.39	0.436
	DU-145	1.77	1.67	0.410
***Melanoma***	LOX IMV1	1.65	1.60	0.194
	MALME-3M	1.60	1.51	0.316
	M14	2.14	1.65	0.344
	MDA-MB-435	1.93	1.68	0.298
	SK-MEL-2	2.48	1.81	0.670
	SK-MEL-28	1.98	1.80	0.455
	SK-MEL-5	1.95	1.70	1.08
	UACC-257	2.13	1.56	0.496
	UACC-62	1.66	1.40	0.400
	IGROV1	2.10	1.67	0.236
	OVCAR-3	1.44	1.63	0.222
***Ovarian Cancer***	OVCAR-4	1.96	1.42	0.764
	OVCAR-5	1.60	1.58	0.568
	OVCAR-8	1.43	1.13	0.409
	NCI/ADR-RES	2.16	1.77	0.356
	SK-OV-3	5.28	1.85	3.30
	786-0	2.44	1.73	0.216
	A498	1.16	1.28	0.539
	ACHN	1.39	1.65	0.299
***Renal Cancer***	CAKI-1	1.02	1.24	1.07
	RXF 393	1.27	1.20	0.222
	SN12C	2.34	1.56	0.415
	TK-10	3.18	1.92	1.46
	UO-31	1.12	1.28	0.208
	MCF-7	0.885	1.34	0.326
***Breast Cancer***	MDA-MB-231/ATCC	1.64	1.88	0.463
	HS 578T	1.82	1.94	0.458
	BT-549	1.37	1.34	0.295
	T-47D	nd^f^	nd^f^	0.312
	MDA-MB-468	1.88	1.70	0.627
	CCRF-CEM	0.566	1.66	0.321
	HL-60(TB)	0.795	1.47	0.292
***Leukemia***	K-562	1.55	1.99	0.293
	MOLT-4	0.759	1.70	0.366
	RPMI-8226	1.16	1.66	0.252
	SR	1.07	2.15	0.284
	MID GI_50_^g^	1.698	1.621	0.245
	MID TGI ^h^	4.265	4.265	1.905
	MID LC_50_^i^	14.45	13.183	18.62

^a^ Data obtained from NCI in vitro human tumor cell screen 5 dose assay [72]. ^b^ GI50 is the molar concentration of the compound causing a 50% inhibition of the growth of the tumor cells; ^c^ NSC 815558; ^d^ NSC 815556; ^e^ NSC 815561; ^f^ Nd: Not determined; ^g^ MID GI_50_: MG-MID full-panel mean-graph mid-point; ^h^ TGI (total growth inhibition) is the mean concentration required to completely inhibit the growth of all cells over the total NCI 60 cell line panel; ^i^ LC_50_ is the mean concentration required to kill 50% of the cells over the total NCI 60-cell-line panel in the assay.

**Table 7:** Summary of NCI 60-cell lines’ mean screening results (GI_50,_ TGI and LC_50_) for selected compounds.

NCI Reference Number	Compound Number	Mean GI_50_ (µM) ^a^	Mean TGI (µM) ^b^	Mean LC_50_ (µM) ^a^
**803357**	**23c**	1.905	4.670	16.98
**803361**	**23d**	2.511	7.580	29.51
**815555**	**23f**	1.819	4.570	14.79
**803358**	**23h**	1.698	4.265	14.45
**815556**	**23l**	1.621	4.265	13.18
**803363**	**23m**	2.511	7.760	28.18
**803355**	**23n**	1.659	4.570	14.79
**803360**	**23o**	1.778	3.890	10.97
**815557**	**24f**	2.089	19.95	72.40
**815558**	**24l**	1.698	10.72	43.70
**815561**	**25n**	0.245	1.905	18.62
**815562**	**26n**	7.585	100.0	100.0

^a^ GI_50_ and LC_50_ are the mean concentrations required to inhibit growth and kill 50% of the cells in the assay (all cell lines), respectively [73]. ^b^ TGI is the mean concentration required to completely inhibit the growth of all cells [73].

**Table 8:** Overlay of ethanoanthracenes (**20a**, **20b**, **20d**, **20f**, **23a** and **25n**) and nitrostyrene **18b** on maprotiline with their overlay scores ^a^.

Compound No.	Overlay of Compounds (Green) with Maprotiline (Pink)	Overlay (F) Score	Overlay (S) Score	Mean IC_50_ (µM)
**20a**	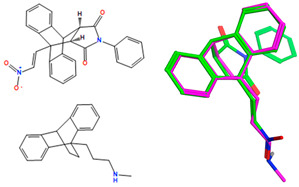	−157.66	−66.46	0.55
**20b**	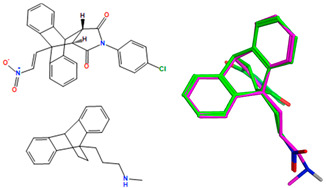	−158.37	−48.91	0.76
**20d**	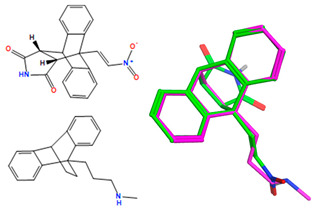	−156.30	−86.35	0.77
**20f**	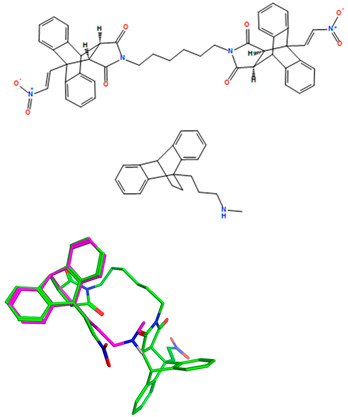	−163.50	−65.11	0.53
**23a**	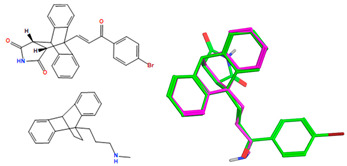	−165.19	−79.80	2.33
**25n**	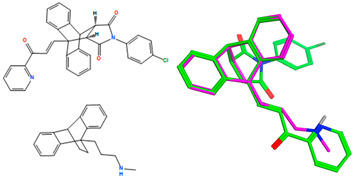	−169.14	−58.04	1.09
**Anthracene 18b**	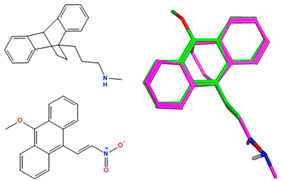	−132.50	−36.05	0.73

^a^ Overlay of ethanoanthracene compounds series and nitrostyrene compound **18b** on maprotiline with their overlay scores (F and S) in MOE 2022, 2D images and mean IC_50_ values. The F column contains the similarity score (the lower, the better) of the alignment. The S column has the sum of the U (the average internal energy of the ligand) and F values. The compounds shown represent those with the best mean IC_50_ values on the HG-3 and PGA-1 cell lines.

## Data Availability

The original contributions presented in the study are included in the article/Supplementary Materials, further inquiries can be directed to the corresponding author.

## Supplementary Materials

The following supplementary information is available online at https://www.mdpi.com/article/10.3390/ph17081034/s1: Experimental details for the preparation of nitrovinylethanoanthracenes **20a–h** and (E)-3-(anthracen-9-yl)-1-phenylprop-2-en-1-ones **21a-q**; Figure S1: Stability analysis for **23n** at pH 4.0, pH 7.5 and pH 9.0; Figure S2: Bioavailability analysis for compound 23a; Figures S3-S8; X-ray analysis for **21k**, **23f**, **24a**, **23h** and **27**; Figures S9–49: ^1^H NMR, ^13^C NMR, DEPT-90, HSQC and HMBC for ethanoanthracenes; Table S1: X-ray data for compounds **21k**, **23f**, **23h**, 24a, 24g, 25f and 27; Table S2: Stability data for ethanoanthracene 23n at pH 4.0, pH 7.5 and pH 9.0; Tables S3–S6; COMPARE analysis for compounds **23n**, **23h**, **25n** and **24l**; Tables S7–S9: Tier-1 profiling screen, physicochemical descriptors, Lipinski properties pharmacokinetic, ADMET and drug-likeness predictions for selected ethanoanthracenes; and Table S10: Overlay of ethanoanthracene compounds series with maprotiline with their overlay scores.

## Abbreviations

ADA	Adenosine deaminase
ADME	Absorption, distribution, metabolism and excretion
BBB	Blood–brain barrier
Bcl-2	B-cell lymphoma-2 protein
BCR	B-cell receptor
BTK	Bruton’s tyrosine kinase
BL	Burkitt’s lymphoma
CB2	Cannabinoid receptor
CLL	Chronic lymphocytic leukemia
CYP450	Cytochrome P450
CYP24A1	25-Hydroxyvitamin D-24-hydroxylase
DHOD	Dihydroorotate dehydrogenase
DEPT	Distortionless enhancement via polarization transfer
DMSO	Dimethylsulfoxide
EBV	Epstein–Barr virus
FACS	Fluorescence-activated cell sorting
FBS	Fetal bovine serum
FDA	The United States Food and Drug Administration
FITC	Fluorescein isothiocyanate
GSK-3 beta	Glycogen synthase kinase-3 beta
HBD	Hydrogen bond donors
HBA	Hydrogen bond acceptors
HEPES	N-2-Hydroxyethylpiperazine-N′-2-ethanesulfonic acid
HMBC	Heteronuclear multiple-bond correlation
HSQC	Heteronuclear single quantum coherence
HRMS	High-resolution mass spectrometry
IC_50_	Half-maximal inhibitory concentration
IGVH	Immunoglobulin heavy-chain gene
INT	Iodonitrotetrazolium chloride
IR	Infrared
LDH	Lactate dehydrogenase
MALT1	Mucosa-associated lymphoid tissue–lymphoma translocation protein 1
MEM	Eagle’s minimum essential medium
NAC	N-acetylcysteine
NCI	National Cancer Institute
Nrf2	Nuclear factor erythroid 2–related factor 2
NHL	Non-Hodgkin lymphoma
NMR	Nuclear magnetic resonance
PAINS	Pan-assay interference compounds
PBMCs	Peripheral blood mononuclear cells
PI3Kδ	Phosphoinositide 3-kinase δ isoform
PI	Propidium iodide
PD-1	Programmed cell death protein 1
ROS	Reactive oxygen species
RPMI 1640	Roswell Park Memorial Institute 1640 medium
RT	Richter’s transformation
Syk	Spleen tyrosine kinase
TPSA	Topological polar surface area
