# Supplementary material for: Synthesis and Biochemical Evaluation of Ethanoanthracenes and Related Compounds: Antiproliferative and Pro-Apoptotic Effects in Chronic Lymphocytic Leukemia (CLL)
Source: Pharmaceuticals (Basel). 2024 Aug 5;17(8):1034. doi: 10.3390/ph17081034 (PMC11359702; doi:10.3390/ph17081034)
Supplement: Supplementary file 1 [file pharmaceuticals-17-01034-s001.zip › pharmaceuticals-3110507-supplementary.pdf]

Synthesis and Biochemical evaluation of Ethanoanthracenes and Related Compounds: Antiproliferative and Pro-apoptotic Effects in Chronic Lymphocytic Leukaemia (CLL)

James. P. McKeown [1,2], Andrew J. Byrne [1,2], Sandra A. Bright [3], Clara E. Charleton [1,2], Shubhangi Kandwal [4,5,6], Ivan Čmelo [5,6], Brendan Twamley [7], Anthony M. McElligott [8], Darren Fayne [5,6], Niamh M. O'Boyle [1,2], D. Clive Williams [3] and Mary J. Meegan [1,2]*

[1] School of Pharmacy and Pharmaceutical Sciences, The University of Dublin, Trinity College, Panoz Institute, East End 4/5, Dublin 2, D02 PN40, Dublin, Ireland; andrew.byrne87@gmail.com (A.J.B.); mckeowjp@tcd.ie (J.P.M.); mmeegan@tcd.ie (M.J.M.); charletc@tcd.ie (C.E.C.); nioboyle@tcd.ie (N.O'B)

[2] School of Pharmacy and Pharmaceutical Sciences, Trinity Biomedical Sciences Institute, Trinity College Dublin, 152-160 Pearse St, Dublin 2, D02 R590, Dublin, Ireland; andrew.byrne87@gmail.com (A.J.B.); mckeowjp@tcd.ie (J.P.M.); mmeegan@tcd.ie (M.J.M.); charletc@tcd.ie (C.E.C.); nioboyle@tcd.ie (N.O'B)

[3] School of Biochemistry and Immunology, Trinity Biomedical Sciences Institute, Trinity College Dublin, 152-160 Pearse St, D02 R590, Dublin 2, Ireland; brightsandi@gmail.com (S.A.B.); clive.williams@tcd.ie (D.C.W.)

[4] Molecular Design Group, School of Biochemistry and Immunology, Trinity Biomedical Sciences Institute, Trinity College Dublin, 152-160 Pearse St, Dublin 2, D02 R59; kandwals@tcd.ie (S.K.)

[5] Molecular Design Group, School of Chemical Sciences, Dublin City University, Glasnevin, Dublin, D09 V209, Ireland; kandwals@tcd.ie (S.K.), darren.p.fayne@dcu.ie (D.F.), ivan.cmelo@dcu.ie, (I.Č.)

[6] DCU Life Sciences Institute, Dublin City University, Glasnevin, Dublin, D09 V209, Dublin, Ireland; kandwals@tcd.ie (S.K.), darren.p.fayne@dcu.ie (D.F.), Ivan Čmelo (I.Č.)

[7] School of Chemistry, Trinity College Dublin, Dublin 2, D02 P3X2, Ireland; twamleyb@tcd.ie (B.T.)

[8] Discipline of Haematology, School of Medicine, Trinity Translational Medicine Institute, St. James's Hospital and Trinity College, Dublin 8, D08 W9RT, Dublin, Ireland; tony.mcelligott@tcd.ie (A.M.McE.)

* Correspondence: mmeeegan@tcd.ie

Supplementary Information

Experimental chemistry

Table S1: Crystal data and structure refinement for 21k, 23f, 23h, 24a, 24g, 25f and 27.
Table S2: Stability study for compounds **21a, 21i, 22h, 23a, 23g, 23n, 24a, 24h, 26a** and **26n**
Table S3: Top five ranking compounds with **25n**according to NCI-COMPARE Pearson correlation coefficient analysis of GI_{50} profiles
Table S4: Top five ranking compounds with **23n** according to NCI-COMPARE Pearson correlation coefficient analysis GI 50 profiles
Table S5: Top five ranking compounds with **23h** according to NCI-COMPARE Pearson correlation coefficient analysis GI $_{50}$ profiles
Table S6: Top five ranking compounds with **24l** according to NCI-COMPARE Pearson correlation coefficient analysis of GI_{50} profiles
Table S7: Physicochemical descriptors and mean IC_{50} values (CLL) of selected ethanoanthracene compounds (**20a-e**, **23a, 23c, 23d**, **23f-i, 23k-p**, **24f**, **24l, 25n, 26n, 27**)
Table S8: Pharmacokinetic interaction estimations with P-gp and CYP 450 isozymes of selected compounds (**20a-e**, **23a, 23c, 23d**, **23f-I, 23k-p**, **24f**, **24l, 25n, 26n, 27**)
Table S9: Drug-likeness of representative compounds assessed via leadlikeness and reactivity/toxicity filters for selected compounds (**20a-e**, **23a, 23c, 23d**, **23f-I, 23k-p**, **24f**, **24l, 25n, 26n, 27**)
Table S10: Overlay of ethanoanthracene compounds series and nitrostyrene compound **18b** on maprotiline with their overlay scores

Figure S1: Stability testing of compound **23n** at pH 4.0, 7.4, and 9.0 over 24 h
Figure S2: Bioavailability Radar displayed for compound **23n**
Figure S3: X-ray structure of (*E*)-3-(anthracen-9-yl)-1-(3,4,5-trimethoxyphenyl)prop-2-en-1-one **21k** and corresponding crystal packing arrangement
Figure S4: X-ray structure of (*E*)-9-(3-oxo-3-phenylprop-1-en-1-yl)-9,10-dihydro-9,10-[3,4]epipyrroloanthracene-12,14-dione (**23f**) and its corresponding crystal packing arrangement
Figure S5: Packing arrangement of (*E*)-9-(3-(4-bromophenyl)-3-oxoprop-1-en-1-yl)-13-phenyl-9,10-dihydro-9,10-[3,4]epipyrroloanthracene-12,14-dione **24a**
Figure S6: Packing arrangement of dimethyl (*E*)-9-(3-oxo-3-phenylprop-1-en-1-yl)-9,10-dihydro-9,10-ethenoanthracene-11,12-dicarboxylate **27**
Figure S7: Major and minor disordered moieties of **23h** with heteroatoms labelled for clarity. Atomic displacement shown at 50% probability
Figure S8: Packing diagrams of **23h** viewed normal to the (**A**) a-axis and (**B**) b-axis
Figures S9-S49: ^{1}H and 13C NMR spectra

References

Experimental

Chemistry: Materials and methods

All reagents were commercially available and were used without any further purification unless otherwise indicated. Melting points were measured on a Stuart Melting Point Apparatus, SSMP10, and are uncorrected. Infra-red (IR) spectra were recorded on a Perkin Elmer FT-IR Paragon 1000 spectrometer. ^{1}H and ^{13}C nuclear magnetic resonance spectra (NMR) were recorded at 25 °C on a Bruker DPX 400 spectrometer (400.13 MHz, ^{1}H; 100.61 MHz, ^{13}C), Bruker Avance III 400 or Avance II 600 (400.13 MHz/ 600.13 MHz, 1H; 100.61 MHz/150.61 MHz, 13C) in either CDCl3 or DMSO-*d*, in either $CDCl_3$ (internal standard tetramethylsilane (TMS)) or CD_3OD or DMSO-d_6. For $CDCl_3$, ^{1}H-NMR spectra were assigned relative to the TMS peak at 0.00 ppm and ^{13}C-NMR spectra were assigned relative to the middle $CDCl_3$ peak at 77.0 ppm. For CD_3OD, ^{1}H and ^{13}C-NMR spectra were assigned relative to the center peaks of the CD_3OD multiplets at 3.30 ppm and 49.00 ppm respectively. Coupling constants are reported in Hertz. High resolution mass spectrometry (HRMS) was carried out by Dr. Gary Hessman of the School of Chemistry, Trinity College Dublin using atmospheric pressure chemical ionisation (APCI) with occasional use of electrospray ionisation (ESI-MS) in both positive and negative modes. ESI mass spectra were acquired using a Bruker micrOTOF-Q III spectrometer interfaced to a Dionex UltiMate 3000 LC in positive and negative modes as required. Masses were recorded over the range 100–1400 *m*/*z*. APCI experiments were carried out on a Bruker micrOTOF-Q III spectrometer interfaced to a Dionex UltiMate 3000 LC or direct insertion probe. The instrument was operated in positive or negative mode as required. Masses were recorded over a range of 100–1600 *m*/*z*. Mass measurement accuracies of <±5 ppm were obtained. TLC was carried out on silica gel on aluminium foils with fluorescent indicator F-254 nm. Flash column chromatography was carried out on Merck Kieselgel 60 (particle size 0.040-0.063 mm). Analytical high-performance liquid chromatography (HPLC) was performed using a Waters 2487 Dual Wavelength Absorbance detector, a Waters 1525 binary HPLC pump and a Waters 717plus Autosampler, with a Thermo Scientific Hypersil GOLD C18 reverse phase 5 μm 150 × 4.6 mm chromatography column. Samples were detected using wavelengths of 232 nm compounds. All samples were analysed using acetonitrile (75%): TFA (0.1%) in water (25%) over 15 min and a flow rate of 1 mL/min. Microwave experiments were carried out using the Discover CEM microwave synthesiser on standard power setting (300 watts) unless otherwise stated.

General Procedure for synthesis of nitrovinylanthracene 18a, 18b

To a solution of 9-anthraldehyde (2 g, 9.7 mmol) in the appropriate nitroalkane (nitromethane, nitroethane, nitropropane) (15 mL) was added piperidinium acetate (1.5 g, 10.3 mmol). (Piperidinium acetate was prepared from piperidine 6.6 mL and acetic acid 3 mL). The solution was heated at 90 C for 1.5 h under nitrogen for 1 h, then cooled to room temperature and poured onto 100 mL of ice cold H2O. Following DCM extraction, the organic layers were combined, dried (Na2SO4) and solvent removed. The product was recrystallised from an appropriate solvent.

(*E*)-9-(2-nitrovinyl)anthracene (18a) was prepared from 9-anthraldehyde (9.7 mmol, 2 g) and nitromethane (15 mL) following the general procedure above. The product was recrystallized from methanol and diethyl ether as red crystals 2.41 g (99%), Mp. 145–147 °C [1]

(*E*)-9-Chloro-10-(2-nitrovinyl)anthracene 18b was prepared from 10-chloroanthracene-9-carbaldehyde (5 mmol, 1.2 g) and nitromethane (15 mL) following the general procedure above. The product was recrystallized from methanol and diethyl ether as orange crystals, 1.01 g (71%), Mp. 232–234 °C [1].

General Procedure for synthesis of maleimides 19b-d

To a solution of maleic anhydride (20 mmol) dissolved in diethyl ether (25 mL) was added the appropriately substituted amine (20 mmol) dissolved in diethyl ether (10 mL).The reaction was stirred under a reflux condenser at RT for 1 hour. The precipitated solid was isolated by filtration and washed with diethyl ether. This solid was immediately used in the next step and treated with sodium acetate (0.7 g) and acetic anhydride (10 mL). This mixture was heated to 90 °C for 0.5 h, and the mixture was poured over ice water (100 mL). The precipitated solid was isolated and recrystallized from ethanol.

1-(4-Chlorophenyl)-1*H*-pyrrole-2, 5-dione 19b was prepared from maleic anhydride and *p*-chloroaniline according to general procedure above. The product was obtained as light green solid (55 %), Mp. 118-124 °C [1].

1-(4-Benzoylphenyl)-1*H*-pyrrole-2,5-dione 19c was prepared from maleic anhydride and (4-aminophenyl)(phenyl)methanone according to general procedure above. The product was obtained as white crystalline solid (29%), Mp. 156-160 °C, [1].

1,10-(Hexane-1,6-diyl)bis(1*H*-pyrrole-2,5-dione) 19d was prepared from maleic anhydride (20 mmol) and 1,4-diaminobenzene (20 mmol) following the general procedure above. The product was obtained as yellow crystals (60%), Mp. 130–136 C [1].

General method: Preparation of (*E*)-9-(2-nitrovinyl)-9,10,11,15-tetrahydro-9,10-[3,4]epipyrroloanthracene-12,14-diones 20a-g and (*E*)-9-(2-Nitrovinyl)-9,10-dihydro-9,10-[3,4]furanoanthracene-12,14-dione 20h

To a solution of the appropriate nitrovinylanthracene (1.0 mmol) in toluene (2 mL) was added the dienophile (1.3 mmol). The mixture was heated, with stirring at 90 °C for 48 h. The reaction was then cooled to RT and the resulting solid was isolated by filtration. The solid product was sequentially washed with toluene (2 mL) and diethyl ether (2 mL). The product was then recrystallized from toluene.

(*E*)-9-(2-Nitrovinyl)-13-phenyl-9,10-dihydro-9,10-[3,4]epipyrroloanthracene-12,14-dione 20a: Preparation from (*E*)-9-(2-nitrovinyl) anthracene (1.0 mmol, 0.25 g) and *N*-phenylmaleimide (1.3 mmol, 0.23 g) according to general procedure above; the product was obtained as a colourless solid (30%), Mp. 256-257 °C [1].

(*E*)-13-(4-Chlorophenyl)-9-(2-nitrovinyl)-9,10-dihydro-9,10-[3,4]epipyrroloanthracene-12,14-dione 20b: Preparation from (*E*)-9-(2-nitrovinyl)anthracene (1.0 mmol, 0.25 g) and 1-(4-chlorophenyl)-1*H*-pyrrole-2,5-dione (1.3 mmol, 0.27 g) according to general procedure above; colourless solid (51 %) Mp: 238-241 °C [1].(HPLC:95.79, RT 6.66 min).

(*E*)-13-(4-Benzoylphenyl)-9-(2-nitrovinyl)-9,10-dihydro-9,10-[3,4]epipyrroloanthracene-12,14-dione 20c: Preparation from (*E*)-9-(2-nitrovinyl)anthracene **18a** and maleimide according to general procedure above; yellow solid (40%) Mp. 232-234 °C [1].

(*E*)-9-(2-Nitrovinyl)-9,10-dihydro-9,10-[3,4]epipyrroloanthracene-12,14-dione 20d: Preparation from (*E*)-9-(2-nitrovinyl)anthracene **18a** (1.0 mmol, 0.25 g) and maleimide (1.3 mmol, 0.13 g) according to general procedure above, colourless solid (50 %), Mp. 176-178 °C [1].

(*E*)-10-(2-Nitrovinyl)-9,10-dihydro-9,10-ethanoanthracene-11-carbonitrile 20e: Preparation from (*E*)-9-(2-nitrovinyl)anthracene **18a** (1.0 mmol, 0.25 g) and acrylonitrile (3.0 mmol, 0.2 mL) according to general procedure above; orange crystals (30%) Mp. 223-225 °C [1].

13,13′-(Hexane-1,6-diyl)bis(9-((*E*)-2-nitrovinyl)-9,10-dihydro-9,10[3,4]epipyrroloanthracene-12,14 dione) 20f: Preparation from (*E*)-9-(2-nitrovinyl)anthracene **18a** (0.25 g, 1 mmol) and the required maleimide **19d** (0.5 mmol) following the general procedure above to afford the product as a colourless solid, 39 mg (10%), Mp. 186–188 °C [1].

(*E*)-9-Chloro-10-(2-nitrovinyl)-9,10-dihydro-9,10-[3,4]furanoanthracene-12,14-dione 20g: Preparation from (*E*)-9-chloro-10-(2-nitrovinyl)anthracene **18b** (0.28 g, 1 mmol) and maleic anhydride (0.13 g, 1.3 mmol) following general procedure above. The product was obtained as a colourless solid 58 mg (15%), Mp. 275–277 °C [1].

(*E*)-9-(2-Nitrovinyl)-9,10-dihydro-9,10-[3,4]furanoanthracene-12,14-dione 20h: Preparation from (*E*)-9-(2-nitrovinyl)anthracene **18a** (0.25 g, 1 mmol) and maleic anhydride (0.13 g, 1.3 mmol) following general above. The product was obtained as a colourless solid, 276 mg (80%), Mp. 244–245 C [1].

General Procedure for the preparation of (*E*)-3-(anthracen-9-yl)-1-phenylprop-2-en-1-ones 21a-q

To a solution of NaOH (6 mmol, 0.24 g) in 50% aqueous EtOH (20 mL) was added the appropriate acetophenone (5.02 mmol). After dissolution of the acetophenone, 9-anthracenecarboxaldehyde (5.02 mmol, 1.035 g) was added, resulting in a coloured solution. This was stirred at room temperature for 24 h to give a coloured suspension which was filtered, washed with minimal cold EtOH and dried. The crude product was then recrystallized from the minimal amount of EtOH or MeOH to afford pure product.

(*E*)-3-Anthracen-9-yl)-1-(4-bromophenyl)prop-2-en-1-one 21a: Preparation from 4-bromoacetophenone (5.02 mmol, 1 g) and 9-anthracenecarboxaldehyde (5.02 mmol, 1.035 g) according to the general procedure above to afford yellow crystals (65%) Mp. 164 °C [2]. (HPLC: 88.45%, RT 2.37 min). ^{1}H NMR (400 MHz, $CDCl_3$) δ 7.46 - 7.54 (m, 5 H, 4xArH, 1x C=CH), 7.61 - 7.66 (m, 2 H, ArH), 7.89 - 7.95 (m, 2H, ArH), 7.99 - 8.04 (m, 2 H,ArH), 8.24 - 8.29 (m, 2 H, ArH), 8.46 (br.s, 1 H, ArH), 8.80 (d, *J*=15.76 Hz, 1 H, C=CH). ^{13}C NMR (101 MHz, $CDCl_3$) ppm 125.15, 125.43, 125.35, 126.5, 128.26, 128.62, 128.95, 129.61, 129.84, 130.18, 130.37, 131.27, 132.05, 136.58, 142.47, 188.50 (C=O) IR *ν* max (KBr): 3048.16 (Ar C–H), 1593.87, 1332.11 (Ar C=C), 1730.51 (*trans* C=C), 1657.97 (C=O), 1070.45 (Ar Br) cm^{-1}

(*E*)-3-(Anthracen-9-yl)-1-(4-nitrophenyl)prop-2-en-1-one 21b: Preparation from 4-nitroacetophenone (5.02 mmol, 829.1 mg) and 9-anthracenecarboxaldehyde (5.02 mmol, 1.035 g) according to the general

procedure above; red crystals (78%), Mp. 159-162 °C [2]. ^{1}H NMR (400 MHz, $CDCl_3$) δ 7.48 - 7.56 (m, 5 H, 4x ArH, 1x C=CH), 8.01 - 8.07 (m, 2 H, ArH), 8.18 - 8.24 (m, 2 H, ArH), 8.24 - 8.30 (m, 2 H, ArH), 8.33 - 8.38 (m, 2 H, ArH), 8.51 (br.s, 1 H, ArH), 8.87 (d, *J*=15.34 Hz, 1 H, C=CH). ^{13}C NMR (101 MHz, $CDCl_3$) ppm 123.93, 124.92, 125.5, 126.78, 129.05, 129.13, 129.58, 129.67, 130.01, 131.26, 142.57, 143.87, 188.08 (C=O). IR *ν* max (KBr): 3104.08, 3081.04, 3048.01, 2981.55 (Ar C-H), 1659.87 (C=O), 1624.09 (*trans* C=C), 1598.52, 1582.22, 1441.80, 1407.66 cm (Ar C=C), 1523.92, 1344.05 (Ar NO_2) cm^{-1}.

(*E*)-3-(Anthracen-9-yl)-1-(4-ethylphenyl)prop-2-en-1-one 21c: Preparation from 4- ethylacetophenone (5.02 mmol, 744 mg) and 9-anthracenecarboxaldehyde (5.02 mmol, 1.035 g) according to the general procedure above; yellow/orange crystals (61%) Mp. 128-129 °C [3]. ^{1}H NMR (400 MHz, $CDCl_3$) δ 1.27 (t, *J*=7.67 Hz, 3 H, CH_3) 2.73 (q, *J*=7.60 Hz, 2 H,CH_2) 7.33 (d, *J*=8.29 Hz, 2 H) 7.47 - 7.57 (m, 5H, 4 x ArH , 1xC=CH),7.98 - 8.05 (m, 4 H, ArH) 8.27 - 8.34 (m, 2 H, ArH) 8.45 (br.s, 1 H, ArH) 8.77 (d, *J*=16.17 Hz, 1 H,C=CH). ^{13}C NMR (101 MHz, $CDCl_3$) ppm 15.20, 28.99, 125.35, 125.40, 126.34, 128.27, 128.87, 128.96, 129.61, 130.33, 131.13, 131.30, 135.56, 141.40, 150.16, 189.19 (C=O) IR *ν* max (KBr): 3318.10, 3048.93, 3081.76, 2970.29, 2883.67 (Ar C-H), 1658.74 (C=O), 1622.32 (*trans* C=C), 1591.76, 1518.23, 1441.48, 1409.24 (Ar C=C), 1463.79 (CH_2), 1376.63 (CH_3) cm^{-1}

(*E*)-3-(Anthracen-9-yl)-1-(2,4-dichlorophenyl)prop-2-en-1-one 21d: Preparation from 2',4'-dichloroacetophenone (5.02 mmol, 949 mg) and 9-anthracenecarboxaldehyde (5.02 mmol, 1.035 g) according to the general procedure above; yellow solid (75%) [4]. Mp. 142-143 °C . ^{1}H NMR (400 MHz, $CDCl_3$) δ 7.14 (d, *J*=16.17 Hz, 1 H, C=CH), 7.41 (dd, *J*=8.29, 1.66 Hz, 1 H, ArH,) 7.46 - 7.56 (m, 5 H, ArH), 7.61 (d, *J*=8.29 Hz, 1 H, ArH), 7.99 - 8.04 (m, 2 H, ArH), 8.20 - 8.26 (m, 2 H,ArH), 8.48 (br.s, 1 H, ArH), 8.52 (d, *J*=16.59 Hz, 1 H, C=CH). ^{13}C NMR (101 MHz, $CDCl_3$) ppm 124.98, 125.46, 126.66, 128.95, 128.97, 129.05, 129.52, 130.29, 130.67, 131.24, 134.38, 137.33, 143.64, 191.96 (C=O). IR *ν* max (KBr): 3085.39, 3050.17 (Ar C-H), 1622.99 (*trans* C=C), 1663.97, 1588.07, 1556.09, 1465.62, 1013.77 (Ar-Cl) cm^{-1}.

(*E*)-3-(Anthracen-9-yl)-1-phenylprop-2-en-1-one 21f: Preparation from acetophenone (5.02 mmol, 0.59 mL) and 1.035 g of 9-anthracenecarboxaldehyde (5.02 mmol, 1.035 g) according to the general procedure above; yellow crystals (44 %), Mp. 128 – 130 °C [5]. ^{1}H NMR (400 MHz, $CDCl_3$) δ 8.76 - 8.84 (m, 1 H), 8.81 (d, *J*=15.76 Hz, 1 H), 8.44 (s, 1 H,) 8.28 - 8.34 (m, 2 H), 8.08 - 8.13 (m, 2 H), 7.98 - 8.04 (m, 2 H), 7.56 - 7.65 (m, 2 H), 7.47 -7.56 (m, 6 H). ^{13}C NMR (101 MHz, $CDCl_3$) ppm 125.29, 125.43, 126.44, 128.45, 128.64 - 129.03, 129.63, 130.14, 131.02, 131.31, 137.91, 141.89, 189.65 (C=O) . IR *ν* max (KBr): 3057.23 (Ar C-H), 1662.94 (Alkene C=C), 1604.17 (Ar C=C) cm^{-1}.

(*E*)-3-(Anthracen-9-yl)-1-(4-iodophenyl)prop-2-en-1-one 21g: Preparation from 4- iodoacetophenone (5.02 mmol, 1.235 g) and 9-anthracenecarboxaldehyde (5.02 mmol, 1.035 g), according to the general procedure above; orange crystals (80%), Mp. 167-173 °C [6]. ^{1}H NMR (400 MHz, $CDCl_3$) δ 7.44 - 7.54 (m, 5 H, 4x ArH, 1xC=CH), 7.75 - 7.80 (m, 2 H, ArH), 7.84 - 7.89 (m, 2 H, ArH), 7.99 - 8.05 (m, 2 H, ArH), 8.24 - 8.29 (m, 2 H, ArH), 8.46 (br.s, 1 H, ArH), 8.80 (d, *J*=16.17 Hz, 1 H, C=CH). ^{13}C NMR (101 MHz, $CDCl_3$) ppm 101.06, 125.17, 125.46, 126.54, 128.65, 128.96, 129.63, 130.06, 129.86, 130.33, 131.28, 137.12, 138.05, 142.46, 188.80 (C=O). IR$_{Vmax}$ (ATR): 3045.07 (Ar C-H), 1593.90 (C=O), 1618.99 (*trans* C=C) 1658.74, 1577.36, 1515.35, 1439.81 (Ar C=C), 669.23 (C-I) cm^{-1}.

(*E*)-3-(Anthracen-9-yl)-1-(4-fluorophenyl)prop-2-en-1-one 21h: Preparation from 4-fluoroacetophenone (5.02 mmol, 0.61 mL) and 9-anthracenecarboxaldehyde (5.02 mmol, 1.035 g), according to the general procedure above; yellow crystals (88%), Mp. 142 °C [6]. ^{1}H NMR (400 MHz, $CDCl_3$) δ 7.15 - 7.22 (m, 2 H, ArH), 7.47 - 7.55 (m, 5 H, 4x ArH, 1x C=CH), 8.00 - 8.04 (m, 2H, ArH), 8.08 - 8.14 (m, 2H, ArH), 8.26 - 8.31 (m, 2 H, ArH), 8.46 (br.s, 1 H, ArH), 8.80 (d, *J*=16.17 Hz, 1H,C=CH). ^{13}C NMR (101 MHz, $CDCl_3$) ppm 115.77, 115.99, 125.20, 125.43, 126.47, 128.51, 128.93, 129.61, 130.52, 131.27, 131.28, 131.36, 134.24, 142.13, 164.50, 167.03, 187.95 (C=O).

(*E*)-3-(Anthracen-9-yl)-1-(4-methoxyphenyl)prop-2-en-1-one 21i: Preparation from 4-methoxyacetophenone (5.02 mmol, 0.754 g) and 9-anthracenecarboxaldehyde (5.02 mmol, 1.035 g) according to the general procedure above; yellow powder (58%), Mp. 122-125 °C [5]. ^{1}H NMR (400 MHz, $CDCl_3$) δ 3.89 (s, 3H) 7.00 (d, *J*=8.71 Hz, 2H) 7.47 -7.59 (m, 5 H) 8.01 - 8.06 (m, 2H) 8.07 - 8.12 (m, 2H) 8.29 - 8.35 (m, 2H) 8.47 (s, 1H) 8.78 (d, *J*=15.76 Hz, 1H).^{13}C NMR (101 MHz, $CDCl_3$) ppm 55.50, 113.98, 125.40, 126.31, 128.19, 128.86, 129.61, 130.48, 130.73 - 131.17, 131.31, 141.01, 163.65, 187.90 (C=O). IR *ν* max (KBr): 3045.86 (Ar C-H), 1654.13 (C=O), 1593.97 (Ar C=C), 1254.32 (C-O) cm^{-1}.

(*E*)-3-(Anthracen-9-yl)-1-(*p*-tolyl)prop-2-en-1-one 21j: Preparation from 4- methylacetophenone (5.02 mmol, 0.67 mL) and 9-anthracenecarboxaldehyde (5.02 mmol, 1.035 g) according to the general procedure above; yellow powder (61%), Mp. 98-103 °C [5]. ^{1}H NMR (400 MHz, $CDCl_3$) δ 2.45 (s, 3H), 7.32 (d, *J*=8.29 Hz, 2H), 7.48 -7.59 (m, 5H), 7.96 - 8.09 (m, 4H), 8.29 - 8.36 (m, 2H), 8.47 (s, 1H), 8.79 (d, *J*=15.76 Hz, 1H) ^{13}C NMR (101 MHz, $CDCl_3$) ppm: 28.34, 124.83, 125.36, 125.81 - 126.41, 126.90, 128.05 - 129.12, 129.62, 130.09 - 130.73, 131.19 - 131.90, 134.46, 135.11 - 135.51, 138.33, 139.35, 142.48, 143.98, 146.79, 188.50 (C=O). IR *ν* max (KBr): 3046.66 (Ar C-H), 1657.95 (C=O), 1592.79 (Ar C=C), 1441.79 (C-CH_3) cm^{-1}.

(*E*)-3-Anthracen-9-yl)-1-(4-chorophenyl)prop-2-en-1-one 21l: Preparation from 4- chloroacetophenone (5.02 mmol, 0.65 mL) and of 9-anthracenecarboxaldehyde (5.02 mmol, 1.035 g) according to the general procedure above; yellow crystals (65%), Mp. 142 – 145 °C [2]. ^{1}H NMR (400 MHz, $CDCl_3$) δ 7.46 - 7.56 (m, 7 H) 8.00 - 8.06 (m, 4 H) 8.26 -8.32 (m, 2 H) 8.48 (s, 1 H) 8.81 (d, *J*=15.76 Hz, 1 H). ^{13}C NMR (101 MHz, $CDCl_3$) ppm: 125.18, 125.46, 126.53, 128.63, 128.96, 129.08, 129.63, 129.88, 130.10, 130.41, 131.28, 136.17, 139.56, 142.44, 188.31 (C=O). IR *ν* max (KBr): 3048.63 (Ar C-H), 1660.22 (C=O), 1596.31 (Ar C=C), 731.61 (C-Cl) cm^{-1}.

(*E*)-3-(Anthracen-9-yl)-1-(pyridin-4-yl)prop-2-en-1-one 21m: Preparation from 4-acetylpyridine (5.02 mmol, 0.55 mL) and 9-anthracenecarboxaldehyde (5.02 mmol, 1.035 g), according to the general procedure above; orange crystals (49%), Mp. 170-173 °C [7]. ^{1}H NMR (400 MHz, $CDCl_3$) δ 7.44 - 7.48 (d, 1H, CH=C), 7.49 - 7.56 (m, 3H, ArH), 7.80 - 7.85 (m, 2H, ArH), 7.98 - 8.05 (m, 2H, ArH), 8.21 - 8.28 (m, 2H, Ar H), 8.46 (br.s, 1H), 8.80 - 8.87 (m, 3H, ArH, C=CH). ^{13}C NMR (101 MHz, $CDCl_3$) ppm 121.57, 124.92, 125.49, 126.75, 129.03 129.09, 129.64, 129.84, 131.23, 143.86, 143.90, 150.94, 188.94 (C=O). IR *ν* max (KBr): 3047.23, 2971.86 (Ar C-H), 1587 (C=O), 1621.69 (*trans* C=C), 1661. 62, 1597.45, 1518.42, 1441.76 (Ar C=C), 1266.30 (C-N) cm^{-1}.

(*E*)-3-(Anthracen-9-yl)-1-(pyridin-2-yl)prop-2-en-1-one 21n : Preparation from 2-acetylpyridine (5.02 mmol, 0.56 mL) and 9-anthracenecarboxaldehyde (5.02 mmol, 1.035 g), according to the general procedure above; green solid (58%), Mp. 97-101 °C [5]. ^{1}H NMR (400 MHz, $CDCl_3$) δ 7.44 - 7.53 (m, 5H, ArH, CH=C), 7.90 (td, *J*=7.78, 1.87 Hz, 1 H, ArH), 7.99 - 8.04 (m, 2H, ArH), 8.23 - 8.30 (m, 2H, ArH), 8.33 - 8.38 (m, 2H, ArH), 8.46 (br.s, 1H, ArH), 8.66 - 8.70 (m, 1H, ArH), 8.92 (d, *J*=16.17 Hz, 1H, C=CH). ^{13}C NMR (101 MHz, $CDCl_3$) ppm 123.1, 125.4, 125.5, 126.4, 127.0, 128.5, 128.9, 130.2, 137.1, 137.9, 141.8, 149.1, 172.4 (chalcone C=O).

(*E*)-3-(Anthracen-9-yl)-1-(furan-2-yl)prop-2-en-1-one 21o: Preparation from 2-acetylfuran (5.02 mmol, 552.8 mg) and 9-anthracenecarboxaldehyde (5.02 mmol, 1.035 g), according to the general procedure above; yellow crystals (67%), Mp. 148-150 °C [8]. ^{1}H NMR (400 MHz, $CDCl_3$) δ 6.60 (dd, *J*=3.73, 1.66 Hz, 1 H, C=CH), 7.32 - 7.36 (m, 1 H, CH=C), 7.42 (d, *J*=16.17 Hz, 1 H, CH=C), 7.47 - 7.54 (m, 4 H, ArH), 7.64 - 7.68 (m, 1 H, CH=C), 7.99 - 8.05 (m, 2 H, ArH), 8.26 - 8.32 (m, 2H, ArH), 8.46 (br.s, 1 H, ArH), 8.84 (d, *J*=15.76 Hz, 1H, CH=C). ^{13}C NMR (101 MHz, $CDCl_3$) ppm 112.61, 118.13, 125.28, 125.40, 126.41, 128.48, 128.88, 129.64, 129.93, 130.27, 131.28, 141.14, 146.92, 153.59, 177.39 (C=O). IR *ν* max (KBr): 3082, 3048.70 (Ar C-H), 1587.15 (C=O), 1622.50 (*trans* C=C), 1663.59, 1555.20, 1518.79, 1441.13 (Ar C=C), 1013.08 (C-O) cm^{-1}.

(*E*)-3-(Anthracen-9-yl)-1-(naphthalen-2-yl)prop-2-en-1-one 21p: Preparation from 1-(naphthalen-2-yl)ethan-1-one (5.02 mmol, 0.855 g) and 9-anthracenecarboxaldehyde (5.02 mmol, 1.035 g), according to the general procedure above; yellow crystals (38%), Mp. 160-164 °C [9]. ^{1}H NMR (400 MHz, $CDCl_3$) δ 7.47 - 7.56 (m, 5 H, ArH) 7.57 - 7.63 (m, 1 H, ArH) 7.72 (d, *J*=15.76 Hz, 1 H, CH=C) 7.87 - 7.98 (m, 3 H, ArH) 8.00 - 8.06 (m, 2 H, ArH) 8.19 (dd, *J*=8.71, 1.66 Hz, 1 H,ArH) 8.31 - 8.37 (m, 2 H, ArH) 8.45 - 8.50 (m, 1 H, ArH) 8.56 (br.s, 1 H, ArH) 8.85 (d, *J*=15.76 Hz, 1 H,C=CH). ^{13}C NMR (101 MHz, $CDCl_3$) ppm 124.48,125.00, 125.29 - 125.48, 125.89, 126.21, 126.44 ,126.84 ,127.09 ,127.83, 128.03 - 128.05, 128.42, 128.55, 128.67 - 128.76, 128.83 - 128.97. 129.14,129.58, 129.66, 130.37, 131.01 - 131.12, 131.32, 132.56, 134.54, 135.07, 135.21, 135.60, 138.95, 141.80, 189.41 (C=O).

(*E*)-3-(Anthracen-9-yl)-1-(thiophen-2-yl)prop-2-en-1-one 21q: Preparation from 2-acetylthiophene (5.02 mmol, 0.54 mL) and 9-anthracenecarboxaldehyde (5.02 mmol, 1.035 g), according to the general procedure above; yellow crystals (86%), Mp. 157-158 °C [6]. ^{1}H NMR (400 MHz, $CDCl_3$) δ 7.18 (dd, *J*=4.98, 3.73 Hz, 1 H,C=CH), 7.42 (d, *J*=15.76 Hz, 1 H, C=CH), 7.47 - 7.54 (m, 4 H, ArH), 7.72 (dd, *J*=4.77, 1.04 Hz, 1 H,C=CH), 7.82 (dd, *J*=3.94, 1.04 Hz, 1 H,(C=CH), 8.00 - 8.06 (m, 2 H, ArH), 8.26 - 8.32 (m, 2 H, ArH), 8.47 (br.s, 1 H, ArH), 8.81 (d, *J*=15.76 Hz, 1 H,C=CH). ^{13}C NMR (101 MHz, $CDCl_3$) ppm 125.27, 125.43, 126.44, 128.37, 128.45, 128.89, 129.63, 129.93, 130.71, 131.28, 132.27, 134.37, 141.21, 145.41, 181.48 (C=O).

Table S1: Crystal data and structure refinement for 21k, 23f, 23h, 24a, 24g, 25f and 27

CCDC No.	2341799	2341800	2341801	2341802	2341802	2341804	2341805
Identification code	**21k**	**23f**	**23h**	**24a**	**24g**	**25f**	**27**
Empirical formula	$C_{26}H_{22}O_4$	$C_{27}H_{19}NO_3$	$C_{27}H_{18}FNO_3$	$C_{33}H_{22}BrNO_3$	$C_{33}H_{22}INO_3$	$C_{33}H_{22}ClNO_3$	$C_{29}H_{22}O_5$
Formula weight	398.43	405.43	423.42	560.42	607.41	515.96	450.46
Temperature (K)	100(2)	100(2)	100(2)	100(2)	100(2)	100(2)	100(2)
Crystal system	monoclinic	monoclinic	triclinic	monoclinic	monoclinic	monoclinic	monoclinic
Space group	$P2_1/c$	$P2_1/c$	P-1	$P2_1/c$	$P2_1/n$	Cc	$P2_1/c$
a (Å)	29.099(3)	20.1992(16)	6.5671(7)	13.5908(4)	13.7687(3)	11.0462(5)	18.9744(16)
b (Å)	5.4348(5)	6.5975(5)	8.1003(8)	8.0761(2)	7.9215(2)	30.0538(14)	8.7405(7)
c (Å)	12.6086(11)	15.2217(11)	19.914(3)	23.2409(6)	23.9185(6)	8.1065(4)	14.7391(12)
α (°)	90	90	82.445(5)	90	90	90	90
β (°)	98.582(3)	109.811(2)	82.163(6)	106.0230(10)	105.7027(12)	113.531(3)	111.108(3)
γ (°)	90	90	75.998(4)	90	90	90	90
Volume (Å^3)	1971.7(3)	1908.5(3)	1012.9(2)	2451.83(11)	2511.40(11)	2467.4(2)	2280.4(3)
Z	4	4	2	4	4	4	4
ρ_{calc} (g/cm^3)	1.342	1.411	1.388	1.518	1.606	1.389	1.312
μ (mm^{-1})	0.090	0.092	0.097	1.715	1.312	1.671	0.726
F(000)	840.0	848.0	440.0	1144.0	1216.0	1072.0	944.0
Crystal size (mm^3)	0.26 × 0.13 × 0.04	0.22 × 0.13 × 0.06	0.29 × 0.19 × 0.06	0.23 × 0.15 × 0.12	0.23 × 0.21 × 0.2	0.15 × 0.11 × 0.05	0.36 × 0.17 × 0.14
Radiation	MoKα (λ = 0.71073)	MoKα (λ = 0.71073)	Mo Kα (λ = 0.71073)	MoKα (λ = 0.71073)	MoKα (λ = 0.71073)	CuKα (λ = 1.54178)	CuKα (λ = 1.54178)
Reflections collected	18897	20477	15760	65657	58595	35493	27728
Independent reflections	3698 R_{int} = 0.0772 R_{sigma} = 0.0583	4272 R_{int} = 0.0765 R_{sigma} = 0.0533	3942 R_{int} = 0.0433 R_{sigma} = 0.0402	4872 R_{int} = 0.0860 R_{sigma} = 0.0369	8049 R_{int} = 0.0461 R_{sigma} = 0.0304	3970 R_{int} = 0.0969 R_{sigma} = 0.0469	4310 R_{int} = 0.0438 R_{sigma} = 0.0267
Data/restraints/parameters	3698/0/274	4272/1/280	3942/121/286	4872/1044/566	8049/0/343	3970/2/343	4310/0/310
Goodness-of-fit on F^2	1.122	1.043	1.134	1.063	0.913	1.104	1.057
Final R* indexes [I≥2σ (I)]	R_1 = 0.0754 wR_2 = 0.1683	R_1 = 0.0508 wR_2 = 0.1051	R_1 = 0.0787 wR_2 = 0.2257	R_1 = 0.0388 wR_2 = 0.0758	R_1 = 0.0356 wR_2 = 0.1092	R_1 = 0.0654 wR_2 = 0.1758	R_1 = 0.0373 wR_2 = 0.1007
Final R indexes [all data]	R_1 = 0.1046 wR_2 = 0.1803	R_1 = 0.0872 wR_2 = 0.1223	R_1 = 0.0894 wR_2 = 0.2324	R_1 = 0.0622 wR_2 = 0.0832	R_1 = 0.0498 wR_2 = 0.1197	R_1 = 0.0740 wR_2 = 0.1844	R_1 = 0.0395 wR_2 = 0.1027
Largest diff. peak/hole / e Å^{-3}	0.28/-0.33	0.23/-0.30	0.56/-0.48	0.35/-0.45	1.59/-1.35	0.56/-0.30	0.22/-0.21
Flack parameter	-	-	-	-	-	-0.04(2)	-

*$R_1 = \sum ||F_o| - |F_c|| / \sum |F_o|$, $wR_2 = [\sum w(F_o^2 - F_c^2)^2 / \sum w(F_o^2)^2]^{1/2}$.

Table S2: Stability study for compounds **21a, 21i, 22h, 23a, 23g, 23n, 24a, 24h, 26a,** and **26n** at pH 4.0, pH 7.5 and pH 9.0 over 24 h[a]

Compound	% remaining 24 h pH 4	% remaining 24 h pH 7.4	% remaining 24 h pH 9	Half-life (h) pH 4	Half-life (h) pH 7.4	Half-life (h) pH 9
**21a**	46.27	38.59	38.61	3.25	2	1.5
**21i**	27.85	26.64	42.07	~11.5	3.85	~11.5
**22h**	0.81	3.13	n/a	2.1	1	n/a
**23a**	3.12	1.38	0.48	13.3	~7.5	~7.5
**23g**	7.98	11.02	10.50	11.3	8.9	5.5
**23n**	38.86	70.29	88.59	19	>24	>24
**24a**	48.41	45.23	33.58	4.17	23.5	23.5
**24h**	24.97	79.48	80.99	14.8	>24	>24
**26a**	63.95	61.03	66.0	>24	>24	>24
**26n**	10.97	40.39	51.20	8.5	16.5	>24

[a]All samples were analysed using acetonitrile - water (80:20%, 70:30% , 60:40% isocratic) as the mobile phase over 10 min and a flow rate of 1 mL/min. Stock solutions are prepared by dissolving 5 mg of compound in mobile phase (10 mL). Anhydride compound **22h** was not detected at its retention time at 5.2 minutes at pH 9.0. Data presented represents the results of single experiments.

Table S3: Top five ranking compounds with **25n**according to NCI-COMPARE Pearson correlation coefficient analysis of GI_{50} profiles[a]

Rank	Compound	Correlation
**1**	Anguidine	0.422
**2**	Bleomycin	0.37
**3**	3-HP	0.364
**4**	O6-Methylguanine	0.36
**5**	4-Ipomeanol	0.344

[a]The target set was the standard agent database and the target set endpoints were selected to be equal to the seed end points. Standard COMPARE analysis was performed. Correlation values (r) are Pearson correlation coefficients.

Table S4: Top five ranking compounds with **23n** according to NCI-COMPARE Pearson correlation coefficient analysis GI_{50} profiles[a]

Rank	Compound	Correlation
**1**	Dichloroallyl lawsone	0.505
**2**	Anguidine	0.496
**3**	Triazinate	0.496
**4**	Tamoxifen	0.488
**5**	Diglycoaldehyde	0.486

[a]The target set was the standard agent database and the target set endpoints were selected to be equal to the seed end points. Standard COMPARE analysis was performed. Correlation values (r) are Pearson correlation coefficients.

Table S5: Top five ranking compounds with **23h** according to NCI-COMPARE Pearson correlation coefficient analysis GI $_{50}$ profiles[a]

Rank	Compound	Correlation
**1**	2-Deoxycoformycin	0.918
**2**	Caracemide	0.663
**3**	Fluorodopan	0.631
**4**	Hydroxyurea	0.587
**5**	Melphalan	0.538

[a]The target set was the standard agent database and the target set endpoints were selected to be equal to the seed end points. Standard COMPARE analysis was performed. Correlation values (r) are Pearson correlation coefficients.

Table S6: Top five ranking compounds with **24l** according to NCI-COMPARE Pearson correlation coefficient analysis of GI_{50} profiles[a]

Rank	Compound	Correlation
**1**	Fluorodopan	0.631
**2**	Pyrimidine-5-glycoaldehyde	0.531
**3**	Hydroxyurea	0.512
**4**	Glyoxylic acid	0.505
**5**	(Carboxyphthalato)platinum	0.496

[a]The target set was the standard agent database and the target set endpoints were selected to be equal to the seed end points. Standard COMPARE analysis was performed. Correlation values (r) are Pearson correlation coefficients.

Table S7: Physicochemical descriptors and mean IC_{50} values (CLL) of selected ethanoanthracene compounds (**20a-e**, **23a, 23c, 23d**, **23f-I, 23k-p**, **24f**, **24l, 25n, 26n, 27**) [a].

Cpd No.	MW	Molar Refractivity (m^3/mol)	Num. HBD	Num. HBA	Num. Rot Bonds	TPSA (Å^2)	Log P (consensus)[c]	Water Solubility (consensus) (mg/mL)[d]	GI Absorption[b]	Mean IC_{50} (µM) CLL
**20a**	422.43	123.29	0	4	3	83.20	3.29	1.93x10^{-3}	High	0.55
**20b**	456.88	128.30	0	4	3	83.20	3.81	0.509x10^{-3}	High	0.76
**20c**	456.88	127.97	0	4	3	83.20	3.54	1.102 x10^{-3}	High	0.8
**20d**	346.34	98.15	1	4	2	91.99	1.86	61.4 x10^{-3}	High	0.77
**20e**	302.33	88.09	0	3	2	69.61	2.98	16.88 x10^{-3}	High	1.4
**23a**	484.34	127.46	1	3	3	63.24	4.35	0.973 x10^{-3}	High	2.33
**23c**	433.50	129.53	1	3	4	63.24	4.36	1.035 x10^{-3}	High	3.67
**23d**	474.33	129.78	1	3	3	63.24	4.76	0.321 x10^{-3}	High	2.33
**23f**	405.44	119.76	1	3	3	63.24	3.73	4.97 x10^{-3}	High	7.43
**23g**	531.34	132.48	1	3	3	63.24	4.38	0.98 x10^{-3}	High	3.62
**23h**	423.44	119.72	1	4	3	63.24	4.05	3.88 x10^{-3}	High	3.77
**23i**	435.47	126.25	1	4	4	72.47	3.72	4.01 x10^{-3}	High	11.4
**23k**	495.52	139.24	1	6	6	90.93	3.66	2.62 x10^{-3}	High	5.19
**23l**	439.89	124.77	1	3	3	63.24	4.27	1.26 x10^{-3}	High	2.72
**23m**	406.43	117.56	1	4	3	76.13	3.00	29.8 x10^{-3}	High	3.62
**23n**	406.43	117.56	1	4	3	76.13	3.03	15.1 x10^{-3}	High	1.94
**23o**	395.41	112.03	1	4	3	76.38	3.11	12.3 x10^{-3}	High	3.67
**23p**	455.50	137.27	1	3	3	63.24	4.62	0.335 x10^{-3}	High	1.36
**24f**	481.54	144.90	0	3	4	54.45	3.69	0.151 x10^{-3}	High	7.24
**24l**	515.99	149.91	0	3	4	54.45	5.66	0.038 x10^{-3}	High	1.88
**25n**	516.97	147.71	0	4	4	67.34	4.98	0.011 x10^{-3}	High	1.09
**26n**	586.63	172.57	0	5	6	84.41	5.51	0.015 x10^{-3}	High	1.12
**27**	450.48	126.47	0	5	7	69.67	4.58	0.628 x10^{-3}	High	-

[a]SwissADME http://www.swissadme.ch; [b]according to the white of the BOILED-Egg; [c]Average of all five predictions; [d] Solubility class: Log S scale: Insoluble < -10 < Poorly < -6 < Moderately < -4 < Soluble < -2 Very < 0 < Highly

Table S8: Pharmacokinetic interaction estimations with P-gp and CYP 450 isozymes of selected compounds (**20a-e**, **23a, 23c, 23d**, **23f-I, 23k-p**, **24f**, **24l, 25n, 26n, 27**)[a]

Compound Number	P-gp substrate	CYP1A2 Inhibitor	CYP2C19 inhibitor	CYP2C9 inhibitor	CYP2D6 Inhibitor	CYP3A4 Inhibitor	Skin permeation Coefficient (cm/s)	BBB permeable[b]	Abbott Bioavailability Score[c]
**20a**	No	No	Yes	Yes	No	Yes	-6.00	No	0.55
**20b**	No	No	Yes	Yes	No	No	-5.77	No	0.55
**20c**	No	No	Yes	Yes	No	Yes	-6.08	No	0.55
**20d**	Yes	Yes	Yes	Yes	No	No	-6.77	No	0.55
**20e**	Yes	No	Yes	Yes	No	No	-5.60	Yes	0.55
**23a**	No	No	Yes	Yes	No	No	-6.05	Yes	0.55
**23c**	No	No	Yes	Yes	No	No	-5.66	Yes	0.55
**23d**	No	No	Yes	Yes	No	No	-5.59	Yes	0.55
**23f**	No	No	Yes	Yes	No	No	-6.06	Yes	0.55
**23g**	No	No	Yes	Yes	No	No	-6.37	Yes	0.17
**23h**	No	No	Yes	Yes	No	No	-6.10	Yes	0.55
**23i**	No	No	Yes	Yes	No	Yes	-6.27	Yes	0.55
**23k**	Yes	No	Yes	Yes	No	Yes	-6.27	No	0.55
**23l**	No	No	Yes	Yes	No	No	-5.82	Yes	0.55
**23m**	No	Yes	Yes	Yes	No	No	-6.83	Yes	0.55
**23n**	No	Yes	Yes	Yes	No	No	-6.59	Yes	0.55
**23o**	No	Yes	Yes	Yes	No	No	-6.43	Yes	0.55
**23p**	No	No	No	Yes	No	No	-5.48	Yes	0.55
**24f**	No	No	No	Yes	No	No	-5.29	Yes	0.55
**24l**	No	No	No	Yes	No	No	-5.05	No	0.17
**25n**	No	No	Yes	Yes	No	No	-5.58	No	0.17
**26n**	No	No	No	Yes	No	No	-5.50	No	0.17
**27**	No	No	Yes	Yes	No	Yes	-5.53	Yes	0.55

[a] SwissADME: a free web tool to evaluate pharmacokinetics, drug-likeness and medicinal chemistry friendliness of small molecules http://www.swissadme.ch; [b]According to the yolk of the BOILED-Egg; [c]Probability of F > 10% in rat 0.55

Table S9: Drug-likeness of representative compounds assessed *via* leadlikeness and reactivity/toxicity filters for selected compounds (**20a-e**, **23a, 23c, 23d**, **23f-I, 23k-p, 24f, 24l, 25n, 26n, 27**)[a,d]

Compound Number	Lipinski	Ghose	Veber	Egan	Muegge	PAINS[b]	Brenk[c]
**20a**	Yes; 0 violation	Yes	Yes	Yes	Yes	0 alerts	3 alerts
**20b**	Yes; 0 violation	Yes	Yes	Yes	Yes	0 alerts	3 alerts
**20c**	Yes; 0 violation	Yes	Yes	Yes	Yes	0 alerts	4 alerts
**20d**	Yes; 0 violation	Yes	Yes	Yes	Yes	0 alerts	3 alerts
**20e**	Yes; 0 violation	Yes	Yes	Yes	Yes	0 alerts	2 alerts
**23a**	Yes; 1 violation: MLOGP>4.15	No; 1 violation: MW>480	Yes	Yes	Yes	0 alerts	2 alerts
**23c**	Yes; 1 violation: MLOGP>4.15	Yes	Yes	Yes	Yes	0 alerts	2 alerts
**23d**	Yes; 1 violation: MLOGP>4.15	Yes	Yes	Yes	No; 1 violation: XLOGP3>5	0 alerts	2 alerts
**23f**	Yes; 0 violation	Yes	Yes	Yes	Yes	0 alerts	2 alerts
**23g**	No; 2 violations: MW>500, MLOGP>4.15	No; 2 violations: MW>480, MR>130	Yes	Yes	Yes	0 alerts	3 alerts
**23h**	Yes; 1 violation: MLOGP>4.15	Yes	Yes	Yes	Yes	0 alerts	2 alerts
**23i**	Yes; 0 violation	Yes	Yes	Yes	Yes	0 alerts	2 alerts
**23k**	Yes; 0 violation	No; 2 violations: MW>480, MR>130	Yes	Yes	Yes	0 alerts	2 alerts
**23l**	Yes; 1 violation: MLOGP>4.15	Yes	Yes	Yes	Yes	0 alerts	2 alerts
**23m**	Yes; 0 violation	Yes	Yes	Yes	Yes	0 alerts	2 alerts
**23n**	Yes; 0 violation	Yes	Yes	Yes	Yes	0 alerts	2 alerts
**23o**	Yes; 0 violation	Yes	Yes	Yes	Yes	0 alerts	2 alerts
**23p**	Yes; 1 violation: MLOGP>4.15	No; 1 violation: MR>130	Yes	Yes	No; 1 violation: XLOGP3>5	0 alerts	2 alerts
**24f**	Yes; 1 violation: MLOGP>4.15	No; 2 violations: MW>480, MR>130	Yes	Yes	No; 1 violation: XLOGP3>5	0 alerts	2 alerts
**24l**	No; 2 violations: MW>500, MLOGP>4.15	No; 3 violations: MW>480, WLOGP>5.6, MR>130	Yes	No; 1 violation: WLOGP>5.88	No; 1 violation: XLOGP3>5	0 alerts	2 alerts
**25n**	No; 2 violations: MW>500, MLOGP>4.15	No; 2 violations: MW>480, MR>130	Yes	Yes	No; 1 violation: XLOGP3>5	0 alerts	2 alerts
**26n**	No; 2 violations: MW>500, MLOGP>4.15	No; 4 violations: MW>480, WLOGP>5.6, MR>130, #atoms>70	Yes	No; 1 violation: WLOGP>5.88	No; 2 violations: XLOGP3>5, #rings>7	0 alerts	2 alerts
**27**	Yes; 0 violation	Yes	Yes	Yes	Yes	0 alerts	2 alerts

[a]SwissADME http://www.swissadme.ch [b]Pan Assay Interference Structures: (PAINS); [c]Brenk Structural Alert e.g. Michael acceptor, more than 2 esters; nitro group, oxygen-nitrogen single bond, phthalimide, alkyl halide; [d]Additional druglikeness assessment methods including Ghose, Veber and Muegge filters together with medicinal chemistry structure alert assessments PAINS (pan assay interference compounds) and Brenk filters were used to identify compounds which are potentially toxic, chemically reactive, metabolically unstable compounds or having poor pharmacokinetics.

Table S10: Overlay of ethanoanthracene compound series and nitrostyrene compound **18b** on maprotiline with their overlay scores[a]

Compound No	Overlay of compounds (green) with Maprotiline (pink)	Overlay (F) score	Overlay (S) score	Mean IC_{50} (µM)
**20a**	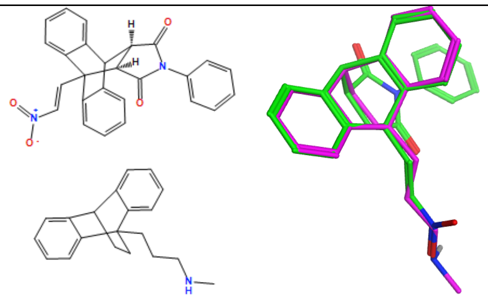	-157.66	-66.46	0.55
**20b**	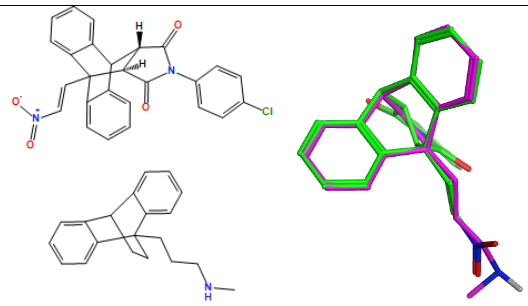	-158.37	-48.91	0.76
**20d**	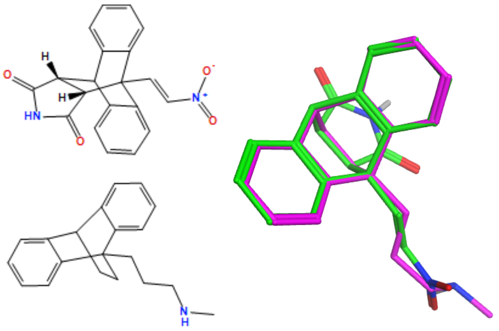	-156.30	-86.35	0.77
**20e**	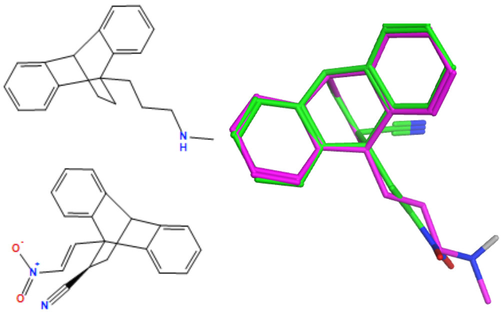	-153.63	-63.82	1.4

**23a**	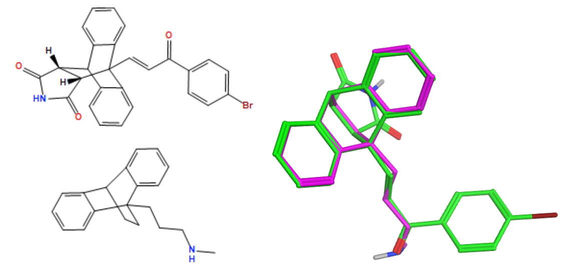	-165.19	-79.80	2.33
**23c**	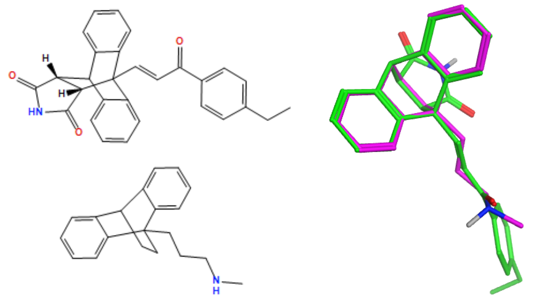	-164.14	-80.62	3.67
**23f**	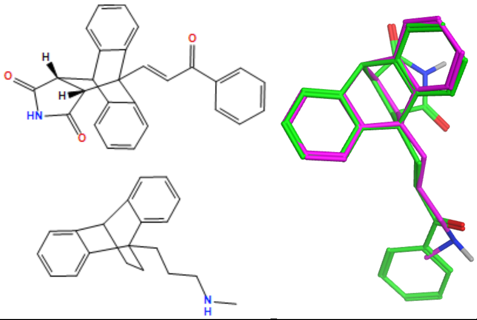	-164.43	-77.42	7.43
**23g**	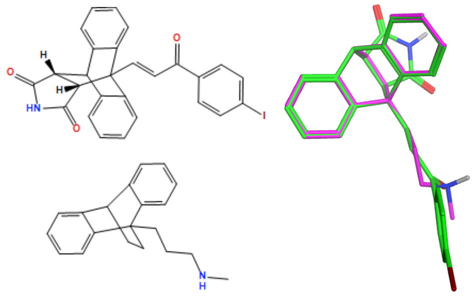	-166.39	-80.01	3.62
**23h**	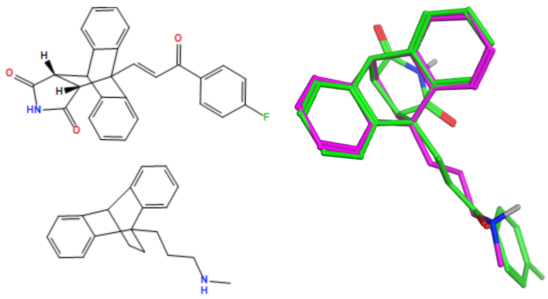	-163.77	-81.29	3.77

**23i**	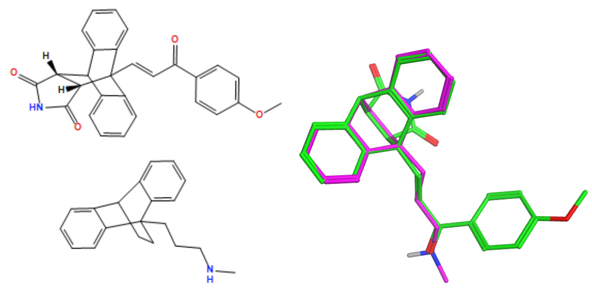	-164.10	-75.88	11.4
**23k**	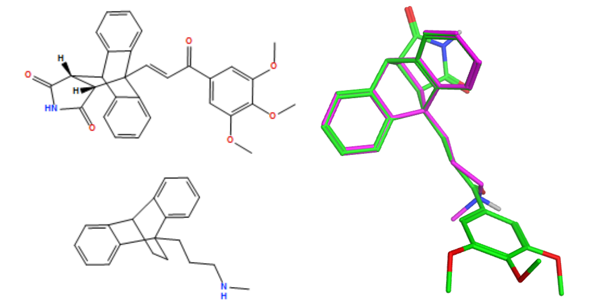	-167.56	-63.56	5.19
**23l**	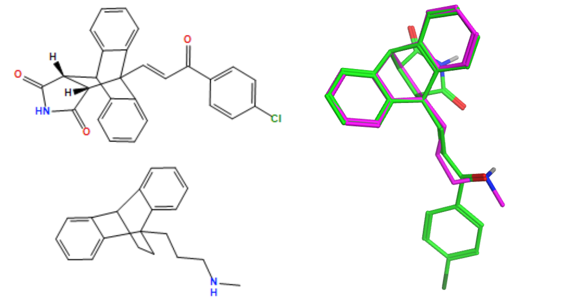	-165.47	-81.20	2.72
**23n**	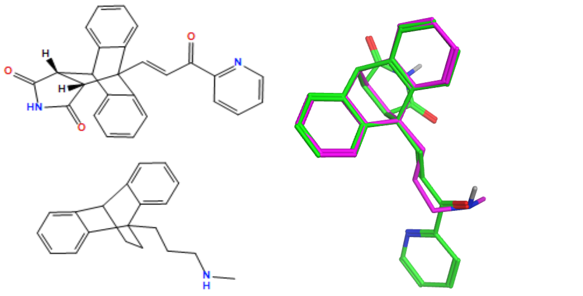	-165.52	-77.82	1.94
**23p**	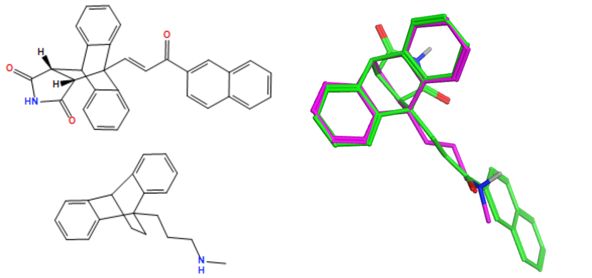	-163.76	-73.82	1.36

**24f**	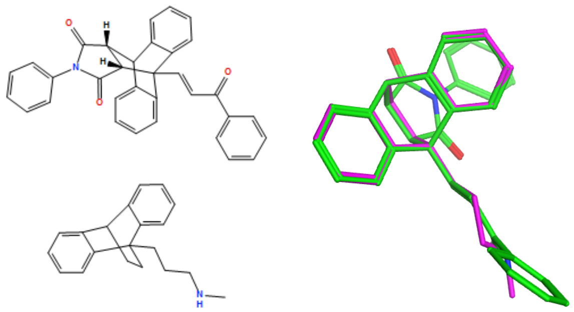	-167.06	-59.34	7.24
**24l**	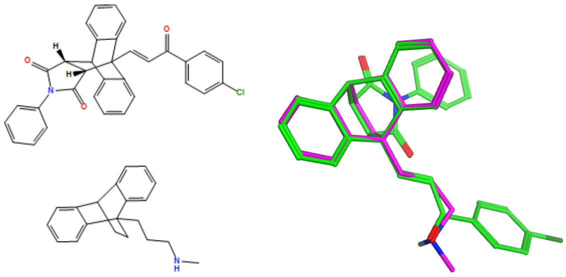	-165.68	-58.10	1.88
**25n**	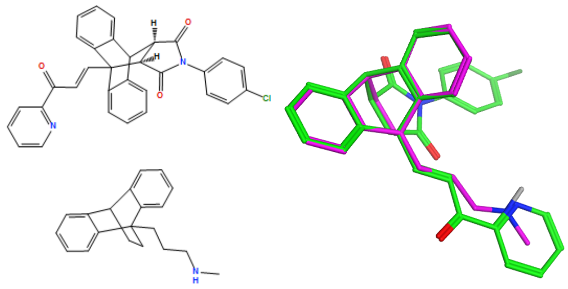	-169.14	-58.04	1.09
**26n**	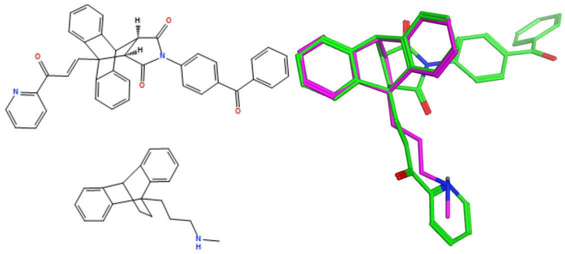	-168.68	-36.59	1.12

**20f**	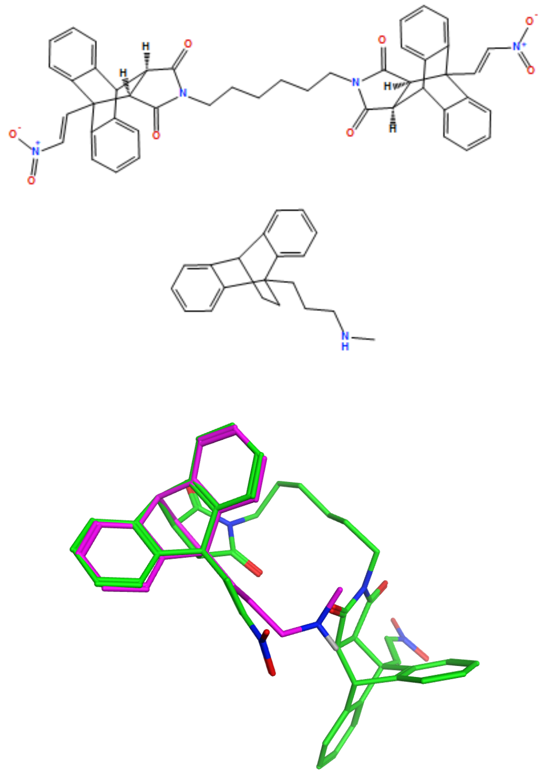	-163.50	-65.11	0.53
**Anthracene 18b**	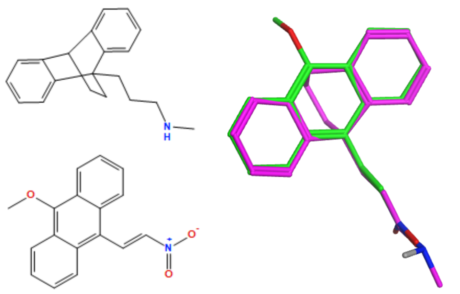	-132.50	-36.05	0.73

[a]Overlay of ethanoanthracene compounds series and nitrostyrene compound **18b** on maprotiline with their overlay scores (F and S) in MOE 2022, 2D images and mean IC_{50} values. The F column contains the similarity score (the lower the better) of the alignment. The S column has the sum of the U (not shown but is the average internal energy of the ligand) and F values.

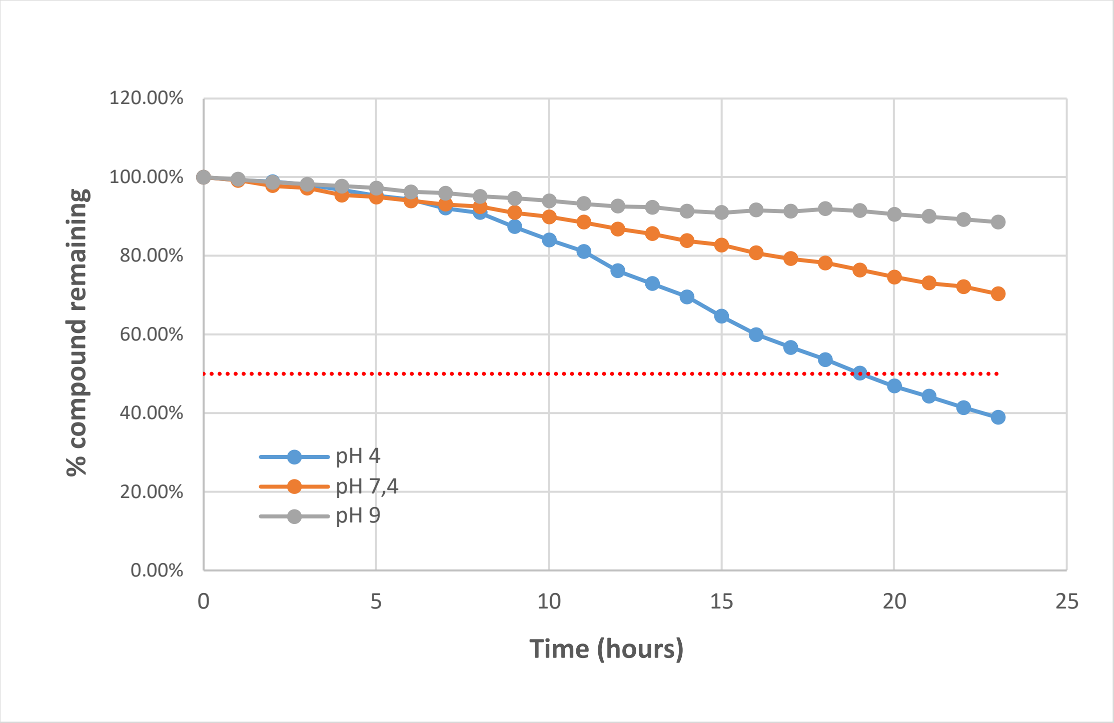

Figure S1: Stability testing of compound **23n** at pH 4.0, 7.4, and 9.0 over 24 h

Samples were analysed using acetonitrile - water (80:20%, 70:30%, 60:40% isocratic) as the mobile phase over 10 min and a flow rate of 1 mL/min. Stock solutions are prepared by dissolving 5 mg of compounds in mobile phase (10 mL). Data presented represents the results of single experiments.

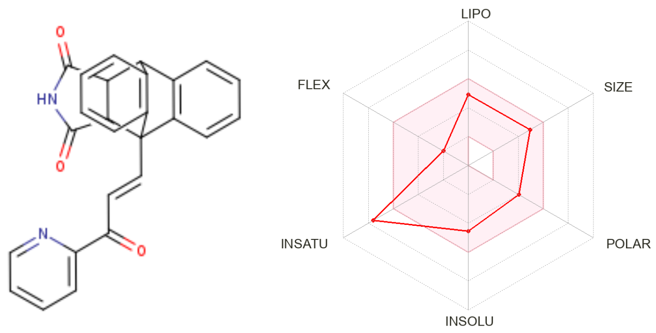

Figure S2: The *Bioavailability Radar* displayed for compound **23n**.

Six physicochemical properties are considered for analysis of drug-likeness: lipophilicity, size, polarity, solubility, flexibility and saturation. A physicochemical range on each axis is depicted as a pink area in which the radar plot of the molecule has to fall entirely to be considered drug-like. The pink area represents the optimal range for each properties (lipophilicity: XLOGP3 between −0.7 and +5.0, size: MW between 150 and 500 g/mol, polarity: TPSA between 20 and 130 Å^2, solubility: log S not higher than 6, saturation: fraction of carbons in the sp^3 hybridization not less than 0.25, and flexibility: no more than 9 rotatable bonds. (SwissADME http://www.swissadme.ch).

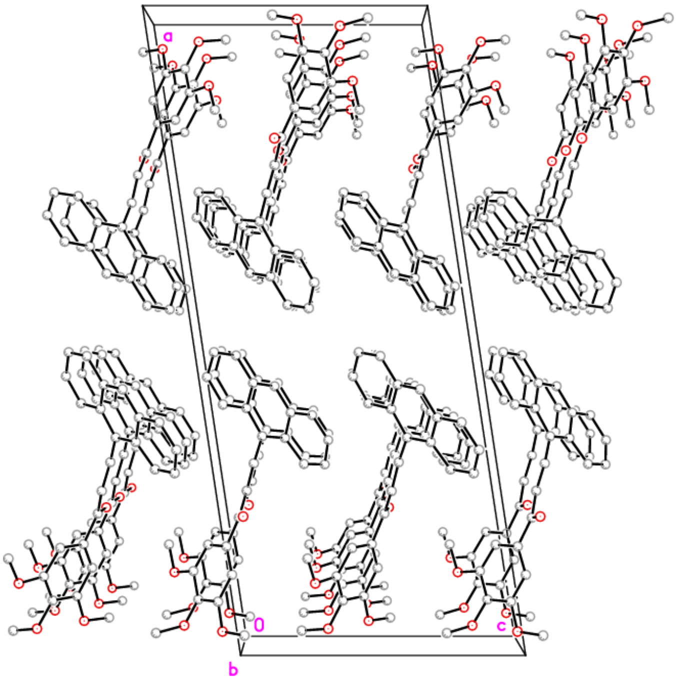

Figure S3 : Crystal packing arrangement seen in the X-ray structure of (E)-3-(anthracen-9-yl)-1-(3,4,5-trimethoxyphenyl)prop-2-en-1-one (**21k**) viewed normal to the b-axis. Hydrogen atoms omitted for clarity.

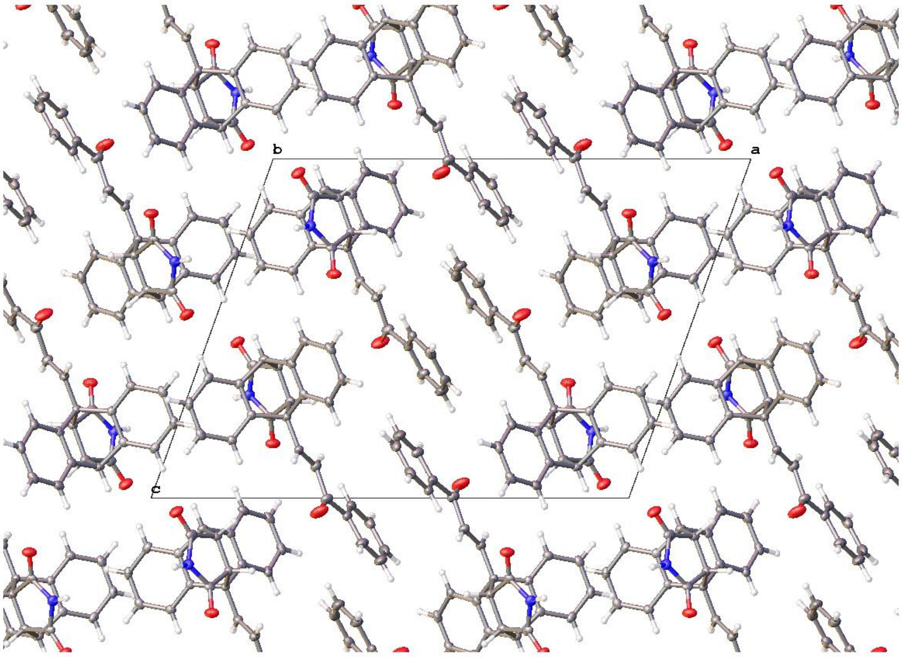

Figure S4: Crystal packing arrangement in the X-ray structure of (E)-9-(3-oxo-3-phenylprop-1-en-1-yl)-9,10-dihydro-9,10-[3,4]epipyrroloanthracene-12,14-dione (**23f**) viewed normal to the b-axis.

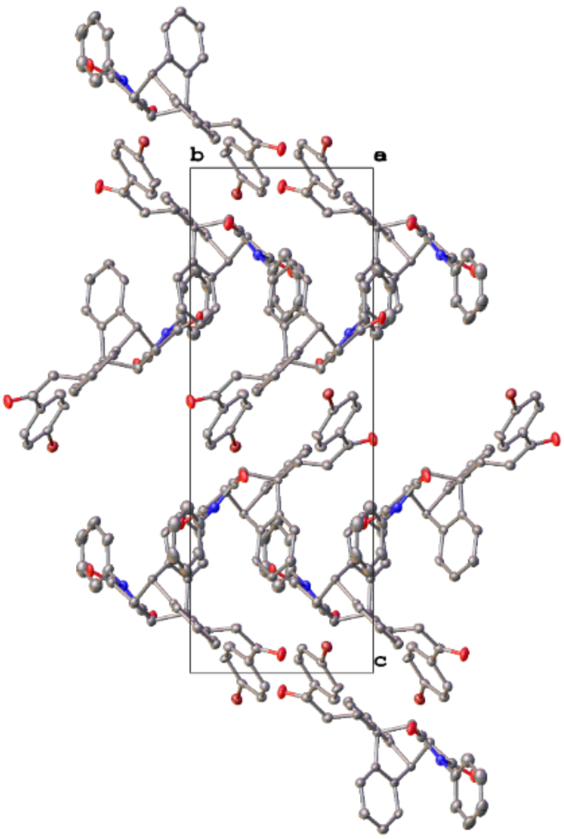

Figure S5: Packing arrangement of (*E*)-9-(3-(4-bromophenyl)-3-oxoprop-1-en-1-yl)-13-phenyl-9,10-dihydro-9,10-[3,4]epipyrroloanthracene-12,14-dione (**24a**) viewed normal to the a-axis. Hydrogen atoms omitted for clarity.

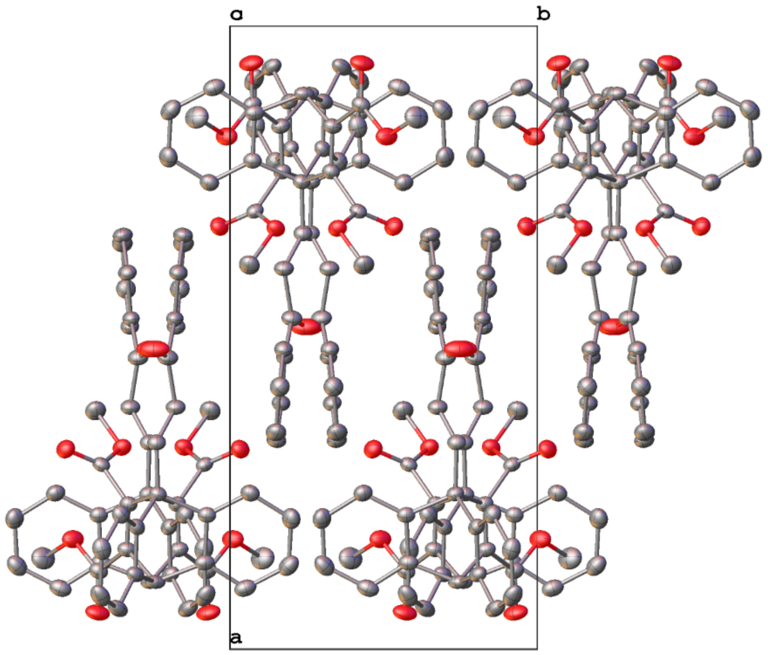

Figure S6: Packing arrangement of dimethyl (*E*)-9-(3-oxo-3-phenylprop-1-en-1-yl)-9,10-dihydro-9,10-ethenoanthracene-11,12-dicarboxylate (**27**) viewede normal to the c-axis. Hydrogen atoms omitted forr clarity.

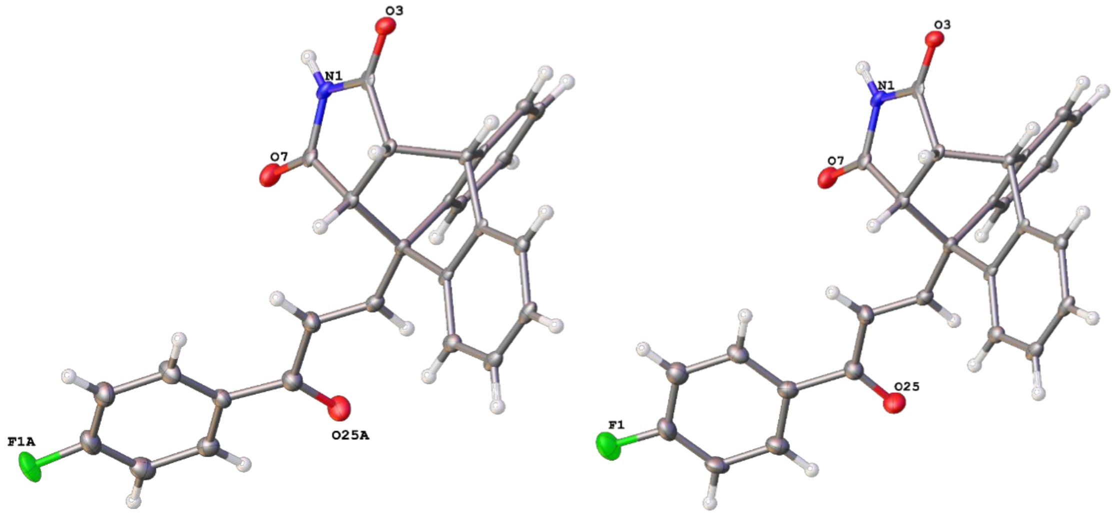

Figure S7: Major and minor disordered moieties of **23h** with heteroatoms labelled for clarity. Atomic displacement shown at 50% probability

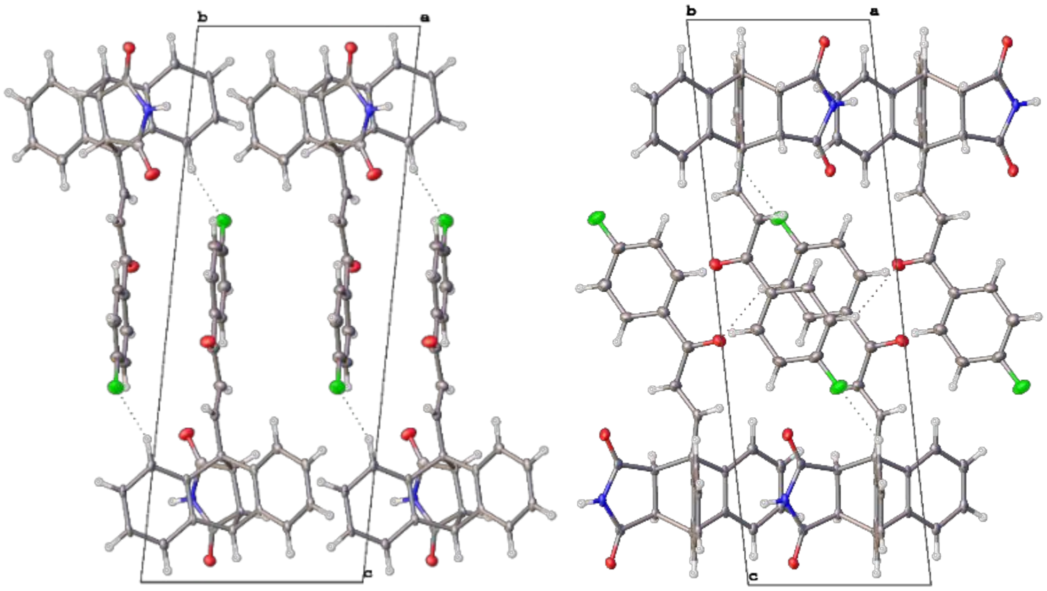

A B

Figure S8: Packing diagrams of (**23h**) viewed normal to the (**A**) a-axis and (**B**) b-axis.

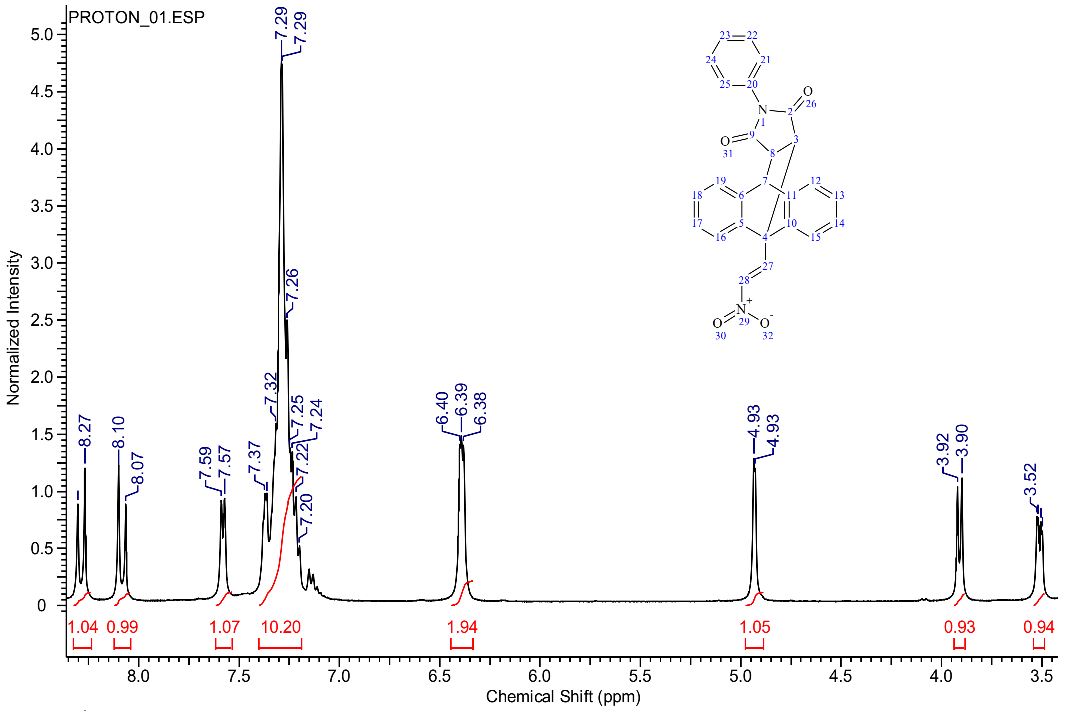

Figure S9: ^{1}H NMR spectrum of (*E*)-9-(2-nitrovinyl)-13-phenyl-9,10-dihydro-9,10-[3,4]epipyrroloanthracene-12,14-dione (**20a**)

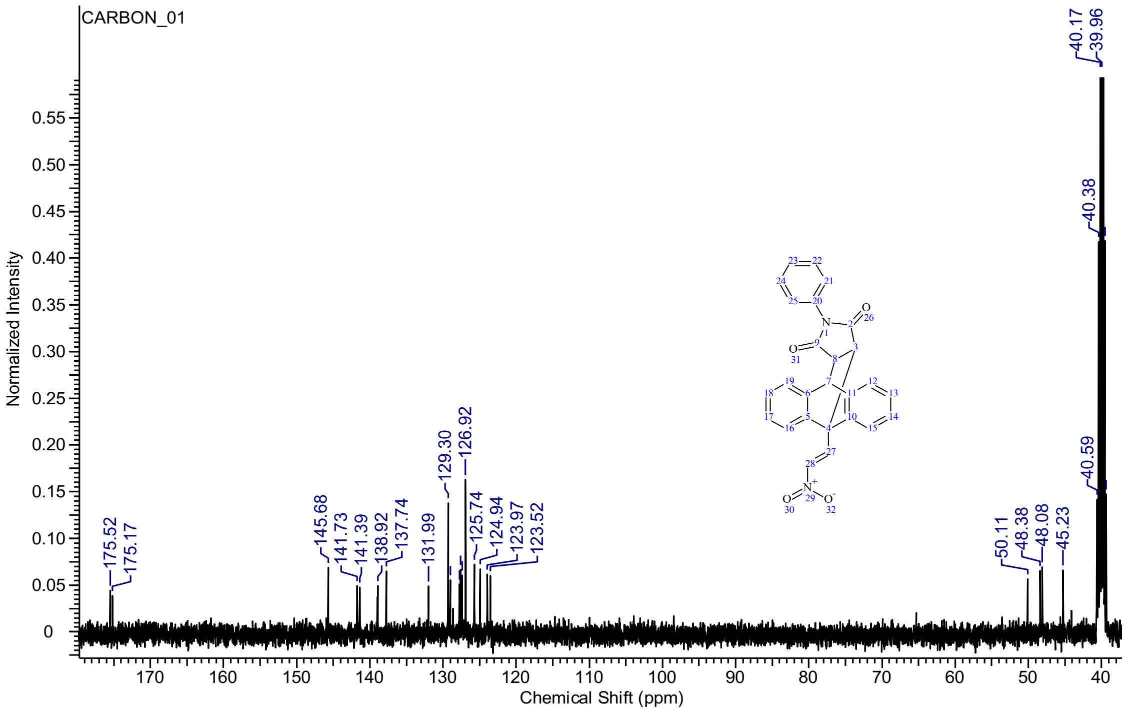

Figure S10: ^{13}C NMR spectrum of (*E*)-9-(2-nitrovinyl)-13-phenyl-9,10-dihydro-9,10-[3,4]epipyrroloanthracene-12,14-dione (**20a**)

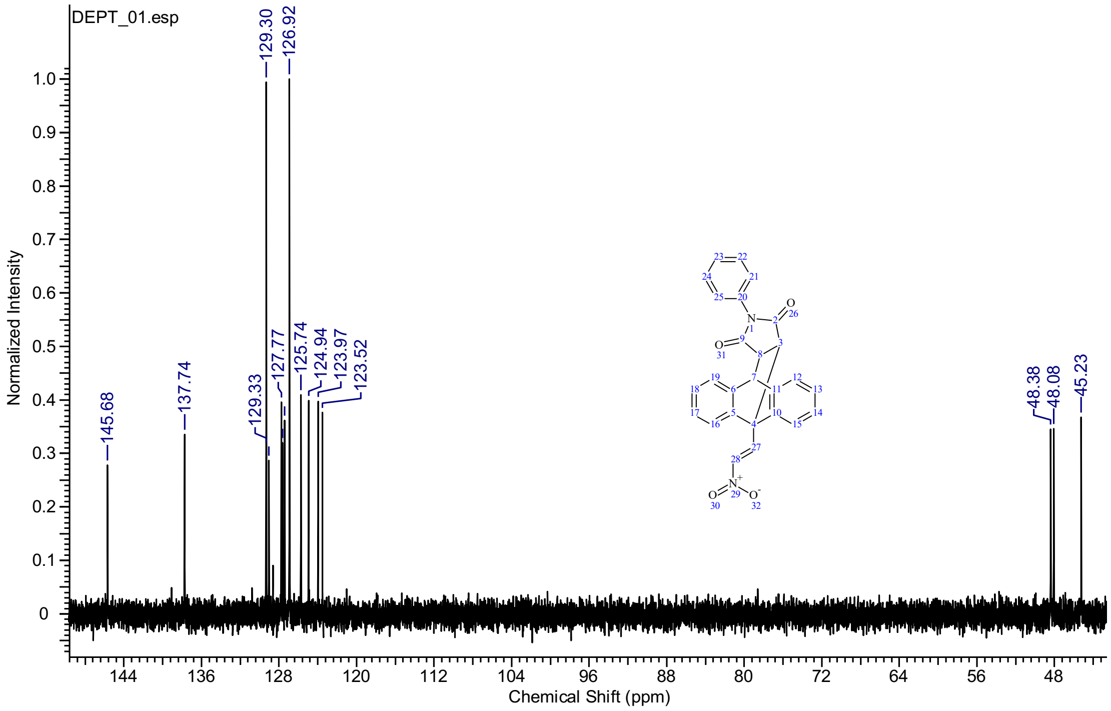

Figure S11: DEPT 90 spectrum of (*E*)-9-(2-nitrovinyl)-13-phenyl-9,10-dihydro-9,10-[3,4]epipyrroloanthracene-12,14-dione (**20a**)

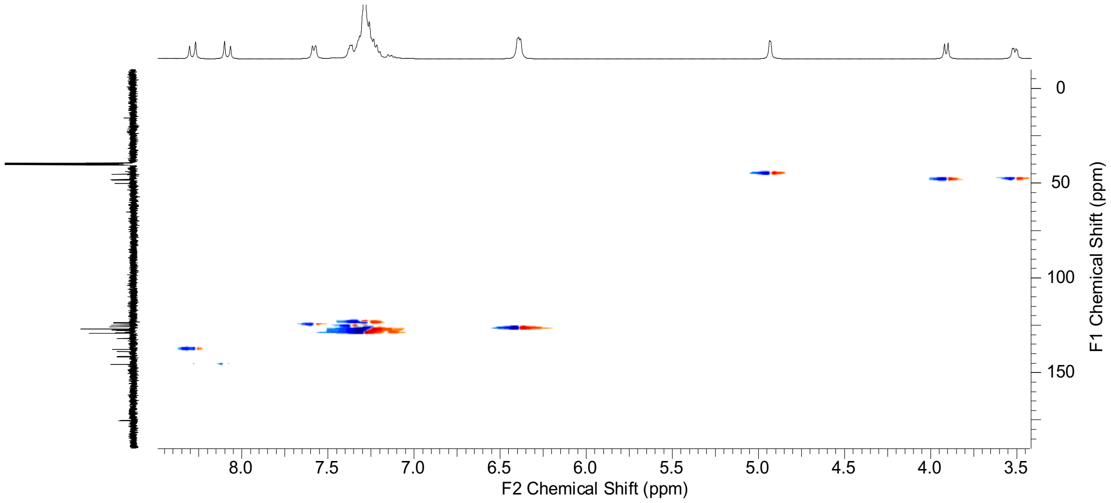

Figure S12: HSQC spectrum of (*E*)-9-(2-nitrovinyl)-13-phenyl-9,10-dihydro-9,10-[3,4]epipyrroloanthracene-12,14-dione (**20a**)

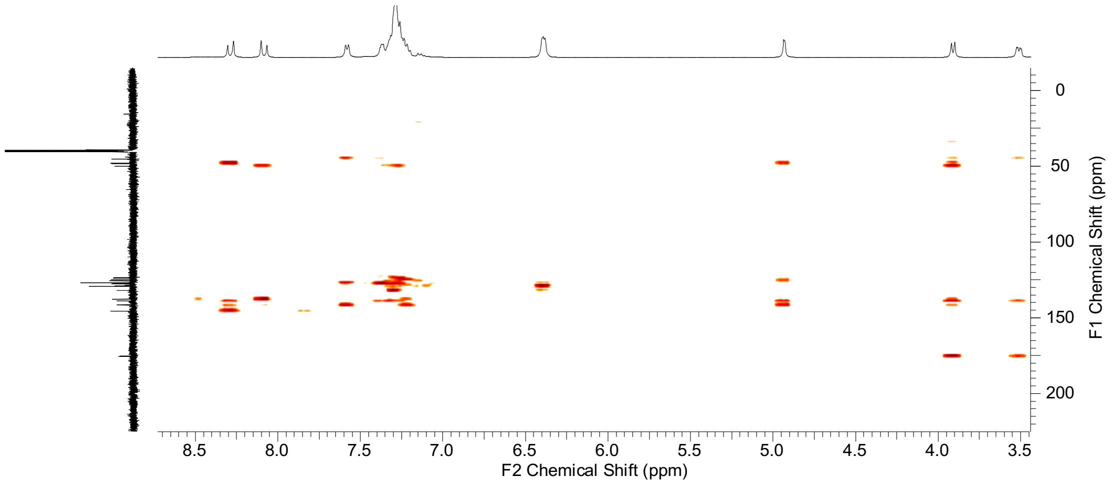

Figure S13: HMBC spectrum of (*E*)-9-(2-nitrovinyl)-13-phenyl-9,10-dihydro-9,10-[3,4]epipyrroloanthracene-12,14-dione (**20a**)

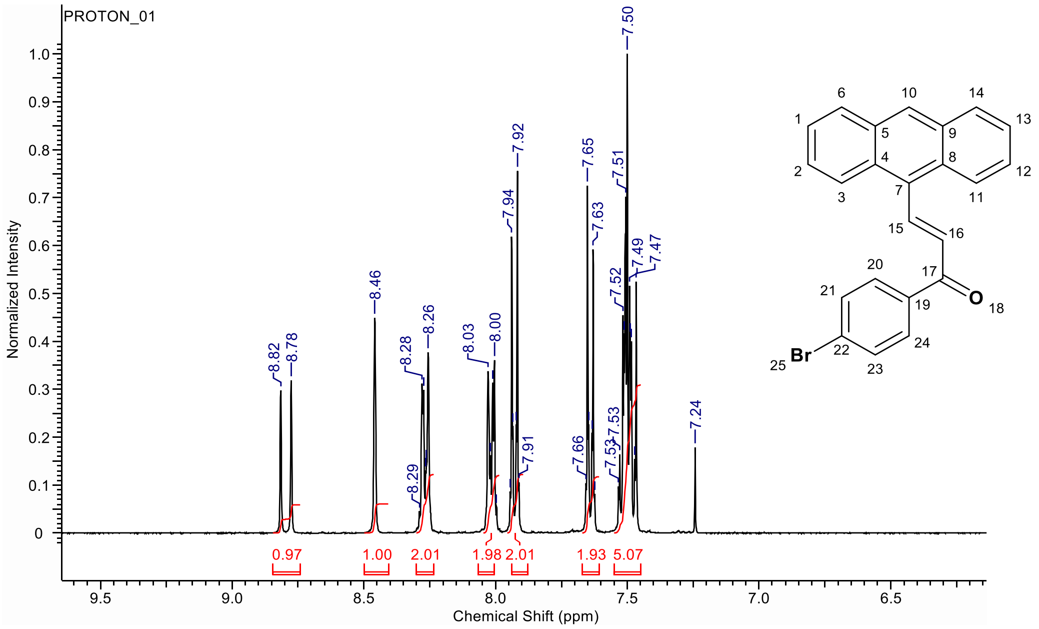

Figure S14: ^{1}H NMR spectrum of (*E*)-3-(anthracen-9-yl)-1-(4-bromophenyl)prop-2-en-1-one (**21a**)

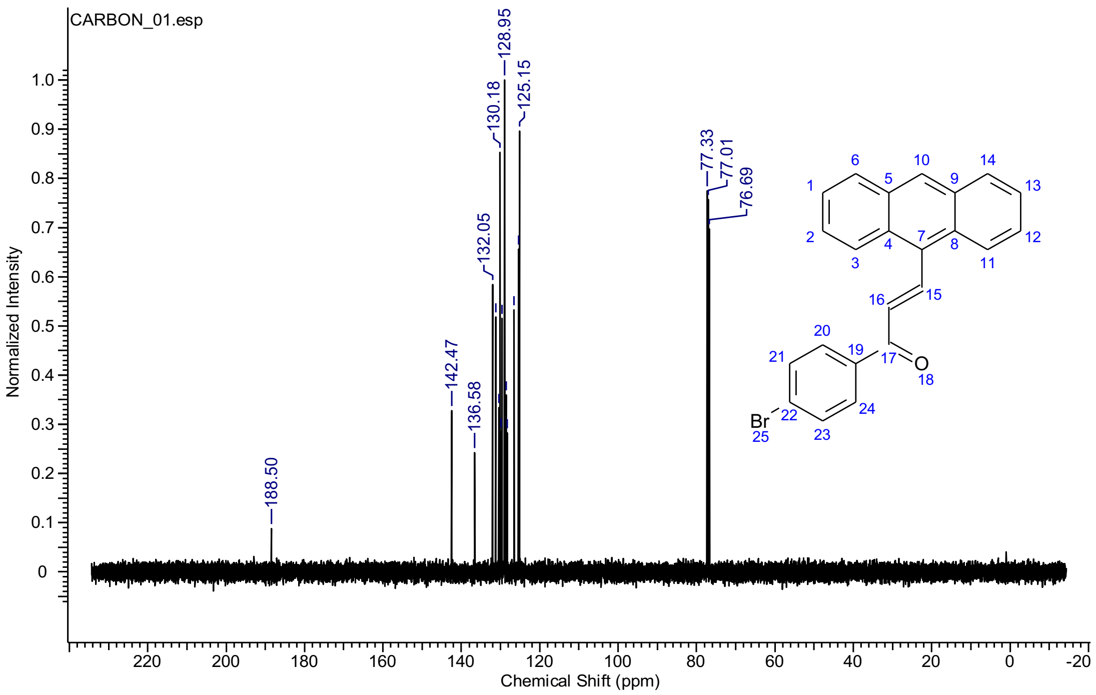

Figure S15: ^{13}C NMR spectrum of (*E*)-3-(anthracen-9-yl)-1-(4-bromophenyl)prop-2-en-1-one (**21a**)

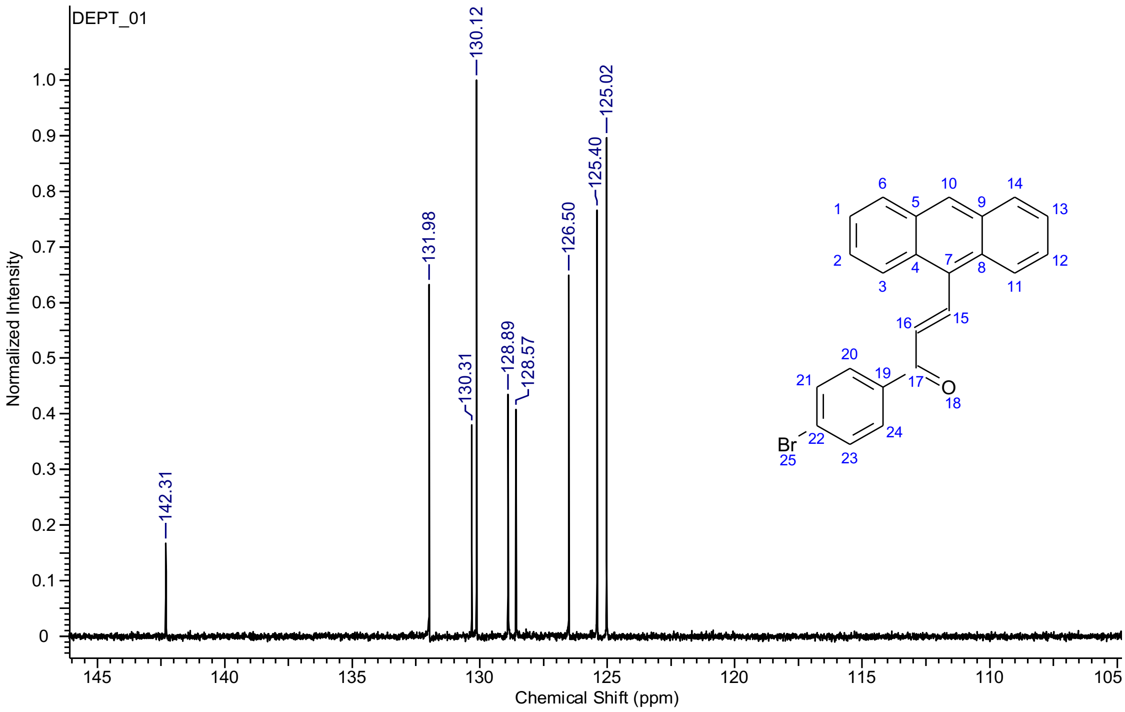

Figure S16: DEPT 90 spectrum of (*E*)-3-(anthracen-9-yl)-1-(4-bromophenyl)prop-2-en-1-one (**21a**)

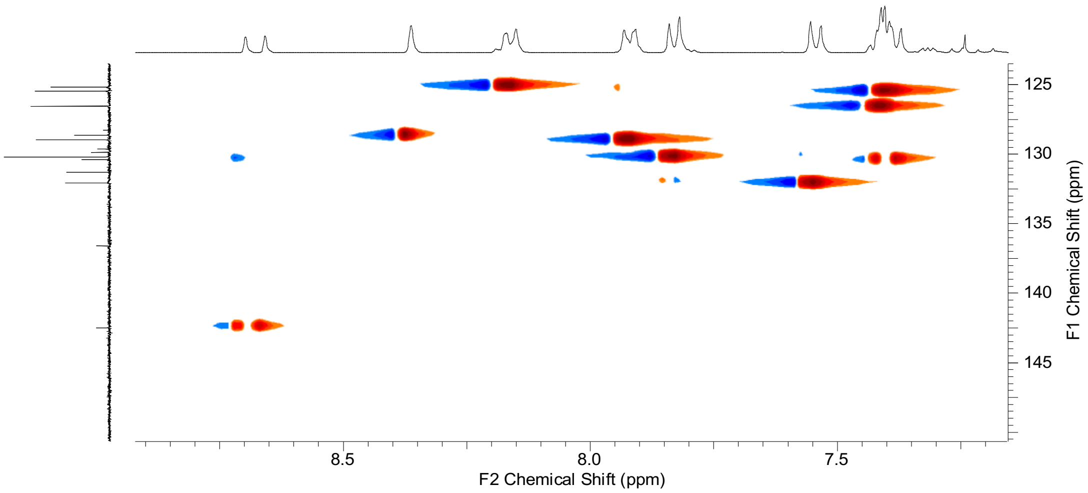

Figure S17: HSQC spectrum of (*E*)-3-(anthracen-9-yl)-1-(4-bromophenyl)prop-2-en-1-one (**21a**)

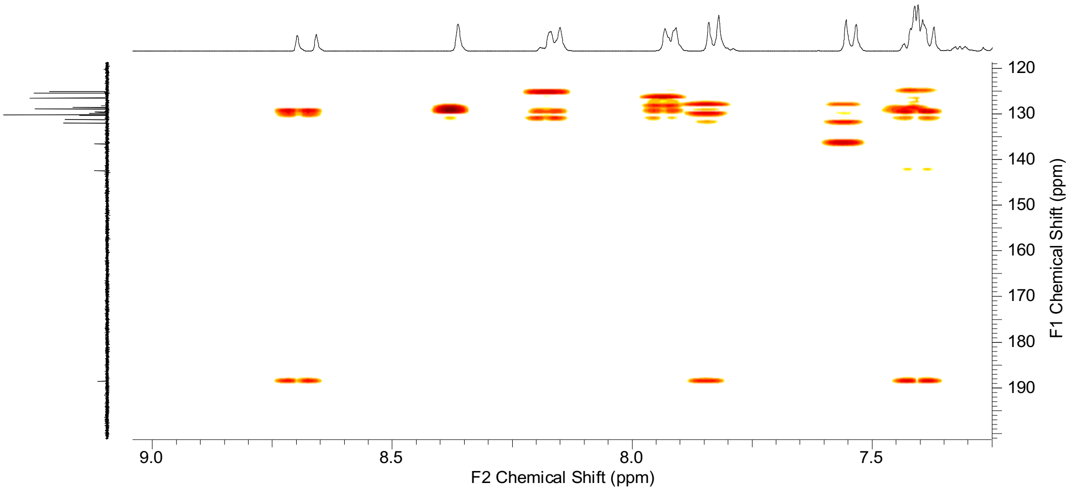

Figure S18: HMBC spectrum of (*E*)-3-(anthracen-9-yl)-1-(4-bromophenyl)prop-2-en-1-one (**21a**)

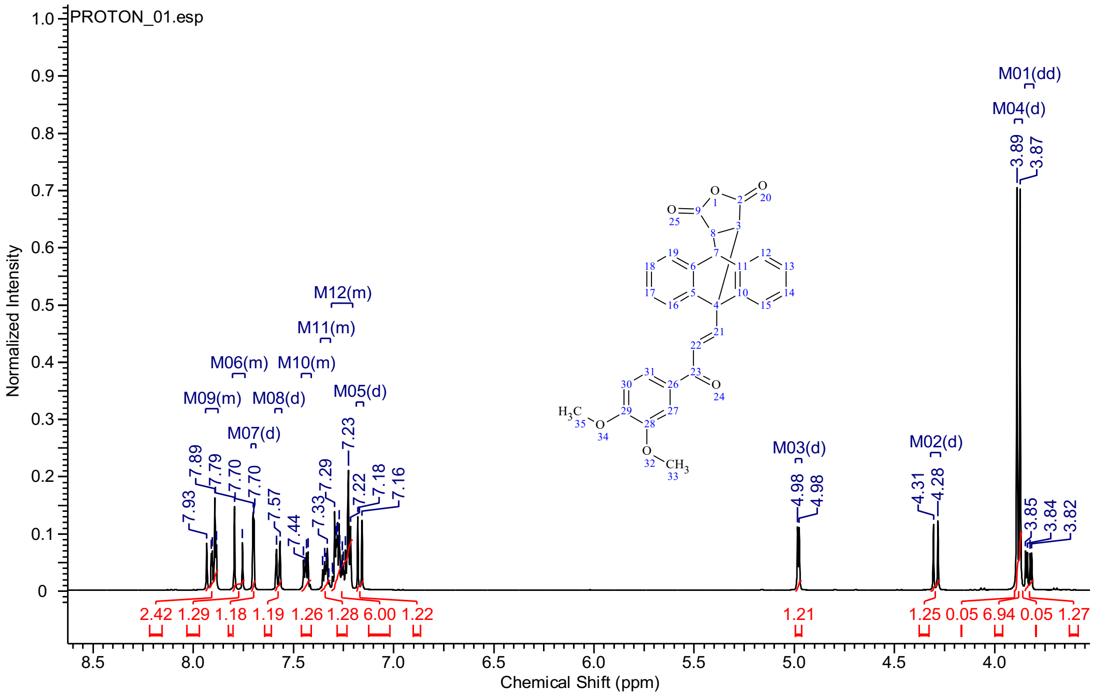

Figure S19: ^{1}H NMR spectrum of (*E*)-3-(3,4-dimethoxyphenyl)-3-oxoprop-1-en-1-yl)-9,10-dihydro-9,10 [3,4]furanoanthracene-12,14-dione (**22e**)

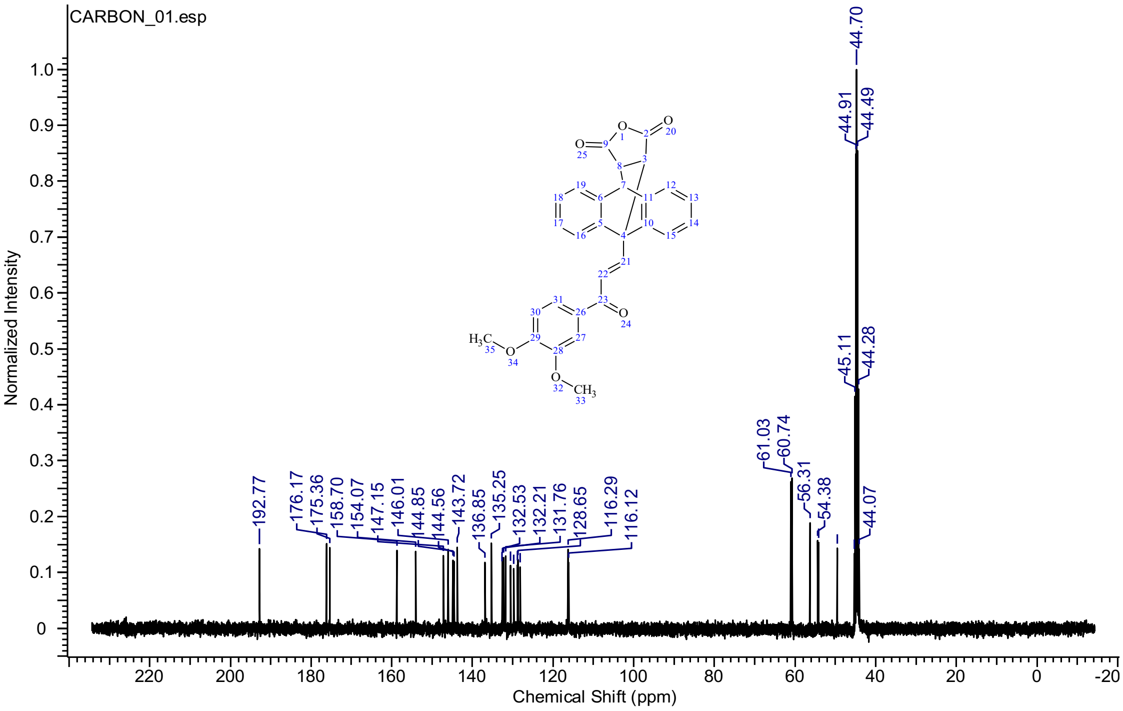

Figure S20: ^{13}C NMR spectrum of (*E*)-3-(3,4-dimethoxyphenyl)-3-oxoprop-1-en-1-yl)-9,10-dihydro-9,10 [3,4]furanoanthracene-12,14-dione (**22e**)

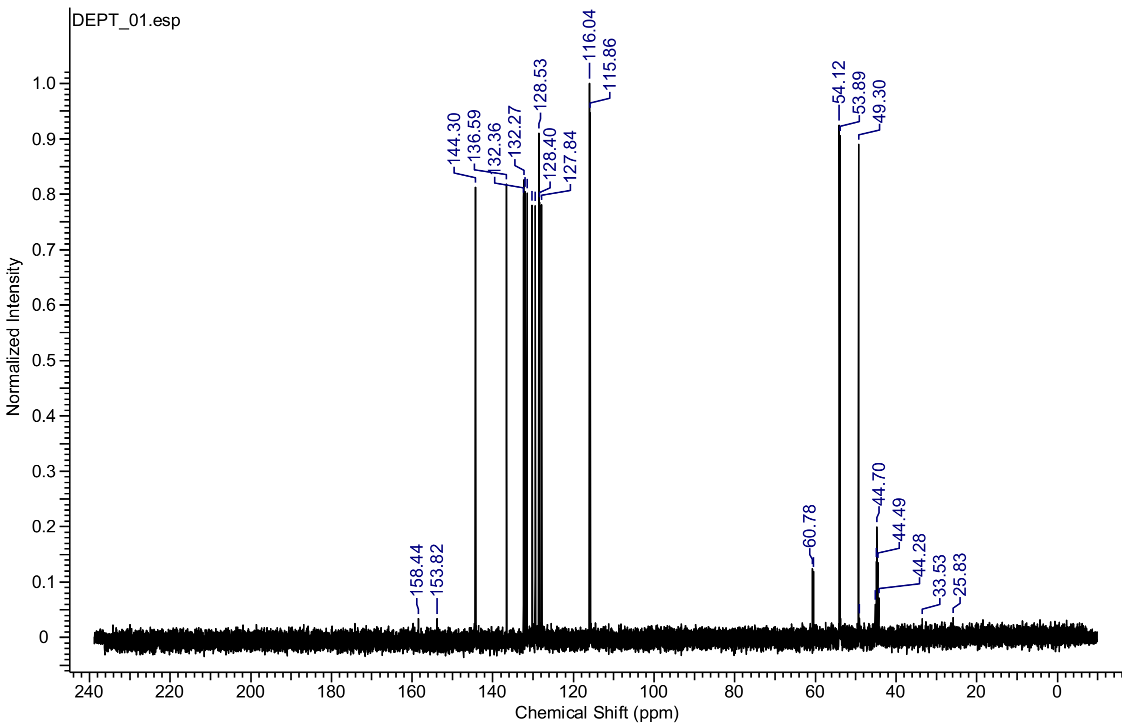

Figure S21: DEPT 90 of (*E*)-3-(3,4-dimethoxyphenyl)-3-oxoprop-1-en-1-yl)-9,10-dihydro-9,10 [3,4]furanoanthracene-12,14-dione (**22e**)

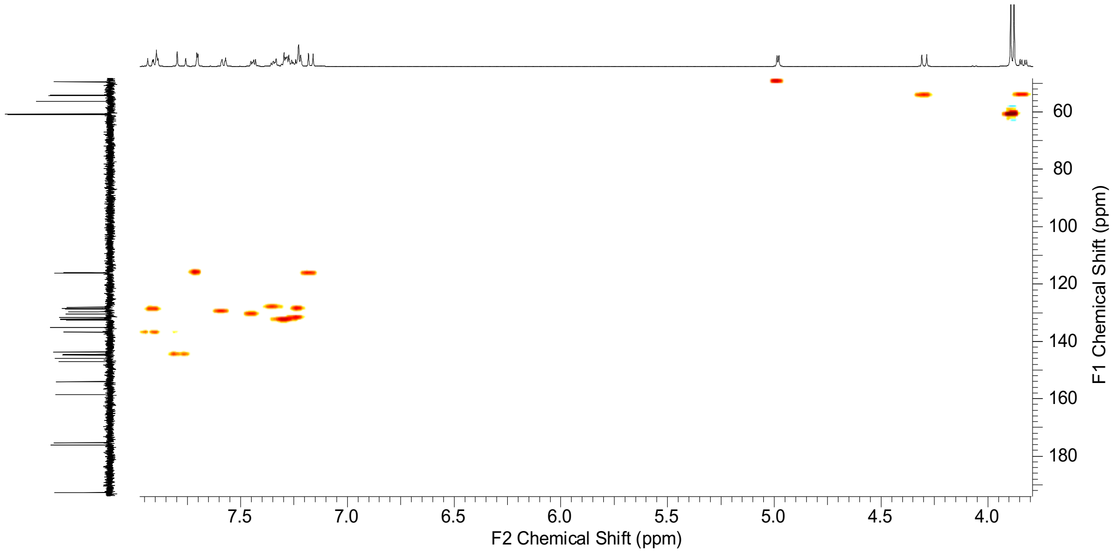

Figure S22: HSQC spectrum of (*E*)-3-(3,4-dimethoxyphenyl)-3-oxoprop-1-en-1-yl)-9,10-dihydro-9,10 [3,4]furanoanthracene-12,14-dione (**22e**)

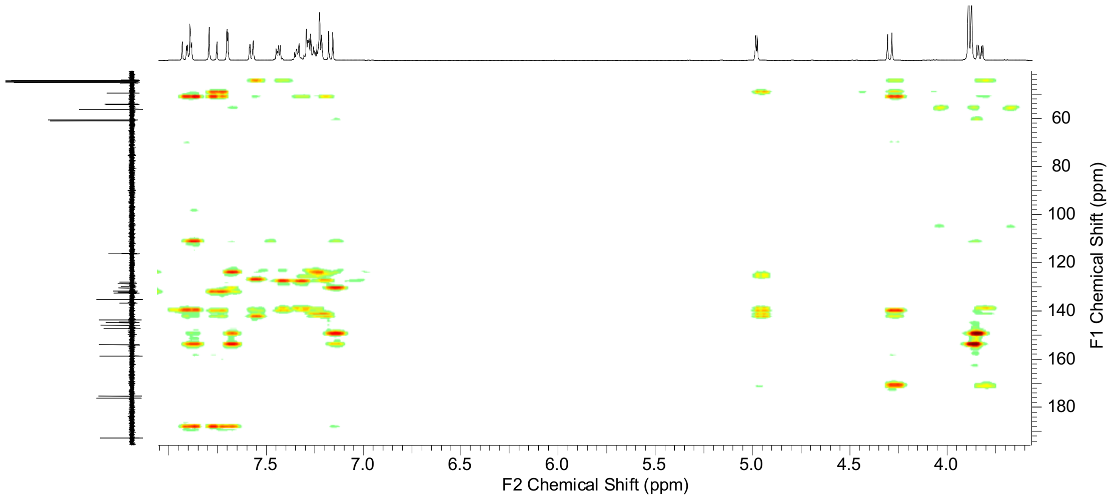

Figure S23: HMBC spectrum of (*E*)-3-(3,4-dimethoxyphenyl)-3-oxoprop-1-en-1-yl)-9,10-dihydro-9,10 [3,4]furanoanthracene-12,14-dione (**22e**)

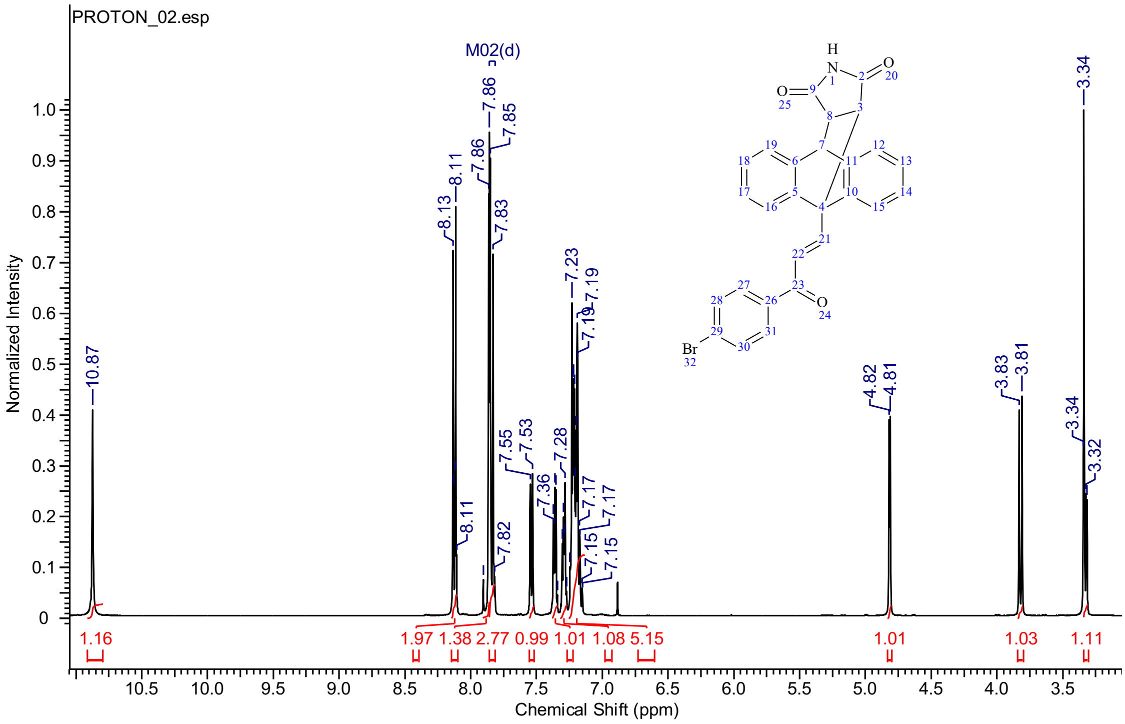

Figure S24: ^{1}H-NMR spectrum of (*E*)-9-(3-(4-bromophenyl)-3-oxoprop-1-en-1-yl)-9,10-dihydro-9,10- [3,4]epipyrroloanthracene-12,14-dione (**23a**)

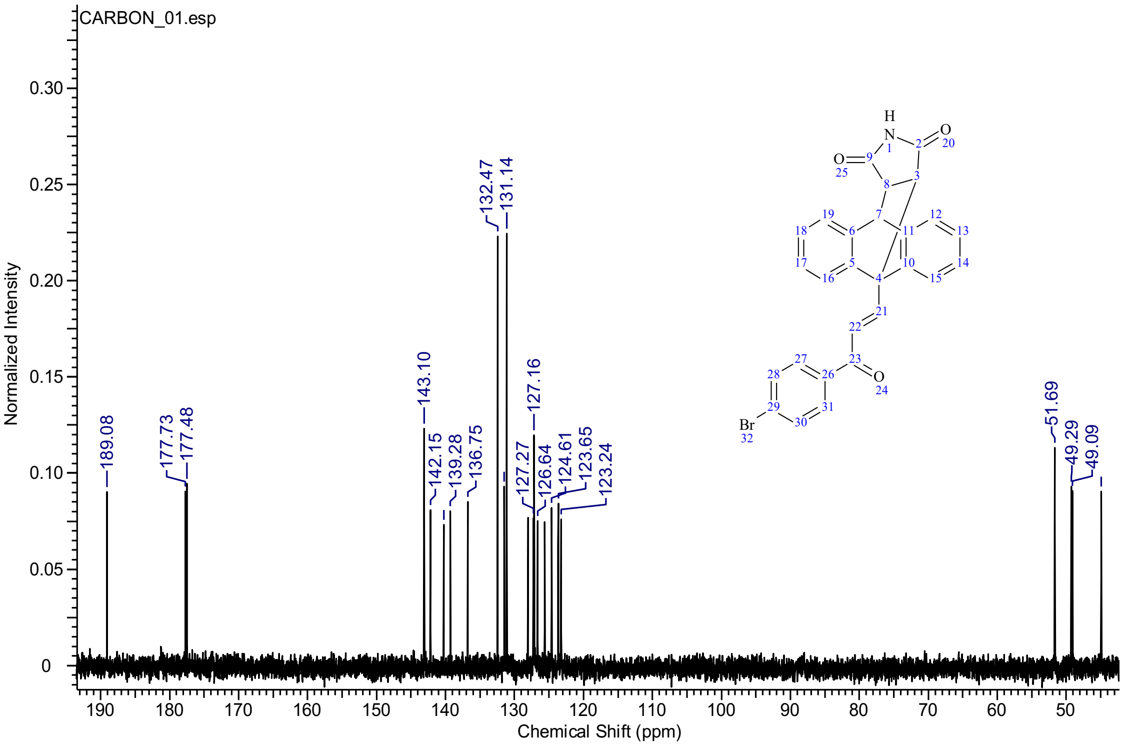

Figure S25: ^{13}C NMR spectrum of (*E*)-9-(3-(4-bromophenyl)-3-oxoprop-1-en-1-yl)-9,10-dihydro-9,10- [3,4]epipyrroloanthracene-12,14-dione (**23a**)

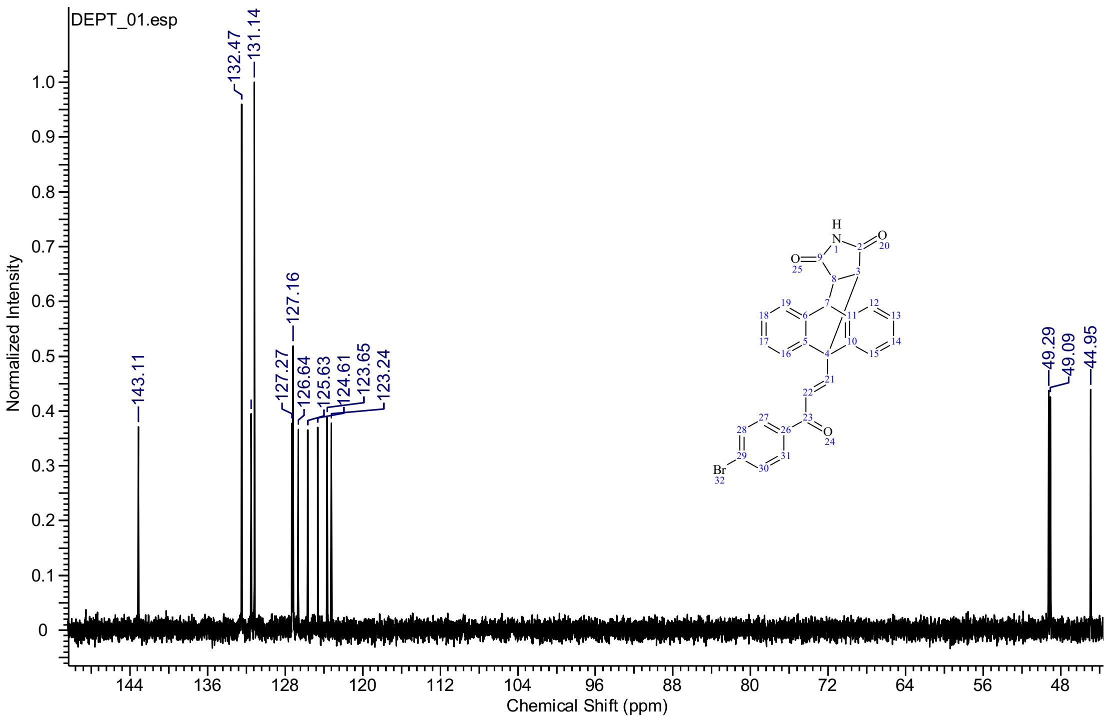

Figure S26: DEPT 90 of (E)-9-(3-(4-bromophenyl)-3-oxoprop-1-en-1-yl)-9,10-dihydro-9,10-[3,4]epipyrroloanthracene-12,14-dione (**23a**)

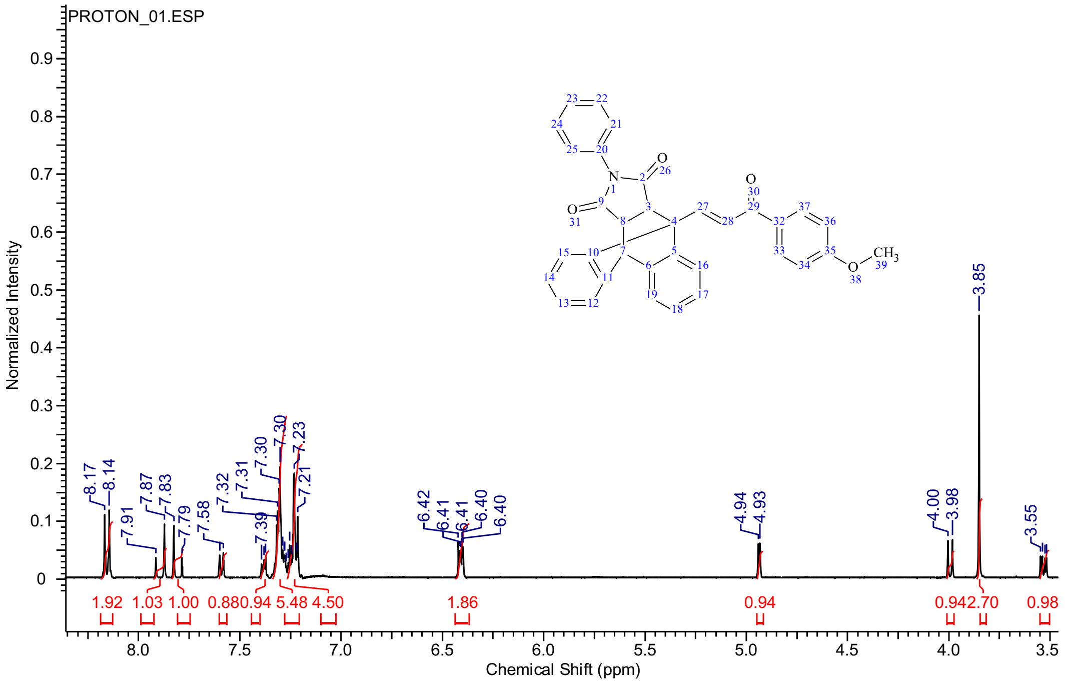

Figure S27: ^{1}H NMR spectrum of (*E*)-9-(3-(4-methoxyphenyl)-3-oxoprop-1-en-1-yl)-13-phenyl-9,10-dihydro-9,10-[3,4]epipyrroloanthracene-12,14-dione (**24i**)

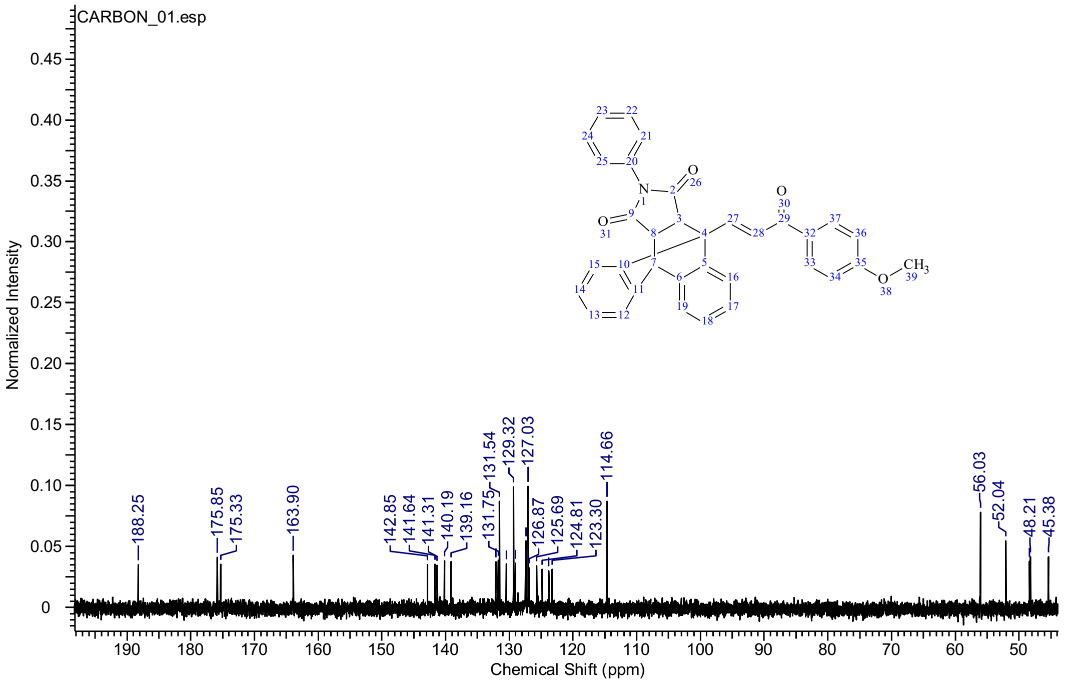

Figure S28: ^{13}C NMR spectrum of (*E*)-9-(3-(4-methoxyphenyl)-3-oxoprop-1-en-1-yl)-13-phenyl-9,10-dihydro-9,10-[3,4]epipyrroloanthracene-12,14-dione (**24i**)

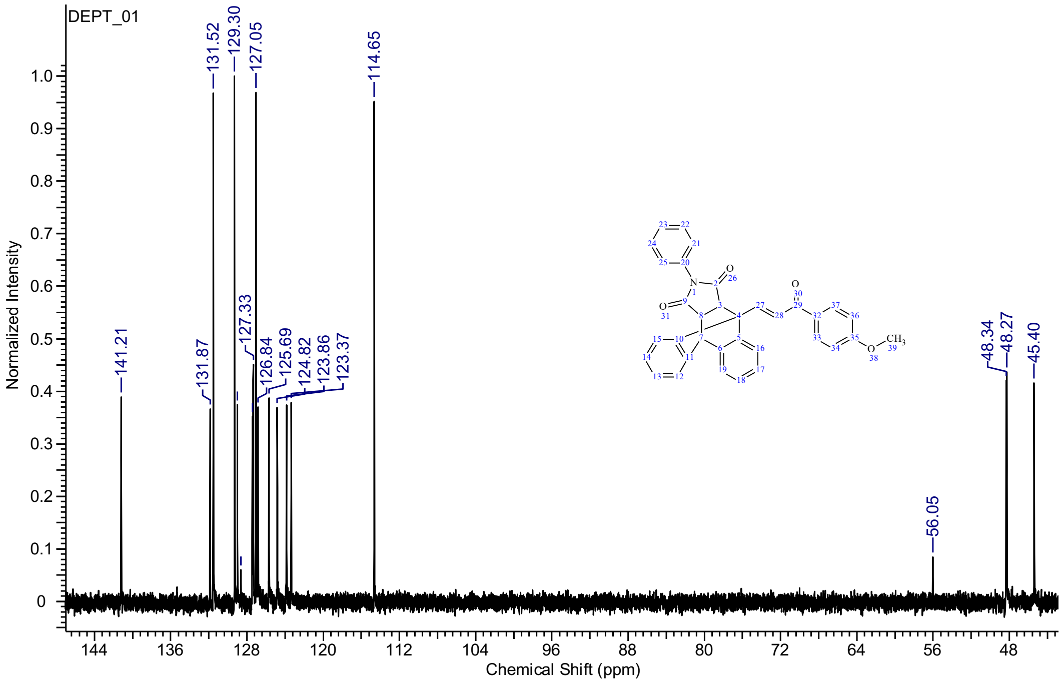

Figure S29: DEPT 90 spectrum of (*E*)-9-(3-(4-methoxyphenyl)-3-oxoprop-1-en-1-yl)-13-phenyl-9,10-dihydro-9,10-[3,4]epipyrroloanthracene-12,14-dione (**24i**)

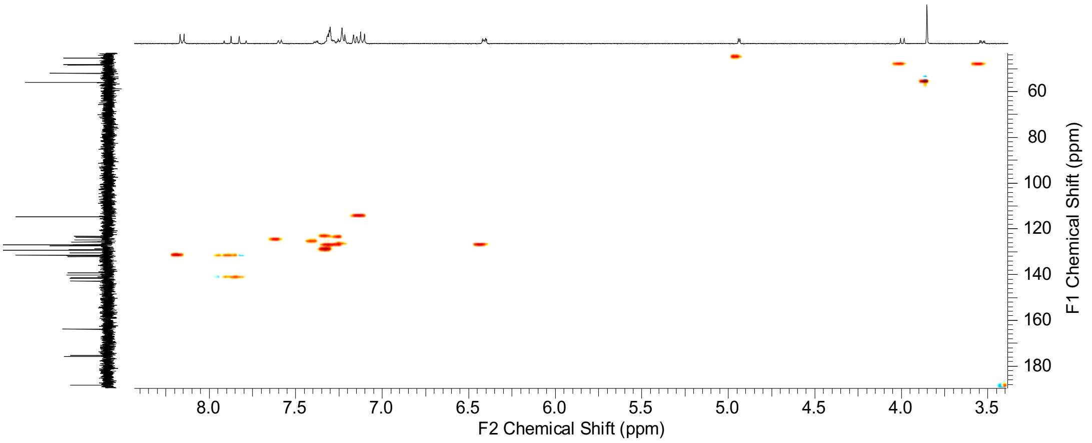

Figure S30: HSQC spectrum of of (*E*)-9-(3-(4-methoxyphenyl)-3-oxoprop-1-en-1-yl)-13-phenyl-9,10-dihydro-9,10-[3,4]epipyrroloanthracene-12,14-dione (**24i**)

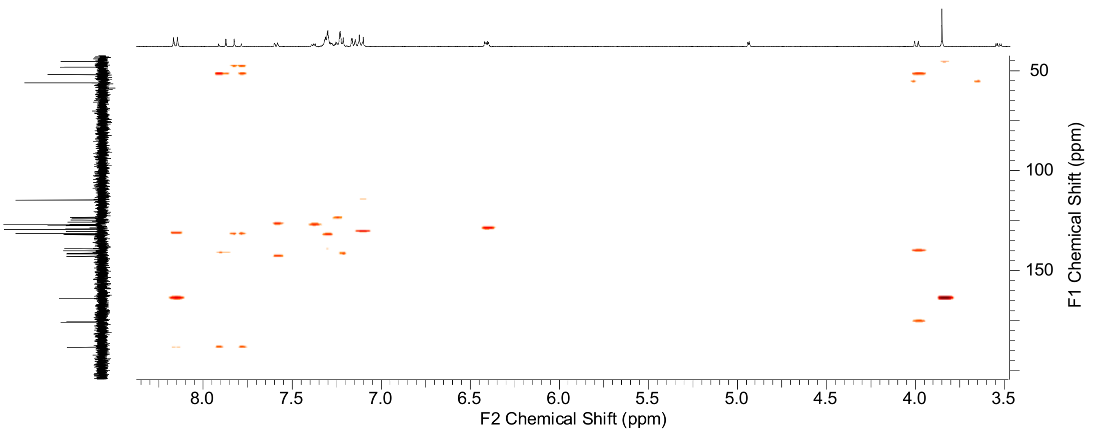

Figure S31: HMBC spectrum of of (*E*)-9-(3-(4-methoxyphenyl)-3-oxoprop-1-en-1-yl)-13-phenyl-9,10-dihydro-9,10-[3,4]epipyrroloanthracene-12,14-dione (**24i**)

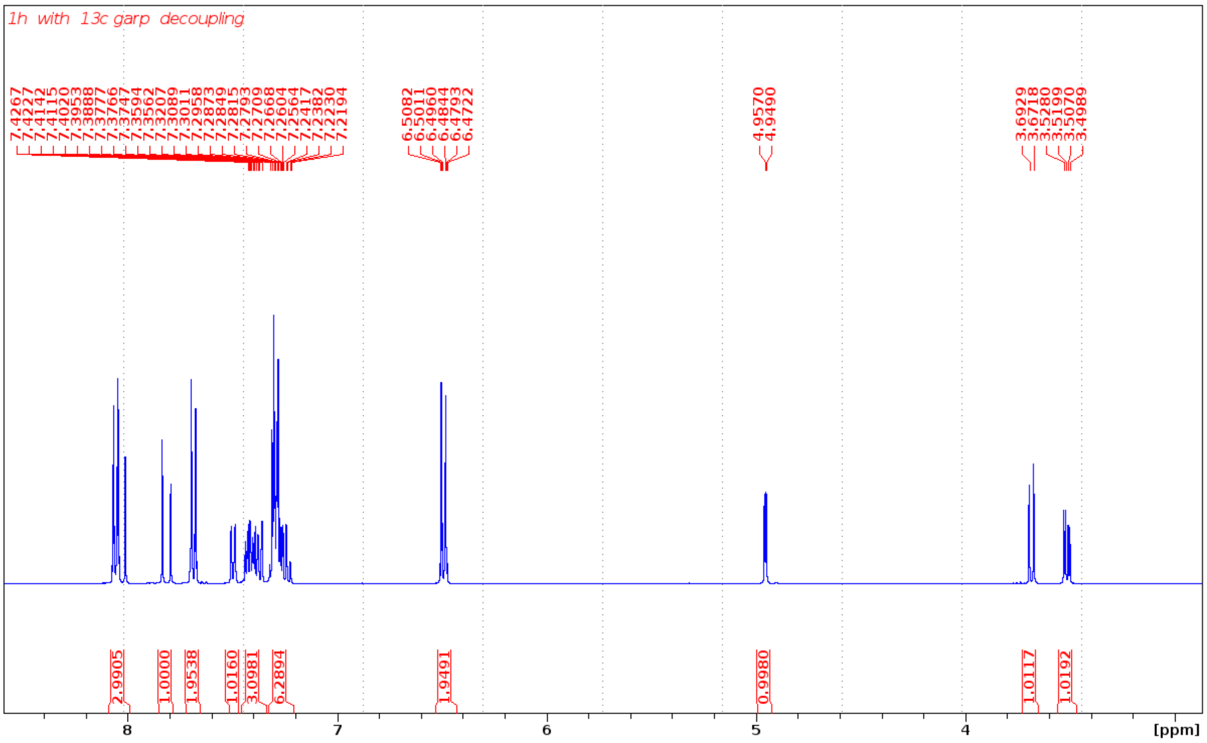

Figure S32: ^{1}H NMR spectrum (*E*)-9-(3-(4-Bromophenyl)-3-oxoprop-1-en-1-yl) -13-(4-chlorophenyl)-9,10-dihydro- 9,10-[3,4]epipyrroloanthracene-12,14-dione (**25a**)

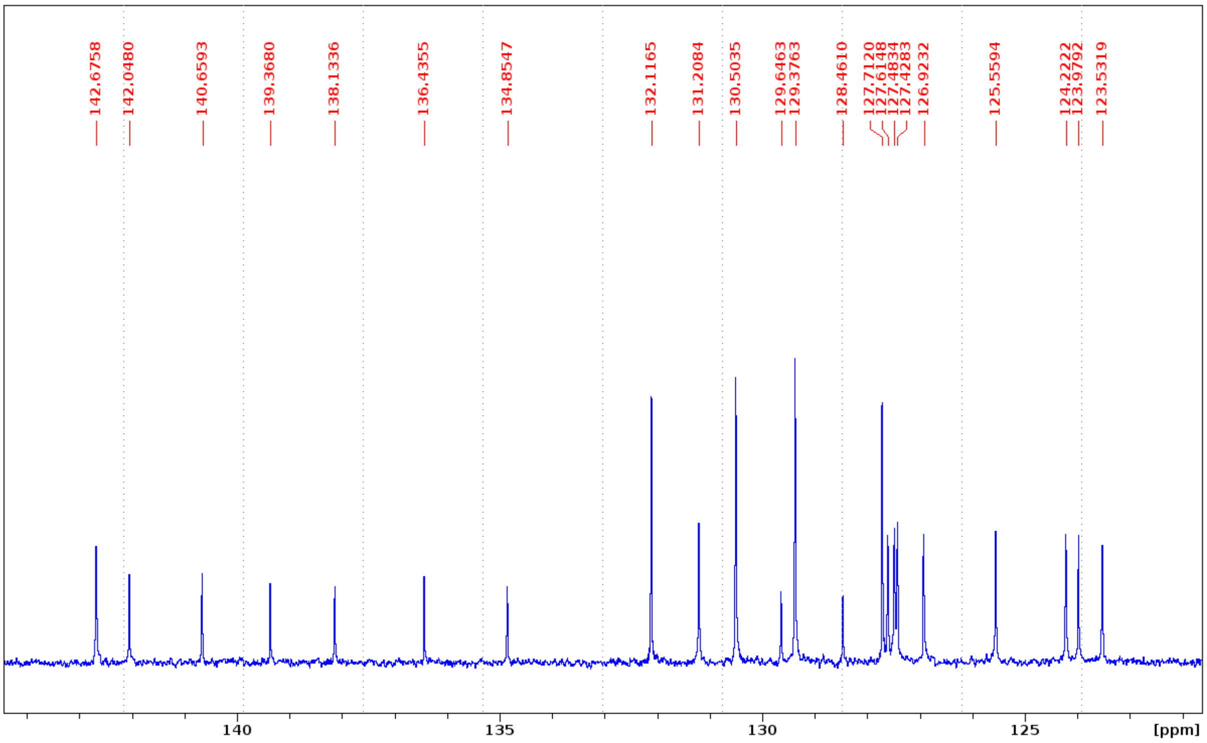

Figure S33: ^{13}C NMR spectrum (*E*)-9-(3-(4-Bromophenyl)-3-oxoprop-1-en-1-yl)-13-(4-chlorophenyl)-9,10-dihydro- 9,10-[3,4]epipyrroloanthracene-12,14-dione (**25a**)

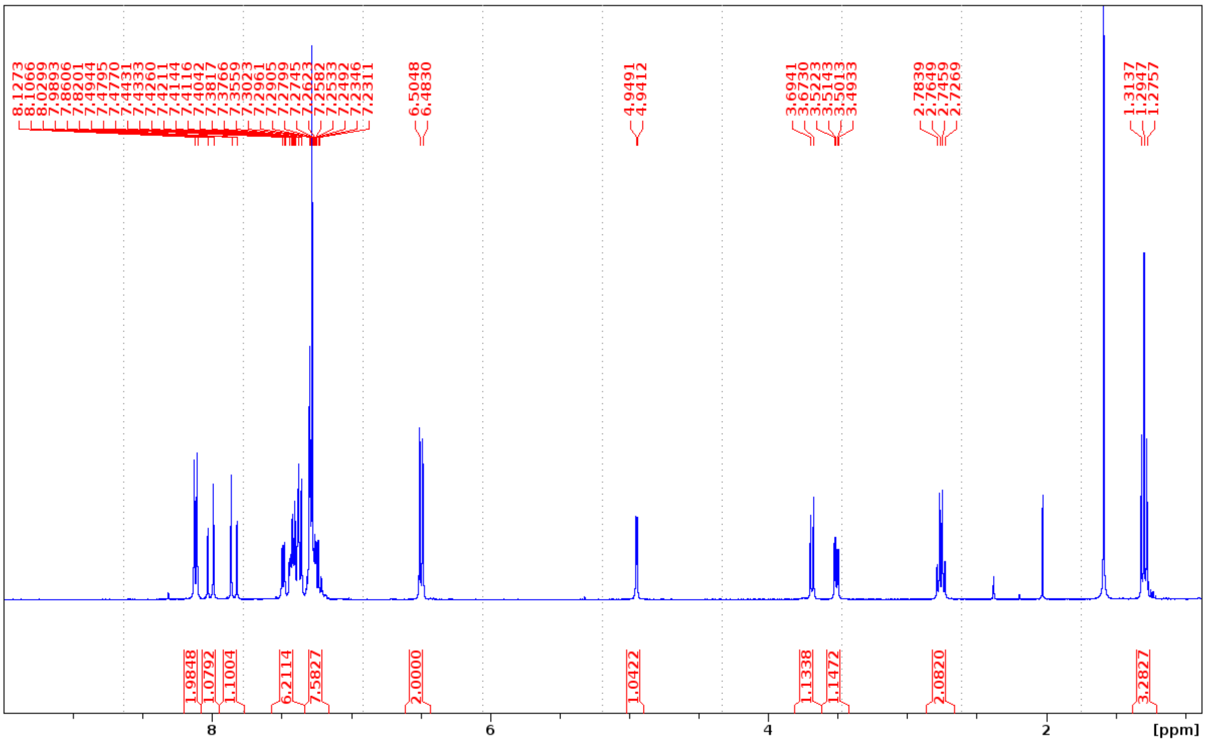

Figure S34: ^{1}H NMR spectrum *E*)-13-(4-Chlorophenyl)-9-(3-(4-ethylphenyl)-3-oxoprop-1-en-1-yl)-9,10-dihydro- 9,10-[3,4]epipyrroloanthracene-12,14-dione (**25c**)

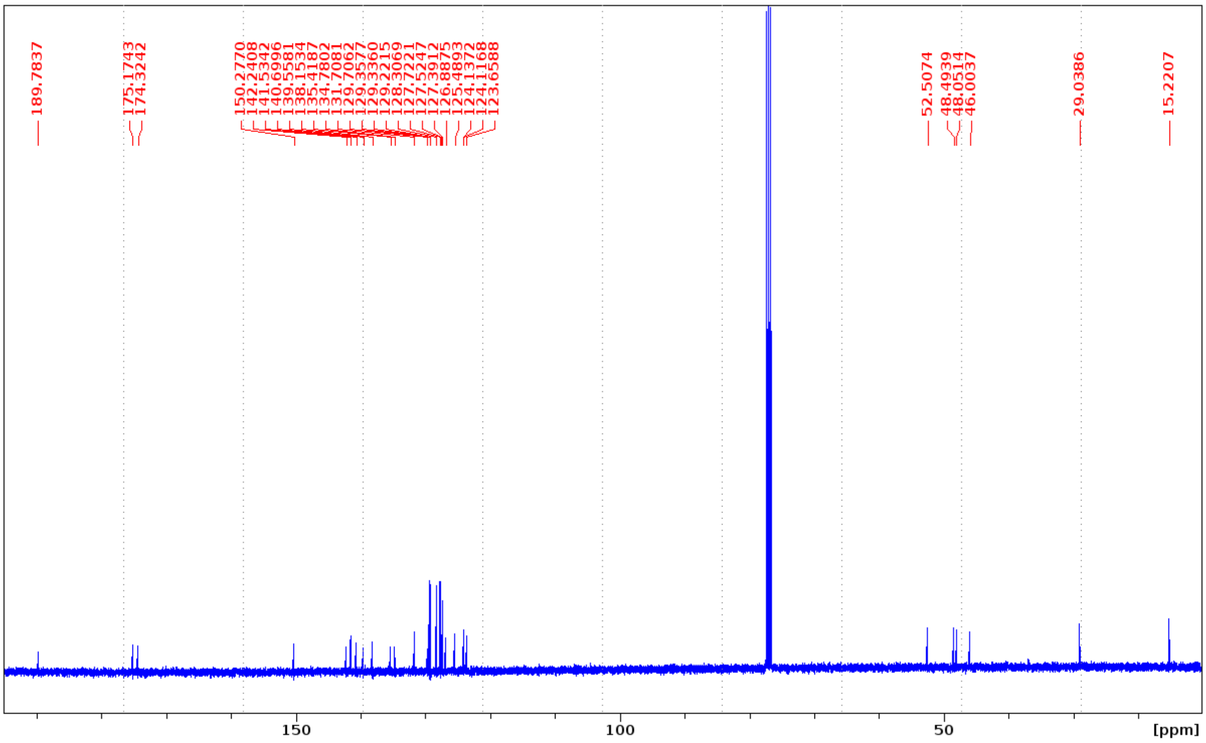

Figure S35: ^{13}C NMR spectrum *E*)-13-(4-Chlorophenyl)-9-(3-(4-ethylphenyl)-3-oxoprop-1-en-1-yl)-9,10-dihydro- 9,10-[3,4]epipyrroloanthracene-12,14-dione (**25c**)

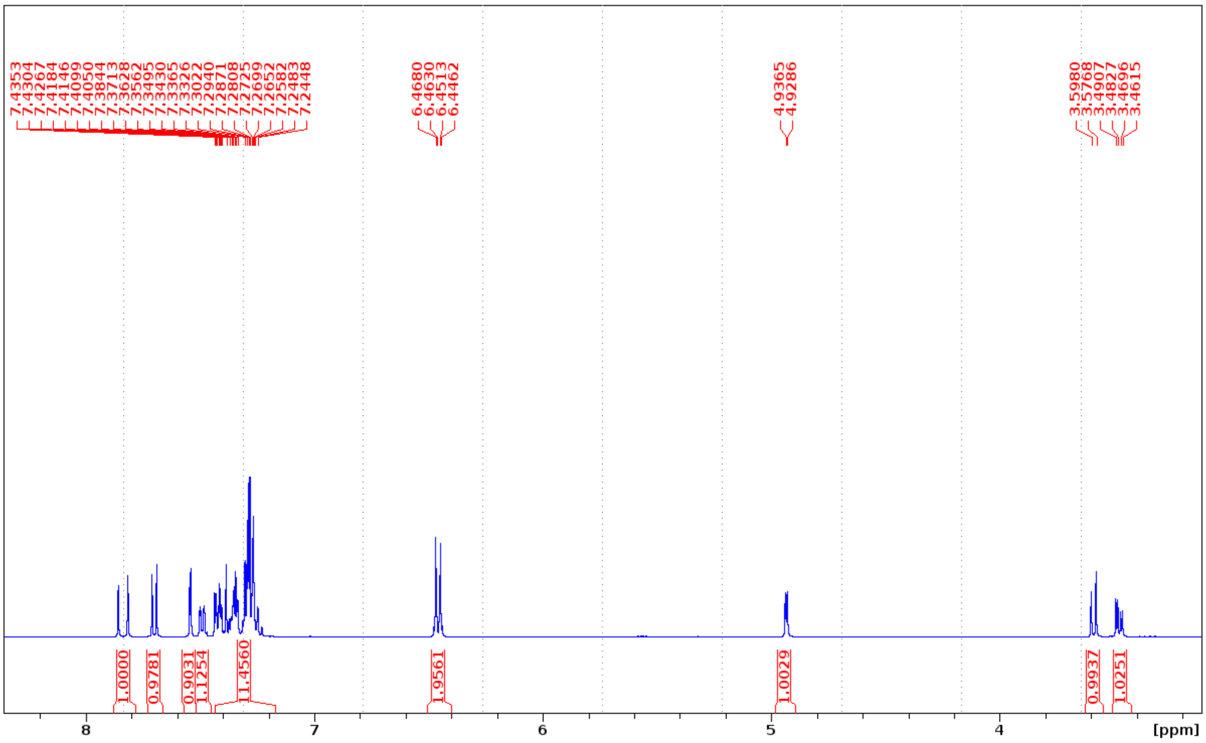

Figure S36: ^{1}H NMR spectrum (*E*)-13-(4-Chlorophenyl)-9-(3-(2,4-dichlorophenyl)-3-oxoprop-1-en-1-yl)-9,10- dihydro-9,10-[3,4]epipyrroloanthracene-12,14-dione (**25d**)

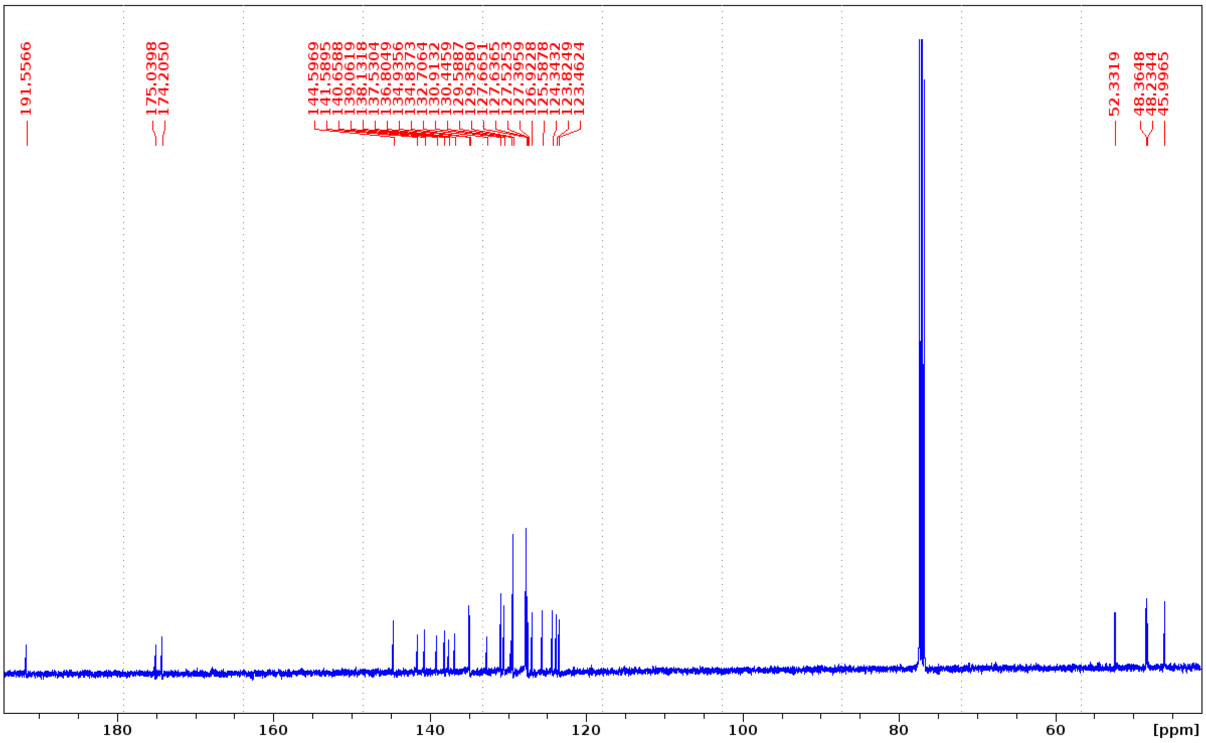

Figure S37: ^{13}C NMR spectrum (*E*)-13-(4-Chlorophenyl)-9-(3-(2,4-dichlorophenyl)-3-oxoprop-1-en-1-yl)-9,10- dihydro-9,10-[3,4]epipyrroloanthracene-12,14-dione (**25d**)

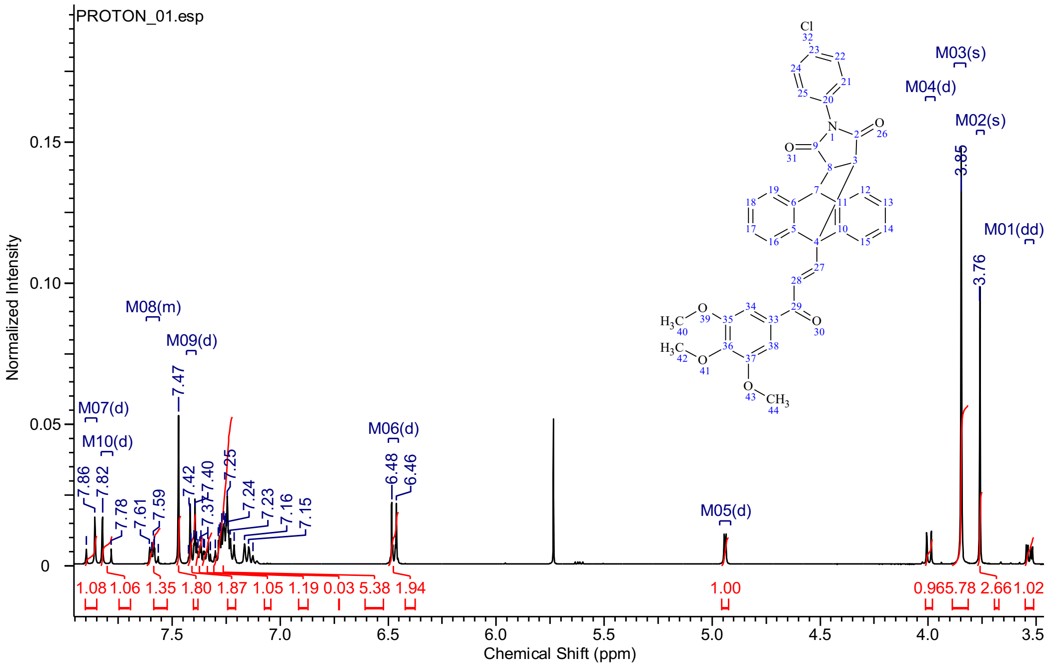

Figure S38: ^{1}H NMR spectrum of (*E*)-13-(4-chlorophenyl)-9-(3-oxo-3-phenylprop-1-en-1-yl)-9,10-dihydro-9,10-[3,4]epipyrroloanthracene-12,14-dione (**25k**)

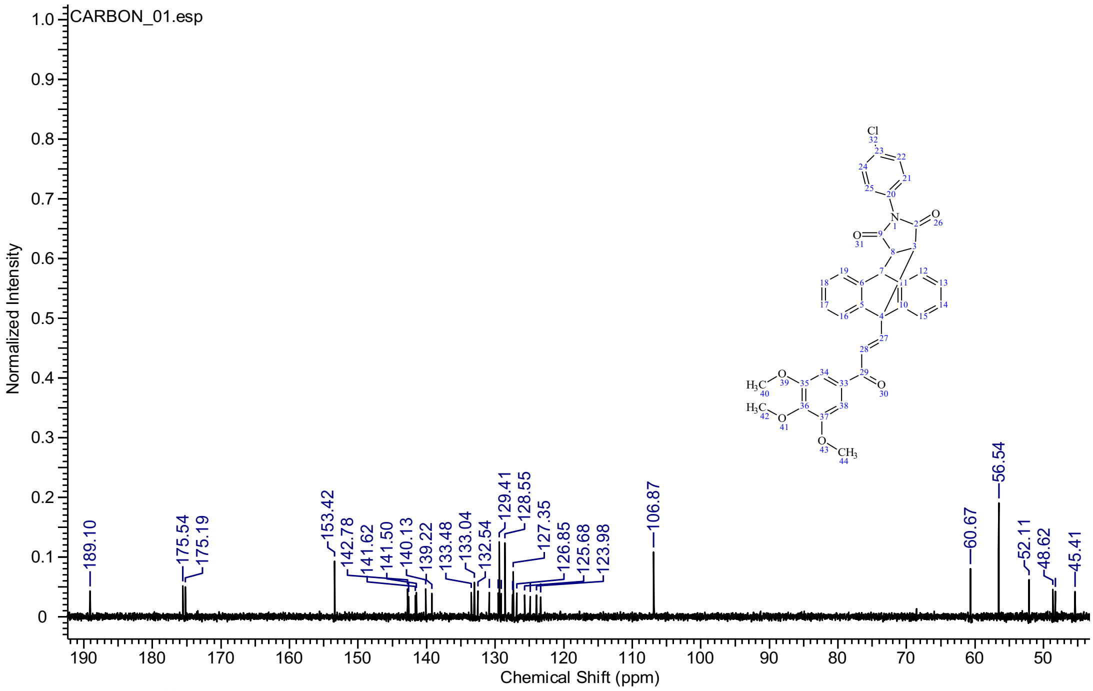

Figure S39 ^{13}C NMR spectrum of (*E*)-13-(4-chlorophenyl)-9-(3-oxo-3-phenylprop-1-en-1-yl)-9,10-dihydro-9,10-[3,4]epipyrroloanthracene-12,14-dione (**25k**)

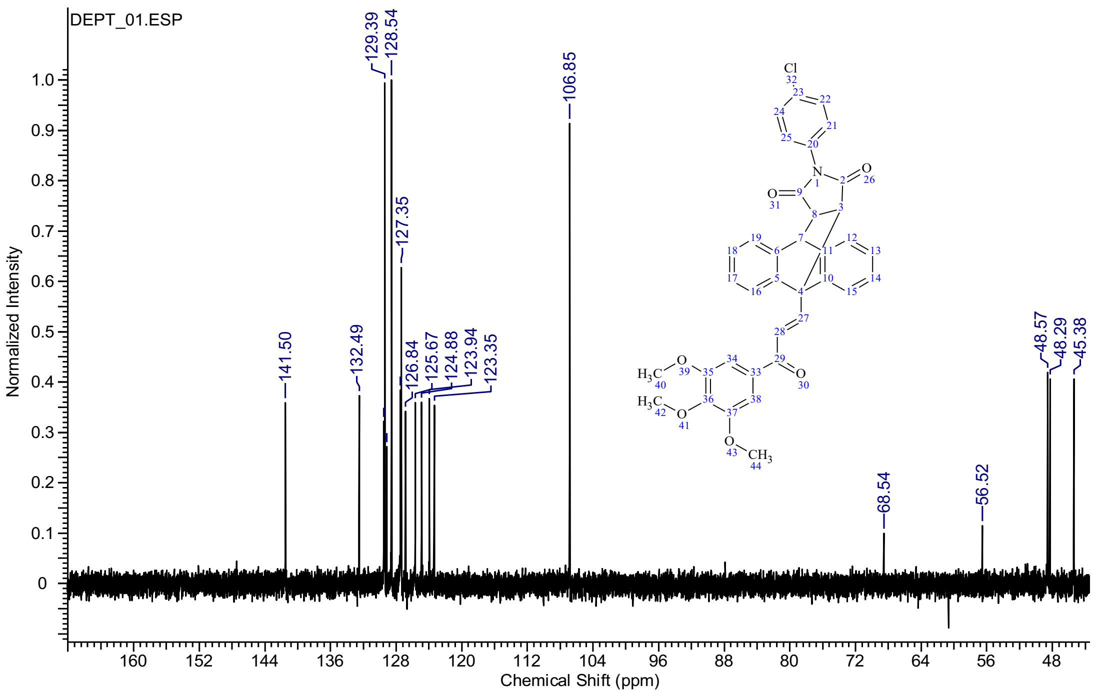

Figure 40 DEPT 90 spectrum of (E)-13-(4-chlorophenyl)-9-(3-oxo-3-phenylprop-1-en-1-yl)-9,10-dihydro-9,10-[3,4]epipyrroloanthracene-12,14-dione (**25k**)

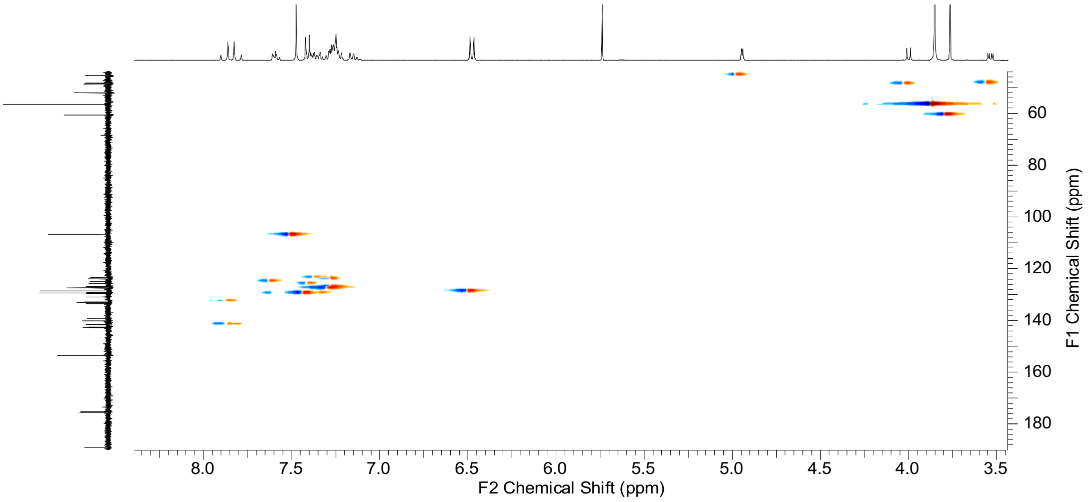

Figure S41: HSQC spectrum of (*E*)-13-(4-chlorophenyl)-9-(3-oxo-3-phenylprop-1-en-1-yl)-9,10-dihydro-9,10-[3,4]epipyrroloanthracene-12,14-dione (**25k**)

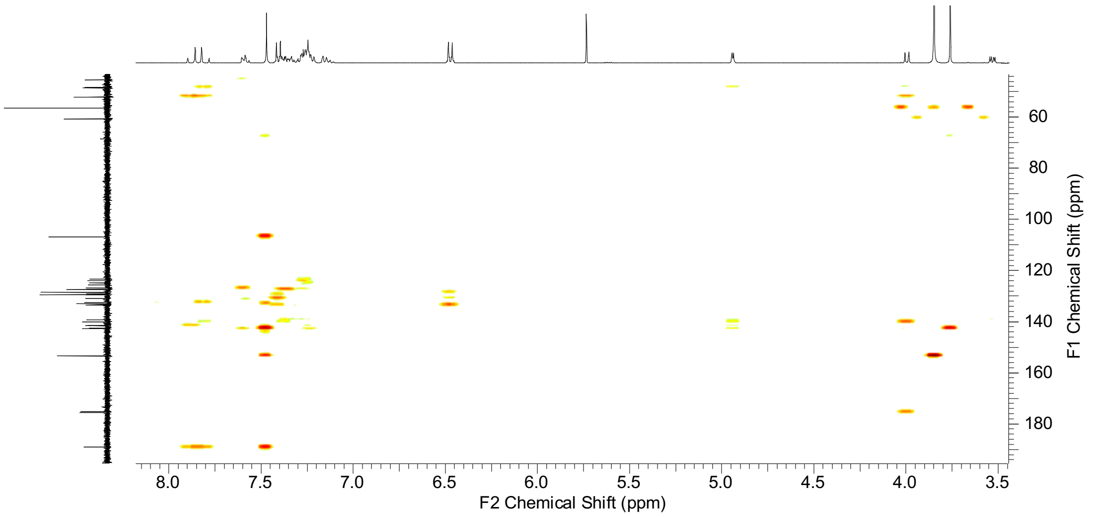

Figure S42: HMBC spectrum of (E)-13-(4-chlorophenyl)-9-(3-oxo-3-phenylprop-1-en-1-yl)-9,10-dihydro-9,10-[3,4]epipyrroloanthracene-12,14-dione (**25k**)

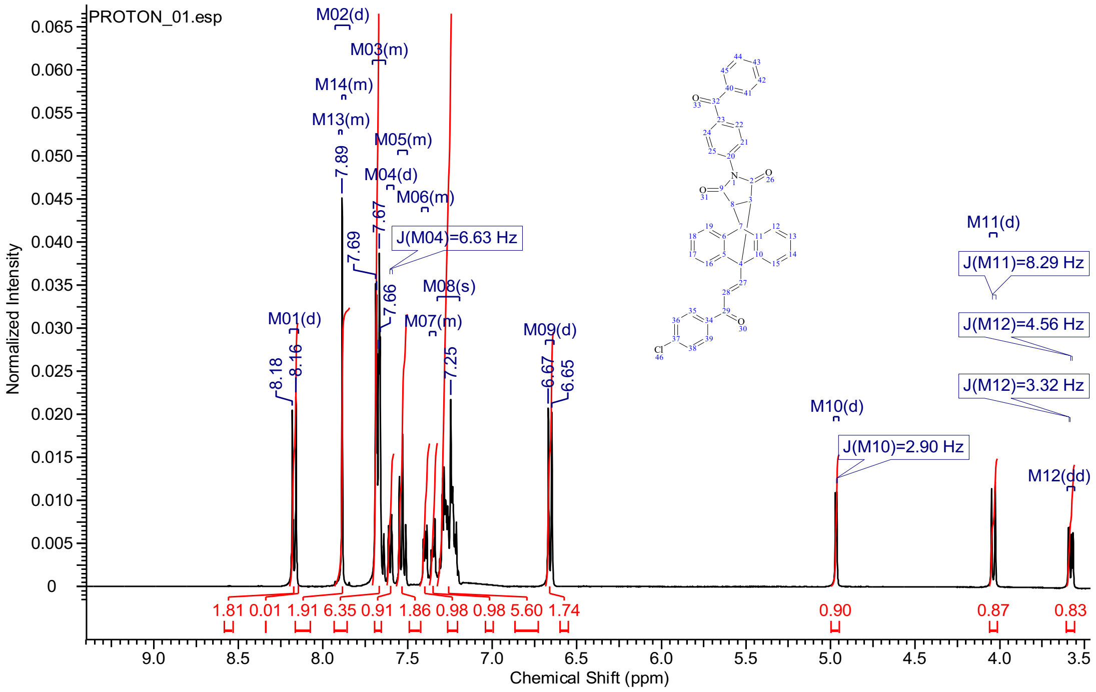

Figure S43: ^{1}H NMR spectrum of (E)-13-(4-benzoylphenyl)-9-(3-(4-chlorophenyl)-3-oxoprop-1-en-1-yl)-9,10-dihydro-9,10-[3,4]epipyrroloanthracene-12,14-dione (**26l**)

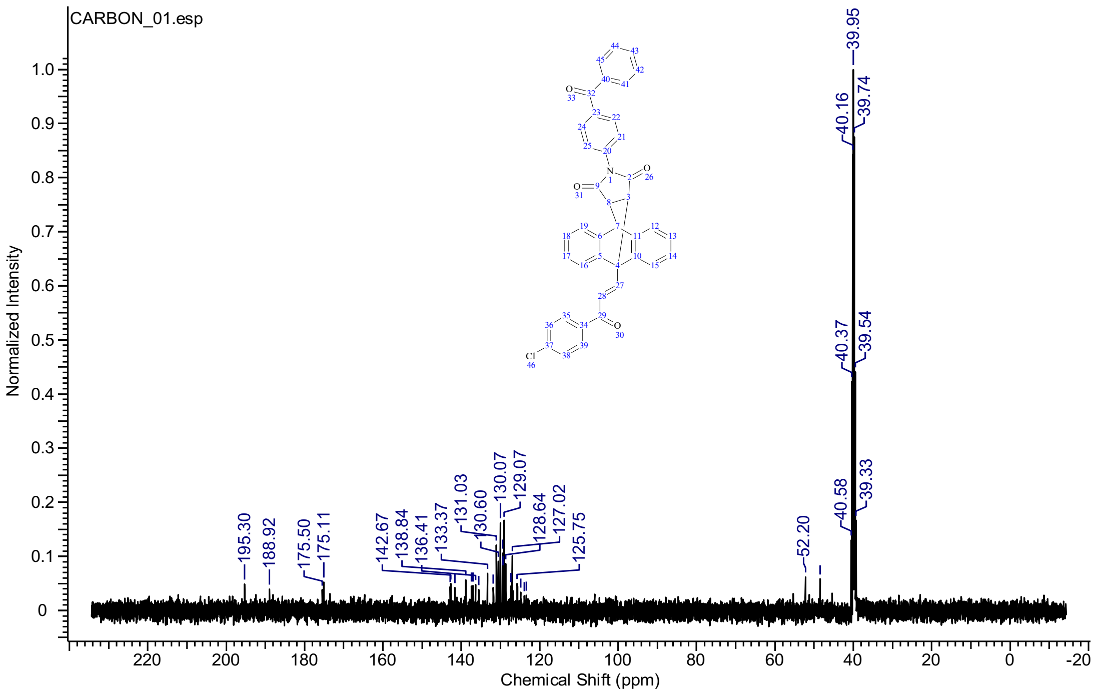

Figure S44: ^{13}C NMR spectrum of (E)-13-(4-benzoylphenyl)-9-(3-(4-chlorophenyl)-3-oxoprop-1-en-1-yl)-9,10-dihydro-9,10-[3,4]epipyrroloanthracene-12,14-dione (**26l**)

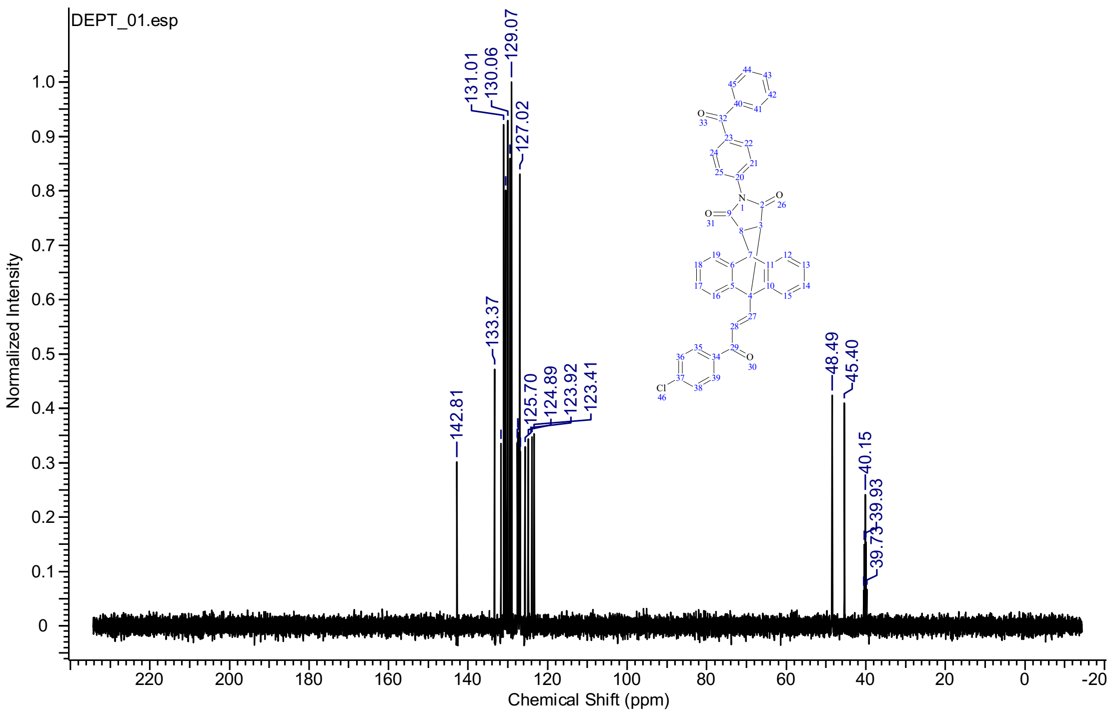

Figure S45: DEPT 90 spectrum of (*E*)-13-(4-benzoylphenyl)-9-(3-(4-chlorophenyl)-3-oxoprop-1-en-1-yl)-9,10-dihydro-9,10-[3,4]epipyrroloanthracene-12,14-dione (**26l**)

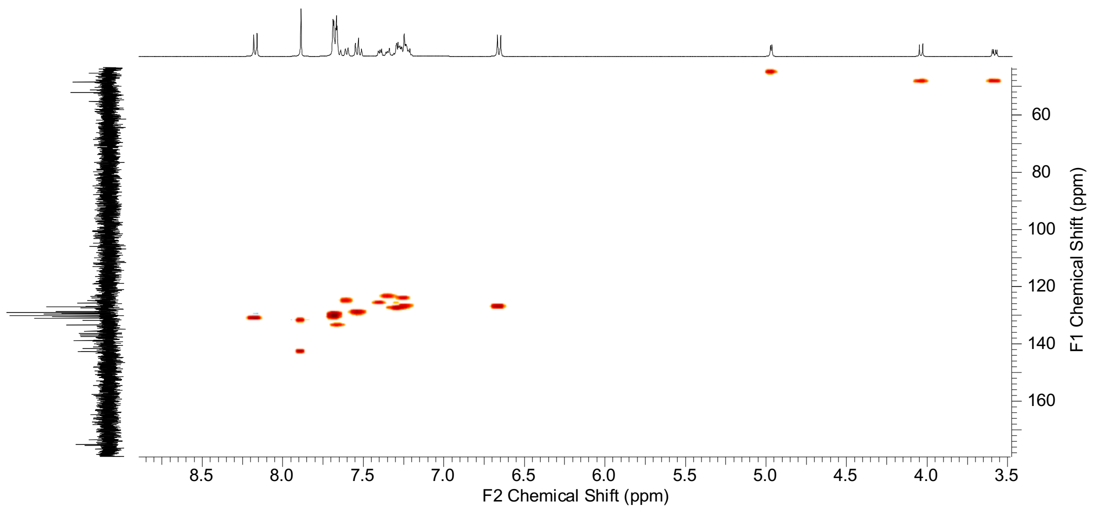

Figure S46: HSQC spectrum of (*E*)-13-(4-benzoylphenyl)-9-(3-(4-chlorophenyl)-3-oxoprop-1-en-1-yl)-9,10-dihydro-9,10-[3,4]epipyrroloanthracene-12,14-dione (**26l**)

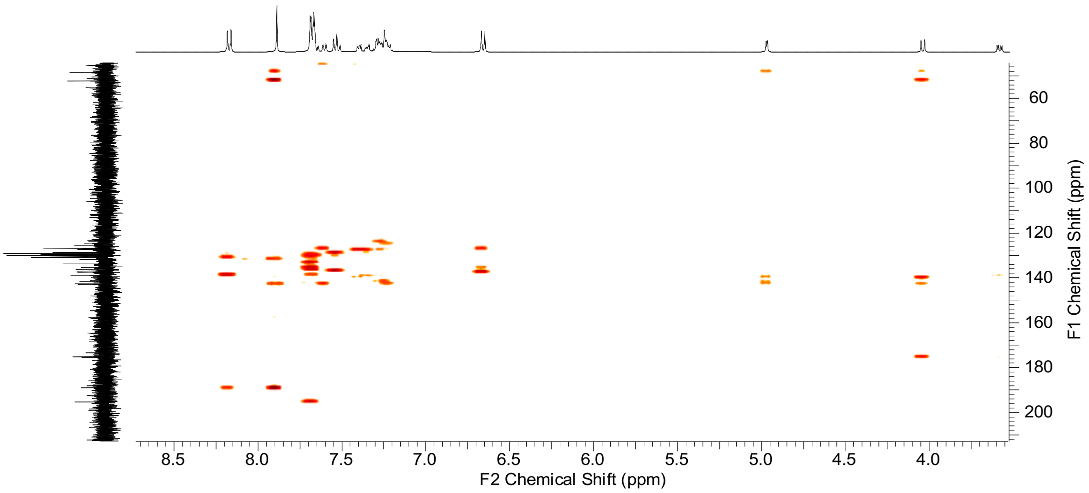

Figure S47: HMBC spectrum of (*E*)-13-(4-benzoylphenyl)-9-(3-(4-chlorophenyl)-3-oxoprop-1-en-1-yl)-9,10-dihydro-9,10-[3,4]epipyrroloanthracene-12,14-dione (**26l**)

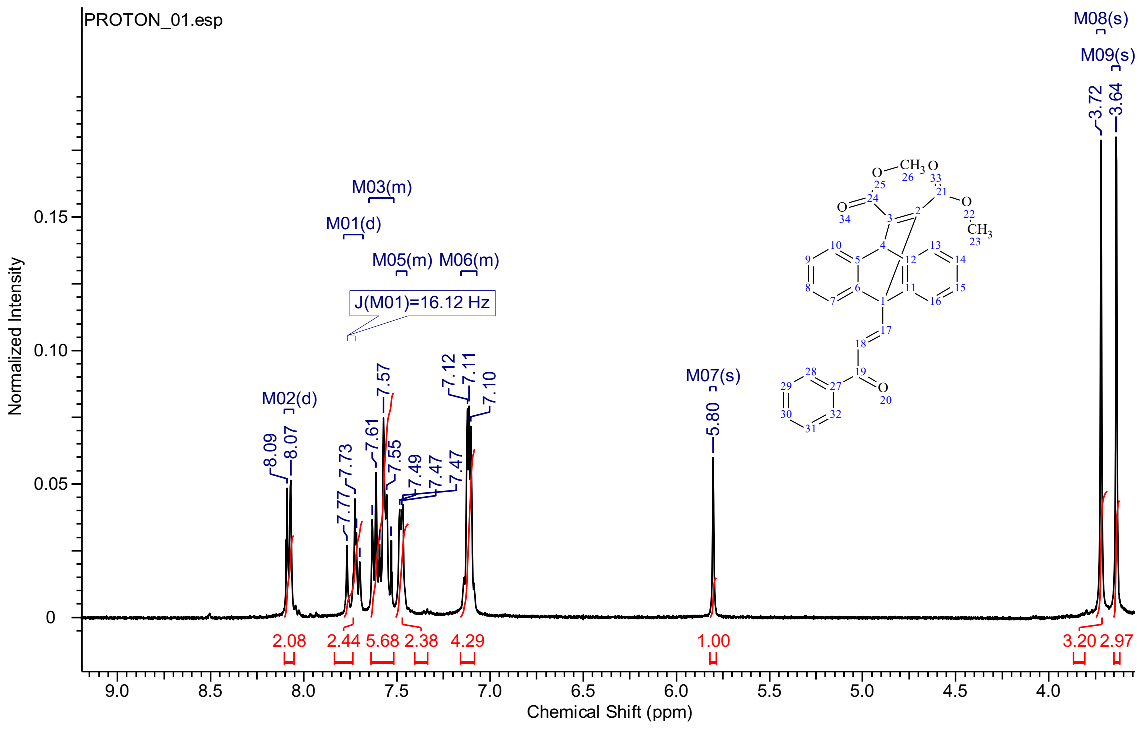

Figure S48: ^{1}H NMR spectrum of dimethyl (*E*)-9-(3-oxo-3-phenylprop-1-en-1-yl)-9,10-dihydro-9,10-ethenoanthracene-11,12-dicarboxylate (**27**)

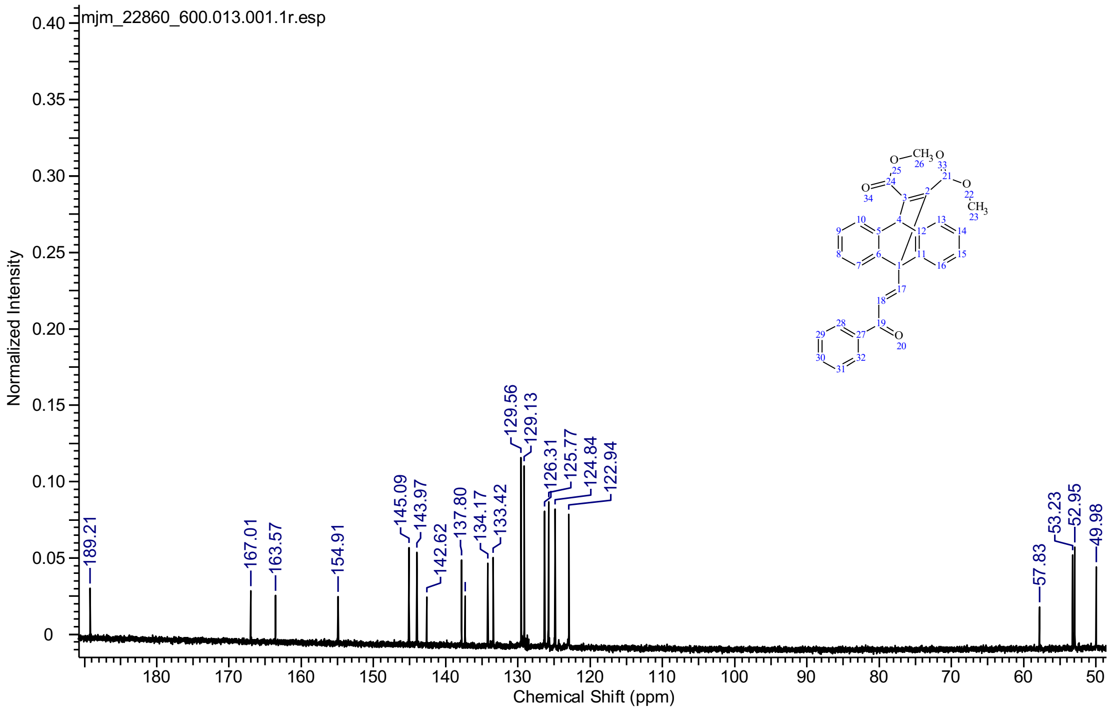

Figure S49: ^{13}C NMR spectrum of dimethyl (*E*)-9-(3-oxo-3-phenylprop-1-en-1-yl)-9,10-dihydro-9,10-ethenoanthracene-11,12-dicarboxylate (**27**)

References

1. Byrne, A.J.; Bright, S.A.; McKeown, J.P.; O'Brien, J.E.; Twamley, B.; Fayne, D.; Williams, D.C.; Meegan, M.J. Design, synthesis and biochemical evaluation of novel ethanoanthracenes and related compounds to target burkitt's lymphoma. *Pharmaceuticals (Basel)* **2020**, *13*.
2. James, J.P.; Ishwar B.K.; More, U.A.; Joshi, S.D. Design, synthesis, molecular modeling, and admet studies of some pyrazoline derivatives as shikimate kinase inhibitors. *Medicinal Chemistry Research* **2018**, *27*, 546-559.
3. Becker, H.D.; Beckers, H.C.; Sandros, K.; Andersson, K. Photochemistry of the anthracene chromophore: Novel isomerization of 1-(9-anthryl)-2-benzoylethylenes. *Tetrahedron Letters* **1985**, *26*, 1589-1592.
4. Velagapudi, N.; Sharma, N; Bandla, R. Synthesis, screening and qsar analysis of chalcone derivatives as potential anti bacterial agents. *International Journal of Pharmaceutical, Chemical and Biological Sciences* **2017**, *7*, 211-218.
5. Mishra, N.; Sasmal, D. Development of selective and reversible pyrazoline based mao-b inhibitors: Virtual screening, synthesis and biological evaluation. *Bioorganic & Medicinal Chemistry Letters* **2011**, *21*, 1969-1973.
6. Buu-Hoi, N.P.; Hoan, N. Some anthracene derivatives of potential biological interest. *Journal of Organic Chemistry* **1951**, *16*, 874-881.
7. Gonzalez, J.J.; Ortega, E.; Rothemund, M.; Gold, M.; Vicente, C.; de Haro, C.; Bautista, D.; Schobert, R.; Ruiz, J. Luminescent gold(i) complexes of 1-pyridyl-3-anthracenylchalcone inducing apoptosis in colon carcinoma cells and antivascular effects. *Inorganic Chemistry* **2019**, *58*, 12954-12963.
8. Kharbanda, C.; Alam, M.S.; Hamid, H.; Javed, K.; Dhulap, A.; Bano, S.; Ali, Y. Antidiabetic effect of novel benzenesulfonylureas as ppar-γ agonists and their anticancer effect. *Bioorganic & Medicinal Chemistry Letters* **2015**, *25*, 4601-4605.
9. Levai, A.; Jeko, J. Synthesis of 1-substituted 3,5-diaryl-2-pyrazolines by the reaction of α,β-unsaturated ketones with hydrazines. *Journal of Heterocyclic Chemistry* **2006**, *43*, 111-115.
